# A target map of clinical combination therapies in oncology: an analysis of clinicaltrials.gov

**DOI:** 10.1007/s12672-023-00758-4

**Published:** 2023-08-21

**Authors:** Jing Yang, Heming Kang, Liyang Lyu, Wei Xiong, Yuanjia Hu

**Affiliations:** 1 Institute of Chinese Medical Sciences, State Key Laboratory of Quality Research in Chinese Medicine, University of Macau, Macao SAR, China; 2 DPM, Faculty of Health Sciences, University of Macau, Room 1049, E12, Macao SAR, 999078 China; 3 Department of Orthopedic Surgery, Affiliated Hospital of Zunyi Medical University, Zunyi, China

**Keywords:** Combination therapy, Targeted therapy, Immuno-oncology therapy, PD-1/PD-L1 inhibitors, Kinase inhibitors, Bispecific antibodies

## Abstract

Combination therapies have taken center stage for cancer treatment, however, there is a lack of a comprehensive portrait to quantitatively map the current clinical combination progress. This study aims to capture clinical combination therapies of the validated FDA-approved new oncology drugs by a macro data analysis and to summarize combination mechanisms and strategies in the context of the existing literature. A total of 72 new molecular entities or new therapeutic biological products for cancer treatment approved by the FDA from 2017 to 2021 were identified, and the data on their related 3334 trials were retrieved from the database of ClinicalTrials.gov. Moreover, these sampled clinical trials were refined by activity status and combination relevance and labeled with the relevant clinical arms and drug combinations, as well as drug targets and target pairs. Combination therapies are increasingly prevalent in clinical trials of new oncology drugs. From retrospective work, existing clinical combination therapies in oncology are driven by different patterns (i.e., rational design and industry trends). The former can be represented by mechanism-based or structure-based combinations, such as targeting different domains of HER2 protein or in-series co-targeting in RAF plus MEK inhibitors. The latter is an empirically driven strategy, including redundant combinations in hot targets, such as PD-1/PD-L1, PI3K, CDK4/6, and PARP. Because of an explosion in the number of clinical trials and the resultant shortage of available patients, it is essential to rationally design drug combinations.

**Supplementary Information:**

The online version contains supplementary material available at 10.1007/s12672-023-00758-4.

## Introduction

We are witnessing the availability of more and newer entities for cancer treatment. At the same time, diverse resistance profiles in cancer have been reported for the single-agent treatments [1, 2]. Combination strategies have certainly been regarded as necessary for the majority of new targeted drugs [3] and many combination therapies have shown durable responses [4]. On the other hand, with the increasing number of new drugs, possible combinations among them are growing exponentially. However, available patients for clinical trials particularly those who meet specific clinical investigation objectives, are relatively limited. So the agents of industry as well as academia have widely expressed the difficulty of recruiting patients and have called for smart designs of clinical combinations to conserve patient resources [4–6]. Before this, it is of great significance to retrospectively summarize existing clinical combination therapies in oncology, especially to understand the combination patterns of target pairs.

Combination mechanisms in clinical treatments are complicated and various. The combination activity has relied on the contributions of the accompanying components [7] according to the synergy effect model [3] and drug sensitivity theory [8]. Many studies have technologically researched clinical anticancer combinations, particularly the combination progress toward specific targets (e.g., programmed cell death protein-1 (PD-1)/programmed cell death ligand 1 (PD-L1) [9, 10], cyclin-dependent kinase 4/6 (CDK4/6) [11], or poly (ADP-ribose) polymerase (PARP) [12]), specific therapies (e.g., immuno-oncology (IO) therapies [13–15]), specific cancer types [16, 17], or even the perspectives of future directions [4, 18]. However, most of these studies mainly focus on explicating the progress by enumerating related combination cases, and there is a lack of quantitative analysis of the interactions between different types of therapies. Thus, this study aims to capture the clinical combination therapies of the newly validated FDA-approved oncology drugs using a macro data analysis and to summarize combination mechanisms and strategies in the context of the existing literature.

## Data and method

**Fig. 1:**
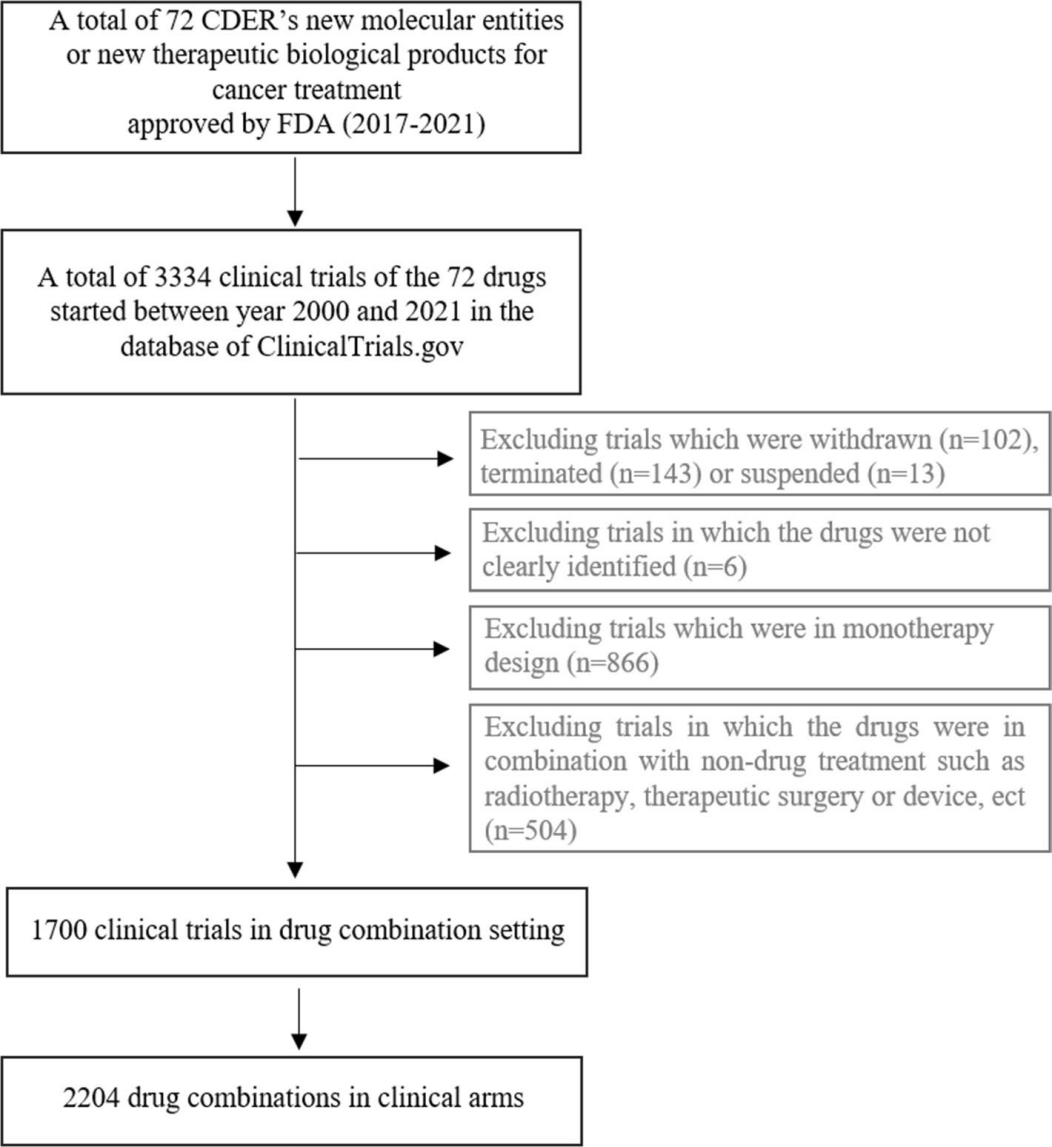
Data processing diagram

This study involved 72 New Molecular Entities (NMEs) or new therapeutic biological products for cancer treatment approved by the FDA from 2017 to 2021 (Supplementary Table 1). The drug data were collected from the US FDA’s official website, and the data of 3334 relevant clinical trials from 2000 to 2021 were retrieved from ClinicalTrials.gov. To extract active drug combination treatments, the following clinical trials were excluded: (1) terminated trials, (2) withdrawn trials, (3) suspended trials, (4) trials in which the drugs were not clearly identified, (5) trials in monotherapy designs, and (6) trials in which the combinations were designed between drug and non-drug treatment, such as radiotherapy, therapeutic surgery or device. Moreover, the drug names were extracted from each arm of these included trials, when the drugs were described in combination use in the same treatment cycle. Meanwhile, the target information for each drug in combination use was identified and tagged from the trial briefings in ClinicalTrials.gov, the Drugbank database, or the websites of pharmaceutical companies. The detailed data processing flow is shown in Fig. 1, while the original data on drug combinations are recorded in Supplementary Table 2. In addition, to further map the registration status for these clinical combinations, the approved combination regimens were collected from the FDA’s website and sorted into Supplementary Table 3. The information on bispecific antibodies (BsAbs) including bispecific T-cell engagers (BiTEs) were highlighted and gathered from ClinicalTrials.gov or the developer’s websites (Supplementary Table 4).

A descriptive statistical analysis was conducted for this study. The number of trials and the rate of monotherapies were calculated using Excel 2016. The raw data for Fig. 3 were also processed using Excel for the formatting as shown in Supplementary Table 2. Then, this formatted data was fed into the Pajek64 software to generate a network with its network function. With the export tool of Pajek64, the matrix of the network was generated and displayed as a heat map (as shown in Fig. 3) using Excel 2016.

## Results and discussion

### The decline of monotherapies

**Fig. 2:**
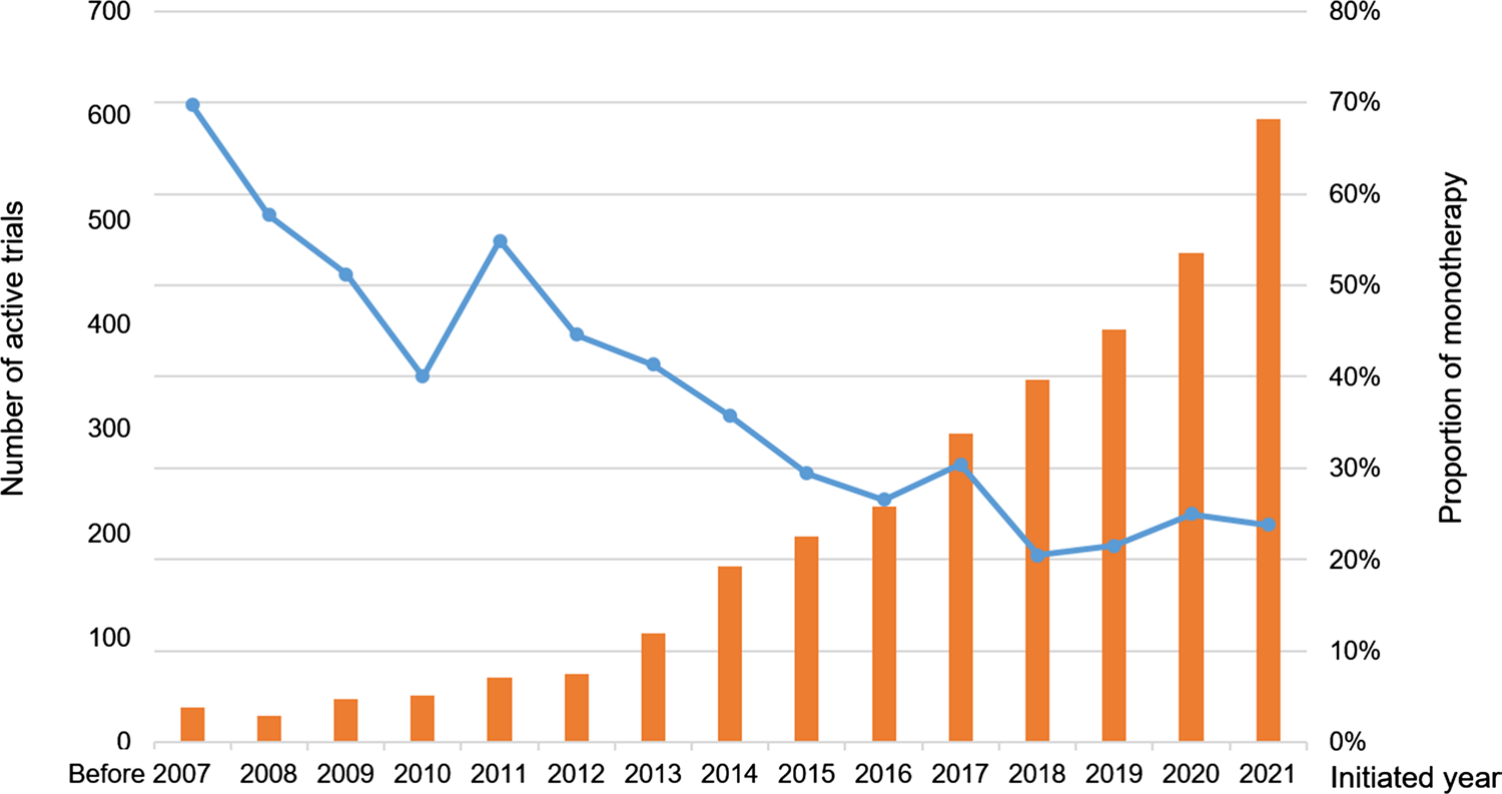
The number of clinical trials initiated yearly and the rate of monotherapies. The bar chart shows that the number of newly initiated trials increased by year for the 72 new oncology drugs approved from 2017 to 2021. However, according to the line bar, the proportions of monotherapy setting of these trials decreased from 70 to 20–30%

The number of yearly initiated trials has increased over time, but the proportions of monotherapy setting have fallen sharply from 70 to 20–30%. (Fig. 2). Expanding efficacy and scope, the combination regime has emerged as a promising strategy in the later stages of the drug development cycle.

### Conventional anticancer regimens combined with targeted therapies

**Fig. 3:**
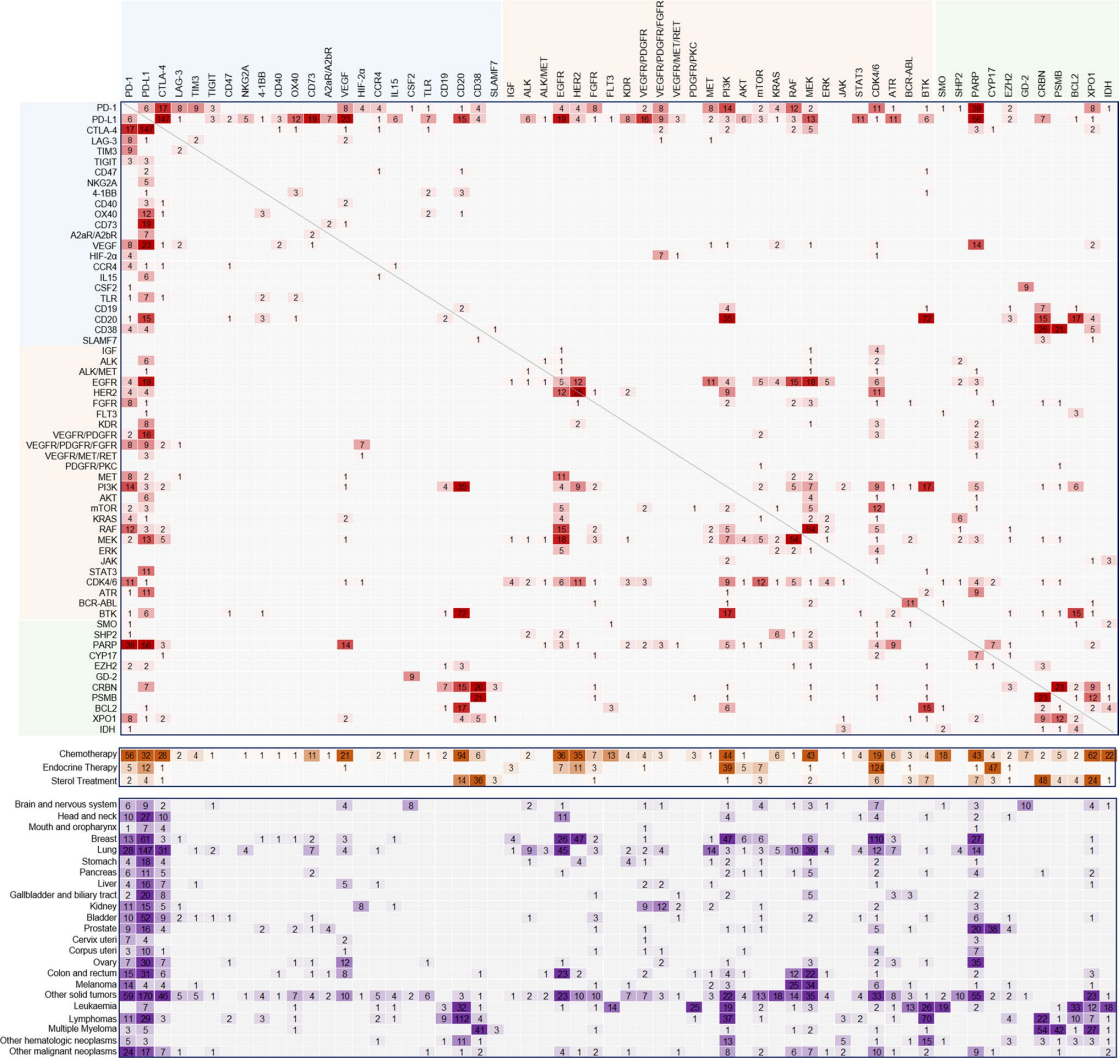
Heat map of target combinations, conventional therapies, and cancer types. These three components are shown in three blue boxes. Targets are grouped into three categories: IO, kinase axis, and miscellaneous, indicated in light blue, yellow, and green, respectively. Only targets with a total count of five or more are shown in this figure, and darker colors of the cells indicate higher combination frequencies

Targeted therapies have achieved revolutionary advances in cancer treatments [19, 20]. The conventional therapies, such as chemotherapy, endocrine therapy and the commonly used steroids, are respectively grouped to show the interactions with targeted regimes. Figure 3 shows that most targeted therapies have been investigated in combination with chemotherapeutic agents. For some therapeutic targets in the treatment of breast or prostate cancer, including PD-1/PD-L1, epithelial growth factor receptor (EGFR), human epidermal growth factor receptor 2 (HER2), phosphatidylinositol-3-kinase (PI3K), CDK4/6, PARP, and cytochrome P450 family 17 (CYP17), their inhibitors are often used in combination with endocrine therapy. Notably, as a common treatment for adverse reactions caused by anticancer reagents [21, 22], steroids are also combined at relatively broad levels with IO or targeted drugs toward CD20, CD38, cereblon (CRBN), exportin 1 (XPO1) and so on. However, the role of steroids on IO regimens is unclear [23–25]. The clinical data of 640 patients with non–small-cell lung cancer (NSCLC) treated with PD-1/PD-L1 inhibitors showed that the baseline use of more than 10 mg of prednisone equivalent daily resulted in poor progression-free survival (PFS) and overall survival (OS) compared to the use of less than 10 mg of prednisone [26]. Similar poor outcomes have been shown for the greater than 20 mg or 10–19 mg of prednisone group compared with the less than 10 mg group. The dose of less than 10 mg of prednisone in this study [26] was regarded as within the range of physiologic adrenal replacement, and the less than 10 mg group was combined with the noncorticosteroid group.

### Combinations in IO therapies

Checkpoint blockades have been the mainstay of IO therapies [27, 28]. Among them, PD-1/PD-L1 inhibitors are dominant in the treatment of hematologic and solid tumors. However, their low clinical response rates in many tumors [29–31] necessitate using combinations to overcome these limitations [10, 32, 33]. Apart from the widely used combination with traditional chemotherapies, PD-1/PD-L1 inhibitors also act with other IO therapies in different patterns. One approach is the dual blockade of immune checkpoints, such as blockers of PD-1/PD-L1 plus inhibitors of cytotoxic T-lymphocyte associated protein 4 (CTLA-4), Lymphocyte activating 3 (LAG-3), T cell immunoglobulin and mucin-domain containing-3 (TIM-3), T cell immunoreceptor with Ig and ITIM domains (TIGIT), CD47 or NKG2A. Among these, the combination of PD-L1 and CTLA-4 has the highest frequency of 147 in all sampled target pairs. Even the combination of PD-1 and PD-L1 inhibitors has a frequency of six, as shown in Fig. 3, which has also been verified and approved by the FDA. Another strategy is to combine decreasing immunosuppression and activating co-stimulatory functions, such as adding anti-PD-L1 therapies to agonists of CD137 (4-1BB), CD40, or OX40. Other analogous assemblies include anti-LAG-3 plus anti-TIM-3, anti–CTLA-4 therapy plus agonists of CD40 or OX40.

Another focus for immunotherapy is to improve the tumor microenvironment (TME) and there are myriad associated molecules, such as the immunosuppressive factors, including adenosine, Endothelial growth factor (VEGF), hypoxia-inducible factor (HIF), or cytokines and chemokines, such as Interleukin-15 (IL15), granulocyte–macrophage colony-stimulating factor (GM-CSF) or CC chemokines, which have also been reviewed with limited efficacy in monotherapy and have been suggested in combination with other treatments [34]. PD-1/PD-L1 inhibitors are still the preferred partners for these microenvironment regulators. In this sense, several relevant target pairs that appear in this heatmap are elaborated below in the context of the literature. For example, adenosine, as an immunosuppressive metabolite, can be regulated by the CD39-CD73-A2aR pathway [35, 36]. It is shown that, except intra-pathway combinations, the nodes in the CD39-CD73-A2aR pathway frequently interact with PD-L1. VEGF also creates immunosuppressive TME [37], and VEGF inhibitors could reprogram this environment into immunostimulatory conditions, which promote the antitumor immunity of PD-1/PD-L1 antibodies [38]. HIF is another factor that induce immunosuppressive TME [39] by allowing tumor cell survival in hypoxic situations [40], which increases angiogenesis (VEGF, IL8, etc.), produces high levels of immunosuppressive cells (Treg, TAM cells, etc.) or upregulates immune checkpoint proteins (PD-L1, CD47, etc.) [41]. CC chemokines and their receptor CCR4 mediated the accumulation of Tregs in tumors, which has been suggested as one resistance mechanism for immune therapies and supports the combination of CCR4 antagonists with other checkpoint inhibitors [42]. Cytokines IL15 is associated with an increase in PD-L1 expression, and its agonists could promote the objective response of anti-PD-1/PD-L1 therapies [43]. GM-CSF (also known as CSF2) has been found to be involved in PD-L1 overexpression through the GM-CSF-STAT3 pathway [44]. Furthermore, GM-CSF is a cytokine that serves as a double-edged sword: too little or too much can promote cancer growth [45]. This explains why few combinations of GM-CSF with PD-1/PD-L1 were observed in the map. In addition, TLR ligands are used in combination to overcome primary or acquired PD-1/PD-L1 inhibitor resistance, relying on their role in activating innate immunity [46, 47].

Other IO targets in this snapshot, including CD19, CD20, CD38 and SLAMF7, have been highlighted for their therapeutic role in hematologic malignancies [48–50]. Their blockers are also frequent in combination with other drugs targeting hematologic tumor dominant targets such as bruton tyrosine kinase (BTK), CRBN, proteasome(PSMB), and B-cell lymphoma 2 (BCL2).

### Combinations of kinase-targeted therapies

**Fig. 4:**
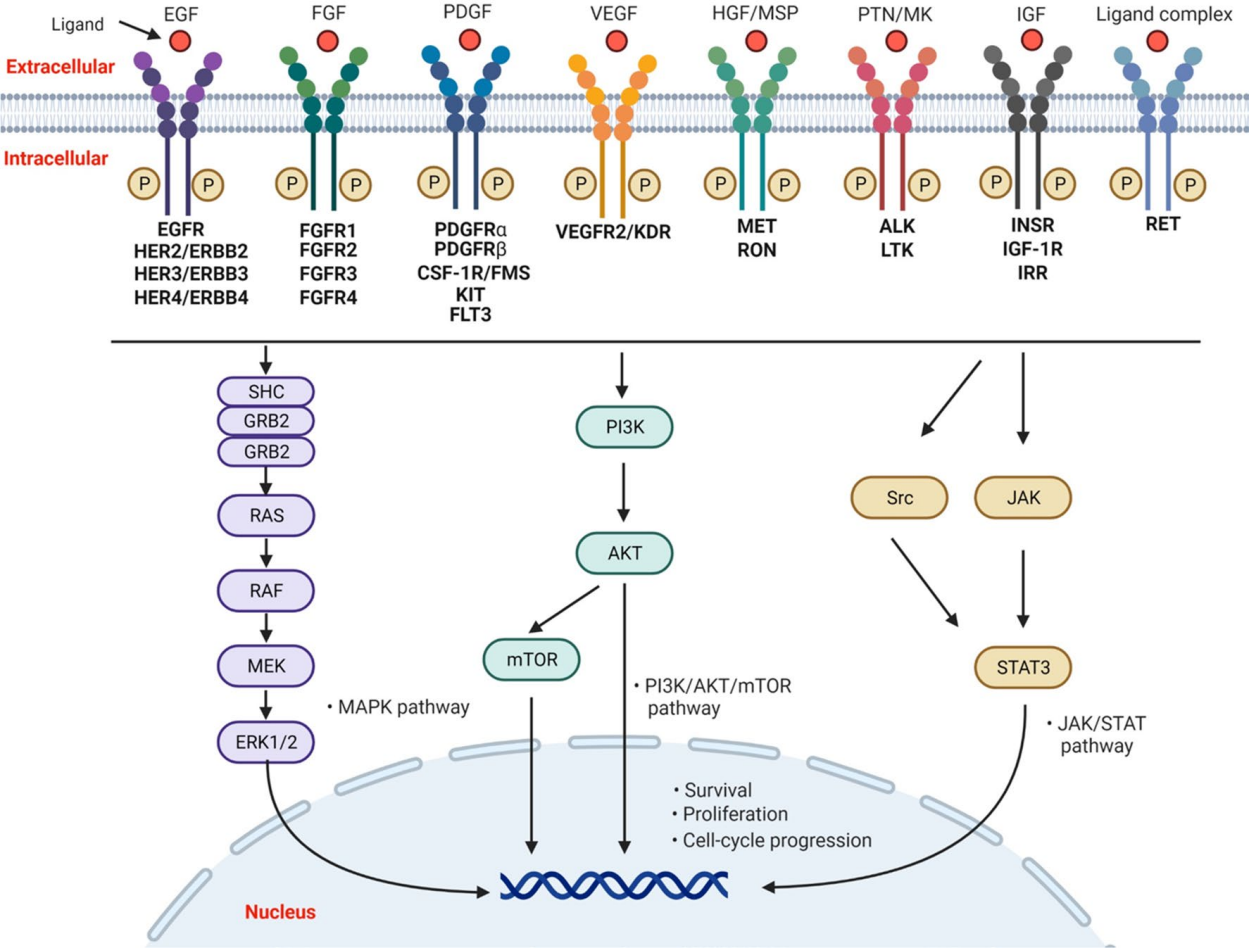
Redundancy between the receptor tyrosine kinase (RTK) signaling pathways (including EGFR, FGFR, PDGFR, VEGFR, MET, ALK, IGF-1R, RET, etc.) and their multiple downstream pathways, covering PI3K/AKT/mTOR, Ras/Raf/MEK/ERK1/2, and JAK/STAT

The kinase inhibitors are another pillar type of successful targeted drugs for cancer treatment that mediate reversible protein phosphorylation to regulate cell survival, proliferation, migration, apoptosis, or other cellular functions [51, 52]. However, the components of this big family exhibit redundancy and crosstalk bioactivities [53–55], which easily produce resistance and stimulate the application of combination regimens [56–58]. A good example, as shown in Fig. 4, is the redundancy between the receptor tyrosine kinase (RTK) signaling pathways (including the ligands and corresponding receptors, such as EGF and EGFR, VEGF and VEGFR, PDGF and PDGFR, or IGF and IGFR) and their multiple downstream pathways, covering PI3K/AKT/mTOR, Ras/Raf/MEK/ERK1/2, JAK/STAT or other pathways [59, 60]. Kinase inhibitors usually have multi-target activities, which induce diverse interactions and safety problems [61]. In this context, combination settings frequently happen between upstream and downstream pathways or even in-series co-targeting the same pathway (for example RAF + MEK inhibitors), as shown in Fig. 3.

Moreover, EGFR, HER2, PI3K, MEK and CDK4/6 play an active role in intra-pathway and inter-pathway combination with other targets, with relatively high frequency in Fig. 3. For instance, the combination of PI3K plus CD20 inhibitors has been approved for the market with a high frequency of 35, as shown in Fig. 3; However, concerns remain, particularly after the FDA withdrew the use of two approved PI3K inhibitors, duvelisib and idelalisib, for hematologic cancer therapy.

Cell-cycle-regulating kinases, such as Cyclin-Dependent Kinases (CDK), also have frequent combinations, as depicted in Fig. 3. For example, CDK4/6 inhibitors have been broadly combined with the targeted drugs toward PD-1/PD-L1, targets in various RTK axes, or miscellaneous targets. CDK4/6 have been regarded as the main driving factor controlling the cell cycle from G1 to S phase, and CDK4/6 genes are widely expressed across various tumors [62]. In addition, ATR has been combined at the times of nine with PARP, another DNA repair related protein [63] to overcome resistance [64].

Notably, kinase inhibitors and immune checkpoint blockades are a great combination. They not only show the high frequency of combinations (Fig. 3) but also include several cases approved to be marketed successfully, that is, PD-1/PD-L1 blockers together with the inhibitors of VEGF, BRAF, HER2, FGFR, MEK, or VEGFR (Supplementary Table 3).

### Combinations against a single target

The cells on the diagonal line in Fig. 3 represent the combination of the two drugs toward a single target. ERBB2 (HER2), EGFR, and BCR-ABL are three typical representatives with relatively high internal combination frequencies of 25, 5, and 11, respectively. First, ERBB2 and EGFR (ERBB1) both belong to the ERBB family, which has an extracellular region with four (I-IV) domains, a membrane-spanning region and an intracellular region with a kinase domain [65]. This structural complexity breeds diverse drugs targeting different domains as well as various combination designs. The FDA-approved and launched combinations of HER2 inhibitors are tucatinib plus trastuzumab and pertuzumab plus trastuzumab. The antibodies pertuzumab and trastuzumab are both targeted at HER2 proteins against different extracellular domains and synergistically reduce the HER2 protein level [66]. The small compound tucatinib binds to the intracellular kinase domain, resulting in the inhibition of HER2 phosphorylation and disturbing downstream MAPK and PI3K signaling. The combination of tucatinib and trastuzumab increases cell apoptosis [67]. Second, a typical example of an internal combination of EGFR inhibitors is the association of antibodies plus small molecules. Osimertinib plus necitumumab and neratinib plus cetuximab, separately target extracellular and intracellular regions of the EGFR. In addition, small entities both target to EGFR are also combined, such as osimertinib and gefitinib in trial NCT03944772, and osimertinib and dacomitinib in trial NCT03755102, to observe the possible improvement after disease progressed on osimertinib treatment. Third, the approach of dual inhibition against a single carcinogenic driver has been implemented for BCR-ABL inhibitors. Engineered for allosteric ABL kinase [68], asciminib was developed to combine with other BCR-ABL inhibitors.

### Miscellaneous targeted therapies

In addition to the IO and kinase targeting treatments described above, other targeted regimes based on various mechanisms are shown in light green in the first blue box in Fig. 3. The first case represents smoothened receptor (SMO) functions in cancer development and progression [69] as a key member of the hedgehog (HH) signaling pathway, which is a conserved pathway in the regulation of cancer cell growth [70]. The SMO inhibitors are designed and identified by their potential to suppress tumors and ensure resistance [71], which promotes their combination with other drugs against PD-1/PD-L1, IDH or other kinase targets. Src homology region 2 domain-containing phosphatase (SHP2) has been found to be a member of protein tyrosine phosphatase, which can remove various tyrosine phosphorylation and thus serves as the upstream hub of PI3K/AKT/mTOR, Ras/Raf/MEK/ERK1/2, and JAK/STAT pathways [72]. The SHP inhibitors are used in combination with some targets of these downstream signalings, such as KARS, RAF, or MEK.

Meanwhile, the enzymes of the PARP family tend to be another pan-cancer treatment target with a wide spectrum of combinations, with chemotherapies, PD-1/PD-L1 blockades, kinases or other miscellaneous targeted therapies. Of these, the combination frequency of PARP inhibitors with PD-1 and PD-L1 blockers was as high as 39 and 56, respectively. Within our pool, the target CYP17A1 involves only abiraterone, which manifests survival and life quality improvement in treating prostate cancer [73], and its combination therapies are mainly with endocrine regimens or other targeted drugs including PARP or EZH2 inhibitors. The EZH2 inhibitors have been broadly assessed for their combination possibilities with PD-1/PD-L1, CD20, PARP, CRBN, and other inhibitors. Although perceived as a novel oncology target, EZH2, one of the epigenetic regulators that repress transcription and functions in multiple biological processes [74], has demonstrated its potency in cell proliferation [75]. In our pool, the GD-2 inhibitors only paired with GM-CSF (also known as CSF2) and this combination has been launched for treatment of high-risk neuroblastomas in bone or bone marrow.

There is also a group of therapeutic targets, particularly against hematologic malignancies, including CRBN, PSMB, BCL2, XPO1 and IDH. As demonstrated in Fig. 3, the drugs designed for these targets are being actively tested their combination with each other in the treatment of hematologic cancers, although some of them, such as XPO1 inhibitors, are expanding their usage in solid tumors. These high frequency trials have yielded combination therapies approved for commercialization, including CRBN inhibitors in combination with the inhibitors of CD19, CD20, CD38, SLAMF7 or PSMB, and PSMB inhibitors plus CD38, or XPO1 inhibitors. Of these, CRBN + CD38, CRBN + PSMB and PSMB + CD38 have frequencies of 26, 23, and 21 respectively, forming a solid iron triangle.

### The rational design of drug combinations

According to our data pool of 72 FDA-approved novel drugs, the number of clinical trials initiated each year grows exponentially. However, it is estimated that the available patients could not support the explosion of oncology clinical trials. Taking melanoma as an example, there are no more than 1,500 melanoma patients who can be recruited globally, which will be far from enough to meet the nearly 600 melanoma trials in progress [4]. A survey showed that the average number of planned patient enrollments per trial fell from 429 patients in 2014 to 129 patients in 2019 [76]. The surge in combination design has exacerbated the problems of insufficient patient recruitment which will lead to the failure of an estimated 40% of clinical cancer trials [4].

As mentioned above, pan-cancer treatments such as anti-PD-1/PD-L1 therapies are being extensively combined with traditional and emerging therapies to address their primary and acquired drug resistance in various cancer types. So far, it has been observed that some of the combination therapies have better clinical benefits than the corresponding monotherapies, for example, the combination of PD-1/PD-L1 and CTLA-4 inhibitors successfully increased response rates and median survival time [77], and has been approved by the FDA for the treatment of melanoma, renal cell carcinoma, hepatocellular carcinoma, etc. But the more anti-PD-1/PD-L1 combinations are still queuing up to be validated, and are likely to end up in the process of recruiting available patients. Similar phenomena also occur in clinical combination plans for drugs against PI3K, PARP, or CDK4/6. Another dynamic group includes targets against hematologic malignancies, which showed broad intra-group combinations.

In addition to the wide range of combinational targets, a large number of clinical studies have been repeated to assess several target pairs. Referring to Supplementary Table 2, the combinations of anti-PD-1/PD-L1 and anti-CTLA-4 therapies amount to 164 records, of which there are 19 combination records based on different drugs for NSCLC treatment. In addition, there are 39 clinical trials have been carried out to evaluate the combination of BTK blockers and anti-CD20 monoclonal antibodies for the treatment of non-Hodgkin’s lymphoma. Other high frequently target pairs include MEK + RAF, PD-1/PD-L1 + PARP, and so on. Therefore, as different drugs against the same target are successively approved, there will be more and more repeated trials assessing the same target pairs because of the “me-too” business policy [4].

Based on the information from this target combination therapy map, it is suggested that the existing studies have done precise and meticulous work in revealing the structure of the target protein, and supported the rationality of the drug combination, such as the combination of different inhibitors targeting different domains of HER2 or EGFR proteins. However, the relationship between proteins, as well as redundancy and crosstalk in biological pathways still needs to be further studied. The current high frequency and broad combination setting with several hot targets also suggests many experience-driven traces and we still need to learn lessons from the failed combination designs, such as the increased toxicity of PI3K/mTOR + MEK inhibitors [78]. In terms of the serious shortage of available patients, and the increasing number of new drugs, a rational combination design with more solid evidence seems more urgent than ever before.

### Combinations of new type drugs

The combination therapies would be good references for the development of BsAbs or BiTE antibodies. Many targets of BsAbs or BiTEs in the clinical stage (Supplementary Table 4) can also be mapped to the combination pairs and anti-PD-1/PD-L1 therapies become the basis for the target selection of BsAbs. Except CD3 based BsAbs, the FDA approved BsAbs in oncology is EGFR x cMET, which is also highly frequently tested in combinations. Another common pair, PD-1 + CTLA-4 was recently generated one BsAb approval in China. However, one of the concerns for BsAbs is how to overcome the safety problems of corresponding combination therapies.

Antibody–Drug Conjugate (ADC) is another type of anticancer drug that integrates targeted and cytotoxic effects in one drug. With more functions in one drug, both BsAbs and ADCs can make it easier to reduce the number of tested drugs and effectively decrease the difficulties in the clinical design of the combinations among three or more drugs with distinctive mechanisms. There are also many combination cases under clinical investigation. For example, amivantamab, binding to extracellular domains of EGFR and MET, in combination with lazertinib targeting the intracellular EGFR kinase domain [79], yielded 36% response for osimertinib-relapsed patients [80]. For ADC drugs against hematologic cancers, dominant targets, such as BCMA, CD19, CD20, CD22, CD33, and CD79B, are often combined with reagents toward CD20, BCL, CRBN, or PSMB, which are all superior in hematologic malignancies. In addition, anti-PD-1/PD-L1 therapies are still commonly chosen as accompanying regimens (Supplementary Table 2). Moreover, the combination of inhibitors binding to different extracellular domains or extracellular and intracellular domains of HER-2 protein are also reproduced in the combination designs of HER-2 targeted ADCs.

So far, few of these mentioned combination therapies are approved for marketing (Supplementary Table 3), and most of them are still in the clinical stage. This scoping review mapped these drug combinations to target pairs, which can mechanically support the future clinical combination strategy for academic or industry research.

### Study limitation

From ClinicalTrial.gov, we collected the clinical trials in of 72 NMEs or new therapeutic biological products for cancer treatment approved by FDA over 5 years (2017–2021) to quantitatively map the target pairs based on these drug combinations. For the data resource, only ClinicalTrial.gov was used to collect the raw data, while there are also other clinical registration platforms, such as WHO International Clinical Trials Registry Platform (ICTRP). Therefore, there could be some trials related to the listed drugs not included in ClinicalTrial.gov.

We included the recent 5 years’ newly approved drugs and retrieved 3334 clinical trials. Some of the drugs approved 5 years before 2017 overlapped targets with the included 72 drugs. Therefore, the frequencies for the same target pairs will be different; However, the trend is estimated to be consistent with the results in this study because of the widespread “me-too” business policies.

In this study, we tried to display the impact of target interactions on drug combinations. There are many other drivers of combination trial choice: (1) the emerging target therapies in combination with the standard of care, for example, belantamab mafodotin administered in combination with standard of care (i.e., VRd treatment including velcade (bortezomib), revlimid (lenalidomide) and dexamethasone) for participants with newly diagnosed multiple myeloma (NCT04091126); (2) the combination of new drugs owned by the same companies, such as, LY3214996 (ERK inhibitor) plus abemaciclib (CDK4/6 inhibitor), both of which are developed by Eli Lilly and Company (NCT04391595). All these are interesting directions to further depict the patterns for drug combination development.

In summary, this work can provide researchers and developers of oncology drug combination design decision-making with information, such as opportunities for white space, and how to avoid redundant designs or combination contraindications.

## Conclusion

This work retrospectively illustrates the macroscopic pattern of therapeutic combinations of recent novel anticancer agents. The rational design of numerous clinical trials in this area also can be partially reflected by aggregated evidence in this map, such as, more combination uses of cross-mechanism reagents, and cautious setting of combinations between PD-1/PD-L1 inhibitors and steroid treatment. More importantly, knowledge of what has been done can help outline the historical trajectory of treatment combinations, with which relevant researchers and developers can be more aware of combinations that make sense but are not often explored (i.e., white space should go).

### Supplementary Information

Supplementary file 1 (DOCX 19 KB)

Supplementary file 2 (DOCX 234 KB)

Supplementary file 3 (DOCX 26 KB)

Supplementary file 4 (DOCX 24 KB)

## Data Availability

The datasets of this study are available at the supplementary material.

## Abbreviations

A2AR: Adenosine 2A receptor
ADC: Antibody–drug conjugates
ALK: Anaplastic lymphoma kinase
BCL2: B-cell lymphoma 2
BiTEs: Bispecific T-cell engagers
BsAbs: Bispecific antibodies
BTK: Bruton tyrosine kinase
CDK4/6: Cyclin-dependent kinase 4/6
CDER: The center for drug evaluation and research
CSF2: Colony-stimulating factor 2
CTLA-4: Cytotoxic T-lymphocyte associated protein 4
CYP17: Cytochrome P450 Family 17
EGF: Epithelial growth factor
EGFR: Epithelial growth factor receptor
FGFR: Fibroblast growth factor receptor
GM-CSF: Granulocyte–macrophage colony-stimulating factor
HER2: Human epidermal growth factor receptor 2
HIF: Hypoxia-inducible factor
IGF-1R: Type 1 insulin-like growth factor receptor
IL15: Interleukin-15
LAG3: Lymphocyte activating 3
MAPK: Mitogen-activated protein kinase
MET: Mesenchymal epithelial transition factor
mTOR: Mammalian target of rapamycin
NMEs: New molecular entities
PARP: Poly (ADP-ribose) polymerase
PD-1/PD-L1: Programmed cell death protein-1 (PD-1)/Programmed cell death ligand 1 (PD-L1)
PDGFR: Platelet-derived growth factor receptor
PI3K: Phosphatidylinositol-3-kinase
PSMB: Proteasome
RET: Rearranged during transfection
SHP2: Src homology region 2 domain-containing phosphatase
SLAMF7: SLAM family member 7
TAM: Tumor-associated macrophage
TIGIT: T cell immunoreceptor with Ig and ITIM domains
TIM-3: T cell immunoglobulin and mucin-domain containing-3
Treg: Regulatory T cell
VEGF: Endothelial growth factor
VEGFR: Vascular endothelial growth factor
XPO1: Exportin 1
