# Supplementary material for: A target map of clinical combination therapies in oncology: an analysis of clinicaltrials.gov
Source: Discov Oncol. 2023 Aug 21;14:151. doi: 10.1007/s12672-023-00758-4 (PMC10441974; doi:10.1007/s12672-023-00758-4)
Supplement: Supplementary file 1 — (DOCX 19 KB) [file 12672_2023_758_MOESM1_ESM.docx]

Supplementary Table 1 72 New Molecular Entities (NMEs) or new therapeutic biological products for cancer treatment approved by FDA from 2017 to 2021

| **Drug name** | **Active Ingredient** | **Drug Target** | **Drug Type** | **First approval date** |
| --- | --- | --- | --- | --- |
| Aliqopa | copanlisib | PI3K | Small Molecule | 2017-9-14 |
| Alunbrig | brigatinib | ALK/EGFR | Small Molecule | 2017-4-28 |
| Bavencio | avelumab | PD-L1 | Biological products | 2017-3-23 |
| Besponsa | inotuzumab ozogamicin | CD22/DNA | Biological products | 2017-8-17 |
| Calquence | acalabrutinib | BTK | Small Molecule | 2017-10-31 |
| Idhifa | enasidenib | IDH | Small Molecule | 2017-8-1 |
| Imfinzi | durvalumab | PD-L1 | Biological products | 2017-5-1 |
| Kisqali | ribociclib | CDK4/CDK6 | Small Molecule | 2017-3-13 |
| Nerlynx | neratinib | EGFR | Small Molecule | 2017-7-17 |
| Rydapt | midostaurin | PKC/PDGFR | Small Molecule | 2017-4-28 |
| Verzenio | abemaciclib | CDK4/CDK6 | Small Molecule | 2017-9-28 |
| Zejula | niraparib | PARP | Small Molecule | 2017-3-27 |
| Asparlas | calaspargase pegol-mknl | L-asparagine | Biological products | 2018-12-20 |
| Braftovi | encorafenib | BRAF/CCND1 | Small Molecule | 2018-6-27 |
| Copiktra | duvelisib | PI3K | Small Molecule | 2018-9-24 |
| Daurismo | glasdegib | SMO | Small Molecule | 2018-11-21 |
| Elzonris | tagraxofusp-erzs | CD123 | Biological products | 2018-12-21 |
| Erleada | apalutamide | AR | Small Molecule | 2018-2-14 |
| Gamifant | emapalumab-lzsg | IFNG | Biological products | 2018-11-20 |
| Libtayo | cemiplimab-rwlc | PD-1 | Biological products | 2018-9-28 |
| Lorbrena | lorlatinib | ALK | Small Molecule | 2018-11-2 |
| Lumoxiti | moxetumomab pasudotox-tdfk | CD22/DNA | Biological products | 2018-9-13 |
| Lutathera | lutetium Lu 177 dotatate | SSTR | Small Molecule | 2018-1-26 |
| Mektovi | binimetinib | MEK | Small Molecule | 2018-6-27 |
| Poteligeo | mogamulizumab-kpkc | CCR4 | Biological products | 2018-8-8 |
| Talzenna | talazoparib | PARP | Small Molecule | 2018-10-16 |
| Tibsovo | ivosidenib | IDH | Small Molecule | 2018-7-20 |
| Vitrakvi | larotrectinib | NTRK1/NTRK2/NTRK3 | Small Molecule | 2018-11-26 |
| Vizimpro | dacomitinib | EGFR | Small Molecule | 2018-9-27 |
| Xospata | gilteritinib | FLT3 | Small Molecule | 2018-11-28 |
| Balversa | erdafitinib | FGFR | Small Molecule | 2019-4-12 |
| Brukinsa | zanubrutinib | BTK | Small Molecule | 2019-11-14 |
| Enhertu | fam-trastuzumab deruxtecan-nxki | HER2/TOP1 | Biological products | 2019-12-20 |
| INREBIC | fedratinib | JAK | Small Molecule | 2019-8-16 |
| Nubeqa | darolutamide | AR | Small Molecule | 2019-7-30 |
| Padcev | enfortumab vedotin-ejfv | NECTIN4/Tubulin | Biological products | 2019-12-18 |
| Piqray | alpelisib | PI3K | Small Molecule | 2019-5-24 |
| Polivy | polatuzumab vedotin-piiq | CD79B/Tubulin | Biological products | 2019-6-10 |
| Rozlytrek | entrectinib | NTRK1/NTRK2/NTRK3 | Small Molecule | 2019-8-15 |
| Turalio | pexidartinib | CSF1R/PDGFR | Small Molecule | 2019-8-2 |
| Xpovio | selinexor | XPO1 | Small Molecule | 2019-7-3 |
| Ayvakit | avapritinib | KIT | Small Molecule | 2020-1-9 |
| Blenrep | belantamab mafodotin-blmf | BCMA/Tubulin | Biological products | 2020-8-5 |
| Danyelza | naxitamab | GD-2 | Biological products | 2020-11-25 |
| Gavreto | pralsetinib | RET | Small Molecule | 2020-9-4 |
| Koselugo | selumetinib | MEK | Small Molecule | 2020-4-10 |
| Margenza | margetuximab | HER2 | Biological products | 2020-12-16 |
| Monjuvi | tafasitamab-cxix | CD19 | Biological products | 2020-7-31 |
| Orgovyx | relugolix | GNRHR | Small Molecule | 2020-12-18 |
| Pemazyre | pemigatinib | FGFR | Small Molecule | 2020-4-17 |
| Qinlock | ripretinib | VEGFR/PDGFR/RAF | Small Molecule | 2020-5-15 |
| Retevmo | selpercatinib | RET | Small Molecule | 2020-5-8 |
| Sarclisa | isatuximab | CD38 | Biological products | 2020-3-2 |
| Tabrecta | capmatinib | MET | Small Molecule | 2020-5-6 |
| Tazverik | tazemetostat | EZH2 | Small Molecule | 2020-1-23 |
| Trodelvy | sacituzumab govitecan-hziy | Trop-2/TOP1 | Biological products | 2020-4-22 |
| Tukysa | tucatinib | HER2 | Small Molecule | 2020-4-17 |
| Zepzelca | lurbinectedin | DNA | Small Molecule | 2020-6-15 |
| Scemblix | asciminib | BCR-ABL | Small Molecule | 2021-10-29 |
| Tivdak | tisotumab vedotin-tftv | TF/Tubulin | Biological products | 2021-9-20 |
| Exkivity | mobocertinib | EGFR | Small Molecule | 2021-9-15 |
| Welireg | belzutifan | HIF-2 | Small Molecule | 2021-8-13 |
| Rylaze | asparaginase erwinia chrysanthemi | L-asparagine | Biological products | 2021-6-30 |
| Truseltiq | infigratinib | FGFR | Small Molecule | 2021-5-28 |
| Lumakras | sotorasib | KRAS | Small Molecule | 2021-5-28 |
| Rybrevant | amivantamab-vmjw | EGFR/MET | Biological products | 2021-5-21 |
| Zynlonta | loncastuximab tesirine-lpyl | CD19/DNA | Biological products | 2021-4-23 |
| [Jemperli](http://www.accessdata.fda.gov/scripts/cder/daf/index.cfm?event=overview.process&varApplNo=761174) | dostarlimab-gxly | PD-1 | Biological products | 2021-4-22 |
| Fotivda | tivozanib | VEGFR/PDGFR | Small Molecule | 2021-3-10 |
| Pepaxto | melphalan flufenamide | DNA | Small Molecule | 2021-2-26 |
| Ukoniq | umbralisib | PI3K | Small Molecule | 2021-2-5 |
| Tepmetko | tepotinib | MET | Small Molecule | 2021-2-3 |
