# Supplementary material for: A target map of clinical combination therapies in oncology: an analysis of clinicaltrials.gov
Source: Discov Oncol. 2023 Aug 21;14:151. doi: 10.1007/s12672-023-00758-4 (PMC10441974; doi:10.1007/s12672-023-00758-4)
Supplement: Supplementary file 2 — (DOCX 234 KB) [file 12672_2023_758_MOESM2_ESM.docx]

Supplementary Table 2 The combination setting of clinical trials related to the 72 new oncology drugs approved by FDA

| NCT ID | Arm ID | Drug name | Target  /treatment type | Cancer type |
| --- | --- | --- | --- | --- |
| NCT00093600 | NCT00093600-0 | Daunorubicin | chemotherapy | Leukaemia |
| NCT00093600 | NCT00093600-0 | Cytarabine | chemotherapy | Leukaemia |
| NCT00299494 | NCT00299494-0 | Inotuzumab Ozogamicin | CD22/DNA | Lymphomas |
| NCT00299494 | NCT00299494-0 | Rituximab | CD20 | Lymphomas |
| NCT00300781 | NCT00300781-0 | Neratinib | EGFR | Breast cancer |
| NCT00300781 | NCT00300781-0 | Trastuzumab | HER2 | Breast cancer |
| NCT00398567 | NCT00398567-0 | Neratinib | EGFR | Breast cancer |
| NCT00398567 | NCT00398567-0 | Trastuzumab | HER2 | Breast cancer |
| NCT00445458 | NCT00445458-0 | Neratinib | EGFR | Breast cancer |
| NCT00445458 | NCT00445458-0 | Paclitaxel | chemotherapy | Breast cancer |
| NCT00563147 | NCT00563147-0 | Tivozanib | VEGFR/PDGFR | Kidney cancer |
| NCT00563147 | NCT00563147-0 | Temsirolimus | mTOR | Kidney cancer |
| NCT00600496 | NCT00600496-1 | Selumetinib | MEK | Other solid tumors |
| NCT00600496 | NCT00600496-1 | Docetaxel | chemotherapy | Other solid tumors |
| NCT00600496 | NCT00600496-2 | Selumetinib | MEK | Other solid tumors |
| NCT00600496 | NCT00600496-2 | Dacarbazine | chemotherapy | Other solid tumors |
| NCT00600496 | NCT00600496-3 | Selumetinib | MEK | Other solid tumors |
| NCT00600496 | NCT00600496-3 | Erlotinib | EGFR | Other solid tumors |
| NCT00600496 | NCT00600496-4 | Selumetinib | MEK | Other solid tumors |
| NCT00600496 | NCT00600496-4 | Temsirolimus | mTOR | Other solid tumors |
| NCT00651261 | NCT00651261-0 | Chemotherapy | chemotherapy | Leukaemia |
| NCT00651261 | NCT00651261-0 | Midostaurin | PKC/PDGFR | Leukaemia |
| NCT00660153 | NCT00660153-0 | Tivozanib | VEGFR/PDGFR | Colon and rectum cancers |
| NCT00660153 | NCT00660153-0 | Folfox6 | chemotherapy | Colon and rectum cancers |
| NCT00671034 | NCT00671034-0 | Calaspargase | L-asparagine | Leukaemia |
| NCT00671034 | NCT00671034-0 | Chemotherapy | chemotherapy | Leukaemia |
| NCT00706030 | NCT00706030-0 | Neratinib | EGFR | Breast cancer |
| NCT00706030 | NCT00706030-0 | Vinorelbine | chemotherapy | Breast cancer |
| NCT00717340 | NCT00717340-0 | Tivozanib | VEGFR/PDGFR | Breast cancer |
| NCT00717340 | NCT00717340-0 | Paclitaxel | chemotherapy | Breast cancer |
| NCT00724971 | NCT00724971-0 | Inotuzumab Ozogamicin | CD22/DNA | Lymphomas |
| NCT00724971 | NCT00724971-0 | Rituximab | CD20 | Lymphomas |
| NCT00728390 | NCT00728390-0 | Dacomitinib | EGFR | lung cancers |
| NCT00728390 | NCT00728390-0 | Figitumumab | IGF | lung cancers |
| NCT00741260 | NCT00741260-0 | Neratinib | EGFR | Breast cancer |
| NCT00741260 | NCT00741260-0 | Capecitabine | chemotherapy | Breast cancer |
| NCT00768469 | NCT00768469-0 | Neratinib | EGFR | Other solid tumors |
| NCT00768469 | NCT00768469-0 | Paclitaxel | chemotherapy | Other solid tumors |
| NCT00819546 | NCT00819546-0 | Everolimus | mTOR | Leukaemia |
| NCT00819546 | NCT00819546-0 | Midostaurin | PKC/PDGFR | Leukaemia |
| NCT00838539 | NCT00838539-0 | Neratinib | EGFR | Other solid tumors |
| NCT00838539 | NCT00838539-0 | Temsirolimus | mTOR | Other solid tumors |
| NCT00867087 | NCT00867087-0 | Inotuzumab Ozogamicin | CD22/DNA | Lymphomas |
| NCT00867087 | NCT00867087-0 | Rituximab | CD20 | Lymphomas |
| NCT00878709 | NCT00878709-0 | Neratinib | EGFR | Breast cancer |
| NCT00878709 | NCT00878709-0 | Trastuzumab | HER2 | Breast cancer |
| NCT00890825 | NCT00890825-0 | Selumetinib | MEK | lung cancers |
| NCT00890825 | NCT00890825-0 | Docetaxel | chemotherapy | lung cancers |
| NCT00915018 | NCT00915018-0 | Neratinib | EGFR | Breast cancer |
| NCT00915018 | NCT00915018-0 | Paclitaxel | chemotherapy | Breast cancer |
| NCT00936221 | NCT00936221-0 | Selumetinib | MEK | Melanoma |
| NCT00936221 | NCT00936221-0 | Dacarbazine | chemotherapy | Melanoma |
| NCT00958724 | NCT00958724-0 | Neratinib | EGFR | Other solid tumors |
| NCT00958724 | NCT00958724-0 | Vinorelbine | chemotherapy | Other solid tumors |
| NCT01008150 | NCT01008150-1 | Paclitaxel | chemotherapy | Breast cancer |
| NCT01008150 | NCT01008150-1 | Neratinib | EGFR | Breast cancer |
| NCT01008150 | NCT01008150-2 | Paclitaxel | chemotherapy | Breast cancer |
| NCT01008150 | NCT01008150-2 | Trastuzumab | HER2 | Breast cancer |
| NCT01008150 | NCT01008150-2 | Neratinib | EGFR | Breast cancer |
| NCT01021748 | NCT01021748-0 | Mk2206 | AKT | Other solid tumors |
| NCT01021748 | NCT01021748-0 | Selumetinib | MEK | Other solid tumors |
| NCT01055496 | NCT01055496-1 | Cyclophosphamide | chemotherapy | Lymphomas |
| NCT01055496 | NCT01055496-1 | Inotuzumab Ozogamicin | CD22/DNA | Lymphomas |
| NCT01055496 | NCT01055496-1 | Vincristine | chemotherapy | Lymphomas |
| NCT01055496 | NCT01055496-1 | Prednisone | Steroid Treatment | Lymphomas |
| NCT01055496 | NCT01055496-1 | Rituximab | CD20 | Lymphomas |
| NCT01055496 | NCT01055496-2 | Gemcitabine | chemotherapy | Lymphomas |
| NCT01055496 | NCT01055496-2 | Platinum-Based Chemotherapy | chemotherapy | Lymphomas |
| NCT01055496 | NCT01055496-2 | Inotuzumab Ozogamicin | CD22/DNA | Lymphomas |
| NCT01055496 | NCT01055496-2 | Dexamethasone | Steroid Treatment | Lymphomas |
| NCT01055496 | NCT01055496-2 | Rituximab | CD20 | Lymphomas |
| NCT01058655 | NCT01058655-0 | Everolimus | mTOR | Stomach cancer |
| NCT01058655 | NCT01058655-0 | Tivozanib | VEGFR/PDGFR | Stomach cancer |
| NCT01061749 | NCT01061749-0 | Selumetinib | MEK | Other solid tumors |
| NCT01061749 | NCT01061749-0 | Cixutumumab | IGF | Other solid tumors |
| NCT01084252 | NCT01084252-0 | Isatuximab | CD38 | Other hematologic Neoplasms |
| NCT01084252 | NCT01084252-0 | Dexamethasone | Steroid Treatment | Other hematologic Neoplasms |
| NCT01093573 | NCT01093573-0 | Midostaurin | PKC/PDGFR | Leukaemia |
| NCT01093573 | NCT01093573-0 | Azacitidine | chemotherapy | Leukaemia |
| NCT01111825 | NCT01111825-0 | Temsirolimus | mTOR | Breast cancer |
| NCT01111825 | NCT01111825-0 | Neratinib | EGFR | Breast cancer |
| NCT01116271 | NCT01116271-0 | Selumetinib | MEK | Colon and rectum cancers |
| NCT01116271 | NCT01116271-0 | Irinotecan | chemotherapy | Colon and rectum cancers |
| NCT01121575 | NCT01121575-0 | Crizotinib | ALK/MET | lung cancers |
| NCT01121575 | NCT01121575-0 | Dacomitinib | EGFR | lung cancers |
| NCT01128842 | NCT01128842-0 | Neratinib | EGFR | Other solid tumors |
| NCT01128842 | NCT01128842-0 | Capecitabine | chemotherapy | Other solid tumors |
| NCT01130662 | NCT01130662-0 | Decitabine | chemotherapy | Leukaemia |
| NCT01130662 | NCT01130662-0 | Midostaurin | PKC/PDGFR | Leukaemia |
| NCT01134575 | NCT01134575-0 | Inotuzumab Ozogamicin | CD22/DNA | Leukaemia |
| NCT01134575 | NCT01134575-0 | Rituximab | CD20 | Leukaemia |
| NCT01160718 | NCT01160718-0 | Fulvestrant | Endocrine Therapy | Breast cancer |
| NCT01160718 | NCT01160718-0 | Selumetinib | MEK | Breast cancer |
| NCT01161550 | NCT01161550-0 | Cladribine | chemotherapy | Leukaemia |
| NCT01161550 | NCT01161550-0 | Midostaurin | PKC/PDGFR | Leukaemia |
| NCT01174888 | NCT01174888-0 | Midostaurin | PKC/PDGFR | Leukaemia |
| NCT01174888 | NCT01174888-0 | Bortezomib | PSMB | Leukaemia |
| NCT01174888 | NCT01174888-0 | Chemotherapy | chemotherapy | Leukaemia |
| NCT01183416 | NCT01183416-0 | Naxitamab | GD-2 | Brain and nervous system cancers |
| NCT01183416 | NCT01183416-0 | Gm-Csf | CSF2 | Brain and nervous system cancers |
| NCT01183416 | NCT01183416-0 | Retinoic Acid | RAR | Brain and nervous system cancers |
| NCT01183897 | NCT01183897-0 | Naxitamab | GD-2 | Brain and nervous system cancers |
| NCT01183897 | NCT01183897-0 | Gm-Csf | CSF2 | Brain and nervous system cancers |
| NCT01183897 | NCT01183897-0 | Retinoic Acid | RAR | Brain and nervous system cancers |
| NCT01202877 | NCT01202877-0 | Azacitidine | chemotherapy | Leukaemia |
| NCT01202877 | NCT01202877-0 | Midostaurin | PKC/PDGFR | Leukaemia |
| NCT01206140 | NCT01206140-0 | Temsirolimus | mTOR | Other solid tumors |
| NCT01206140 | NCT01206140-0 | Selumetinib | MEK | Other solid tumors |
| NCT01217450 | NCT01217450-0 | Selumetinib | MEK | Colon and rectum cancers |
| NCT01217450 | NCT01217450-0 | Cetuximab | EGFR | Colon and rectum cancers |
| NCT01219699 | NCT01219699-0 | Alpelisib | PI3K | Breast cancer |
| NCT01219699 | NCT01219699-0 | Fulvestrant | Endocrine Therapy | Breast cancer |
| NCT01222689 | NCT01222689-0 | Erlotinib | EGFR | Pancreas cancer |
| NCT01222689 | NCT01222689-0 | Selumetinib | MEK | Pancreas cancer |
| NCT01229150 | NCT01229150-0 | Selumetinib | MEK | lung cancers |
| NCT01229150 | NCT01229150-0 | Erlotinib | EGFR | lung cancers |
| NCT01242605 | NCT01242605-0 | Platinum-Based Chemotherapy | chemotherapy | Gallbladder and biliary tract cancer |
| NCT01242605 | NCT01242605-0 | Gemcitabine | chemotherapy | Gallbladder and biliary tract cancer |
| NCT01242605 | NCT01242605-0 | Selumetinib | MEK | Gallbladder and biliary tract cancer |
| NCT01248247 | NCT01248247-0 | Selumetinib | MEK | lung cancers |
| NCT01248247 | NCT01248247-0 | Mk2206 | AKT | lung cancers |
| NCT01256359 | NCT01256359-0 | Docetaxel | chemotherapy | Melanoma |
| NCT01256359 | NCT01256359-0 | Selumetinib | MEK | Melanoma |
| NCT01282502 | NCT01282502-0 | Chemotherapy | chemotherapy | Colon and rectum cancers |
| NCT01282502 | NCT01282502-0 | Midostaurin | PKC/PDGFR | Colon and rectum cancers |
| NCT01287130 | NCT01287130-0 | Selumetinib | MEK | Colon and rectum cancers |
| NCT01287130 | NCT01287130-0 | Cetuximab | EGFR | Colon and rectum cancers |
| NCT01290549 | NCT01290549-0 | Polatuzumab Vedotin | CD79B/Tubulin | Lymphomas |
| NCT01290549 | NCT01290549-0 | Rituximab | CD20 | Lymphomas |
| NCT01294735 | NCT01294735-0 | Niraparib | PARP | Other solid tumors |
| NCT01294735 | NCT01294735-0 | Temozolomide | chemotherapy | Other solid tumors |
| NCT01300962 | NCT01300962-1 | Byl719 | PI3K | Breast cancer |
| NCT01300962 | NCT01300962-1 | Capecitabine | chemotherapy | Breast cancer |
| NCT01300962 | NCT01300962-2 | Bkm120 | PI3K | Breast cancer |
| NCT01300962 | NCT01300962-2 | Capecitabine | chemotherapy | Breast cancer |
| NCT01300962 | NCT01300962-3 | Bkm120 | PI3K | Breast cancer |
| NCT01300962 | NCT01300962-3 | Capecitabine | chemotherapy | Breast cancer |
| NCT01300962 | NCT01300962-3 | Trastuzumab | HER2 | Breast cancer |
| NCT01300962 | NCT01300962-4 | Bkm120 | PI3K | Breast cancer |
| NCT01300962 | NCT01300962-4 | Capecitabine | chemotherapy | Breast cancer |
| NCT01300962 | NCT01300962-4 | Lapatinib | EGFR | Breast cancer |
| NCT01306630 | NCT01306630-0 | Tivozanib | VEGFR/PDGFR | Other solid tumors |
| NCT01306630 | NCT01306630-0 | Capecitabine | chemotherapy | Other solid tumors |
| NCT01333475 | NCT01333475-0 | Mk2206 | AKT | Colon and rectum cancers |
| NCT01333475 | NCT01333475-0 | Selumetinib | MEK | Colon and rectum cancers |
| NCT01337765 | NCT01337765-0 | Dactolisib | PI3K | Other solid tumors |
| NCT01337765 | NCT01337765-0 | Binimetinib | MEK | Other solid tumors |
| NCT01352273 | NCT01352273-0 | Binimetinib | MEK | Other solid tumors |
| NCT01352273 | NCT01352273-0 | Raf265 | RAF | Other solid tumors |
| NCT01363232 | NCT01363232-0 | Buparlisib | PI3K | Other solid tumors |
| NCT01363232 | NCT01363232-0 | Binimetinib | MEK | Other solid tumors |
| NCT01364051 | NCT01364051-0 | Cediranib | KDR | Melanoma |
| NCT01364051 | NCT01364051-0 | Selumetinib | MEK | Melanoma |
| NCT01371630 | NCT01371630-0 | Inotuzumab Ozogamicin | CD22/DNA | Leukaemia |
| NCT01371630 | NCT01371630-0 | Chemotherapy | chemotherapy | Leukaemia |
| NCT01392521 | NCT01392521-0 | Copanlisib | PI3K | Others |
| NCT01392521 | NCT01392521-0 | Refametinib | MEK | Others |
| NCT01394016 | NCT01394016-0 | Abemaciclib | CDK4/CDK6 | Others |
| NCT01394016 | NCT01394016-0 | Fulvestrant | Endocrine Therapy | Others |
| NCT01411410 | NCT01411410-0 | Paclitaxel | chemotherapy | Others |
| NCT01411410 | NCT01411410-0 | Copanlisib | PI3K | Others |
| NCT01423123 | NCT01423123-0 | Paclitaxel | chemotherapy | Breast cancer |
| NCT01423123 | NCT01423123-0 | Trastuzumab | HER2 | Breast cancer |
| NCT01423123 | NCT01423123-0 | Neratinib | EGFR | Breast cancer |
| NCT01449058 | NCT01449058-0 | Alpelisib | PI3K | Others |
| NCT01449058 | NCT01449058-0 | Binimetinib | MEK | Others |
| NCT01460537 | NCT01460537-0 | Copanlisib | PI3K | Others |
| NCT01460537 | NCT01460537-0 | Gemcitabine | chemotherapy | Others |
| NCT01477606 | NCT01477606-0 | Midostaurin | PKC/PDGFR | Leukaemia |
| NCT01477606 | NCT01477606-0 | Cytarabine | chemotherapy | Leukaemia |
| NCT01477606 | NCT01477606-0 | Daunorubicin | chemotherapy | Leukaemia |
| NCT01478594 | NCT01478594-0 | Tivozanib | VEGFR/PDGFR | Colon and rectum cancers |
| NCT01478594 | NCT01478594-0 | Mfolfox6 | chemotherapy | Colon and rectum cancers |
| NCT01494662 | NCT01494662-0 | Neratinib | EGFR | Breast cancer |
| NCT01494662 | NCT01494662-0 | Capecitabine | chemotherapy | Breast cancer |
| NCT01525602 | NCT01525602-0 | Pexidartinib | CSF1R/PDGFR | Other solid tumors |
| NCT01525602 | NCT01525602-0 | Paclitaxel | chemotherapy | Other solid tumors |
| NCT01535989 | NCT01535989-0 | Inotuzumab Ozogamicin | CD22/DNA | Lymphomas |
| NCT01535989 | NCT01535989-0 | Temsirolimus | mTOR | Lymphomas |
| NCT01543698 | NCT01543698-0 | Binimetinib | MEK | Other solid tumors |
| NCT01543698 | NCT01543698-0 | Encorafenib | RAF | Other solid tumors |
| NCT01546038 | NCT01546038-1 | Glasdegib | SMO | Leukaemia |
| NCT01546038 | NCT01546038-1 | Cytarabine | chemotherapy | Leukaemia |
| NCT01546038 | NCT01546038-2 | Glasdegib | SMO | Leukaemia |
| NCT01546038 | NCT01546038-2 | Decitabine | chemotherapy | Leukaemia |
| NCT01546038 | NCT01546038-3 | Glasdegib | SMO | Leukaemia |
| NCT01546038 | NCT01546038-3 | Daunorubicin | chemotherapy | Leukaemia |
| NCT01546038 | NCT01546038-3 | Cytarabine | chemotherapy | Leukaemia |
| NCT01562990 | NCT01562990-0 | Inotuzumab Ozogamicin | CD22/DNA | Lymphomas |
| NCT01562990 | NCT01562990-0 | Rituximab | CD20 | Lymphomas |
| NCT01586624 | NCT01586624-0 | Vandetanib | VEGFR/EGFR | lung cancers |
| NCT01586624 | NCT01586624-0 | Selumetinib | MEK | lung cancers |
| NCT01596751 | NCT01596751-0 | Pexidartinib | CSF1R/PDGFR | Breast cancer |
| NCT01596751 | NCT01596751-0 | Eribulin | chemotherapy | Breast cancer |
| NCT01605916 | NCT01605916-0 | Selumetinib | MEK | lung cancers |
| NCT01605916 | NCT01605916-0 | Docetaxel | chemotherapy | lung cancers |
| NCT01610336 | NCT01610336-0 | Capmatinib | MET | lung cancers |
| NCT01610336 | NCT01610336-0 | Gefitinib | EGFR | lung cancers |
| NCT01613950 | NCT01613950-0 | Alpelisib | PI3K | Stomach cancer |
| NCT01613950 | NCT01613950-0 | Luminespib | HSP90 | Stomach cancer |
| NCT01623349 | NCT01623349-0 | Alpelisib | PI3K | Other solid tumors |
| NCT01623349 | NCT01623349-0 | Olaparib | PARP | Other solid tumors |
| NCT01649336 | NCT01649336-0 | Binimetinib | MEK | Ovary cancer |
| NCT01649336 | NCT01649336-0 | Paclitaxel | chemotherapy | Ovary cancer |
| NCT01655225 | NCT01655225-0 | Samotolisib | PI3K | Others |
| NCT01655225 | NCT01655225-0 | Abemaciclib | CDK4/CDK6 | Others |
| NCT01655225 | NCT01655225-0 | Letrozole | Endocrine Therapy | Others |
| NCT01658943 | NCT01658943-0 | Mk2206 | AKT | Pancreas cancer |
| NCT01658943 | NCT01658943-0 | Selumetinib | MEK | Pancreas cancer |
| NCT01662804 | NCT01662804-0 | Naxitamab | GD-2 | Brain and nervous system cancers |
| NCT01662804 | NCT01662804-0 | Il12 | IL12 | Brain and nervous system cancers |
| NCT01664910 | NCT01664910-0 | Inotuzumab Ozogamicin | CD22/DNA | Other hematologic Neoplasms |
| NCT01664910 | NCT01664910-0 | Fludarabine | chemotherapy | Other hematologic Neoplasms |
| NCT01664910 | NCT01664910-0 | Bendamustine | chemotherapy | Other hematologic Neoplasms |
| NCT01664910 | NCT01664910-0 | Rituximab | CD20 | Other hematologic Neoplasms |
| NCT01670877 | NCT01670877-0 | Neratinib | EGFR | Breast cancer |
| NCT01670877 | NCT01670877-0 | Fulvestrant | Endocrine Therapy | Breast cancer |
| NCT01679119 | NCT01679119-0 | Inotuzumab Ozogamicin | CD22/DNA | Lymphomas |
| NCT01679119 | NCT01679119-0 | Rituximab | CD20 | Lymphomas |
| NCT01679119 | NCT01679119-0 | Cyclophosphamide | chemotherapy | Lymphomas |
| NCT01679119 | NCT01679119-0 | Vincristine | chemotherapy | Lymphomas |
| NCT01679119 | NCT01679119-0 | Prednisolone | Steroid Treatment | Lymphomas |
| NCT01691898 | NCT01691898-0 | Obinutuzumab | CD20 | Lymphomas |
| NCT01691898 | NCT01691898-0 | Polatuzumab Vedotin | CD79B/Tubulin | Lymphomas |
| NCT01719380 | NCT01719380-1 | Encorafenib | RAF | Colon and rectum cancers |
| NCT01719380 | NCT01719380-1 | Cetuximab | EGFR | Colon and rectum cancers |
| NCT01719380 | NCT01719380-2 | Lgx818 | RAF | Colon and rectum cancers |
| NCT01719380 | NCT01719380-2 | Cetuximab | EGFR | Colon and rectum cancers |
| NCT01719380 | NCT01719380-3 | Encorafenib | RAF | Colon and rectum cancers |
| NCT01719380 | NCT01719380-3 | Alpelisib | PI3K | Colon and rectum cancers |
| NCT01719380 | NCT01719380-3 | Cetuximab | EGFR | Colon and rectum cancers |
| NCT01719380 | NCT01719380-4 | Encorafenib | RAF | Colon and rectum cancers |
| NCT01719380 | NCT01719380-4 | Alpelisib | PI3K | Colon and rectum cancers |
| NCT01719380 | NCT01719380-4 | Cetuximab | EGFR | Colon and rectum cancers |
| NCT01735968 | NCT01735968-0 | Imatinib | BCR-ABL | Gallbladder and biliary tract cancer |
| NCT01735968 | NCT01735968-0 | Alpelisib | PI3K | Gallbladder and biliary tract cancer |
| NCT01737008 | NCT01737008-0 | Dacomitinib | EGFR | Head and Neck Neoplasms |
| NCT01737008 | NCT01737008-0 | Platinum-Based Chemotherapy | chemotherapy | Head and Neck Neoplasms |
| NCT01749969 | NCT01749969-0 | Lsatuximab | CD38 | Multiple Myeloma |
| NCT01749969 | NCT01749969-0 | Lenalidomide | CRBN | Multiple Myeloma |
| NCT01749969 | NCT01749969-0 | Dexamethasone | Steroid Treatment | Multiple Myeloma |
| NCT01750281 | NCT01750281-0 | Selumetinib | MEK | lung cancers |
| NCT01750281 | NCT01750281-0 | Docetaxel | chemotherapy | lung cancers |
| NCT01757626 | NCT01757626-0 | Naxitamab | GD-2 | Brain and nervous system cancers |
| NCT01757626 | NCT01757626-0 | Gm-Csf | CSF2 | Brain and nervous system cancers |
| NCT01781572 | NCT01781572-0 | Ribociclib | CDK4/CDK6 | Melanoma |
| NCT01781572 | NCT01781572-0 | Binimetinib | MEK | Melanoma |
| NCT01783197 | NCT01783197-1 | Paclitaxel | chemotherapy | lung cancers |
| NCT01783197 | NCT01783197-1 | Platinum-Based Chemotherapy | chemotherapy | lung cancers |
| NCT01783197 | NCT01783197-1 | Selumetinib | MEK | lung cancers |
| NCT01783197 | NCT01783197-2 | Pemetrexed | chemotherapy | lung cancers |
| NCT01783197 | NCT01783197-2 | Platinum-Based Chemotherapy | chemotherapy | lung cancers |
| NCT01783197 | NCT01783197-2 | Selumetinib | MEK | lung cancers |
| NCT01783197 | NCT01783197-3 | Pemetrexed | chemotherapy | lung cancers |
| NCT01783197 | NCT01783197-3 | Selumetinib | MEK | lung cancers |
| NCT01790126 | NCT01790126-0 | Lhrha | Endocrine Therapy | Prostate cancer |
| NCT01790126 | NCT01790126-0 | Apalutamide | Endocrine Therapy | Prostate cancer |
| NCT01790503 | NCT01790503-0 | Pexidartinib | CSF1R/PDGFR | Brain and nervous system cancers |
| NCT01790503 | NCT01790503-0 | Temozolomide | chemotherapy | Brain and nervous system cancers |
| NCT01791478 | NCT01791478-0 | Alpelisib | PI3K | Breast cancer |
| NCT01791478 | NCT01791478-0 | Letrozole | Endocrine Therapy | Breast cancer |
| NCT01792687 | NCT01792687-0 | Apalutamide | Endocrine Therapy | Prostate cancer |
| NCT01792687 | NCT01792687-0 | Abiraterone | CYP17A1 | Prostate cancer |
| NCT01792687 | NCT01792687-0 | Prednisone | Steroid Treatment | Prostate cancer |
| NCT01808573 | NCT01808573-0 | Neratinib | EGFR | Breast cancer |
| NCT01808573 | NCT01808573-0 | Capecitabine | chemotherapy | Breast cancer |
| NCT01809210 | NCT01809210-0 | Selumetinib | MEK | lung cancers |
| NCT01809210 | NCT01809210-0 | Chemotherapy | chemotherapy | lung cancers |
| NCT01822613 | NCT01822613-0 | Elgemtumab | ERBB3 | Other solid tumors |
| NCT01822613 | NCT01822613-0 | Alpelisib | PI3K | Other solid tumors |
| NCT01827267 | NCT01827267-0 | Neratinib | EGFR | lung cancers |
| NCT01827267 | NCT01827267-0 | Temsirolimus | mTOR | lung cancers |
| NCT01828034 | NCT01828034-0 | Gemcitabine | chemotherapy | Gallbladder and biliary tract cancer |
| NCT01828034 | NCT01828034-0 | Platinum-Based Chemotherapy | chemotherapy | Gallbladder and biliary tract cancer |
| NCT01828034 | NCT01828034-0 | Binimetinib | MEK | Gallbladder and biliary tract cancer |
| NCT01830361 | NCT01830361-0 | Midostaurin | PKC/PDGFR | Leukaemia |
| NCT01830361 | NCT01830361-0 | Cytarabine | chemotherapy | Leukaemia |
| NCT01831089 | NCT01831089-0 | Lurbinectedin | chemotherapy | Other solid tumors |
| NCT01831089 | NCT01831089-0 | Paclitaxel | chemotherapy | Other solid tumors |
| NCT01831089 | NCT01831089-0 | Bevacizumab | VEGF | Other solid tumors |
| NCT01857193 | NCT01857193-1 | Ribociclib | CDK4/CDK6 | Breast cancer |
| NCT01857193 | NCT01857193-1 | Everolimus | mTOR | Breast cancer |
| NCT01857193 | NCT01857193-1 | Exemestane | Endocrine Therapy | Breast cancer |
| NCT01857193 | NCT01857193-2 | Ribociclib | CDK4/CDK6 | Breast cancer |
| NCT01857193 | NCT01857193-2 | Exemestane | Endocrine Therapy | Breast cancer |
| NCT01859026 | NCT01859026-0 | Binimetinib | MEK | lung cancers |
| NCT01859026 | NCT01859026-0 | Erlotinib | EGFR | lung cancers |
| NCT01870505 | NCT01870505-1 | Alpelisib | PI3K | Breast cancer |
| NCT01870505 | NCT01870505-1 | Letrozole | Endocrine Therapy | Breast cancer |
| NCT01870505 | NCT01870505-2 | Alpelisib | PI3K | Breast cancer |
| NCT01870505 | NCT01870505-2 | Exemestane | Endocrine Therapy | Breast cancer |
| NCT01871675 | NCT01871675-1 | Duvelisib | PI3K | Other hematologic Neoplasms |
| NCT01871675 | NCT01871675-1 | Rituximab | CD20 | Other hematologic Neoplasms |
| NCT01871675 | NCT01871675-2 | Duvelisib | PI3K | Other hematologic Neoplasms |
| NCT01871675 | NCT01871675-2 | Bendamustine | chemotherapy | Other hematologic Neoplasms |
| NCT01872260 | NCT01872260-0 | Ribociclib | CDK4/CDK6 | Breast cancer |
| NCT01872260 | NCT01872260-0 | Alpelisib | PI3K | Breast cancer |
| NCT01872260 | NCT01872260-0 | Letrozole | Endocrine Therapy | Breast cancer |
| NCT01885949 | NCT01885949-0 | Tivozanib | VEGFR/PDGFR | Prostate cancer |
| NCT01885949 | NCT01885949-0 | Enzalutamide | Endocrine Therapy | Prostate cancer |
| NCT01909453 | NCT01909453-0 | Encorafenib | RAF | Melanoma |
| NCT01909453 | NCT01909453-0 | Binimetinib | MEK | Melanoma |
| NCT01911507 | NCT01911507-0 | Capmatinib | MET | lung cancers |
| NCT01911507 | NCT01911507-0 | Erlotinib | EGFR | lung cancers |
| NCT01920061 | NCT01920061-0 | Gedatolisib | PI3K/mTOR | Others |
| NCT01920061 | NCT01920061-0 | Dacomitinib | EGFR | Others |
| NCT01921335 | NCT01921335-0 | Tucatinib | HER2 | Breast cancer |
| NCT01921335 | NCT01921335-0 | Trastuzumab | HER2 | Breast cancer |
| NCT01923168 | NCT01923168-0 | Alpelisib | PI3K | Breast cancer |
| NCT01923168 | NCT01923168-0 | Letrozole | Endocrine Therapy | Breast cancer |
| NCT01925131 | NCT01925131-0 | Chemotherapy | chemotherapy | Other hematologic Neoplasms |
| NCT01925131 | NCT01925131-0 | Inotuzumab Ozogamicin | CD22/DNA | Other hematologic Neoplasms |
| NCT01927341 | NCT01927341-0 | Binimetinib | MEK | Colon and rectum cancers |
| NCT01927341 | NCT01927341-0 | Panitumumab | EGFR | Colon and rectum cancers |
| NCT01928459 | NCT01928459-0 | Infigratinib | FGFR | Other solid tumors |
| NCT01928459 | NCT01928459-0 | Alpelisib | PI3K | Other solid tumors |
| NCT01933932 | NCT01933932-0 | Selumetinib | MEK | lung cancers |
| NCT01933932 | NCT01933932-0 | Docetaxel | chemotherapy | lung cancers |
| NCT01938612 | NCT01938612-0 | Durvalumab | PD-L1 | Other solid tumors |
| NCT01938612 | NCT01938612-0 | Tremelimumab | CTLA-4 | Other solid tumors |
| NCT01951157 | NCT01951157-0 | Gemcitabine | chemotherapy | lung cancers |
| NCT01951157 | NCT01951157-0 | Lurbinectedin | chemotherapy | lung cancers |
| NCT01953926 | NCT01953926-1 | Neratinib | EGFR | Other solid tumors |
| NCT01953926 | NCT01953926-1 | Trastuzumab | HER2 | Other solid tumors |
| NCT01953926 | NCT01953926-2 | Neratinib | EGFR | Other solid tumors |
| NCT01953926 | NCT01953926-2 | Fulvestrant | Endocrine Therapy | Other solid tumors |
| NCT01953926 | NCT01953926-2 | Trastuzumab | HER2 | Other solid tumors |
| NCT01958021 | NCT01958021-0 | Ribociclib | CDK4/CDK6 | Breast cancer |
| NCT01958021 | NCT01958021-0 | Letrozole | Endocrine Therapy | Breast cancer |
| NCT01970540 | NCT01970540-0 | Doxorubicin | chemotherapy | Other solid tumors |
| NCT01970540 | NCT01970540-0 | Lurbinectedin | chemotherapy | Other solid tumors |
| NCT01970553 | NCT01970553-0 | Gemcitabine | chemotherapy | Other solid tumors |
| NCT01970553 | NCT01970553-0 | Lurbinectedin | chemotherapy | Other solid tumors |
| NCT01974752 | NCT01974752-0 | Selumetinib | MEK | Melanoma |
| NCT01974752 | NCT01974752-0 | Dacarbazine | chemotherapy | Melanoma |
| NCT01975831 | NCT01975831-0 | Durvalumab | PD-L1 | Other solid tumors |
| NCT01975831 | NCT01975831-0 | Tremelimumab | CTLA-4 | Other solid tumors |
| NCT01980667 | NCT01980667-0 | Lurbinectedin | chemotherapy | Other solid tumors |
| NCT01980667 | NCT01980667-0 | Platinum-Based Chemotherapy | chemotherapy | Other solid tumors |
| NCT01982955 | NCT01982955-1 | Tepotinib | MET | lung cancers |
| NCT01982955 | NCT01982955-1 | Gefitinib | EGFR | lung cancers |
| NCT01982955 | NCT01982955-2 | Pemetrexed | chemotherapy | lung cancers |
| NCT01982955 | NCT01982955-2 | Platinum-Based Chemotherapy | chemotherapy | lung cancers |
| NCT01983501 | NCT01983501-0 | Tucatinib | HER2 | Breast cancer |
| NCT01983501 | NCT01983501-0 | Trastuzumab Emtansine | HER2/Tubulin | Breast cancer |
| NCT01991379 | NCT01991379-0 | Binimetinib | MEK | Other solid tumors |
| NCT01991379 | NCT01991379-0 | Imatinib | BCR-ABL | Other solid tumors |
| NCT01992653 | NCT01992653-1 | Polatuzumab Vedotin | CD79B/Tubulin | Lymphomas |
| NCT01992653 | NCT01992653-1 | Obinutuzumab | CD20 | Lymphomas |
| NCT01992653 | NCT01992653-1 | Cyclophosphamide | chemotherapy | Lymphomas |
| NCT01992653 | NCT01992653-1 | Doxorubicin | chemotherapy | Lymphomas |
| NCT01992653 | NCT01992653-1 | Prednisone | Steroid Treatment | Lymphomas |
| NCT01992653 | NCT01992653-2 | Polatuzumab Vedotin | CD79B/Tubulin | Lymphomas |
| NCT01992653 | NCT01992653-2 | Rituximab | CD20 | Lymphomas |
| NCT01992653 | NCT01992653-2 | Cyclophosphamide | chemotherapy | Lymphomas |
| NCT01992653 | NCT01992653-2 | Doxorubicin | chemotherapy | Lymphomas |
| NCT01992653 | NCT01992653-2 | Prednisone | Steroid Treatment | Lymphomas |
| NCT02000947 | NCT02000947-0 | Durvalumab | PD-L1 | lung cancers |
| NCT02000947 | NCT02000947-0 | Tremelimumab | CTLA-4 | lung cancers |
| NCT02006485 | NCT02006485-1 | Ublituximab | CD20 | Other hematologic Neoplasms |
| NCT02006485 | NCT02006485-1 | Umbralisib | PI3K | Other hematologic Neoplasms |
| NCT02006485 | NCT02006485-2 | Ublituximab | CD20 | Other hematologic Neoplasms |
| NCT02006485 | NCT02006485-2 | Umbralisib | PI3K | Other hematologic Neoplasms |
| NCT02006485 | NCT02006485-2 | Ibrutinib | BTK | Other hematologic Neoplasms |
| NCT02006485 | NCT02006485-3 | Ublituximab | CD20 | Other hematologic Neoplasms |
| NCT02006485 | NCT02006485-3 | Umbralisib | PI3K | Other hematologic Neoplasms |
| NCT02006485 | NCT02006485-3 | Bendamustine | chemotherapy | Other hematologic Neoplasms |
| NCT02025114 | NCT02025114-0 | Selumetinib | MEK | lung cancers |
| NCT02025114 | NCT02025114-0 | Gefitinib | EGFR | lung cancers |
| NCT02025192 | NCT02025192-1 | Tucatinib | HER2 | Breast cancer |
| NCT02025192 | NCT02025192-1 | Capecitabine | chemotherapy | Breast cancer |
| NCT02025192 | NCT02025192-2 | Tucatinib | HER2 | Breast cancer |
| NCT02025192 | NCT02025192-2 | Trastuzumab | HER2 | Breast cancer |
| NCT02025192 | NCT02025192-3 | Tucatinib | HER2 | Breast cancer |
| NCT02025192 | NCT02025192-3 | Capecitabine | chemotherapy | Breast cancer |
| NCT02025192 | NCT02025192-3 | Trastuzumab | HER2 | Breast cancer |
| NCT02027961 | NCT02027961-1 | Durvalumab | PD-L1 | Melanoma |
| NCT02027961 | NCT02027961-1 | Dabrafenib | RAF | Melanoma |
| NCT02027961 | NCT02027961-1 | Trametinib | MEK | Melanoma |
| NCT02027961 | NCT02027961-2 | Durvalumab | PD-L1 | Melanoma |
| NCT02027961 | NCT02027961-2 | Trametinib | MEK | Melanoma |
| NCT02035813 | NCT02035813-0 | Ribociclib | CDK4/CDK6 | Breast cancer |
| NCT02035813 | NCT02035813-0 | Endocrine Therapy | Endocrine Therapy | Breast cancer |
| NCT02038010 | NCT02038010-0 | Alpelisib | PI3K | Breast cancer |
| NCT02038010 | NCT02038010-0 | Trastuzumab Emtansine | HER2/Tubulin | Breast cancer |
| NCT02038777 | NCT02038777-1 | Glasdegib | SMO | Leukaemia |
| NCT02038777 | NCT02038777-1 | Cytarabine | chemotherapy | Leukaemia |
| NCT02038777 | NCT02038777-2 | Glasdegib | SMO | Leukaemia |
| NCT02038777 | NCT02038777-2 | Daunorubicin | chemotherapy | Leukaemia |
| NCT02038777 | NCT02038777-2 | Cytarabine | chemotherapy | Leukaemia |
| NCT02038777 | NCT02038777-3 | Glasdegib | SMO | Leukaemia |
| NCT02038777 | NCT02038777-3 | Azacitidine | chemotherapy | Leukaemia |
| NCT02039336 | NCT02039336-0 | Dacomitinib | EGFR | Colon and rectum cancers |
| NCT02039336 | NCT02039336-0 | Mirdametinib | MEK | Colon and rectum cancers |
| NCT02041481 | NCT02041481-0 | Binimetinib | MEK | Colon and rectum cancers |
| NCT02041481 | NCT02041481-0 | Folfox | chemotherapy | Colon and rectum cancers |
| NCT02044120 | NCT02044120-1 | Niraparib | PARP | Other solid tumors |
| NCT02044120 | NCT02044120-1 | Temozolomide | chemotherapy | Other solid tumors |
| NCT02044120 | NCT02044120-2 | Niraparib | PARP | Other solid tumors |
| NCT02044120 | NCT02044120-2 | Irinotecan | chemotherapy | Other solid tumors |
| NCT02044120 | NCT02044120-3 | Niraparib | PARP | Other solid tumors |
| NCT02044120 | NCT02044120-3 | Irinotecan | chemotherapy | Other solid tumors |
| NCT02044120 | NCT02044120-3 | Temozolomide | chemotherapy | Other solid tumors |
| NCT02049515 | NCT02049515-0 | Duvelisib | PI3K | Leukaemia |
| NCT02049515 | NCT02049515-0 | Ofatumumab | CD20 | Leukaemia |
| NCT02049593 | NCT02049593-1 | Talazoparib | PARP | Other solid tumors |
| NCT02049593 | NCT02049593-1 | Temozolomide | chemotherapy | Other solid tumors |
| NCT02049593 | NCT02049593-2 | Talazoparib | PARP | Other solid tumors |
| NCT02049593 | NCT02049593-2 | Irinotecan | chemotherapy | Other solid tumors |
| NCT02051751 | NCT02051751-0 | Alpelisib | PI3K | Other solid tumors |
| NCT02051751 | NCT02051751-0 | Paclitaxel | chemotherapy | Other solid tumors |
| NCT02057133 | NCT02057133-1 | Abemaciclib | CDK4/CDK6 | Breast cancer |
| NCT02057133 | NCT02057133-1 | Letrozole | Endocrine Therapy | Breast cancer |
| NCT02057133 | NCT02057133-2 | Abemaciclib | CDK4/CDK6 | Breast cancer |
| NCT02057133 | NCT02057133-2 | Anastrozole | Endocrine Therapy | Breast cancer |
| NCT02057133 | NCT02057133-3 | Abemaciclib | CDK4/CDK6 | Breast cancer |
| NCT02057133 | NCT02057133-3 | Tamoxifen | Endocrine Therapy | Breast cancer |
| NCT02057133 | NCT02057133-4 | Abemaciclib | CDK4/CDK6 | Breast cancer |
| NCT02057133 | NCT02057133-4 | Exemestane | Endocrine Therapy | Breast cancer |
| NCT02057133 | NCT02057133-5 | Abemaciclib | CDK4/CDK6 | Breast cancer |
| NCT02057133 | NCT02057133-5 | Exemestane | Endocrine Therapy | Breast cancer |
| NCT02057133 | NCT02057133-5 | Everolimus | mTOR | Breast cancer |
| NCT02057133 | NCT02057133-6 | Abemaciclib | CDK4/CDK6 | Breast cancer |
| NCT02057133 | NCT02057133-6 | Trastuzumab | HER2 | Breast cancer |
| NCT02057133 | NCT02057133-7 | Samotolisib | PI3K | Breast cancer |
| NCT02057133 | NCT02057133-7 | Abemaciclib | CDK4/CDK6 | Breast cancer |
| NCT02057133 | NCT02057133-7 | Fulvestrant | Endocrine Therapy | Breast cancer |
| NCT02057133 | NCT02057133-8 | Abemaciclib | CDK4/CDK6 | Breast cancer |
| NCT02057133 | NCT02057133-8 | Trastuzumab | HER2 | Breast cancer |
| NCT02057133 | NCT02057133-8 | Pertuzumab | HER2 | Breast cancer |
| NCT02057133 | NCT02057133-8 | Loperamide | Other supportive therapy | Breast cancer |
| NCT02057133 | NCT02057133-9 | Abemaciclib | CDK4/CDK6 | Breast cancer |
| NCT02057133 | NCT02057133-9 | Endocrine Therapy | Endocrine Therapy | Breast cancer |
| NCT02058381 | NCT02058381-0 | Alpelisib | PI3K | Breast cancer |
| NCT02058381 | NCT02058381-0 | Tamoxifen | Endocrine Therapy | Breast cancer |
| NCT02058381 | NCT02058381-0 | Goserelin | Endocrine Therapy | Breast cancer |
| NCT02077933 | NCT02077933-1 | Alpelisib | PI3K | Other solid tumors |
| NCT02077933 | NCT02077933-1 | Everolimus | mTOR | Other solid tumors |
| NCT02077933 | NCT02077933-2 | Alpelisib | PI3K | Other solid tumors |
| NCT02077933 | NCT02077933-2 | Everolimus | mTOR | Other solid tumors |
| NCT02077933 | NCT02077933-2 | Exemestane | Endocrine Therapy | Other solid tumors |
| NCT02077933 | NCT02077933-3 | Alpelisib | PI3K | Other solid tumors |
| NCT02077933 | NCT02077933-3 | Exemestane | Endocrine Therapy | Other solid tumors |
| NCT02078609 | NCT02078609-0 | Lgh447 | PIM | Leukaemia |
| NCT02078609 | NCT02078609-0 | Midostaurin | PKC/PDGFR | Leukaemia |
| NCT02079636 | NCT02079636-1 | Abemaciclib | CDK4/CDK6 | lung cancers |
| NCT02079636 | NCT02079636-1 | Pemetrexed | chemotherapy | lung cancers |
| NCT02079636 | NCT02079636-2 | Abemaciclib | CDK4/CDK6 | lung cancers |
| NCT02079636 | NCT02079636-2 | Gemcitabine | chemotherapy | lung cancers |
| NCT02079636 | NCT02079636-3 | Abemaciclib | CDK4/CDK6 | lung cancers |
| NCT02079636 | NCT02079636-3 | Ramucirumab | KDR | lung cancers |
| NCT02079636 | NCT02079636-4 | Abemaciclib | CDK4/CDK6 | lung cancers |
| NCT02079636 | NCT02079636-4 | Samotolisib | PI3K | lung cancers |
| NCT02079636 | NCT02079636-5 | Abemaciclib | CDK4/CDK6 | lung cancers |
| NCT02079636 | NCT02079636-5 | Pembrolizumab | PD-1 | lung cancers |
| NCT02081378 | NCT02081378-1 | Asciminib | BCR-ABL | Leukaemia |
| NCT02081378 | NCT02081378-1 | Nilotinib | BCR-ABL | Leukaemia |
| NCT02081378 | NCT02081378-2 | Asciminib | BCR-ABL | Leukaemia |
| NCT02081378 | NCT02081378-2 | Imatinib | BCR-ABL | Leukaemia |
| NCT02081378 | NCT02081378-3 | Asciminib | BCR-ABL | Leukaemia |
| NCT02081378 | NCT02081378-3 | Dasatinib | BCR-ABL | Leukaemia |
| NCT02088112 | NCT02088112-0 | Durvalumab | PD-L1 | lung cancers |
| NCT02088112 | NCT02088112-0 | Gefitinib | EGFR | lung cancers |
| NCT02088684 | NCT02088684-1 | Ribociclib | CDK4/CDK6 | Breast cancer |
| NCT02088684 | NCT02088684-1 | Fulvestrant | Endocrine Therapy | Breast cancer |
| NCT02088684 | NCT02088684-1 | Alpelisib | PI3K | Breast cancer |
| NCT02088684 | NCT02088684-2 | Ribociclib | CDK4/CDK6 | Breast cancer |
| NCT02088684 | NCT02088684-2 | Fulvestrant | Endocrine Therapy | Breast cancer |
| NCT02088684 | NCT02088684-2 | Buparlisib | PI3K | Breast cancer |
| NCT02093403 | NCT02093403-0 | Decitabine | chemotherapy | Leukaemia |
| NCT02093403 | NCT02093403-0 | Selinexor | XPO1 | Leukaemia |
| NCT02100852 | NCT02100852-0 | Umbralisib | PI3K | Leukaemia |
| NCT02100852 | NCT02100852-0 | Obinutuzumab | CD20 | Leukaemia |
| NCT02100852 | NCT02100852-0 | Chlorambucil | chemotherapy | Leukaemia |
| NCT02106507 | NCT02106507-0 | Apalutamide | Endocrine Therapy | Prostate cancer |
| NCT02106507 | NCT02106507-0 | Everolimus | mTOR | Prostate cancer |
| NCT02107703 | NCT02107703-0 | Abemaciclib | CDK4/CDK6 | Breast cancer |
| NCT02107703 | NCT02107703-0 | Fulvestrant | Endocrine Therapy | Breast cancer |
| NCT02115295 | NCT02115295-0 | Cladribine | chemotherapy | Leukaemia |
| NCT02115295 | NCT02115295-0 | Idarubicin | Chemotherapy | Leukaemia |
| NCT02115295 | NCT02115295-0 | Cytarabine | chemotherapy | Leukaemia |
| NCT02116777 | NCT02116777-0 | Talazoparib | PARP | Other solid tumors |
| NCT02116777 | NCT02116777-0 | Temozolomide | chemotherapy | Other solid tumors |
| NCT02117219 | NCT02117219-1 | Durvalumab | PD-L1 | Lymphomas |
| NCT02117219 | NCT02117219-1 | Tremelimumab | CTLA-4 | Lymphomas |
| NCT02117219 | NCT02117219-2 | Durvalumab | PD-L1 | Lymphomas |
| NCT02117219 | NCT02117219-2 | Tremelimumab | CTLA-4 | Lymphomas |
| NCT02117219 | NCT02117219-2 | Azacitidine | chemotherapy | Lymphomas |
| NCT02117219 | NCT02117219-3 | Durvalumab | PD-L1 | Lymphomas |
| NCT02117219 | NCT02117219-3 | Azacitidine | chemotherapy | Lymphomas |
| NCT02118337 | NCT02118337-0 | Medi0680 | PD-1 | Kidney cancer |
| NCT02118337 | NCT02118337-0 | Durvalumab | PD-L1 | Kidney cancer |
| NCT02123758 | NCT02123758-0 | Abiraterone | CYP17A1 | Prostate cancer |
| NCT02123758 | NCT02123758-0 | Prednisone | Steroid Treatment | Prostate cancer |
| NCT02123758 | NCT02123758-0 | Apalutamide | Endocrine Therapy | Prostate cancer |
| NCT02137356 | NCT02137356-0 | Selinexor | XPO1 | Colon and rectum cancers |
| NCT02137356 | NCT02137356-0 | Capecitabine | chemotherapy | Colon and rectum cancers |
| NCT02141347 | NCT02141347-0 | Tremelimumab | CTLA-4 | Other solid tumors |
| NCT02141347 | NCT02141347-0 | Durvalumab | PD-L1 | Other solid tumors |
| NCT02143466 | NCT02143466-1 | Osimertinib | EGFR | lung cancers |
| NCT02143466 | NCT02143466-1 | Azd6094 | MET | lung cancers |
| NCT02143466 | NCT02143466-2 | Osimertinib | EGFR | lung cancers |
| NCT02143466 | NCT02143466-2 | Selumetinib | MEK | lung cancers |
| NCT02143466 | NCT02143466-3 | Osimertinib | EGFR | lung cancers |
| NCT02143466 | NCT02143466-3 | Durvalumab | PD-L1 | lung cancers |
| NCT02143466 | NCT02143466-4 | Encorafenib | RAF | lung cancers |
| NCT02143466 | NCT02143466-4 | Binimetinib | MEK | lung cancers |
| NCT02143466 | NCT02143466-5 | Encorafenib | RAF | lung cancers |
| NCT02143466 | NCT02143466-5 | Binimetinib | MEK | lung cancers |
| NCT02143466 | NCT02143466-5 | Ribociclib | CDK4/CDK6 | lung cancers |
| NCT02143466 | NCT02143466-6 | Encorafenib | RAF | lung cancers |
| NCT02143466 | NCT02143466-6 | Binimetinib | MEK | lung cancers |
| NCT02143466 | NCT02143466-6 | Infigratinib | FGFR | lung cancers |
| NCT02143466 | NCT02143466-7 | Encorafenib | RAF | lung cancers |
| NCT02143466 | NCT02143466-7 | Binimetinib | MEK | lung cancers |
| NCT02143466 | NCT02143466-7 | Buparlisib | PI3K | lung cancers |
| NCT02143466 | NCT02143466-8 | Encorafenib | RAF | lung cancers |
| NCT02143466 | NCT02143466-8 | Binimetinib | MEK | lung cancers |
| NCT02143466 | NCT02143466-8 | Capmatinib | MET | lung cancers |
| NCT02144038 | NCT02144038-0 | Lgh447 | PIM | Multiple Myeloma |
| NCT02144038 | NCT02144038-0 | Alpelisib | PI3K | Multiple Myeloma |
| NCT02151084 | NCT02151084-1 | Selumetinib | MEK | Gallbladder and biliary tract cancer |
| NCT02151084 | NCT02151084-1 | Platinum-Based Chemotherapy | chemotherapy | Gallbladder and biliary tract cancer |
| NCT02151084 | NCT02151084-2 | Selumetinib | MEK | Gallbladder and biliary tract cancer |
| NCT02151084 | NCT02151084-2 | Gemcitabine | chemotherapy | Gallbladder and biliary tract cancer |
| NCT02154776 | NCT02154776-0 | Ribociclib | CDK4/CDK6 | Breast cancer |
| NCT02154776 | NCT02154776-0 | Letrozole | Endocrine Therapy | Breast cancer |
| NCT02154776 | NCT02154776-0 | Buparlisib | PI3K | Breast cancer |
| NCT02155088 | NCT02155088-0 | Alpelisib | PI3K | Pancreas cancer |
| NCT02155088 | NCT02155088-0 | Gemcitabine | chemotherapy | Pancreas cancer |
| NCT02155088 | NCT02155088-0 | Paclitaxel | chemotherapy | Pancreas cancer |
| NCT02157324 | NCT02157324-0 | Acalabrutinib | BTK | Leukaemia |
| NCT02157324 | NCT02157324-0 | Acp-319 | PI3K | Leukaemia |
| NCT02158091 | NCT02158091-0 | Duvelisib | PI3K | Leukaemia |
| NCT02158091 | NCT02158091-0 | Fludarabine | chemotherapy | Leukaemia |
| NCT02158091 | NCT02158091-0 | Cyclophosphamide | chemotherapy | Leukaemia |
| NCT02158091 | NCT02158091-0 | Rituximab | CD20 | Leukaemia |
| NCT02159066 | NCT02159066-1 | Encorafenib | RAF | Melanoma |
| NCT02159066 | NCT02159066-1 | Binimetinib | MEK | Melanoma |
| NCT02159066 | NCT02159066-2 | Encorafenib | RAF | Melanoma |
| NCT02159066 | NCT02159066-2 | Binimetinib | MEK | Melanoma |
| NCT02159066 | NCT02159066-2 | Ribociclib | CDK4/CDK6 | Melanoma |
| NCT02159066 | NCT02159066-3 | Encorafenib | RAF | Melanoma |
| NCT02159066 | NCT02159066-3 | Binimetinib | MEK | Melanoma |
| NCT02159066 | NCT02159066-3 | Infigratinib | FGFR | Melanoma |
| NCT02159066 | NCT02159066-4 | Encorafenib | RAF | Melanoma |
| NCT02159066 | NCT02159066-4 | Binimetinib | MEK | Melanoma |
| NCT02159066 | NCT02159066-4 | Buparlisib | PI3K | Melanoma |
| NCT02159066 | NCT02159066-5 | Encorafenib | RAF | Melanoma |
| NCT02159066 | NCT02159066-5 | Binimetinib | MEK | Melanoma |
| NCT02159066 | NCT02159066-5 | Capmatinib | MET | Melanoma |
| NCT02164006 | NCT02164006-0 | Umbralisib | PI3K | Lymphomas |
| NCT02164006 | NCT02164006-0 | Brentuximab Vedotin | CD30/Tubulin | Lymphomas |
| NCT02167854 | NCT02167854-0 | Elgemtumab | ERBB3 | Breast cancer |
| NCT02167854 | NCT02167854-0 | Alpelisib | PI3K | Breast cancer |
| NCT02167854 | NCT02167854-0 | Trastuzumab | HER2 | Breast cancer |
| NCT02178436 | NCT02178436-1 | Gemcitabine | chemotherapy | Pancreas cancer |
| NCT02178436 | NCT02178436-1 | Paclitaxel | chemotherapy | Pancreas cancer |
| NCT02178436 | NCT02178436-1 | Selinexor | XPO1 | Pancreas cancer |
| NCT02178436 | NCT02178436-2 | Gemcitabine | chemotherapy | Pancreas cancer |
| NCT02178436 | NCT02178436-2 | Selinexor | XPO1 | Pancreas cancer |
| NCT02179671 | NCT02179671-1 | Gefitinib | EGFR | lung cancers |
| NCT02179671 | NCT02179671-1 | Durvalumab | PD-L1 | lung cancers |
| NCT02179671 | NCT02179671-2 | Osimertinib | EGFR | lung cancers |
| NCT02179671 | NCT02179671-2 | Durvalumab | PD-L1 | lung cancers |
| NCT02179671 | NCT02179671-3 | Selumetinib | MEK | lung cancers |
| NCT02179671 | NCT02179671-3 | Docetaxel | chemotherapy | lung cancers |
| NCT02179671 | NCT02179671-3 | Durvalumab | PD-L1 | lung cancers |
| NCT02179671 | NCT02179671-4 | Tremelimumab | CTLA-4 | lung cancers |
| NCT02179671 | NCT02179671-4 | Durvalumab | PD-L1 | lung cancers |
| NCT02180711 | NCT02180711-1 | Acalabrutinib | BTK | Lymphomas |
| NCT02180711 | NCT02180711-1 | Rituximab | CD20 | Lymphomas |
| NCT02180711 | NCT02180711-2 | Acalabrutinib | BTK | Lymphomas |
| NCT02180711 | NCT02180711-2 | Rituximab | CD20 | Lymphomas |
| NCT02180711 | NCT02180711-2 | Lenalidomide | CRBN | Lymphomas |
| NCT02185690 | NCT02185690-0 | Binimetinib | MEK | lung cancers |
| NCT02185690 | NCT02185690-0 | Platinum-Based Chemotherapy | chemotherapy | lung cancers |
| NCT02186834 | NCT02186834-0 | Selinexor | XPO1 | Multiple Myeloma |
| NCT02186834 | NCT02186834-0 | Doxorubicin | chemotherapy | Multiple Myeloma |
| NCT02186834 | NCT02186834-0 | Dexamethasone | Steroid Treatment | Multiple Myeloma |
| NCT02188264 | NCT02188264-0 | Selumetinib | MEK | Colon and rectum cancers |
| NCT02188264 | NCT02188264-0 | Cyclosporine | Calcineurin Inhibitors | Colon and rectum cancers |
| NCT02199665 | NCT02199665-0 | Selinexor | XPO1 | Multiple Myeloma |
| NCT02199665 | NCT02199665-0 | Carfilzomib | PSMB | Multiple Myeloma |
| NCT02199665 | NCT02199665-0 | Dexamethasone | Steroid Treatment | Multiple Myeloma |
| NCT02210364 | NCT02210364-0 | Lurbinectedin | chemotherapy | Other solid tumors |
| NCT02210364 | NCT02210364-0 | Capecitabine | chemotherapy | Other solid tumors |
| NCT02211014 | NCT02211014-0 | Acalabrutinib | BTK | Multiple Myeloma |
| NCT02211014 | NCT02211014-0 | Dexamethasone | Steroid Treatment | Multiple Myeloma |
| NCT02212561 | NCT02212561-0 | Selinexor | XPO1 | Leukaemia |
| NCT02212561 | NCT02212561-0 | Fludarabine | chemotherapy | Leukaemia |
| NCT02212561 | NCT02212561-0 | Cytarabine | chemotherapy | Leukaemia |
| NCT02220842 | NCT02220842-1 | Atezolizumab | PD-L1 | Lymphomas |
| NCT02220842 | NCT02220842-1 | Obinutuzumab | CD20 | Lymphomas |
| NCT02220842 | NCT02220842-2 | Atezolizumab | PD-L1 | Lymphomas |
| NCT02220842 | NCT02220842-2 | Tazemetostat | EZH2 | Lymphomas |
| NCT02221960 | NCT02221960-0 | Medi6383 | OX40 | Other solid tumors |
| NCT02221960 | NCT02221960-0 | Durvalumab | PD-L1 | Other solid tumors |
| NCT02222922 | NCT02222922-0 | Cofetuzumab Pelidotin | PTK-7/Tubulin | Others |
| NCT02222922 | NCT02222922-0 | Avelumab | PD-L1 | Others |
| NCT02236000 | NCT02236000-0 | Neratinib | EGFR | Leukaemia |
| NCT02236000 | NCT02236000-0 | Trastuzumab Emtansine | HER2/Tubulin | Leukaemia |
| NCT02236013 | NCT02236013-0 | Idarubicin | chemotherapy | Leukaemia |
| NCT02236013 | NCT02236013-0 | Cytarabine | chemotherapy | Leukaemia |
| NCT02236013 | NCT02236013-0 | Daunorubicin | chemotherapy | Leukaemia |
| NCT02236013 | NCT02236013-0 | Gilteritinib | FLT3 | Leukaemia |
| NCT02246621 | NCT02246621-1 | Abemaciclib | CDK4/CDK6 | Breast cancer |
| NCT02246621 | NCT02246621-1 | Anastrozole | Endocrine Therapy | Breast cancer |
| NCT02246621 | NCT02246621-2 | Abemaciclib | CDK4/CDK6 | Breast cancer |
| NCT02246621 | NCT02246621-2 | Letrozole | Endocrine Therapy | Breast cancer |
| NCT02249091 | NCT02249091-0 | Selinexor | XPO1 | Leukaemia |
| NCT02249091 | NCT02249091-0 | Cytarabine | chemotherapy | Leukaemia |
| NCT02249091 | NCT02249091-0 | Idarubicin | Chemotherapy | Leukaemia |
| NCT02250326 | NCT02250326-0 | Paclitaxel | chemotherapy | lung cancers |
| NCT02250326 | NCT02250326-0 | Durvalumab | PD-L1 | lung cancers |
| NCT02257541 | NCT02257541-0 | Infigratinib | FGFR | Other solid tumors |
| NCT02257541 | NCT02257541-0 | Imatinib | BCR-ABL | Other solid tumors |
| NCT02257567 | NCT02257567-1 | Polatuzumab Vedotin | CD79B/Tubulin | Lymphomas |
| NCT02257567 | NCT02257567-1 | Bendamustine | chemotherapy | Lymphomas |
| NCT02257567 | NCT02257567-1 | Obinutuzumab | CD20 | Lymphomas |
| NCT02257567 | NCT02257567-2 | Polatuzumab Vedotin | CD79B/Tubulin | Lymphomas |
| NCT02257567 | NCT02257567-2 | Bendamustine | chemotherapy | Lymphomas |
| NCT02257567 | NCT02257567-2 | Rituximab | CD20 | Lymphomas |
| NCT02257736 | NCT02257736-0 | Abiraterone | CYP17A1 | Prostate cancer |
| NCT02257736 | NCT02257736-0 | Prednisone | Steroid Treatment | Prostate cancer |
| NCT02257736 | NCT02257736-0 | Apalutamide | Endocrine Therapy | Prostate cancer |
| NCT02261220 | NCT02261220-0 | Durvalumab | PD-L1 | Other solid tumors |
| NCT02261220 | NCT02261220-0 | Tremelimumab | CTLA-4 | Other solid tumors |
| NCT02262741 | NCT02262741-0 | Durvalumab | PD-L1 | Head and Neck Neoplasms |
| NCT02262741 | NCT02262741-0 | Tremelimumab | CTLA-4 | Head and Neck Neoplasms |
| NCT02264678 | NCT02264678-0 | Ceralasertib | ATR | Other solid tumors |
| NCT02264678 | NCT02264678-0 | Durvalumab | PD-L1 | Other solid tumors |
| NCT02268851 | NCT02268851-0 | Umbralisib | PI3K | Other hematologic Neoplasms |
| NCT02268851 | NCT02268851-0 | Ibrutinib | BTK | Other hematologic Neoplasms |
| NCT02269293 | NCT02269293-0 | Selinexor | XPO1 | Other solid tumors |
| NCT02269293 | NCT02269293-0 | Paclitaxel | chemotherapy | Other solid tumors |
| NCT02269293 | NCT02269293-0 | Platinum-Based Chemotherapy | chemotherapy | Other solid tumors |
| NCT02273219 | NCT02273219-0 | Sotrastaurin | PKC | Melanoma |
| NCT02273219 | NCT02273219-0 | Alpelisib | PI3K | Melanoma |
| NCT02278120 | NCT02278120-1 | Ribociclib | CDK4/CDK6 | Breast cancer |
| NCT02278120 | NCT02278120-1 | Goserelin | Endocrine Therapy | Breast cancer |
| NCT02278120 | NCT02278120-1 | Nsai | Endocrine Therapy | Breast cancer |
| NCT02278120 | NCT02278120-2 | Ribociclib | CDK4/CDK6 | Breast cancer |
| NCT02278120 | NCT02278120-2 | Goserelin | Endocrine Therapy | Breast cancer |
| NCT02278120 | NCT02278120-2 | Tamoxifen | Endocrine Therapy | Breast cancer |
| NCT02278133 | NCT02278133-0 | Wnt974 | Porcupine | Colon and rectum cancers |
| NCT02278133 | NCT02278133-0 | Encorafenib | RAF | Colon and rectum cancers |
| NCT02278133 | NCT02278133-0 | Cetuximab | EGFR | Colon and rectum cancers |
| NCT02281084 | NCT02281084-0 | Azacitidine | chemotherapy | Lymphomas |
| NCT02281084 | NCT02281084-0 | Durvalumab | PD-L1 | Lymphomas |
| NCT02282371 | NCT02282371-0 | Cetuximab | EGFR | Head and Neck Neoplasms |
| NCT02282371 | NCT02282371-0 | Alpelisib | PI3K | Head and Neck Neoplasms |
| NCT02283775 | NCT02283775-0 | Isatuximab | CD38 | Multiple Myeloma |
| NCT02283775 | NCT02283775-0 | Pomalidomide | CRBN | Multiple Myeloma |
| NCT02283775 | NCT02283775-0 | Dexamethasone | Steroid Treatment | Multiple Myeloma |
| NCT02291055 | NCT02291055-0 | Adxs11-001 | vaccine | Other solid tumors |
| NCT02291055 | NCT02291055-0 | Durvalumab | PD-L1 | Other solid tumors |
| NCT02292550 | NCT02292550-0 | Ribociclib | CDK4/CDK6 | lung cancers |
| NCT02292550 | NCT02292550-0 | Ceritinib | ALK | lung cancers |
| NCT02296918 | NCT02296918-0 | Acalabrutinib | BTK | Leukaemia |
| NCT02296918 | NCT02296918-0 | Obinutuzumab | CD20 | Leukaemia |
| NCT02299518 | NCT02299518-1 | Mitoxantrone | chemotherapy | Leukaemia |
| NCT02299518 | NCT02299518-1 | Etoposide | chemotherapy | Leukaemia |
| NCT02299518 | NCT02299518-1 | Cytarabine | chemotherapy | Leukaemia |
| NCT02299518 | NCT02299518-1 | Selinexor | XPO1 | Leukaemia |
| NCT02299518 | NCT02299518-2 | Etoposide | chemotherapy | Leukaemia |
| NCT02299518 | NCT02299518-2 | Selinexor | XPO1 | Leukaemia |
| NCT02301130 | NCT02301130-1 | Mogamulizumab | CCR4 | Other solid tumors |
| NCT02301130 | NCT02301130-1 | Durvalumab | PD-L1 | Other solid tumors |
| NCT02301130 | NCT02301130-2 | Mogamulizumab | CCR4 | Other solid tumors |
| NCT02301130 | NCT02301130-2 | Tremelimumab | CTLA-4 | Other solid tumors |
| NCT02303392 | NCT02303392-0 | Selinexor | XPO1 | Other hematologic Neoplasms |
| NCT02303392 | NCT02303392-0 | Ibrutinib | BTK | Other hematologic Neoplasms |
| NCT02310321 | NCT02310321-0 | Idarubicin | chemotherapy | Leukaemia |
| NCT02310321 | NCT02310321-0 | Cytarabine | chemotherapy | Leukaemia |
| NCT02310321 | NCT02310321-0 | Gilteritinib | FLT3 | Leukaemia |
| NCT02311361 | NCT02311361-0 | Durvalumab | PD-L1 | Pancreas cancer |
| NCT02311361 | NCT02311361-0 | Tremelimumab | CTLA-4 | Pancreas cancer |
| NCT02311998 | NCT02311998-0 | Bosutinib | BCR-ABL | Leukaemia |
| NCT02311998 | NCT02311998-0 | Inotuzumab Ozogamicin | CD22/DNA | Leukaemia |
| NCT02316197 | NCT02316197-0 | Nedisertib | DNA-PK | Others |
| NCT02316197 | NCT02316197-0 | Avelumab | PD-L1 | Others |
| NCT02317874 | NCT02317874-0 | Talazoparib | PARP | Other solid tumors |
| NCT02317874 | NCT02317874-0 | Paclitaxel | chemotherapy | Other solid tumors |
| NCT02317874 | NCT02317874-0 | Platinum-Based Chemotherapy | chemotherapy | Other solid tumors |
| NCT02318277 | NCT02318277-0 | Durvalumab | PD-L1 | Other solid tumors |
| NCT02318277 | NCT02318277-0 | Epacadostat | IDO1 | Other solid tumors |
| NCT02319044 | NCT02319044-0 | Durvalumab | PD-L1 | Head and Neck Neoplasms |
| NCT02319044 | NCT02319044-0 | Tremelimumab | CTLA-4 | Head and Neck Neoplasms |
| NCT02323126 | NCT02323126-0 | Nivolumab | PD-1 | lung cancers |
| NCT02323126 | NCT02323126-0 | Capmatinib | MET | lung cancers |
| NCT02328014 | NCT02328014-0 | Acalabrutinib | BTK | Lymphomas |
| NCT02328014 | NCT02328014-0 | Acp-319 | PI3K | Lymphomas |
| NCT02332850 | NCT02332850-0 | Dexamethasone | Steroid Treatment | Multiple Myeloma |
| NCT02332850 | NCT02332850-0 | Isatuximab | CD38 | Multiple Myeloma |
| NCT02332850 | NCT02332850-0 | Carfilzomib | PSMB | Multiple Myeloma |
| NCT02333370 | NCT02333370-1 | Ribociclib | CDK4/CDK6 | Breast cancer |
| NCT02333370 | NCT02333370-1 | Tamoxifen | Endocrine Therapy | Breast cancer |
| NCT02333370 | NCT02333370-2 | Ribociclib | CDK4/CDK6 | Breast cancer |
| NCT02333370 | NCT02333370-2 | Letrozole | Endocrine Therapy | Breast cancer |
| NCT02333370 | NCT02333370-3 | Ribociclib | CDK4/CDK6 | Breast cancer |
| NCT02333370 | NCT02333370-3 | Fulvestrant | Endocrine Therapy | Breast cancer |
| NCT02335944 | NCT02335944-0 | Capmatinib | MET | lung cancers |
| NCT02335944 | NCT02335944-0 | Nazartinib | EGFR | lung cancers |
| NCT02336165 | NCT02336165-0 | Durvalumab | PD-L1 | Brain and nervous system cancers |
| NCT02336165 | NCT02336165-0 | Bevacizumab | VEGF | Brain and nervous system cancers |
| NCT02336815 | NCT02336815-0 | Selinexor | XPO1 | Multiple Myeloma |
| NCT02336815 | NCT02336815-0 | Dexamethasone | Steroid Treatment | Multiple Myeloma |
| NCT02337530 | NCT02337530-0 | Selumetinib | MEK | lung cancers |
| NCT02337530 | NCT02337530-0 | Chemotherapy | chemotherapy | lung cancers |
| NCT02340975 | NCT02340975-0 | Durvalumab | PD-L1 | Stomach cancer |
| NCT02340975 | NCT02340975-0 | Tremelimumab | CTLA-4 | Stomach cancer |
| NCT02343042 | NCT02343042-0 | Selinexor | XPO1 | Multiple Myeloma |
| NCT02343042 | NCT02343042-0 | Dexamethasone | Steroid Treatment | Multiple Myeloma |
| NCT02343042 | NCT02343042-0 | Pomalidomide | CRBN | Multiple Myeloma |
| NCT02343042 | NCT02343042-0 | Elotuzumab | SLAMF7 | Multiple Myeloma |
| NCT02343172 | NCT02343172-0 | Siremadlin | p53/MDM2 | Other solid tumors |
| NCT02343172 | NCT02343172-0 | Ribociclib | CDK4/CDK6 | Other solid tumors |
| NCT02344472 | NCT02344472-0 | Endocrine Therapy | Endocrine Therapy | Breast cancer |
| NCT02344472 | NCT02344472-0 | Trastuzumab | HER2 | Breast cancer |
| NCT02344472 | NCT02344472-0 | Pertuzumab | HER2 | Breast cancer |
| NCT02344472 | NCT02344472-0 | Ribociclib | CDK4/CDK6 | Breast cancer |
| NCT02351739 | NCT02351739-0 | Acalabrutinib | BTK | Bladder cancer |
| NCT02351739 | NCT02351739-0 | Pembrolizumab | PD-1 | Bladder cancer |
| NCT02352948 | NCT02352948-0 | Durvalumab | PD-L1 | lung cancers |
| NCT02352948 | NCT02352948-0 | Tremelimumab | CTLA-4 | lung cancers |
| NCT02354131 | NCT02354131-0 | Niraparib | PARP | Ovary cancer |
| NCT02354131 | NCT02354131-0 | Bevacizumab | VEGF | Ovary cancer |
| NCT02358356 | NCT02358356-0 | Capecitabine | chemotherapy | Brain and nervous system cancers |
| NCT02358356 | NCT02358356-0 | Lutetium Lu 177 Dotatate | SSTR | Brain and nervous system cancers |
| NCT02358473 | NCT02358473-0 | Mogamulizumab | CCR4 | lung cancers |
| NCT02358473 | NCT02358473-0 | Docetaxel | chemotherapy | lung cancers |
| NCT02362035 | NCT02362035-0 | Acalabrutinib | BTK | Other hematologic Neoplasms |
| NCT02362035 | NCT02362035-0 | Pembrolizumab | PD-1 | Other hematologic Neoplasms |
| NCT02362048 | NCT02362048-0 | Acalabrutinib | BTK | Pancreas cancer |
| NCT02362048 | NCT02362048-0 | Pembrolizumab | PD-1 | Pancreas cancer |
| NCT02367040 | NCT02367040-0 | Copanlisib | PI3K | Lymphomas |
| NCT02367040 | NCT02367040-0 | Rituximab | CD20 | Lymphomas |
| NCT02367456 | NCT02367456-0 | Glasdegib | SMO | Other hematologic Neoplasms |
| NCT02367456 | NCT02367456-0 | Azacitidine | chemotherapy | Other hematologic Neoplasms |
| NCT02369874 | NCT02369874-0 | Durvalumab | PD-L1 | Head and Neck Neoplasms |
| NCT02369874 | NCT02369874-0 | Tremelimumab | CTLA-4 | Head and Neck Neoplasms |
| NCT02370706 | NCT02370706-0 | Lgh447 | PIM | Other hematologic Neoplasms |
| NCT02370706 | NCT02370706-0 | Ruxolitinib | JAK | Other hematologic Neoplasms |
| NCT02370706 | NCT02370706-0 | Ribociclib | CDK4/CDK6 | Other hematologic Neoplasms |
| NCT02379247 | NCT02379247-0 | Alpelisib | PI3K | Breast cancer |
| NCT02379247 | NCT02379247-0 | Paclitaxel | chemotherapy | Breast cancer |
| NCT02383212 | NCT02383212-1 | Cemiplimab-Rwlc | PD-1 | Other solid tumors |
| NCT02383212 | NCT02383212-1 | Cyclophosphamide | chemotherapy | Other solid tumors |
| NCT02383212 | NCT02383212-2 | Cemiplimab-Rwlc | PD-1 | Other solid tumors |
| NCT02383212 | NCT02383212-2 | Gm-Csf | CSF2 | Other solid tumors |
| NCT02383212 | NCT02383212-2 | Cyclophosphamide | chemotherapy | Other solid tumors |
| NCT02384850 | NCT02384850-0 | Selinexor | XPO1 | Colon and rectum cancers |
| NCT02384850 | NCT02384850-0 | Mfolfox6 | chemotherapy | Colon and rectum cancers |
| NCT02386826 | NCT02386826-0 | Capmatinib | MET | Other solid tumors |
| NCT02386826 | NCT02386826-0 | Bevacizumab | VEGF | Other solid tumors |
| NCT02392793 | NCT02392793-1 | Talazoparib | PARP | Other solid tumors |
| NCT02392793 | NCT02392793-1 | Irinotecan | chemotherapy | Other solid tumors |
| NCT02392793 | NCT02392793-2 | Talazoparib | PARP | Other solid tumors |
| NCT02392793 | NCT02392793-2 | Irinotecan | chemotherapy | Other solid tumors |
| NCT02392793 | NCT02392793-2 | Temozolomide | chemotherapy | Other solid tumors |
| NCT02393248 | NCT02393248-1 | Gemcitabine | chemotherapy | Other solid tumors |
| NCT02393248 | NCT02393248-1 | Platinum-Based Chemotherapy | chemotherapy | Other solid tumors |
| NCT02393248 | NCT02393248-1 | Pemigatinib | FGFR | Other solid tumors |
| NCT02393248 | NCT02393248-2 | Pembrolizumab | PD-1 | Other solid tumors |
| NCT02393248 | NCT02393248-2 | Pemigatinib | FGFR | Other solid tumors |
| NCT02393248 | NCT02393248-3 | Docetaxel | chemotherapy | Other solid tumors |
| NCT02393248 | NCT02393248-3 | Pemigatinib | FGFR | Other solid tumors |
| NCT02393248 | NCT02393248-4 | Trastuzumab | HER2 | Other solid tumors |
| NCT02393248 | NCT02393248-4 | Pemigatinib | FGFR | Other solid tumors |
| NCT02393248 | NCT02393248-5 | Retifanlimab | PD-1 | Other solid tumors |
| NCT02393248 | NCT02393248-5 | Pemigatinib | FGFR | Other solid tumors |
| NCT02395172 | NCT02395172-0 | Avelumab | PD-L1 | lung cancers |
| NCT02395172 | NCT02395172-0 | Docetaxel | chemotherapy | lung cancers |
| NCT02399085 | NCT02399085-0 | Tafasitamab | CD19 | Lymphomas |
| NCT02399085 | NCT02399085-0 | Lenalidomide | CRBN | Lymphomas |
| NCT02400476 | NCT02400476-0 | Neratinib | EGFR | Breast cancer |
| NCT02400476 | NCT02400476-0 | Loperamide | Other supportive therapy | Breast cancer |
| NCT02401048 | NCT02401048-0 | Ibrutinib | BTK | Lymphomas |
| NCT02401048 | NCT02401048-0 | Durvalumab | PD-L1 | Lymphomas |
| NCT02401815 | NCT02401815-0 | Plx9486 | KIT | Other solid tumors |
| NCT02401815 | NCT02401815-0 | Pexidartinib | CSF1R/PDGFR | Other solid tumors |
| NCT02403271 | NCT02403271-0 | Ibrutinib | BTK | Other solid tumors |
| NCT02403271 | NCT02403271-0 | Durvalumab | PD-L1 | Other solid tumors |
| NCT02403310 | NCT02403310-0 | Selinexor | XPO1 | Leukaemia |
| NCT02403310 | NCT02403310-0 | Daunorubicin | chemotherapy | Leukaemia |
| NCT02403310 | NCT02403310-0 | Cytarabine | chemotherapy | Leukaemia |
| NCT02411591 | NCT02411591-0 | Necitumumab | EGFR | lung cancers |
| NCT02411591 | NCT02411591-0 | Abemaciclib | CDK4/CDK6 | lung cancers |
| NCT02416908 | NCT02416908-0 | Selinexor | XPO1 | Leukaemia |
| NCT02416908 | NCT02416908-0 | Cladribine | chemotherapy | Leukaemia |
| NCT02416908 | NCT02416908-0 | Cytarabine | chemotherapy | Leukaemia |
| NCT02416908 | NCT02416908-0 | G-Csf | CSF3 | Leukaemia |
| NCT02419495 | NCT02419495-1 | Selinexor | XPO1 | Other solid tumors |
| NCT02419495 | NCT02419495-1 | Platinum-Based Chemotherapy | chemotherapy | Other solid tumors |
| NCT02419495 | NCT02419495-10 | Selinexor | XPO1 | Other solid tumors |
| NCT02419495 | NCT02419495-10 | Capecitabine | chemotherapy | Other solid tumors |
| NCT02419495 | NCT02419495-10 | Platinum-Based Chemotherapy | chemotherapy | Other solid tumors |
| NCT02419495 | NCT02419495-11 | Selinexor | XPO1 | Other solid tumors |
| NCT02419495 | NCT02419495-11 | Olaparib | PARP | Other solid tumors |
| NCT02419495 | NCT02419495-12 | Selinexor | XPO1 | Other solid tumors |
| NCT02419495 | NCT02419495-12 | Pembrolizumab | PD-1 | Other solid tumors |
| NCT02419495 | NCT02419495-13 | Selinexor | XPO1 | Other solid tumors |
| NCT02419495 | NCT02419495-13 | Nivolumab | PD-1 | Other solid tumors |
| NCT02419495 | NCT02419495-14 | Selinexor | XPO1 | Other solid tumors |
| NCT02419495 | NCT02419495-14 | Nivolumab | PD-1 | Other solid tumors |
| NCT02419495 | NCT02419495-14 | Ipilimumab | CTLA-4 | Other solid tumors |
| NCT02419495 | NCT02419495-2 | Selinexor | XPO1 | Other solid tumors |
| NCT02419495 | NCT02419495-2 | Paclitaxel | chemotherapy | Other solid tumors |
| NCT02419495 | NCT02419495-3 | Selinexor | XPO1 | Other solid tumors |
| NCT02419495 | NCT02419495-3 | Eribulin | chemotherapy | Other solid tumors |
| NCT02419495 | NCT02419495-4 | Selinexor | XPO1 | Other solid tumors |
| NCT02419495 | NCT02419495-4 | Doxorubicin | chemotherapy | Other solid tumors |
| NCT02419495 | NCT02419495-4 | Cyclophosphamide | chemotherapy | Other solid tumors |
| NCT02419495 | NCT02419495-5 | Selinexor | XPO1 | Other solid tumors |
| NCT02419495 | NCT02419495-5 | Platinum-Based Chemotherapy | chemotherapy | Other solid tumors |
| NCT02419495 | NCT02419495-5 | Paclitaxel | chemotherapy | Other solid tumors |
| NCT02419495 | NCT02419495-6 | Selinexor | XPO1 | Other solid tumors |
| NCT02419495 | NCT02419495-6 | Platinum-Based Chemotherapy | chemotherapy | Other solid tumors |
| NCT02419495 | NCT02419495-6 | Pemetrexed | chemotherapy | Other solid tumors |
| NCT02419495 | NCT02419495-7 | Selinexor | XPO1 | Other solid tumors |
| NCT02419495 | NCT02419495-7 | Topotecan | chemotherapy | Other solid tumors |
| NCT02419495 | NCT02419495-8 | Selinexor | XPO1 | Other solid tumors |
| NCT02419495 | NCT02419495-8 | Folfiri | chemotherapy | Other solid tumors |
| NCT02419495 | NCT02419495-9 | Selinexor | XPO1 | Other solid tumors |
| NCT02419495 | NCT02419495-9 | Irinotecan | chemotherapy | Other solid tumors |
| NCT02422615 | NCT02422615-0 | Ribociclib | CDK4/CDK6 | Breast cancer |
| NCT02422615 | NCT02422615-0 | Fulvestrant | Endocrine Therapy | Breast cancer |
| NCT02431559 | NCT02431559-1 | Doxorubicin | chemotherapy | Ovary cancer |
| NCT02431559 | NCT02431559-1 | Durvalumab | PD-L1 | Ovary cancer |
| NCT02431559 | NCT02431559-2 | Doxorubicin | chemotherapy | Ovary cancer |
| NCT02431559 | NCT02431559-2 | Durvalumab | PD-L1 | Ovary cancer |
| NCT02431559 | NCT02431559-2 | Motolimod | TLR | Ovary cancer |
| NCT02437318 | NCT02437318-0 | Fulvestrant | Endocrine Therapy | Breast cancer |
| NCT02437318 | NCT02437318-0 | Alpelisib | PI3K | Breast cancer |
| NCT02441946 | NCT02441946-0 | Abemaciclib | CDK4/CDK6 | Breast cancer |
| NCT02441946 | NCT02441946-0 | Anastrozole | Endocrine Therapy | Breast cancer |
| NCT02448290 | NCT02448290-0 | Docetaxel | chemotherapy | Stomach cancer |
| NCT02448290 | NCT02448290-0 | Selumetinib | MEK | Stomach cancer |
| NCT02448303 | NCT02448303-0 | Acalabrutinib | BTK | lung cancers |
| NCT02448303 | NCT02448303-0 | Pembrolizumab | PD-1 | lung cancers |
| NCT02448537 | NCT02448537-1 | Doxorubicin | chemotherapy | Other solid tumors |
| NCT02448537 | NCT02448537-1 | Lurbinectedin | chemotherapy | Other solid tumors |
| NCT02448537 | NCT02448537-2 | Gemcitabine | chemotherapy | Other solid tumors |
| NCT02448537 | NCT02448537-2 | Lurbinectedin | chemotherapy | Other solid tumors |
| NCT02450656 | NCT02450656-0 | Afatinib | EGFR | Other solid tumors |
| NCT02450656 | NCT02450656-0 | Selumetinib | MEK | Other solid tumors |
| NCT02453282 | NCT02453282-0 | Durvalumab | PD-L1 | lung cancers |
| NCT02453282 | NCT02453282-0 | Tremelimumab | CTLA-4 | lung cancers |
| NCT02454179 | NCT02454179-0 | Acalabrutinib | BTK | Head and Neck Neoplasms |
| NCT02454179 | NCT02454179-0 | Pembrolizumab | PD-1 | Head and Neck Neoplasms |
| NCT02454933 | NCT02454933-0 | Osimertinib | EGFR | lung cancers |
| NCT02454933 | NCT02454933-0 | Durvalumab | PD-L1 | lung cancers |
| NCT02471911 | NCT02471911-0 | Selinexor | XPO1 | Lymphomas |
| NCT02471911 | NCT02471911-0 | Rituximab | CD20 | Lymphomas |
| NCT02471911 | NCT02471911-0 | Chemotherapy | chemotherapy | Lymphomas |
| NCT02472275 | NCT02472275-0 | Pexidartinib | CSF1R/PDGFR | Prostate cancer |
| NCT02472275 | NCT02472275-0 | Androgen Deprivation Therapy (Adt) | Endocrine Therapy | Prostate cancer |
| NCT02475681 | NCT02475681-0 | Acalabrutinib | BTK | Leukaemia |
| NCT02475681 | NCT02475681-0 | Obinutuzumab | CD20 | Leukaemia |
| NCT02476123 | NCT02476123-0 | Mogamulizumab | CCR4 | Other solid tumors |
| NCT02476123 | NCT02476123-0 | Nivolumab | PD-1 | Other solid tumors |
| NCT02484404 | NCT02484404-1 | Durvalumab | PD-L1 | Other solid tumors |
| NCT02484404 | NCT02484404-1 | Cediranib | KDR | Other solid tumors |
| NCT02484404 | NCT02484404-2 | Durvalumab | PD-L1 | Other solid tumors |
| NCT02484404 | NCT02484404-2 | Olaparib | PARP | Other solid tumors |
| NCT02484404 | NCT02484404-3 | Durvalumab | PD-L1 | Other solid tumors |
| NCT02484404 | NCT02484404-3 | Olaparib | PARP | Other solid tumors |
| NCT02484404 | NCT02484404-3 | Cediranib | KDR | Other solid tumors |
| NCT02489318 | NCT02489318-0 | Apalutamide | Endocrine Therapy | Prostate cancer |
| NCT02489318 | NCT02489318-0 | Androgen Deprivation Therapy (Adt) | Endocrine Therapy | Prostate cancer |
| NCT02489448 | NCT02489448-0 | Durvalumab | PD-L1 | Breast cancer |
| NCT02489448 | NCT02489448-0 | Paclitaxel | chemotherapy | Breast cancer |
| NCT02489448 | NCT02489448-0 | Doxorubicin | chemotherapy | Breast cancer |
| NCT02489448 | NCT02489448-0 | Cyclophosphamide | chemotherapy | Breast cancer |
| NCT02493530 | NCT02493530-0 | Umbralisib | PI3K | Other hematologic Neoplasms |
| NCT02493530 | NCT02493530-0 | Ruxolitinib | JAK | Other hematologic Neoplasms |
| NCT02493751 | NCT02493751-0 | Axitinib | VEGFR/PDGFR | Kidney cancer |
| NCT02493751 | NCT02493751-0 | Avelumab | PD-L1 | Kidney cancer |
| NCT02494921 | NCT02494921-0 | Ribociclib | CDK4/CDK6 | Prostate cancer |
| NCT02494921 | NCT02494921-0 | Docetaxel | chemotherapy | Prostate cancer |
| NCT02494921 | NCT02494921-0 | Prednisone | Steroid Treatment | Prostate cancer |
| NCT02499328 | NCT02499328-1 | Danvatirsen | STAT3 | Head and Neck Neoplasms |
| NCT02499328 | NCT02499328-1 | Durvalumab | PD-L1 | Head and Neck Neoplasms |
| NCT02499328 | NCT02499328-2 | Azd5069 | CXCR2 | Head and Neck Neoplasms |
| NCT02499328 | NCT02499328-2 | Durvalumab | PD-L1 | Head and Neck Neoplasms |
| NCT02502786 | NCT02502786-0 | Naxitamab | GD-2 | Other solid tumors |
| NCT02502786 | NCT02502786-0 | Gm-Csf | CSF2 | Other solid tumors |
| NCT02503358 | NCT02503358-0 | Selumetinib | MEK | lung cancers |
| NCT02503358 | NCT02503358-0 | Paclitaxel | chemotherapy | lung cancers |
| NCT02503774 | NCT02503774-0 | Oleclumab | CD73 | Other solid tumors |
| NCT02503774 | NCT02503774-0 | Durvalumab | PD-L1 | Other solid tumors |
| NCT02510001 | NCT02510001-0 | Binimetinib | MEK | Colon and rectum cancers |
| NCT02510001 | NCT02510001-0 | Crizotinib | ALK/MET | Colon and rectum cancers |
| NCT02513186 | NCT02513186-0 | Isatuximab | CD38 | Multiple Myeloma |
| NCT02513186 | NCT02513186-0 | Bortezomib | PSMB | Multiple Myeloma |
| NCT02513186 | NCT02513186-0 | Cyclophosphamide | chemotherapy | Multiple Myeloma |
| NCT02513186 | NCT02513186-0 | Dexamethasone | Steroid Treatment | Multiple Myeloma |
| NCT02516241 | NCT02516241-0 | Durvalumab | PD-L1 | Bladder cancer |
| NCT02516241 | NCT02516241-0 | Tremelimumab | CTLA-4 | Bladder cancer |
| NCT02519348 | NCT02519348-1 | Durvalumab | PD-L1 | Liver cancer |
| NCT02519348 | NCT02519348-1 | Tremelimumab | CTLA-4 | Liver cancer |
| NCT02519348 | NCT02519348-2 | Durvalumab | PD-L1 | Liver cancer |
| NCT02519348 | NCT02519348-2 | Bevacizumab | VEGF | Liver cancer |
| NCT02527434 | NCT02527434-0 | Durvalumab | PD-L1 | Other solid tumors |
| NCT02527434 | NCT02527434-0 | Tremelimumab | CTLA-4 | Other solid tumors |
| NCT02530476 | NCT02530476-0 | Selinexor | XPO1 | Leukaemia |
| NCT02530476 | NCT02530476-0 | Sorafenib | VEGFR/PDGFR/FGFR/RAF | Leukaemia |
| NCT02531516 | NCT02531516-0 | Apalutamide | Endocrine Therapy | Prostate cancer |
| NCT02531516 | NCT02531516-0 | Bicalutamide | Endocrine Therapy | Prostate cancer |
| NCT02535078 | NCT02535078-0 | Imcgp100 | cell therapy | Melanoma |
| NCT02535078 | NCT02535078-0 | Durvalumab | PD-L1 | Melanoma |
| NCT02535078 | NCT02535078-0 | Tremelimumab | CTLA-4 | Melanoma |
| NCT02535247 | NCT02535247-0 | Pembrolizumab | PD-1 | Lymphomas |
| NCT02535247 | NCT02535247-0 | Copanlisib | PI3K | Lymphomas |
| NCT02535286 | NCT02535286-0 | Tg-1501 | PD-L1 | Leukaemia |
| NCT02535286 | NCT02535286-0 | Ublituximab | CD20 | Leukaemia |
| NCT02535286 | NCT02535286-0 | Umbralisib | PI3K | Leukaemia |
| NCT02536794 | NCT02536794-0 | Durvalumab | PD-L1 | Breast cancer |
| NCT02536794 | NCT02536794-0 | Tremelimumab | CTLA-4 | Breast cancer |
| NCT02537223 | NCT02537223-0 | Alpelisib | PI3K | Head and Neck Neoplasms |
| NCT02537223 | NCT02537223-0 | Platinum-Based Chemotherapy | chemotherapy | Head and Neck Neoplasms |
| NCT02537418 | NCT02537418-0 | Durvalumab | PD-L1 | Other solid tumors |
| NCT02537418 | NCT02537418-0 | Tremelimumab | CTLA-4 | Other solid tumors |
| NCT02537444 | NCT02537444-0 | Acalabrutinib | BTK | Ovary cancer |
| NCT02537444 | NCT02537444-0 | Pembrolizumab | PD-1 | Ovary cancer |
| NCT02542293 | NCT02542293-0 | Durvalumab | PD-L1 | lung cancers |
| NCT02542293 | NCT02542293-0 | Tremelimumab | CTLA-4 | lung cancers |
| NCT02546661 | NCT02546661-1 | Durvalumab | PD-L1 | Bladder cancer |
| NCT02546661 | NCT02546661-1 | Azd4547 | FGFR | Bladder cancer |
| NCT02546661 | NCT02546661-2 | Durvalumab | PD-L1 | Bladder cancer |
| NCT02546661 | NCT02546661-2 | Olaparib | PARP | Bladder cancer |
| NCT02546661 | NCT02546661-3 | Durvalumab | PD-L1 | Bladder cancer |
| NCT02546661 | NCT02546661-3 | Azd1775 | WEE | Bladder cancer |
| NCT02546661 | NCT02546661-4 | Durvalumab | PD-L1 | Bladder cancer |
| NCT02546661 | NCT02546661-4 | Vistusertib | mTOR | Bladder cancer |
| NCT02546661 | NCT02546661-5 | Durvalumab | PD-L1 | Bladder cancer |
| NCT02546661 | NCT02546661-5 | Danvatirsen | STAT3 | Bladder cancer |
| NCT02546661 | NCT02546661-6 | Durvalumab | PD-L1 | Bladder cancer |
| NCT02546661 | NCT02546661-6 | Selumetinib | MEK | Bladder cancer |
| NCT02549651 | NCT02549651-1 | Durvalumab | PD-L1 | Lymphomas |
| NCT02549651 | NCT02549651-1 | Tremelimumab | CTLA-4 | Lymphomas |
| NCT02549651 | NCT02549651-2 | Durvalumab | PD-L1 | Lymphomas |
| NCT02549651 | NCT02549651-2 | Danvatirsen | STAT3 | Lymphomas |
| NCT02551159 | NCT02551159-0 | Durvalumab | PD-L1 | Head and Neck Neoplasms |
| NCT02551159 | NCT02551159-0 | Tremelimumab | CTLA-4 | Head and Neck Neoplasms |
| NCT02554812 | NCT02554812-1 | Avelumab | PD-L1 | Other solid tumors |
| NCT02554812 | NCT02554812-1 | Utomilumab | 4-1BB | Other solid tumors |
| NCT02554812 | NCT02554812-2 | Pf-04518600 | OX40 | Other solid tumors |
| NCT02554812 | NCT02554812-2 | Avelumab | PD-L1 | Other solid tumors |
| NCT02554812 | NCT02554812-3 | Pd0360324 | CSF1 | Other solid tumors |
| NCT02554812 | NCT02554812-3 | Avelumab | PD-L1 | Other solid tumors |
| NCT02554812 | NCT02554812-4 | Utomilumab | 4-1BB | Other solid tumors |
| NCT02554812 | NCT02554812-4 | Pf-04518600 | OX40 | Other solid tumors |
| NCT02554812 | NCT02554812-4 | Avelumab | PD-L1 | Other solid tumors |
| NCT02554812 | NCT02554812-5 | Cmp-001 | TLR | Other solid tumors |
| NCT02554812 | NCT02554812-5 | Avelumab | PD-L1 | Other solid tumors |
| NCT02554812 | NCT02554812-6 | Cmp-001 | TLR | Other solid tumors |
| NCT02554812 | NCT02554812-6 | Avelumab | PD-L1 | Other solid tumors |
| NCT02554812 | NCT02554812-6 | Utomilumab | 4-1BB | Other solid tumors |
| NCT02554812 | NCT02554812-7 | Cmp-001 | TLR | Other solid tumors |
| NCT02554812 | NCT02554812-7 | Avelumab | PD-L1 | Other solid tumors |
| NCT02554812 | NCT02554812-7 | Pf-04518600 | OX40 | Other solid tumors |
| NCT02555189 | NCT02555189-0 | Enzalutamide | Endocrine Therapy | Prostate cancer |
| NCT02555189 | NCT02555189-0 | Ribociclib | CDK4/CDK6 | Prostate cancer |
| NCT02558894 | NCT02558894-0 | Tremelimumab | CTLA-4 | Pancreas cancer |
| NCT02558894 | NCT02558894-0 | Durvalumab | PD-L1 | Pancreas cancer |
| NCT02566993 | NCT02566993-0 | Lurbinectedin | chemotherapy | lung cancers |
| NCT02566993 | NCT02566993-0 | Doxorubicin | chemotherapy | lung cancers |
| NCT02569476 | NCT02569476-0 | Zanubrutinib | BTK | Lymphomas |
| NCT02569476 | NCT02569476-0 | Obinutuzumab | CD20 | Lymphomas |
| NCT02572687 | NCT02572687-0 | Ramucirumab | KDR | Other solid tumors |
| NCT02572687 | NCT02572687-0 | Durvalumab | PD-L1 | Other solid tumors |
| NCT02572843 | NCT02572843-0 | Durvalumab | PD-L1 | lung cancers |
| NCT02572843 | NCT02572843-0 | Chemotherapy | chemotherapy | lung cancers |
| NCT02573363 | NCT02573363-0 | Selinexor | XPO1 | Leukaemia |
| NCT02573363 | NCT02573363-0 | Cytarabine | chemotherapy | Leukaemia |
| NCT02573363 | NCT02573363-0 | Mitoxantrone | chemotherapy | Leukaemia |
| NCT02574663 | NCT02574663-1 | Umbralisib | PI3K | Other solid tumors |
| NCT02574663 | NCT02574663-1 | Paclitaxel | chemotherapy | Other solid tumors |
| NCT02574663 | NCT02574663-1 | Gemcitabine | chemotherapy | Other solid tumors |
| NCT02574663 | NCT02574663-2 | Umbralisib | PI3K | Other solid tumors |
| NCT02574663 | NCT02574663-2 | Folfox | chemotherapy | Other solid tumors |
| NCT02574663 | NCT02574663-3 | Umbralisib | PI3K | Other solid tumors |
| NCT02574663 | NCT02574663-3 | Folfox | chemotherapy | Other solid tumors |
| NCT02574663 | NCT02574663-3 | Bevacizumab | VEGF | Other solid tumors |
| NCT02580058 | NCT02580058-0 | Avelumab | PD-L1 | Ovary cancer |
| NCT02580058 | NCT02580058-0 | Doxorubicin | chemotherapy | Ovary cancer |
| NCT02583477 | NCT02583477-1 | Durvalumab | PD-L1 | Pancreas cancer |
| NCT02583477 | NCT02583477-1 | Paclitaxel | chemotherapy | Pancreas cancer |
| NCT02583477 | NCT02583477-1 | Gemcitabine | chemotherapy | Pancreas cancer |
| NCT02583477 | NCT02583477-2 | Durvalumab | PD-L1 | Pancreas cancer |
| NCT02583477 | NCT02583477-2 | Azd5069 | CXCR2 | Pancreas cancer |
| NCT02583542 | NCT02583542-0 | Selumetinib | MEK | Other solid tumors |
| NCT02583542 | NCT02583542-0 | Vistusertib | mTOR | Other solid tumors |
| NCT02584634 | NCT02584634-1 | Avelumab | PD-L1 | lung cancers |
| NCT02584634 | NCT02584634-1 | Crizotinib | ALK/MET | lung cancers |
| NCT02584634 | NCT02584634-2 | Avelumab | PD-L1 | lung cancers |
| NCT02584634 | NCT02584634-2 | Pf-06463922 | ALK | lung cancers |
| NCT02584647 | NCT02584647-0 | Pexidartinib | CSF1R/PDGFR | Other solid tumors |
| NCT02584647 | NCT02584647-0 | Sirolimus | mTOR | Other solid tumors |
| NCT02584829 | NCT02584829-0 | Avelumab | PD-L1 | Other solid tumors |
| NCT02584829 | NCT02584829-0 | Cell | cell therapy | Other solid tumors |
| NCT02586675 | NCT02586675-0 | Tamoxifen | Endocrine Therapy | Breast cancer |
| NCT02586675 | NCT02586675-0 | Ribociclib | CDK4/CDK6 | Breast cancer |
| NCT02586675 | NCT02586675-0 | Goserelin | Endocrine Therapy | Breast cancer |
| NCT02586987 | NCT02586987-1 | Selumetinib | MEK | Other solid tumors |
| NCT02586987 | NCT02586987-1 | Durvalumab | PD-L1 | Other solid tumors |
| NCT02586987 | NCT02586987-1 | Tremelimumab | CTLA-4 | Other solid tumors |
| NCT02586987 | NCT02586987-2 | Selumetinib | MEK | Other solid tumors |
| NCT02586987 | NCT02586987-2 | Durvalumab | PD-L1 | Other solid tumors |
| NCT02588131 | NCT02588131-0 | Tremelimumab | CTLA-4 | Other solid tumors |
| NCT02588131 | NCT02588131-0 | Durvalumab | PD-L1 | Other solid tumors |
| NCT02592551 | NCT02592551-0 | Tremelimumab | CTLA-4 | Other solid tumors |
| NCT02592551 | NCT02592551-0 | Durvalumab | PD-L1 | Other solid tumors |
| NCT02599363 | NCT02599363-0 | Ribociclib | CDK4/CDK6 | Breast cancer |
| NCT02599363 | NCT02599363-0 | Paclitaxel | chemotherapy | Breast cancer |
| NCT02600897 | NCT02600897-0 | Polatuzumab Vedotin | CD79B/Tubulin | Lymphomas |
| NCT02600897 | NCT02600897-0 | Lenalidomide | CRBN | Lymphomas |
| NCT02600897 | NCT02600897-0 | Obinutuzumab | CD20 | Lymphomas |
| NCT02608216 | NCT02608216-0 | Ribociclib | CDK4/CDK6 | Breast cancer |
| NCT02608216 | NCT02608216-0 | Paclitaxel | chemotherapy | Breast cancer |
| NCT02609776 | NCT02609776-1 | Amivantamab | EGFR/MET | lung cancers |
| NCT02609776 | NCT02609776-1 | Lazertinib | EGFR | lung cancers |
| NCT02609776 | NCT02609776-2 | Amivantamab | EGFR/MET | lung cancers |
| NCT02609776 | NCT02609776-2 | Lazertinib | EGFR | lung cancers |
| NCT02609776 | NCT02609776-2 | Platinum-Based Chemotherapy | chemotherapy | lung cancers |
| NCT02609776 | NCT02609776-2 | Pemetrexed | chemotherapy | lung cancers |
| NCT02611024 | NCT02611024-0 | Lurbinectedin | chemotherapy | Other solid tumors |
| NCT02611024 | NCT02611024-0 | Irinotecan | chemotherapy | Other solid tumors |
| NCT02611323 | NCT02611323-1 | Obinutuzumab | CD20 | Lymphomas |
| NCT02611323 | NCT02611323-1 | Polatuzumab Vedotin | CD79B/Tubulin | Lymphomas |
| NCT02611323 | NCT02611323-1 | Venetoclax | BCL2 | Lymphomas |
| NCT02611323 | NCT02611323-2 | Rituximab | CD20 | Lymphomas |
| NCT02611323 | NCT02611323-2 | Polatuzumab Vedotin | CD79B/Tubulin | Lymphomas |
| NCT02611323 | NCT02611323-2 | Venetoclax | BCL2 | Lymphomas |
| NCT02612311 | NCT02612311-0 | Ublituximab | CD20 | Leukaemia |
| NCT02612311 | NCT02612311-0 | Umbralisib | PI3K | Leukaemia |
| NCT02613650 | NCT02613650-0 | Binimetinib | MEK | Colon and rectum cancers |
| NCT02613650 | NCT02613650-0 | Mfolfiri | chemotherapy | Colon and rectum cancers |
| NCT02614794 | NCT02614794-0 | Tucatinib | HER2 | Breast cancer |
| NCT02614794 | NCT02614794-0 | Capecitabine | chemotherapy | Breast cancer |
| NCT02614794 | NCT02614794-0 | Trastuzumab | HER2 | Breast cancer |
| NCT02616640 | NCT02616640-1 | Durvalumab | PD-L1 | Multiple Myeloma |
| NCT02616640 | NCT02616640-1 | Pomalidomide | CRBN | Multiple Myeloma |
| NCT02616640 | NCT02616640-2 | Durvalumab | PD-L1 | Multiple Myeloma |
| NCT02616640 | NCT02616640-2 | Pomalidomide | CRBN | Multiple Myeloma |
| NCT02616640 | NCT02616640-2 | Dexamethasone | Steroid Treatment | Multiple Myeloma |
| NCT02617277 | NCT02617277-0 | Azd1775 | WEE | Other solid tumors |
| NCT02617277 | NCT02617277-0 | Durvalumab | PD-L1 | Other solid tumors |
| NCT02626455 | NCT02626455-1 | Copanlisib | PI3K | Lymphomas |
| NCT02626455 | NCT02626455-1 | Rituximab | CD20 | Lymphomas |
| NCT02626455 | NCT02626455-1 | Bendamustine | chemotherapy | Lymphomas |
| NCT02626455 | NCT02626455-2 | Copanlisib | PI3K | Lymphomas |
| NCT02626455 | NCT02626455-2 | Rituximab | CD20 | Lymphomas |
| NCT02626455 | NCT02626455-2 | Chemotherapy | chemotherapy | Lymphomas |
| NCT02628132 | NCT02628132-0 | Durvalumab | PD-L1 | Breast cancer |
| NCT02628132 | NCT02628132-0 | Paclitaxel | chemotherapy | Breast cancer |
| NCT02630368 | NCT02630368-1 | Pexastimogene Devacirepvec | oncolytic virus | Other solid tumors |
| NCT02630368 | NCT02630368-1 | Cyclophosphamide | chemotherapy | Other solid tumors |
| NCT02630368 | NCT02630368-2 | Avelumab | PD-L1 | Other solid tumors |
| NCT02630368 | NCT02630368-2 | Pexastimogene Devacirepvec | oncolytic virus | Other solid tumors |
| NCT02630368 | NCT02630368-2 | Cyclophosphamide | chemotherapy | Other solid tumors |
| NCT02631447 | NCT02631447-0 | Binimetinib | MEK | Melanoma |
| NCT02631447 | NCT02631447-0 | Encorafenib | RAF | Melanoma |
| NCT02631447 | NCT02631447-0 | Nivolumab | PD-1 | Melanoma |
| NCT02631447 | NCT02631447-0 | Ipilimumab | CTLA-4 | Melanoma |
| NCT02631590 | NCT02631590-0 | Platinum-Based Chemotherapy | chemotherapy | Gallbladder and biliary tract cancer |
| NCT02631590 | NCT02631590-0 | Gemcitabine | chemotherapy | Gallbladder and biliary tract cancer |
| NCT02631590 | NCT02631590-0 | Copanlisib | PI3K | Gallbladder and biliary tract cancer |
| NCT02632045 | NCT02632045-0 | Fulvestrant | Endocrine Therapy | Breast cancer |
| NCT02632045 | NCT02632045-0 | Ribociclib | CDK4/CDK6 | Breast cancer |
| NCT02632708 | NCT02632708-1 | Ivosidenib | IDH | Leukaemia |
| NCT02632708 | NCT02632708-1 | Cytarabine | chemotherapy | Leukaemia |
| NCT02632708 | NCT02632708-1 | Idarubicin | Chemotherapy | Leukaemia |
| NCT02632708 | NCT02632708-2 | Enasidenib | IDH | Leukaemia |
| NCT02632708 | NCT02632708-2 | Cytarabine | chemotherapy | Leukaemia |
| NCT02632708 | NCT02632708-2 | Idarubicin | Chemotherapy | Leukaemia |
| NCT02639026 | NCT02639026-0 | Durvalumab | PD-L1 | Other solid tumors |
| NCT02639026 | NCT02639026-0 | Tremelimumab | CTLA-4 | Other solid tumors |
| NCT02639910 | NCT02639910-1 | Tafasitamab | CD19 | Leukaemia |
| NCT02639910 | NCT02639910-1 | Idelalisib | PI3K | Leukaemia |
| NCT02639910 | NCT02639910-2 | Tafasitamab | CD19 | Leukaemia |
| NCT02639910 | NCT02639910-2 | Venetoclax | BCL2 | Leukaemia |
| NCT02643303 | NCT02643303-1 | Durvalumab | PD-L1 | Other solid tumors |
| NCT02643303 | NCT02643303-1 | Polyiclc | TLR | Other solid tumors |
| NCT02643303 | NCT02643303-2 | Durvalumab | PD-L1 | Other solid tumors |
| NCT02643303 | NCT02643303-2 | Polyiclc | TLR | Other solid tumors |
| NCT02643303 | NCT02643303-2 | Tremelimumab | CTLA-4 | Other solid tumors |
| NCT02645149 | NCT02645149-0 | Ribociclib | CDK4/CDK6 | Melanoma |
| NCT02645149 | NCT02645149-0 | Trametinib | MEK | Melanoma |
| NCT02649686 | NCT02649686-0 | Durvalumab | PD-L1 | Breast cancer |
| NCT02649686 | NCT02649686-0 | Trastuzumab | HER2 | Breast cancer |
| NCT02650648 | NCT02650648-0 | Naxitamab | GD-2 | Brain and nervous system cancers |
| NCT02650648 | NCT02650648-0 | Cyclophosphamide | chemotherapy | Brain and nervous system cancers |
| NCT02650648 | NCT02650648-0 | Nk Cell | cell therapy | Brain and nervous system cancers |
| NCT02651662 | NCT02651662-0 | Cemiplimab-Rwlc | PD-1 | Lymphomas |
| NCT02651662 | NCT02651662-0 | Regn1979 | CD20/CD3 | Lymphomas |
| NCT02656303 | NCT02656303-0 | Ublituximab | CD20 | Leukaemia |
| NCT02656303 | NCT02656303-0 | Umbralisib | PI3K | Leukaemia |
| NCT02657343 | NCT02657343-0 | Ribociclib | CDK4/CDK6 | Breast cancer |
| NCT02657343 | NCT02657343-0 | Trastuzumab | HER2 | Breast cancer |
| NCT02657889 | NCT02657889-0 | Niraparib | PARP | Other solid tumors |
| NCT02657889 | NCT02657889-0 | Pembrolizumab | PD-1 | Other solid tumors |
| NCT02657928 | NCT02657928-0 | Ribociclib | CDK4/CDK6 | Other solid tumors |
| NCT02657928 | NCT02657928-0 | Letrozole | Endocrine Therapy | Other solid tumors |
| NCT02658214 | NCT02658214-0 | Durvalumab | PD-L1 | Other solid tumors |
| NCT02658214 | NCT02658214-0 | Tremelimumab | CTLA-4 | Other solid tumors |
| NCT02658214 | NCT02658214-0 | Chemotherapy | chemotherapy | Other solid tumors |
| NCT02661022 | NCT02661022-0 | Tagraxofusp-Erzs | CD123 | Multiple Myeloma |
| NCT02661022 | NCT02661022-0 | Pomalidomide | CRBN | Multiple Myeloma |
| NCT02661022 | NCT02661022-0 | Dexamethasone | Steroid Treatment | Multiple Myeloma |
| NCT02664935 | NCT02664935-1 | Selumetinib | MEK | lung cancers |
| NCT02664935 | NCT02664935-1 | Docetaxel | chemotherapy | lung cancers |
| NCT02664935 | NCT02664935-2 | Azd6738 | ATR | lung cancers |
| NCT02664935 | NCT02664935-2 | Durvalumab | PD-L1 | lung cancers |
| NCT02671435 | NCT02671435-0 | Durvalumab | PD-L1 | Other solid tumors |
| NCT02671435 | NCT02671435-0 | Monalizumab | NKG2A | Other solid tumors |
| NCT02675231 | NCT02675231-1 | Abemaciclib | CDK4/CDK6 | Breast cancer |
| NCT02675231 | NCT02675231-1 | Trastuzumab | HER2 | Breast cancer |
| NCT02675231 | NCT02675231-1 | Fulvestrant | Endocrine Therapy | Breast cancer |
| NCT02675231 | NCT02675231-2 | Abemaciclib | CDK4/CDK6 | Breast cancer |
| NCT02675231 | NCT02675231-2 | Trastuzumab | HER2 | Breast cancer |
| NCT02677922 | NCT02677922-1 | Ivosidenib | IDH | Leukaemia |
| NCT02677922 | NCT02677922-1 | Azacitidine | chemotherapy | Leukaemia |
| NCT02677922 | NCT02677922-2 | Enasidenib | IDH | Leukaemia |
| NCT02677922 | NCT02677922-2 | Azacitidine | chemotherapy | Leukaemia |
| NCT02678182 | NCT02678182-0 | Capecitabine | chemotherapy | Other solid tumors |
| NCT02678182 | NCT02678182-0 | Ramucirumab | KDR | Other solid tumors |
| NCT02684006 | NCT02684006-0 | Avelumab | PD-L1 | Kidney cancer |
| NCT02684006 | NCT02684006-0 | Axitinib | VEGFR/PDGFR | Kidney cancer |
| NCT02684318 | NCT02684318-0 | Lurbinectedin | chemotherapy | Other solid tumors |
| NCT02684318 | NCT02684318-0 | Olaparib | PARP | Other solid tumors |
| NCT02685059 | NCT02685059-0 | Durvalumab | PD-L1 | Breast cancer |
| NCT02685059 | NCT02685059-0 | Chemotherapy | chemotherapy | Breast cancer |
| NCT02685657 | NCT02685657-0 | Docetaxel | chemotherapy | Breast cancer |
| NCT02685657 | NCT02685657-0 | Selumetinib | MEK | Breast cancer |
| NCT02685657 | NCT02685657-0 | Doxorubicin | chemotherapy | Breast cancer |
| NCT02685657 | NCT02685657-0 | Cyclophosphamide | chemotherapy | Breast cancer |
| NCT02685826 | NCT02685826-0 | Durvalumab | PD-L1 | Multiple Myeloma |
| NCT02685826 | NCT02685826-0 | Lenalidomide | CRBN | Multiple Myeloma |
| NCT02685826 | NCT02685826-0 | Dexamethasone | Steroid Treatment | Multiple Myeloma |
| NCT02701400 | NCT02701400-0 | Durvalumab | PD-L1 | lung cancers |
| NCT02701400 | NCT02701400-0 | Tremelimumab | CTLA-4 | lung cancers |
| NCT02703623 | NCT02703623-1 | Abiraterone | CYP17A1 | Prostate cancer |
| NCT02703623 | NCT02703623-1 | Prednisone | Steroid Treatment | Prostate cancer |
| NCT02703623 | NCT02703623-1 | Apalutamide | Endocrine Therapy | Prostate cancer |
| NCT02703623 | NCT02703623-2 | Abiraterone | CYP17A1 | Prostate cancer |
| NCT02703623 | NCT02703623-2 | Prednisone | Steroid Treatment | Prostate cancer |
| NCT02703623 | NCT02703623-2 | Apalutamide | Endocrine Therapy | Prostate cancer |
| NCT02703623 | NCT02703623-2 | Ipilimumab | CTLA-4 | Prostate cancer |
| NCT02703623 | NCT02703623-3 | Abiraterone | CYP17A1 | Prostate cancer |
| NCT02703623 | NCT02703623-3 | Prednisone | Steroid Treatment | Prostate cancer |
| NCT02703623 | NCT02703623-3 | Apalutamide | Endocrine Therapy | Prostate cancer |
| NCT02703623 | NCT02703623-3 | Cabazitaxel | chemotherapy | Prostate cancer |
| NCT02703623 | NCT02703623-3 | Platinum-Based Chemotherapy | chemotherapy | Prostate cancer |
| NCT02705105 | NCT02705105-0 | Mogamulizumab | CCR4 | Liver cancer |
| NCT02705105 | NCT02705105-0 | Nivolumab | PD-1 | Liver cancer |
| NCT02705482 | NCT02705482-1 | Tavolixizumab | OX40 | Other solid tumors |
| NCT02705482 | NCT02705482-1 | Durvalumab | PD-L1 | Other solid tumors |
| NCT02705482 | NCT02705482-2 | Tavolixizumab | OX40 | Other solid tumors |
| NCT02705482 | NCT02705482-2 | Tremelimumab | CTLA-4 | Other solid tumors |
| NCT02705859 | NCT02705859-0 | Copanlisib | PI3K | Breast cancer |
| NCT02705859 | NCT02705859-0 | Trastuzumab | HER2 | Breast cancer |
| NCT02706405 | NCT02706405-0 | Jcar014 | cell therapy | Lymphomas |
| NCT02706405 | NCT02706405-0 | Durvalumab | PD-L1 | Lymphomas |
| NCT02712723 | NCT02712723-0 | Ribociclib | CDK4/CDK6 | Breast cancer |
| NCT02712723 | NCT02712723-0 | Letrozole | Endocrine Therapy | Breast cancer |
| NCT02717624 | NCT02717624-1 | Acalabrutinib | BTK | Lymphomas |
| NCT02717624 | NCT02717624-1 | Bendamustine | chemotherapy | Lymphomas |
| NCT02717624 | NCT02717624-1 | Rituximab | CD20 | Lymphomas |
| NCT02717624 | NCT02717624-2 | Acalabrutinib | BTK | Lymphomas |
| NCT02717624 | NCT02717624-2 | Venetoclax | BCL2 | Lymphomas |
| NCT02717624 | NCT02717624-2 | Rituximab | CD20 | Lymphomas |
| NCT02718911 | NCT02718911-0 | Ly3022855 | CSF1R | Other solid tumors |
| NCT02718911 | NCT02718911-0 | Durvalumab | PD-L1 | Other solid tumors |
| NCT02725489 | NCT02725489-0 | Vigil | vaccine | Other solid tumors |
| NCT02725489 | NCT02725489-0 | Durvalumab | PD-L1 | Other solid tumors |
| NCT02726997 | NCT02726997-0 | Durvalumab | PD-L1 | Ovary cancer |
| NCT02726997 | NCT02726997-0 | Platinum-Based Chemotherapy | chemotherapy | Ovary cancer |
| NCT02726997 | NCT02726997-0 | Paclitaxel | chemotherapy | Ovary cancer |
| NCT02729896 | NCT02729896-1 | Obinutuzumab | CD20 | Lymphomas |
| NCT02729896 | NCT02729896-1 | Atezolizumab | PD-L1 | Lymphomas |
| NCT02729896 | NCT02729896-1 | Polatuzumab Vedotin | CD79B/Tubulin | Lymphomas |
| NCT02729896 | NCT02729896-2 | Rituximab | CD20 | Lymphomas |
| NCT02729896 | NCT02729896-2 | Atezolizumab | PD-L1 | Lymphomas |
| NCT02729896 | NCT02729896-2 | Polatuzumab Vedotin | CD79B/Tubulin | Lymphomas |
| NCT02732119 | NCT02732119-0 | Ribociclib | CDK4/CDK6 | Breast cancer |
| NCT02732119 | NCT02732119-0 | Everolimus | mTOR | Breast cancer |
| NCT02732119 | NCT02732119-0 | Exemestane | Endocrine Therapy | Breast cancer |
| NCT02733042 | NCT02733042-1 | Durvalumab | PD-L1 | Other hematologic Neoplasms |
| NCT02733042 | NCT02733042-1 | Lenalidomide | CRBN | Other hematologic Neoplasms |
| NCT02733042 | NCT02733042-1 | Rituximab | CD20 | Other hematologic Neoplasms |
| NCT02733042 | NCT02733042-2 | Durvalumab | PD-L1 | Other hematologic Neoplasms |
| NCT02733042 | NCT02733042-2 | Ibrutinib | BTK | Other hematologic Neoplasms |
| NCT02733042 | NCT02733042-3 | Durvalumab | PD-L1 | Other hematologic Neoplasms |
| NCT02733042 | NCT02733042-3 | Rituximab | CD20 | Other hematologic Neoplasms |
| NCT02733042 | NCT02733042-3 | Bendamustine | chemotherapy | Other hematologic Neoplasms |
| NCT02734004 | NCT02734004-0 | Durvalumab | PD-L1 | Other solid tumors |
| NCT02734004 | NCT02734004-0 | Olaparib | PARP | Other solid tumors |
| NCT02734160 | NCT02734160-0 | Galunisertib | TGFbR1 | Pancreas cancer |
| NCT02734160 | NCT02734160-0 | Durvalumab | PD-L1 | Pancreas cancer |
| NCT02734615 | NCT02734615-1 | Lsz102 | Endocrine Therapy | Breast cancer |
| NCT02734615 | NCT02734615-1 | Ribociclib | CDK4/CDK6 | Breast cancer |
| NCT02734615 | NCT02734615-2 | Lsz102 | Endocrine Therapy | Breast cancer |
| NCT02734615 | NCT02734615-2 | Alpelisib | PI3K | Breast cancer |
| NCT02735239 | NCT02735239-1 | Durvalumab | PD-L1 | Other solid tumors |
| NCT02735239 | NCT02735239-1 | Chemotherapy | chemotherapy | Other solid tumors |
| NCT02735239 | NCT02735239-2 | Durvalumab | PD-L1 | Other solid tumors |
| NCT02735239 | NCT02735239-2 | Tremelimumab | CTLA-4 | Other solid tumors |
| NCT02735239 | NCT02735239-2 | Chemotherapy | chemotherapy | Other solid tumors |
| NCT02736500 | NCT02736500-0 | Lutetium Lu 177 Dotatate | SSTR | Brain and nervous system cancers |
| NCT02736500 | NCT02736500-0 | Capecitabine | chemotherapy | Brain and nervous system cancers |
| NCT02740985 | NCT02740985-1 | Imaradenant | A2aR/A2bR | Other solid tumors |
| NCT02740985 | NCT02740985-1 | Durvalumab | PD-L1 | Other solid tumors |
| NCT02740985 | NCT02740985-2 | Imaradenant | A2aR/A2bR | Other solid tumors |
| NCT02740985 | NCT02740985-2 | Durvalumab | PD-L1 | Other solid tumors |
| NCT02740985 | NCT02740985-2 | Oleclumab | CD73 | Other solid tumors |
| NCT02741388 | NCT02741388-0 | Selinexor | XPO1 | Lymphomas |
| NCT02741388 | NCT02741388-0 | Rituximab | CD20 | Lymphomas |
| NCT02741388 | NCT02741388-0 | Chemotherapy | chemotherapy | Lymphomas |
| NCT02745769 | NCT02745769-0 | Ramucirumab | KDR | Others |
| NCT02745769 | NCT02745769-0 | Abemaciclib | CDK4/CDK6 | Others |
| NCT02747004 | NCT02747004-0 | Abemaciclib | CDK4/CDK6 | Breast cancer |
| NCT02747004 | NCT02747004-0 | Tamoxifen | Endocrine Therapy | Breast cancer |
| NCT02752035 | NCT02752035-0 | Gilteritinib | FLT3 | Leukaemia |
| NCT02752035 | NCT02752035-0 | Azacitidine | chemotherapy | Leukaemia |
| NCT02754011 | NCT02754011-0 | Ribociclib | CDK4/CDK6 | Breast cancer |
| NCT02754011 | NCT02754011-0 | Capecitabine | chemotherapy | Breast cancer |
| NCT02754856 | NCT02754856-0 | Tremelimumab | CTLA-4 | Other solid tumors |
| NCT02754856 | NCT02754856-0 | Durvalumab | PD-L1 | Other solid tumors |
| NCT02762006 | NCT02762006-0 | Tremelimumab | CTLA-4 | Kidney cancer |
| NCT02762006 | NCT02762006-0 | Durvalumab | PD-L1 | Kidney cancer |
| NCT02763319 | NCT02763319-0 | Tafasitamab | CD19 | Lymphomas |
| NCT02763319 | NCT02763319-0 | Bendamustine | chemotherapy | Lymphomas |
| NCT02763566 | NCT02763566-1 | Abemaciclib | CDK4/CDK6 | Breast cancer |
| NCT02763566 | NCT02763566-1 | Aromatase Inhibitor | Endocrine Therapy | Breast cancer |
| NCT02763566 | NCT02763566-2 | Abemaciclib | CDK4/CDK6 | Breast cancer |
| NCT02763566 | NCT02763566-2 | Fulvestrant | Endocrine Therapy | Breast cancer |
| NCT02764333 | NCT02764333-0 | Tpiv200/Hufr-1 | vaccine | Ovary cancer |
| NCT02764333 | NCT02764333-0 | Durvalumab | PD-L1 | Ovary cancer |
| NCT02767063 | NCT02767063-0 | Pioglitazone | Antihyperglycemic | Leukaemia |
| NCT02767063 | NCT02767063-0 | Avelumab | PD-L1 | Leukaemia |
| NCT02770391 | NCT02770391-0 | Apalutamide | Endocrine Therapy | Prostate cancer |
| NCT02770391 | NCT02770391-0 | Leuprolide | Endocrine Therapy | Prostate cancer |
| NCT02772588 | NCT02772588-0 | Leuprolide | Endocrine Therapy | Prostate cancer |
| NCT02772588 | NCT02772588-0 | Abiraterone | CYP17A1 | Prostate cancer |
| NCT02772588 | NCT02772588-0 | Apalutamide | Endocrine Therapy | Prostate cancer |
| NCT02773459 | NCT02773459-0 | Binimetinib | MEK | Gallbladder and biliary tract cancer |
| NCT02773459 | NCT02773459-0 | Capecitabine | chemotherapy | Gallbladder and biliary tract cancer |
| NCT02775903 | NCT02775903-0 | Azacitidine | chemotherapy | Leukaemia |
| NCT02775903 | NCT02775903-0 | Durvalumab | PD-L1 | Leukaemia |
| NCT02777710 | NCT02777710-0 | Durvalumab | PD-L1 | Other solid tumors |
| NCT02777710 | NCT02777710-0 | Pexidartinib | CSF1R/PDGFR | Other solid tumors |
| NCT02779751 | NCT02779751-0 | Abemaciclib | CDK4/CDK6 | Other solid tumors |
| NCT02779751 | NCT02779751-0 | Pembrolizumab | PD-1 | Other solid tumors |
| NCT02780128 | NCT02780128-0 | Ceritinib | ALK | Brain and nervous system cancers |
| NCT02780128 | NCT02780128-0 | Ribociclib | CDK4/CDK6 | Brain and nervous system cancers |
| NCT02780609 | NCT02780609-0 | Selinexor | XPO1 | Multiple Myeloma |
| NCT02780609 | NCT02780609-0 | Melphalan | chemotherapy | Multiple Myeloma |
| NCT02783625 | NCT02783625-1 | Romidepsin | chemotherapy | Lymphomas |
| NCT02783625 | NCT02783625-1 | Duvelisib | PI3K | Lymphomas |
| NCT02783625 | NCT02783625-2 | Bortezomib | PSMB | Lymphomas |
| NCT02783625 | NCT02783625-2 | Duvelisib | PI3K | Lymphomas |
| NCT02784795 | NCT02784795-0 | Crenigacestat | NOTCH | Other solid tumors |
| NCT02784795 | NCT02784795-0 | Abemaciclib | CDK4/CDK6 | Other solid tumors |
| NCT02788773 | NCT02788773-0 | Durvalumab | PD-L1 | Prostate cancer |
| NCT02788773 | NCT02788773-0 | Tremelimumab | CTLA-4 | Prostate cancer |
| NCT02789878 | NCT02789878-0 | Goserelin | Endocrine Therapy | Prostate cancer |
| NCT02789878 | NCT02789878-0 | Prednisone | Steroid Treatment | Prostate cancer |
| NCT02789878 | NCT02789878-0 | Abiraterone | CYP17A1 | Prostate cancer |
| NCT02789878 | NCT02789878-0 | Apalutamide | Endocrine Therapy | Prostate cancer |
| NCT02791334 | NCT02791334-0 | Ly3300054 | PD-L1 | Other solid tumors |
| NCT02791334 | NCT02791334-0 | Abemaciclib | CDK4/CDK6 | Other solid tumors |
| NCT02793583 | NCT02793583-1 | Umbralisib | PI3K | Lymphomas |
| NCT02793583 | NCT02793583-1 | Ublituximab | CD20 | Lymphomas |
| NCT02793583 | NCT02793583-2 | Umbralisib | PI3K | Lymphomas |
| NCT02793583 | NCT02793583-2 | Ublituximab | CD20 | Lymphomas |
| NCT02793583 | NCT02793583-2 | Bendamustine | chemotherapy | Lymphomas |
| NCT02794883 | NCT02794883-0 | Durvalumab | PD-L1 | Brain and nervous system cancers |
| NCT02794883 | NCT02794883-0 | Tremelimumab | CTLA-4 | Brain and nervous system cancers |
| NCT02795182 | NCT02795182-0 | Zanubrutinib | BTK | Other hematologic Neoplasms |
| NCT02795182 | NCT02795182-0 | Tislelizumab | PD-1 | Other hematologic Neoplasms |
| NCT02795429 | NCT02795429-0 | Capmatinib | MET | Liver cancer |
| NCT02795429 | NCT02795429-0 | Spartalizumab | PD-1 | Liver cancer |
| NCT02799602 | NCT02799602-0 | Darolutamide | Endocrine Therapy | Prostate cancer |
| NCT02799602 | NCT02799602-0 | Androgen Deprivation Therapy (Adt) | Endocrine Therapy | Prostate cancer |
| NCT02799602 | NCT02799602-0 | Docetaxel | chemotherapy | Prostate cancer |
| NCT02802098 | NCT02802098-0 | Bevacizumab | VEGF | Breast cancer |
| NCT02802098 | NCT02802098-0 | Durvalumab | PD-L1 | Breast cancer |
| NCT02807454 | NCT02807454-0 | Daratumumab | CD38 | Multiple Myeloma |
| NCT02807454 | NCT02807454-0 | Durvalumab | PD-L1 | Multiple Myeloma |
| NCT02811497 | NCT02811497-0 | Azacitidine | chemotherapy | Other solid tumors |
| NCT02811497 | NCT02811497-0 | Durvalumab | PD-L1 | Other solid tumors |
| NCT02812420 | NCT02812420-0 | Durvalumab | PD-L1 | Other solid tumors |
| NCT02812420 | NCT02812420-0 | Tremelimumab | CTLA-4 | Other solid tumors |
| NCT02813135 | NCT02813135-0 | Ribociclib | CDK4/CDK6 | Others |
| NCT02813135 | NCT02813135-0 | Topotecan | chemotherapy | Others |
| NCT02813135 | NCT02813135-0 | Temozolomide | chemotherapy | Others |
| NCT02815995 | NCT02815995-0 | Durvalumab | PD-L1 | Other solid tumors |
| NCT02815995 | NCT02815995-0 | Tremelimumab | CTLA-4 | Other solid tumors |
| NCT02817633 | NCT02817633-1 | Tsr-022 | TIM3 | Other solid tumors |
| NCT02817633 | NCT02817633-1 | Nivolumab | PD-1 | Other solid tumors |
| NCT02817633 | NCT02817633-2 | Tsr-022 | TIM3 | Other solid tumors |
| NCT02817633 | NCT02817633-2 | Tsr-042 | PD-1 | Other solid tumors |
| NCT02817633 | NCT02817633-3 | Tsr-022 | TIM3 | Other solid tumors |
| NCT02817633 | NCT02817633-3 | Tsr-042 | PD-1 | Other solid tumors |
| NCT02817633 | NCT02817633-3 | Tsr-033 | LAG-3 | Other solid tumors |
| NCT02817633 | NCT02817633-4 | Tsr-022 | TIM3 | Other solid tumors |
| NCT02817633 | NCT02817633-4 | Tsr-042 | PD-1 | Other solid tumors |
| NCT02817633 | NCT02817633-4 | Docetaxel | chemotherapy | Other solid tumors |
| NCT02817633 | NCT02817633-5 | Tsr-022 | TIM3 | Other solid tumors |
| NCT02817633 | NCT02817633-5 | Tsr-042 | PD-1 | Other solid tumors |
| NCT02817633 | NCT02817633-5 | Pemetrexed | chemotherapy | Other solid tumors |
| NCT02817633 | NCT02817633-5 | Platinum-Based Chemotherapy | chemotherapy | Other solid tumors |
| NCT02819596 | NCT02819596-1 | Savolitinib | MET | Kidney cancer |
| NCT02819596 | NCT02819596-1 | Durvalumab | PD-L1 | Kidney cancer |
| NCT02819596 | NCT02819596-2 | Tremelimumab | CTLA-4 | Kidney cancer |
| NCT02819596 | NCT02819596-2 | Durvalumab | PD-L1 | Kidney cancer |
| NCT02821754 | NCT02821754-0 | Durvalumab | PD-L1 | Other solid tumors |
| NCT02821754 | NCT02821754-0 | Tremelimumab | CTLA-4 | Other solid tumors |
| NCT02826434 | NCT02826434-0 | Pvx-410 | vaccine | Breast cancer |
| NCT02826434 | NCT02826434-0 | Durvalumab | PD-L1 | Breast cancer |
| NCT02831686 | NCT02831686-0 | Selinexor | XPO1 | Multiple Myeloma |
| NCT02831686 | NCT02831686-0 | Ixazomib | PSMB | Multiple Myeloma |
| NCT02831686 | NCT02831686-0 | Dexamethasone | Steroid Treatment | Multiple Myeloma |
| NCT02834364 | NCT02834364-0 | Encorafenib | RAF | Multiple Myeloma |
| NCT02834364 | NCT02834364-0 | Binimetinib | MEK | Multiple Myeloma |
| NCT02835222 | NCT02835222-0 | Cytarabine | chemotherapy | Leukaemia |
| NCT02835222 | NCT02835222-0 | Daunorubicin | chemotherapy | Leukaemia |
| NCT02835222 | NCT02835222-0 | Selinexor | XPO1 | Leukaemia |
| NCT02849990 | NCT02849990-0 | Abiraterone | CYP17A1 | Other solid tumors |
| NCT02849990 | NCT02849990-0 | Apalutamide | Endocrine Therapy | Other solid tumors |
| NCT02849990 | NCT02849990-0 | Degarelix | Endocrine Therapy | Other solid tumors |
| NCT02849990 | NCT02849990-0 | Indomethacin | PTGS | Other solid tumors |
| NCT02857270 | NCT02857270-1 | Temuterkib | ERK | Other solid tumors |
| NCT02857270 | NCT02857270-1 | Abemaciclib | CDK4/CDK6 | Other solid tumors |
| NCT02857270 | NCT02857270-2 | Temuterkib | ERK | Other solid tumors |
| NCT02857270 | NCT02857270-2 | Encorafenib | RAF | Other solid tumors |
| NCT02857270 | NCT02857270-2 | Cetuximab | EGFR | Other solid tumors |
| NCT02867007 | NCT02867007-0 | Khk2455 | IDO1 | Other solid tumors |
| NCT02867007 | NCT02867007-0 | Mogamulizumab | CCR4 | Other solid tumors |
| NCT02867020 | NCT02867020-0 | Abiraterone | CYP17A1 | Prostate cancer |
| NCT02867020 | NCT02867020-0 | Prednisone | Steroid Treatment | Prostate cancer |
| NCT02867020 | NCT02867020-0 | Apalutamide | Endocrine Therapy | Prostate cancer |
| NCT02870920 | NCT02870920-0 | Durvalumab | PD-L1 | Colon and rectum cancers |
| NCT02870920 | NCT02870920-0 | Tremelimumab | CTLA-4 | Colon and rectum cancers |
| NCT02874404 | NCT02874404-0 | Umbralisib | PI3K | Lymphomas |
| NCT02874404 | NCT02874404-0 | Ibrutinib | BTK | Lymphomas |
| NCT02877303 | NCT02877303-0 | Blinatumomab | CD19/CD3 | Other hematologic Neoplasms |
| NCT02877303 | NCT02877303-0 | Inotuzumab | CD22 | Other hematologic Neoplasms |
| NCT02877303 | NCT02877303-0 | Chemotherapy | chemotherapy | Other hematologic Neoplasms |
| NCT02878785 | NCT02878785-0 | Decitabine | chemotherapy | Leukaemia |
| NCT02878785 | NCT02878785-0 | Talazoparib | PARP | Leukaemia |
| NCT02879162 | NCT02879162-0 | Durvalumab | PD-L1 | Others |
| NCT02879162 | NCT02879162-0 | Tremelimumab | CTLA-4 | Others |
| NCT02879318 | NCT02879318-0 | Gemcitabine | chemotherapy | Pancreas cancer |
| NCT02879318 | NCT02879318-0 | Paclitaxel | chemotherapy | Pancreas cancer |
| NCT02879318 | NCT02879318-0 | Durvalumab | PD-L1 | Pancreas cancer |
| NCT02879318 | NCT02879318-0 | Tremelimumab | CTLA-4 | Pancreas cancer |
| NCT02882308 | NCT02882308-0 | Durvalumab | PD-L1 | Head and Neck Neoplasms |
| NCT02882308 | NCT02882308-0 | Olaparib | PARP | Head and Neck Neoplasms |
| NCT02888743 | NCT02888743-0 | Durvalumab | PD-L1 | Other solid tumors |
| NCT02888743 | NCT02888743-0 | Tremelimumab | CTLA-4 | Other solid tumors |
| NCT02889523 | NCT02889523-0 | Tazemetostat | EZH2 | Lymphomas |
| NCT02889523 | NCT02889523-0 | Rituximab | CD20 | Lymphomas |
| NCT02889523 | NCT02889523-0 | Chemotherapy | chemotherapy | Lymphomas |
| NCT02898116 | NCT02898116-0 | Ensartinib | ALK | lung cancers |
| NCT02898116 | NCT02898116-0 | Durvalumab | PD-L1 | lung cancers |
| NCT02899195 | NCT02899195-0 | Durvalumab | PD-L1 | Other solid tumors |
| NCT02899195 | NCT02899195-0 | Chemotherapy | chemotherapy | Other solid tumors |
| NCT02902042 | NCT02902042-0 | Encorafenib | RAF | Melanoma |
| NCT02902042 | NCT02902042-0 | Binimetinib | MEK | Melanoma |
| NCT02902042 | NCT02902042-0 | Pembrolizumab | PD-1 | Melanoma |
| NCT02903368 | NCT02903368-0 | Abiraterone | CYP17A1 | Prostate cancer |
| NCT02903368 | NCT02903368-0 | Apalutamide | Endocrine Therapy | Prostate cancer |
| NCT02910700 | NCT02910700-0 | Binimetinib | MEK | Melanoma |
| NCT02910700 | NCT02910700-0 | Encorafenib | RAF | Melanoma |
| NCT02910700 | NCT02910700-0 | Nivolumab | PD-1 | Melanoma |
| NCT02912572 | NCT02912572-1 | Avelumab | PD-L1 | Corpus uteri cancer |
| NCT02912572 | NCT02912572-1 | Talazoparib | PARP | Corpus uteri cancer |
| NCT02912572 | NCT02912572-2 | Avelumab | PD-L1 | Corpus uteri cancer |
| NCT02912572 | NCT02912572-2 | Axitinib | VEGFR/PDGFR | Corpus uteri cancer |
| NCT02913196 | NCT02913196-0 | Apalutamide | Endocrine Therapy | Prostate cancer |
| NCT02913196 | NCT02913196-0 | Abiraterone | CYP17A1 | Prostate cancer |
| NCT02913196 | NCT02913196-0 | Docetaxel | chemotherapy | Prostate cancer |
| NCT02913196 | NCT02913196-0 | Prednisone | Steroid Treatment | Prostate cancer |
| NCT02914938 | NCT02914938-0 | Me-401 | PI3K | Other hematologic Neoplasms |
| NCT02914938 | NCT02914938-0 | Zanubrutinib | BTK | Other hematologic Neoplasms |
| NCT02915523 | NCT02915523-0 | Entinostat | chemotherapy | Ovary cancer |
| NCT02915523 | NCT02915523-0 | Avelumab | PD-L1 | Ovary cancer |
| NCT02923466 | NCT02923466-0 | Vsv-Ifnβ-Nis | oncolytic virus | Other solid tumors |
| NCT02923466 | NCT02923466-0 | Avelumab | PD-L1 | Other solid tumors |
| NCT02924766 | NCT02924766-1 | Niraparib | PARP | Prostate cancer |
| NCT02924766 | NCT02924766-1 | Apalutamide | Endocrine Therapy | Prostate cancer |
| NCT02924766 | NCT02924766-1 | Prednisone | Steroid Treatment | Prostate cancer |
| NCT02924766 | NCT02924766-2 | Niraparib | PARP | Prostate cancer |
| NCT02924766 | NCT02924766-2 | Abiraterone | CYP17A1 | Prostate cancer |
| NCT02924766 | NCT02924766-2 | Prednisone | Steroid Treatment | Prostate cancer |
| NCT02928224 | NCT02928224-0 | Encorafenib | RAF | Colon and rectum cancers |
| NCT02928224 | NCT02928224-0 | Binimetinib | MEK | Colon and rectum cancers |
| NCT02928224 | NCT02928224-0 | Cetuximab | EGFR | Colon and rectum cancers |
| NCT02937818 | NCT02937818-0 | Durvalumab | PD-L1 | lung cancers |
| NCT02937818 | NCT02937818-0 | Tremelimumab | CTLA-4 | lung cancers |
| NCT02938273 | NCT02938273-0 | Cetuximab | EGFR | Head and Neck Neoplasms |
| NCT02938273 | NCT02938273-0 | Avelumab | PD-L1 | Head and Neck Neoplasms |
| NCT02938793 | NCT02938793-0 | Durvalumab | PD-L1 | Others |
| NCT02938793 | NCT02938793-0 | Tremelimumab | CTLA-4 | Others |
| NCT02941926 | NCT02941926-0 | Ribociclib | CDK4/CDK6 | Breast cancer |
| NCT02941926 | NCT02941926-0 | Letrozole | Endocrine Therapy | Breast cancer |
| NCT02946671 | NCT02946671-0 | Mogamulizumab | CCR4 | Other solid tumors |
| NCT02946671 | NCT02946671-0 | Nivolumab | PD-1 | Other solid tumors |
| NCT02949284 | NCT02949284-0 | Apalutamide | Endocrine Therapy | Prostate cancer |
| NCT02949284 | NCT02949284-0 | Abiraterone | CYP17A1 | Prostate cancer |
| NCT02949284 | NCT02949284-0 | Gnrh Agonist | Endocrine Therapy | Prostate cancer |
| NCT02949284 | NCT02949284-0 | Prednisone | Steroid Treatment | Prostate cancer |
| NCT02953457 | NCT02953457-0 | Olaparib | PARP | Ovary cancer |
| NCT02953457 | NCT02953457-0 | Tremelimumab | CTLA-4 | Ovary cancer |
| NCT02953457 | NCT02953457-0 | Durvalumab | PD-L1 | Ovary cancer |
| NCT02962063 | NCT02962063-1 | Durvalumab | PD-L1 | Other solid tumors |
| NCT02962063 | NCT02962063-1 | Platinum-Based Chemotherapy | chemotherapy | Other solid tumors |
| NCT02962063 | NCT02962063-1 | Tremelimumab | CTLA-4 | Other solid tumors |
| NCT02962063 | NCT02962063-2 | Durvalumab | PD-L1 | Other solid tumors |
| NCT02962063 | NCT02962063-2 | Paclitaxel | chemotherapy | Other solid tumors |
| NCT02962063 | NCT02962063-2 | Tremelimumab | CTLA-4 | Other solid tumors |
| NCT02963493 | NCT02963493-0 | Melphalan | chemotherapy | Multiple Myeloma |
| NCT02963493 | NCT02963493-0 | Dexamethasone | Steroid Treatment | Multiple Myeloma |
| NCT02963831 | NCT02963831-0 | Oncos-102 | oncolytic virus | Other solid tumors |
| NCT02963831 | NCT02963831-0 | Durvalumab | PD-L1 | Other solid tumors |
| NCT02964689 | NCT02964689-0 | Binimetinib | MEK | lung cancers |
| NCT02964689 | NCT02964689-0 | Pemetrexed | chemotherapy | lung cancers |
| NCT02964689 | NCT02964689-0 | Platinum-Based Chemotherapy | chemotherapy | lung cancers |
| NCT02972840 | NCT02972840-0 | Acalabrutinib | BTK | Lymphomas |
| NCT02972840 | NCT02972840-0 | Bendamustine | chemotherapy | Lymphomas |
| NCT02972840 | NCT02972840-0 | Rituximab | CD20 | Lymphomas |
| NCT02974725 | NCT02974725-0 | Naporafenib | RAF | Other solid tumors |
| NCT02974725 | NCT02974725-0 | Ribociclib | CDK4/CDK6 | Other solid tumors |
| NCT02977780 | NCT02977780-0 | Neratinib | EGFR | Brain and nervous system cancers |
| NCT02977780 | NCT02977780-0 | Temozolomide | chemotherapy | Brain and nervous system cancers |
| NCT02978482 | NCT02978482-0 | Durvalumab | PD-L1 | Others |
| NCT02978482 | NCT02978482-0 | Tremelimumab | CTLA-4 | Others |
| NCT02981342 | NCT02981342-0 | Abemaciclib | CDK4/CDK6 | Pancreas cancer |
| NCT02981342 | NCT02981342-0 | Samotolisib | PI3K | Pancreas cancer |
| NCT02981628 | NCT02981628-0 | Inotuzumab Ozogamicin | CD22/DNA | Leukaemia |
| NCT02981628 | NCT02981628-0 | Mbfm | chemotherapy | Leukaemia |
| NCT02983578 | NCT02983578-0 | Danvatirsen | STAT3 | Other solid tumors |
| NCT02983578 | NCT02983578-0 | Durvalumab | PD-L1 | Other solid tumors |
| NCT02985125 | NCT02985125-0 | Ribociclib | CDK4/CDK6 | Pancreas cancer |
| NCT02985125 | NCT02985125-0 | Everolimus | mTOR | Pancreas cancer |
| NCT02990338 | NCT02990338-0 | Isatuximab | CD38 | Multiple Myeloma |
| NCT02990338 | NCT02990338-0 | Pomalidomide | CRBN | Multiple Myeloma |
| NCT02990338 | NCT02990338-0 | Dexamethasone | Steroid Treatment | Multiple Myeloma |
| NCT02997332 | NCT02997332-0 | Durvalumab | PD-L1 | Head and Neck Neoplasms |
| NCT02997332 | NCT02997332-0 | Docetaxel | chemotherapy | Head and Neck Neoplasms |
| NCT02997332 | NCT02997332-0 | Platinum-Based Chemotherapy | chemotherapy | Head and Neck Neoplasms |
| NCT02997332 | NCT02997332-0 | 5-Fu | chemotherapy | Head and Neck Neoplasms |
| NCT02997995 | NCT02997995-0 | Durvalumab | PD-L1 | Breast cancer |
| NCT02997995 | NCT02997995-0 | Exemestane | Endocrine Therapy | Breast cancer |
| NCT02999087 | NCT02999087-0 | Cetuximab | EGFR | Head and Neck Neoplasms |
| NCT02999087 | NCT02999087-0 | Avelumab | PD-L1 | Head and Neck Neoplasms |
| NCT03000452 | NCT03000452-0 | Daratumumab | CD38 | Multiple Myeloma |
| NCT03000452 | NCT03000452-0 | Durvalumab | PD-L1 | Multiple Myeloma |
| NCT03003520 | NCT03003520-0 | Durvalumab | PD-L1 | Lymphomas |
| NCT03003520 | NCT03003520-0 | Rituximab | CD20 | Lymphomas |
| NCT03003520 | NCT03003520-0 | Chemotherapy | chemotherapy | Lymphomas |
| NCT03005002 | NCT03005002-0 | Durvalumab | PD-L1 | Other solid tumors |
| NCT03005002 | NCT03005002-0 | Tremelimumab | CTLA-4 | Other solid tumors |
| NCT03005782 | NCT03005782-0 | Regn3767 | LAG-3 | Others |
| NCT03005782 | NCT03005782-0 | Cemiplimab-Rwlc | PD-1 | Others |
| NCT03007407 | NCT03007407-0 | Durvalumab | PD-L1 | Colon and rectum cancers |
| NCT03007407 | NCT03007407-0 | Tremelimumab | CTLA-4 | Colon and rectum cancers |
| NCT03008408 | NCT03008408-0 | Ribociclib | CDK4/CDK6 | Other solid tumors |
| NCT03008408 | NCT03008408-0 | Everolimus | mTOR | Other solid tumors |
| NCT03008408 | NCT03008408-0 | Letrozole | Endocrine Therapy | Other solid tumors |
| NCT03009201 | NCT03009201-0 | Ribociclib | CDK4/CDK6 | Others |
| NCT03009201 | NCT03009201-0 | Doxorubicin | chemotherapy | Others |
| NCT03009981 | NCT03009981-1 | Apalutamide | Endocrine Therapy | Prostate cancer |
| NCT03009981 | NCT03009981-1 | Abiraterone | CYP17A1 | Prostate cancer |
| NCT03009981 | NCT03009981-1 | Degarelix | Endocrine Therapy | Prostate cancer |
| NCT03009981 | NCT03009981-2 | Apalutamide | Endocrine Therapy | Prostate cancer |
| NCT03009981 | NCT03009981-2 | Abiraterone | CYP17A1 | Prostate cancer |
| NCT03009981 | NCT03009981-2 | Leuprolide | Endocrine Therapy | Prostate cancer |
| NCT03011814 | NCT03011814-0 | Durvalumab | PD-L1 | Lymphomas |
| NCT03011814 | NCT03011814-0 | Lenalidomide | CRBN | Lymphomas |
| NCT03015129 | NCT03015129-0 | Durvalumab | PD-L1 | Corpus uteri cancer |
| NCT03015129 | NCT03015129-0 | Tremelimumab | CTLA-4 | Corpus uteri cancer |
| NCT03016338 | NCT03016338-0 | Niraparib | PARP | Corpus uteri cancer |
| NCT03016338 | NCT03016338-0 | Dostarlimab-Gxly | PD-1 | Corpus uteri cancer |
| NCT03019003 | NCT03019003-0 | Decitabine | chemotherapy | Head and Neck Neoplasms |
| NCT03019003 | NCT03019003-0 | Durvalumab | PD-L1 | Head and Neck Neoplasms |
| NCT03022500 | NCT03022500-0 | Durvalumab | PD-L1 | lung cancers |
| NCT03022500 | NCT03022500-0 | Tremelimumab | CTLA-4 | lung cancers |
| NCT03026062 | NCT03026062-0 | Durvalumab | PD-L1 | Ovary cancer |
| NCT03026062 | NCT03026062-0 | Tremelimumab | CTLA-4 | Ovary cancer |
| NCT03033303 | NCT03033303-0 | Naxitamab | GD-2 | Brain and nervous system cancers |
| NCT03033303 | NCT03033303-0 | Gm-Csf | CSF2 | Brain and nervous system cancers |
| NCT03033303 | NCT03033303-0 | Isotretinoin | chemotherapy | Brain and nervous system cancers |
| NCT03040973 | NCT03040973-1 | Capmatinib | MET | Other solid tumors |
| NCT03040973 | NCT03040973-1 | Nazartinib | EGFR | Other solid tumors |
| NCT03040973 | NCT03040973-2 | Capmatinib | MET | Other solid tumors |
| NCT03040973 | NCT03040973-2 | Gefitinib | EGFR | Other solid tumors |
| NCT03042819 | NCT03042819-0 | Selinexor | XPO1 | Other solid tumors |
| NCT03042819 | NCT03042819-0 | Doxorubicin | chemotherapy | Other solid tumors |
| NCT03043313 | NCT03043313-0 | Tucatinib | HER2 | Colon and rectum cancers |
| NCT03043313 | NCT03043313-0 | Trastuzumab | HER2 | Colon and rectum cancers |
| NCT03043872 | NCT03043872-1 | Durvalumab | PD-L1 | lung cancers |
| NCT03043872 | NCT03043872-1 | Tremelimumab | CTLA-4 | lung cancers |
| NCT03043872 | NCT03043872-1 | Etoposide | chemotherapy | lung cancers |
| NCT03043872 | NCT03043872-1 | Cisplatin | chemotherapy | lung cancers |
| NCT03043872 | NCT03043872-2 | Durvalumab | PD-L1 | lung cancers |
| NCT03043872 | NCT03043872-2 | Etoposide | chemotherapy | lung cancers |
| NCT03043872 | NCT03043872-2 | Cisplatin | chemotherapy | lung cancers |
| NCT03046862 | NCT03046862-0 | Durvalumab | PD-L1 | Gallbladder and biliary tract cancer |
| NCT03046862 | NCT03046862-0 | Chemotherapy | chemotherapy | Gallbladder and biliary tract cancer |
| NCT03049735 | NCT03049735-0 | Relugolix | Endocrine Therapy | Other solid tumors |
| NCT03049735 | NCT03049735-0 | E2/Neta | Endocrine Therapy | Other solid tumors |
| NCT03050814 | NCT03050814-1 | Capecitabine | chemotherapy | Colon and rectum cancers |
| NCT03050814 | NCT03050814-1 | Bevacizumab | VEGF | Colon and rectum cancers |
| NCT03050814 | NCT03050814-1 | Avelumab | PD-L1 | Colon and rectum cancers |
| NCT03050814 | NCT03050814-2 | Folfox | chemotherapy | Colon and rectum cancers |
| NCT03050814 | NCT03050814-2 | Bevacizumab | VEGF | Colon and rectum cancers |
| NCT03050814 | NCT03050814-2 | Avelumab | PD-L1 | Colon and rectum cancers |
| NCT03050814 | NCT03050814-3 | Folfox | chemotherapy | Colon and rectum cancers |
| NCT03050814 | NCT03050814-3 | Bevacizumab | VEGF | Colon and rectum cancers |
| NCT03050814 | NCT03050814-3 | Avelumab | PD-L1 | Colon and rectum cancers |
| NCT03050814 | NCT03050814-3 | Ad-Cea Vaccine | vaccine | Colon and rectum cancers |
| NCT03051906 | NCT03051906-0 | Durvalumab | PD-L1 | Head and Neck Neoplasms |
| NCT03051906 | NCT03051906-0 | Cetuximab | EGFR | Head and Neck Neoplasms |
| NCT03052933 | NCT03052933-0 | Copanlisib | PI3K | Lymphomas |
| NCT03052933 | NCT03052933-0 | Gemcitabine | chemotherapy | Lymphomas |
| NCT03054363 | NCT03054363-0 | Tucatinib | HER2 | Breast cancer |
| NCT03054363 | NCT03054363-0 | Palbociclib | CDK4/CDK6 | Breast cancer |
| NCT03054363 | NCT03054363-0 | Letrozole | Endocrine Therapy | Breast cancer |
| NCT03054532 | NCT03054532-0 | Durvalumab | PD-L1 | Lymphomas |
| NCT03054532 | NCT03054532-0 | Lenalidomide | CRBN | Lymphomas |
| NCT03056755 | NCT03056755-1 | Alpelisib | PI3K | Breast cancer |
| NCT03056755 | NCT03056755-1 | Fulvestrant | Endocrine Therapy | Breast cancer |
| NCT03056755 | NCT03056755-2 | Alpelisib | PI3K | Breast cancer |
| NCT03056755 | NCT03056755-2 | Letrozole | Endocrine Therapy | Breast cancer |
| NCT03056833 | NCT03056833-0 | Platinum-Based Chemotherapy | chemotherapy | Other solid tumors |
| NCT03056833 | NCT03056833-0 | Paclitaxel | chemotherapy | Other solid tumors |
| NCT03056833 | NCT03056833-0 | Ribociclib | CDK4/CDK6 | Other solid tumors |
| NCT03057106 | NCT03057106-1 | Durvalumab | PD-L1 | lung cancers |
| NCT03057106 | NCT03057106-1 | Tremelimumab | CTLA-4 | lung cancers |
| NCT03057106 | NCT03057106-2 | Platinum-Based Chemotherapy | chemotherapy | lung cancers |
| NCT03057106 | NCT03057106-2 | Durvalumab | PD-L1 | lung cancers |
| NCT03057106 | NCT03057106-2 | Tremelimumab | CTLA-4 | lung cancers |
| NCT03065387 | NCT03065387-1 | Neratinib | EGFR | Other solid tumors |
| NCT03065387 | NCT03065387-1 | Everolimus | mTOR | Other solid tumors |
| NCT03065387 | NCT03065387-1 | Palbociclib | CDK4/CDK6 | Other solid tumors |
| NCT03065387 | NCT03065387-2 | Neratinib | EGFR | Other solid tumors |
| NCT03065387 | NCT03065387-2 | Everolimus | mTOR | Other solid tumors |
| NCT03065387 | NCT03065387-2 | Trametinib | MEK | Other solid tumors |
| NCT03070301 | NCT03070301-0 | Ribociclib | CDK4/CDK6 | Brain and nervous system cancers |
| NCT03070301 | NCT03070301-0 | Everolimus | mTOR | Brain and nervous system cancers |
| NCT03078751 | NCT03078751-0 | Ribociclib | CDK4/CDK6 | Breast cancer |
| NCT03078751 | NCT03078751-0 | Adjuvant Endocrine Therapy | Endocrine Therapy | Breast cancer |
| NCT03080116 | NCT03080116-0 | Apalutamide | Endocrine Therapy | Prostate cancer |
| NCT03080116 | NCT03080116-0 | Degarelix | Endocrine Therapy | Prostate cancer |
| NCT03084471 | NCT03084471-0 | Tremelimumab | CTLA-4 | Other solid tumors |
| NCT03084471 | NCT03084471-0 | Durvalumab | PD-L1 | Other solid tumors |
| NCT03085095 | NCT03085095-0 | Relugolix | Endocrine Therapy | Prostate cancer |
| NCT03085095 | NCT03085095-0 | E2/Neta | Endocrine Therapy | Prostate cancer |
| NCT03085225 | NCT03085225-0 | Durvalumab | PD-L1 | Other solid tumors |
| NCT03085225 | NCT03085225-0 | Trabectedin | chemotherapy | Other solid tumors |
| NCT03085849 | NCT03085849-0 | Guadecitabine | chemotherapy | lung cancers |
| NCT03085849 | NCT03085849-0 | Durvalumab | PD-L1 | lung cancers |
| NCT03085849 | NCT03085849-0 | Tremelimumab | CTLA-4 | lung cancers |
| NCT03089645 | NCT03089645-0 | Medi5083 | CD40 | Other solid tumors |
| NCT03089645 | NCT03089645-0 | Durvalumab | PD-L1 | Other solid tumors |
| NCT03089645 | NCT03089645-0 | Tremelimumab | CTLA-4 | Other solid tumors |
| NCT03089645 | NCT03089645-0 | Docetaxel | chemotherapy | Other solid tumors |
| NCT03090165 | NCT03090165-0 | Ribociclib | CDK4/CDK6 | Breast cancer |
| NCT03090165 | NCT03090165-0 | Bicalutamide | Endocrine Therapy | Breast cancer |
| NCT03092674 | NCT03092674-0 | Azacitidine | chemotherapy | Leukaemia |
| NCT03092674 | NCT03092674-0 | Midostaurin | PKC/PDGFR | Leukaemia |
| NCT03094052 | NCT03094052-0 | Neratinib | EGFR | Breast cancer |
| NCT03094052 | NCT03094052-0 | Trastuzumab | HER2 | Breast cancer |
| NCT03095274 | NCT03095274-0 | Tremelimumab | CTLA-4 | Brain and nervous system cancers |
| NCT03095274 | NCT03095274-0 | Durvalumab | PD-L1 | Brain and nervous system cancers |
| NCT03095612 | NCT03095612-0 | Selinexor | XPO1 | lung cancers |
| NCT03095612 | NCT03095612-0 | Docetaxel | chemotherapy | lung cancers |
| NCT03096847 | NCT03096847-0 | Ribociclib | CDK4/CDK6 | Breast cancer |
| NCT03096847 | NCT03096847-0 | Letrozole | Endocrine Therapy | Breast cancer |
| NCT03098836 | NCT03098836-0 | Apalutamide | Endocrine Therapy | Prostate cancer |
| NCT03098836 | NCT03098836-0 | Abiraterone | CYP17A1 | Prostate cancer |
| NCT03098836 | NCT03098836-0 | Prednisone | Steroid Treatment | Prostate cancer |
| NCT03099174 | NCT03099174-1 | Xentuzumab | IGF | Breast cancer |
| NCT03099174 | NCT03099174-1 | Abemaciclib | CDK4/CDK6 | Breast cancer |
| NCT03099174 | NCT03099174-2 | Xentuzumab | IGF | Breast cancer |
| NCT03099174 | NCT03099174-2 | Abemaciclib | CDK4/CDK6 | Breast cancer |
| NCT03099174 | NCT03099174-2 | Letrozole | Endocrine Therapy | Breast cancer |
| NCT03099174 | NCT03099174-3 | Xentuzumab | IGF | Breast cancer |
| NCT03099174 | NCT03099174-3 | Abemaciclib | CDK4/CDK6 | Breast cancer |
| NCT03099174 | NCT03099174-3 | Anastrozole | Endocrine Therapy | Breast cancer |
| NCT03099174 | NCT03099174-4 | Xentuzumab | IGF | Breast cancer |
| NCT03099174 | NCT03099174-4 | Abemaciclib | CDK4/CDK6 | Breast cancer |
| NCT03099174 | NCT03099174-4 | Fulvestrant | Endocrine Therapy | Breast cancer |
| NCT03101475 | NCT03101475-0 | Tremelimumab | CTLA-4 | Other solid tumors |
| NCT03101475 | NCT03101475-0 | Durvalumab | PD-L1 | Other solid tumors |
| NCT03101748 | NCT03101748-0 | Neratinib | EGFR | Breast cancer |
| NCT03101748 | NCT03101748-0 | Pertuzumab | HER2 | Breast cancer |
| NCT03101748 | NCT03101748-0 | Trastuzumab | HER2 | Breast cancer |
| NCT03103087 | NCT03103087-0 | Relugolix | Endocrine Therapy | Other solid tumors |
| NCT03103087 | NCT03103087-0 | E2/Neta | Endocrine Therapy | Other solid tumors |
| NCT03104842 | NCT03104842-0 | Isatuximab | CD38 | Multiple Myeloma |
| NCT03104842 | NCT03104842-0 | Carfilzomib | PSMB | Multiple Myeloma |
| NCT03104842 | NCT03104842-0 | Lenalidomide | CRBN | Multiple Myeloma |
| NCT03104842 | NCT03104842-0 | Dexamethasone | Steroid Treatment | Multiple Myeloma |
| NCT03106415 | NCT03106415-0 | Binimetinib | MEK | Breast cancer |
| NCT03106415 | NCT03106415-0 | Pembrolizumab | PD-1 | Breast cancer |
| NCT03107988 | NCT03107988-0 | Lorlatinib | ALK | Brain and nervous system cancers |
| NCT03107988 | NCT03107988-0 | Chemotherapy | chemotherapy | Brain and nervous system cancers |
| NCT03110562 | NCT03110562-0 | Selinexor | XPO1 | Multiple Myeloma |
| NCT03110562 | NCT03110562-0 | Bortezomib | PSMB | Multiple Myeloma |
| NCT03110562 | NCT03110562-0 | Dexamethasone | Steroid Treatment | Multiple Myeloma |
| NCT03113643 | NCT03113643-1 | Tagraxofusp-Erzs | CD123 | Leukaemia |
| NCT03113643 | NCT03113643-1 | Azacitidine | chemotherapy | Leukaemia |
| NCT03113643 | NCT03113643-1 | Venetoclax | BCL2 | Leukaemia |
| NCT03113643 | NCT03113643-2 | Tagraxofusp-Erzs | CD123 | Leukaemia |
| NCT03113643 | NCT03113643-2 | Azacitidine | chemotherapy | Leukaemia |
| NCT03114527 | NCT03114527-0 | Ribociclib | CDK4/CDK6 | Other solid tumors |
| NCT03114527 | NCT03114527-0 | Everolimus | mTOR | Other solid tumors |
| NCT03116529 | NCT03116529-0 | Tremelimumab | CTLA-4 | Other solid tumors |
| NCT03116529 | NCT03116529-0 | Durvalumab | PD-L1 | Other solid tumors |
| NCT03122496 | NCT03122496-0 | Tremelimumab | CTLA-4 | Other solid tumors |
| NCT03122496 | NCT03122496-0 | Durvalumab | PD-L1 | Other solid tumors |
| NCT03122509 | NCT03122509-0 | Tremelimumab | CTLA-4 | Colon and rectum cancers |
| NCT03122509 | NCT03122509-0 | Durvalumab | PD-L1 | Colon and rectum cancers |
| NCT03128619 | NCT03128619-1 | Copanlisib | PI3K | Breast cancer |
| NCT03128619 | NCT03128619-1 | Letrozole | Endocrine Therapy | Breast cancer |
| NCT03128619 | NCT03128619-1 | Palbociclib | CDK4/CDK6 | Breast cancer |
| NCT03128619 | NCT03128619-2 | Copanlisib | PI3K | Breast cancer |
| NCT03128619 | NCT03128619-2 | Letrozole | Endocrine Therapy | Breast cancer |
| NCT03128619 | NCT03128619-3 | Palbociclib | CDK4/CDK6 | Breast cancer |
| NCT03128619 | NCT03128619-3 | Letrozole | Endocrine Therapy | Breast cancer |
| NCT03132467 | NCT03132467-0 | Tremelimumab | CTLA-4 | Breast cancer |
| NCT03132467 | NCT03132467-0 | Durvalumab | PD-L1 | Breast cancer |
| NCT03136627 | NCT03136627-0 | Tivozanib | VEGFR/PDGFR | Kidney cancer |
| NCT03136627 | NCT03136627-0 | Nivolumab | PD-1 | Kidney cancer |
| NCT03141671 | NCT03141671-0 | Gnrh Agonist | Endocrine Therapy | Prostate cancer |
| NCT03141671 | NCT03141671-0 | Abiraterone | CYP17A1 | Prostate cancer |
| NCT03141671 | NCT03141671-0 | Apalutamide | Endocrine Therapy | Prostate cancer |
| NCT03141671 | NCT03141671-0 | Prednisone | Steroid Treatment | Prostate cancer |
| NCT03144778 | NCT03144778-0 | Tremelimumab | CTLA-4 | Other solid tumors |
| NCT03144778 | NCT03144778-0 | Durvalumab | PD-L1 | Other solid tumors |
| NCT03147287 | NCT03147287-0 | Fulvestrant | Endocrine Therapy | Breast cancer |
| NCT03147287 | NCT03147287-0 | Palbociclib | CDK4/CDK6 | Breast cancer |
| NCT03147287 | NCT03147287-0 | Avelumab | PD-L1 | Breast cancer |
| NCT03147885 | NCT03147885-0 | Chemotherapy | chemotherapy | Lymphomas |
| NCT03147885 | NCT03147885-0 | Rituximab | CD20 | Lymphomas |
| NCT03147885 | NCT03147885-0 | Prednisone | Steroid Treatment | Lymphomas |
| NCT03147885 | NCT03147885-0 | Selinexor | XPO1 | Lymphomas |
| NCT03150693 | NCT03150693-0 | Chemotherapy | chemotherapy | Other hematologic Neoplasms |
| NCT03150693 | NCT03150693-0 | Inotuzumab Ozogamicin | CD22/DNA | Other hematologic Neoplasms |
| NCT03151811 | NCT03151811-1 | Melphalan | chemotherapy | Multiple Myeloma |
| NCT03151811 | NCT03151811-1 | Dexamethasone | Steroid Treatment | Multiple Myeloma |
| NCT03151811 | NCT03151811-2 | Pomalidomide | CRBN | Multiple Myeloma |
| NCT03151811 | NCT03151811-2 | Dexamethasone | Steroid Treatment | Multiple Myeloma |
| NCT03152565 | NCT03152565-0 | Avelumab | PD-L1 | Colon and rectum cancers |
| NCT03152565 | NCT03152565-0 | Autologous Dendritic Cell Vaccine | vaccine | Colon and rectum cancers |
| NCT03154281 | NCT03154281-0 | Niraparib | PARP | Breast cancer |
| NCT03154281 | NCT03154281-0 | Everolimus | mTOR | Breast cancer |
| NCT03155997 | NCT03155997-0 | Abemaciclib | CDK4/CDK6 | Breast cancer |
| NCT03155997 | NCT03155997-0 | Endocrine Therapy | Endocrine Therapy | Breast cancer |
| NCT03158064 | NCT03158064-0 | Tremelimumab | CTLA-4 | Other solid tumors |
| NCT03158064 | NCT03158064-0 | Durvalumab | PD-L1 | Other solid tumors |
| NCT03158103 | NCT03158103-0 | Binimetinib | MEK | Other solid tumors |
| NCT03158103 | NCT03158103-0 | Pexidartinib | CSF1R/PDGFR | Other solid tumors |
| NCT03161223 | NCT03161223-1 | Azacitidine | chemotherapy | Lymphomas |
| NCT03161223 | NCT03161223-1 | Durvalumab | PD-L1 | Lymphomas |
| NCT03161223 | NCT03161223-1 | Romidepsin | chemotherapy | Lymphomas |
| NCT03161223 | NCT03161223-2 | Durvalumab | PD-L1 | Lymphomas |
| NCT03161223 | NCT03161223-2 | Pralatrexate | chemotherapy | Lymphomas |
| NCT03161223 | NCT03161223-2 | Romidepsin | chemotherapy | Lymphomas |
| NCT03161223 | NCT03161223-3 | Durvalumab | PD-L1 | Lymphomas |
| NCT03161223 | NCT03161223-3 | Romidepsin | chemotherapy | Lymphomas |
| NCT03161223 | NCT03161223-4 | Durvalumab | PD-L1 | Lymphomas |
| NCT03161223 | NCT03161223-4 | Azacitidine | chemotherapy | Lymphomas |
| NCT03162224 | NCT03162224-0 | Medi0457 | vaccine | Head and Neck Neoplasms |
| NCT03162224 | NCT03162224-0 | Durvalumab | PD-L1 | Head and Neck Neoplasms |
| NCT03162627 | NCT03162627-0 | Selumetinib | MEK | Bladder cancer |
| NCT03162627 | NCT03162627-0 | Olaparib | PARP | Bladder cancer |
| NCT03164616 | NCT03164616-1 | Durvalumab | PD-L1 | lung cancers |
| NCT03164616 | NCT03164616-1 | Tremelimumab | CTLA-4 | lung cancers |
| NCT03164616 | NCT03164616-1 | Chemotherapy | chemotherapy | lung cancers |
| NCT03164616 | NCT03164616-2 | Durvalumab | PD-L1 | lung cancers |
| NCT03164616 | NCT03164616-2 | Chemotherapy | chemotherapy | lung cancers |
| NCT03164772 | NCT03164772-0 | Bi1361849 | vaccine | lung cancers |
| NCT03164772 | NCT03164772-0 | Tremelimumab | CTLA-4 | lung cancers |
| NCT03164772 | NCT03164772-0 | Durvalumab | PD-L1 | lung cancers |
| NCT03167619 | NCT03167619-0 | Olaparib | PARP | Breast cancer |
| NCT03167619 | NCT03167619-0 | Durvalumab | PD-L1 | Breast cancer |
| NCT03170206 | NCT03170206-0 | Binimetinib | MEK | lung cancers |
| NCT03170206 | NCT03170206-0 | Palbociclib | CDK4/CDK6 | lung cancers |
| NCT03173248 | NCT03173248-0 | Ivosidenib | IDH | Leukaemia |
| NCT03173248 | NCT03173248-0 | Azacitidine | chemotherapy | Leukaemia |
| NCT03174275 | NCT03174275-0 | Carboplatin | chemotherapy | Other solid tumors |
| NCT03174275 | NCT03174275-0 | Paclitaxel | chemotherapy | Other solid tumors |
| NCT03174275 | NCT03174275-0 | Durvalumab | PD-L1 | Other solid tumors |
| NCT03174405 | NCT03174405-0 | Avelumab | PD-L1 | Colon and rectum cancers |
| NCT03174405 | NCT03174405-0 | Cetuximab | EGFR | Colon and rectum cancers |
| NCT03174405 | NCT03174405-0 | Folfox | chemotherapy | Colon and rectum cancers |
| NCT03178552 | NCT03178552-1 | Atezolizumab | PD-L1 | lung cancers |
| NCT03178552 | NCT03178552-1 | Vemurafenib | RAF | lung cancers |
| NCT03178552 | NCT03178552-1 | Cobimetinib | MEK | lung cancers |
| NCT03178552 | NCT03178552-2 | Atezolizumab | PD-L1 | lung cancers |
| NCT03178552 | NCT03178552-2 | Bevacizumab | VEGF | lung cancers |
| NCT03178552 | NCT03178552-2 | Platinum-Based Chemotherapy | chemotherapy | lung cancers |
| NCT03178552 | NCT03178552-2 | Pemetrexed | chemotherapy | lung cancers |
| NCT03182634 | NCT03182634-1 | Fulvestrant | Endocrine Therapy | Breast cancer |
| NCT03182634 | NCT03182634-1 | Neratinib | EGFR | Breast cancer |
| NCT03182634 | NCT03182634-2 | Fulvestrant | Endocrine Therapy | Breast cancer |
| NCT03182634 | NCT03182634-2 | Azd5363 | AKT | Breast cancer |
| NCT03182634 | NCT03182634-3 | Olaparib | PARP | Breast cancer |
| NCT03182634 | NCT03182634-3 | Azd6738 | ATR | Breast cancer |
| NCT03189706 | NCT03189706-0 | Naxitamab | GD-2 | Brain and nervous system cancers |
| NCT03189706 | NCT03189706-0 | Irinotecan | chemotherapy | Brain and nervous system cancers |
| NCT03189706 | NCT03189706-0 | Temozolomide | chemotherapy | Brain and nervous system cancers |
| NCT03189706 | NCT03189706-0 | Gm-Csf | CSF2 | Brain and nervous system cancers |
| NCT03192345 | NCT03192345-0 | Sar439459 | TGFβ | Other solid tumors |
| NCT03192345 | NCT03192345-0 | Cemiplimab-Rwlc | PD-1 | Other solid tumors |
| NCT03194867 | NCT03194867-0 | Isatuximab | CD38 | Multiple Myeloma |
| NCT03194867 | NCT03194867-0 | Cemiplimab-Rwlc | PD-1 | Multiple Myeloma |
| NCT03198650 | NCT03198650-0 | Acalabrutinib | BTK | Lymphomas |
| NCT03198650 | NCT03198650-0 | Obinutuzumab | CD20 | Lymphomas |
| NCT03199040 | NCT03199040-0 | Neoantigen Dna Vaccine | vaccine | Breast cancer |
| NCT03199040 | NCT03199040-0 | Durvalumab | PD-L1 | Breast cancer |
| NCT03200587 | NCT03200587-0 | Avelumab | PD-L1 | Kidney cancer |
| NCT03200587 | NCT03200587-0 | Cabozantinib | VEGFR/MET/RET | Kidney cancer |
| NCT03202758 | NCT03202758-0 | Durvalumab | PD-L1 | Colon and rectum cancers |
| NCT03202758 | NCT03202758-0 | Tremelimumab | CTLA-4 | Colon and rectum cancers |
| NCT03202758 | NCT03202758-0 | Folfox | chemotherapy | Colon and rectum cancers |
| NCT03204812 | NCT03204812-0 | Tremelimumab | CTLA-4 | Prostate cancer |
| NCT03204812 | NCT03204812-0 | Durvalumab | PD-L1 | Prostate cancer |
| NCT03206073 | NCT03206073-1 | Pexastimogene Devacirepvec | oncolytic virus | Colon and rectum cancers |
| NCT03206073 | NCT03206073-1 | Durvalumab | PD-L1 | Colon and rectum cancers |
| NCT03206073 | NCT03206073-2 | Pexastimogene Devacirepvec | oncolytic virus | Colon and rectum cancers |
| NCT03206073 | NCT03206073-2 | Durvalumab | PD-L1 | Colon and rectum cancers |
| NCT03206073 | NCT03206073-2 | Tremelimumab | CTLA-4 | Colon and rectum cancers |
| NCT03207256 | NCT03207256-0 | Umbralisib | PI3K | Other hematologic Neoplasms |
| NCT03207256 | NCT03207256-0 | Ublituximab | CD20 | Other hematologic Neoplasms |
| NCT03207529 | NCT03207529-0 | Alpelisib | PI3K | Other solid tumors |
| NCT03207529 | NCT03207529-0 | Enzalutamide | Endocrine Therapy | Other solid tumors |
| NCT03209401 | NCT03209401-0 | Niraparib | PARP | Other solid tumors |
| NCT03209401 | NCT03209401-0 | Platinum-Based Chemotherapy | chemotherapy | Other solid tumors |
| NCT03212469 | NCT03212469-0 | Tremelimumab | CTLA-4 | Other solid tumors |
| NCT03212469 | NCT03212469-0 | Durvalumab | PD-L1 | Other solid tumors |
| NCT03212937 | NCT03212937-0 | Selinexor | XPO1 | Lymphomas |
| NCT03212937 | NCT03212937-0 | Chemotherapy | chemotherapy | Lymphomas |
| NCT03217747 | NCT03217747-1 | Utomilumab | 4-1BB | Prostate cancer |
| NCT03217747 | NCT03217747-1 | Avelumab | PD-L1 | Prostate cancer |
| NCT03217747 | NCT03217747-2 | Pf-04518600 | OX40 | Prostate cancer |
| NCT03217747 | NCT03217747-2 | Avelumab | PD-L1 | Prostate cancer |
| NCT03217747 | NCT03217747-3 | Pf-04518600 | OX40 | Prostate cancer |
| NCT03217747 | NCT03217747-3 | Utomilumab | 4-1BB | Prostate cancer |
| NCT03217747 | NCT03217747-3 | Avelumab | PD-L1 | Prostate cancer |
| NCT03221400 | NCT03221400-0 | Pen-866 | HSP90 | Other solid tumors |
| NCT03221400 | NCT03221400-0 | Niraparib | PARP | Other solid tumors |
| NCT03226418 | NCT03226418-1 | Venetoclax | BCL2 | Leukaemia |
| NCT03226418 | NCT03226418-1 | Azacitidine | chemotherapy | Leukaemia |
| NCT03226418 | NCT03226418-2 | Venetoclax | BCL2 | Leukaemia |
| NCT03226418 | NCT03226418-2 | Decitabine | chemotherapy | Leukaemia |
| NCT03228667 | NCT03228667-1 | N-803 | IL15 | Other solid tumors |
| NCT03228667 | NCT03228667-1 | Avelumab | PD-L1 | Other solid tumors |
| NCT03228667 | NCT03228667-2 | N-803 | IL15 | Other solid tumors |
| NCT03228667 | NCT03228667-2 | Durvalumab | PD-L1 | Other solid tumors |
| NCT03228667 | NCT03228667-3 | N-803 | IL15 | Other solid tumors |
| NCT03228667 | NCT03228667-3 | Avelumab | PD-L1 | Other solid tumors |
| NCT03228667 | NCT03228667-3 | Pd-L1 T-Hank | cell therapy | Other solid tumors |
| NCT03228667 | NCT03228667-4 | N-803 | IL15 | Other solid tumors |
| NCT03228667 | NCT03228667-4 | Durvalumab | PD-L1 | Other solid tumors |
| NCT03228667 | NCT03228667-4 | Pd-L1 T-Hank | cell therapy | Other solid tumors |
| NCT03228667 | NCT03244176-0 | Chemotherapy | chemotherapy | Other solid tumors |
| NCT03228667 | NCT03244176-0 | Rituximab | CD20 | Other solid tumors |
| NCT03233139 | NCT03233139-1 | Cemiplimab-Rwlc | PD-1 | Others |
| NCT03233139 | NCT03233139-1 | Ipilimumab | CTLA-4 | Others |
| NCT03233139 | NCT03233139-1 | Platinum-Based Chemotherapy | chemotherapy | Others |
| NCT03233139 | NCT03233139-2 | Cemiplimab-Rwlc | PD-1 | Others |
| NCT03233139 | NCT03233139-2 | Platinum-Based Chemotherapy | chemotherapy | Others |
| NCT03235245 | NCT03235245-0 | Binimetinib | MEK | Melanoma |
| NCT03235245 | NCT03235245-0 | Encorafenib | RAF | Melanoma |
| NCT03237377 | NCT03237377-0 | Tremelimumab | CTLA-4 | lung cancers |
| NCT03237377 | NCT03237377-0 | Durvalumab | PD-L1 | lung cancers |
| NCT03237390 | NCT03237390-0 | Ribociclib | CDK4/CDK6 | Other solid tumors |
| NCT03237390 | NCT03237390-0 | Gemcitabine | chemotherapy | Other solid tumors |
| NCT03238027 | NCT03238027-0 | Sndx-6352 | CSF1R | Other solid tumors |
| NCT03238027 | NCT03238027-0 | Durvalumab | PD-L1 | Other solid tumors |
| NCT03238196 | NCT03238196-0 | Fulvestrant | Endocrine Therapy | Breast cancer |
| NCT03238196 | NCT03238196-0 | Palbociclib | CDK4/CDK6 | Breast cancer |
| NCT03238196 | NCT03238196-0 | Erdafitinib | FGFR | Breast cancer |
| NCT03244176 | NCT03244176-0 | Avelumab | PD-L1 | Lymphomas |
| NCT03244176 | NCT03244176-0 | Prednisone | Steroid Treatment | Lymphomas |
| NCT03248427 | NCT03248427-0 | Ribociclib | CDK4/CDK6 | Breast cancer |
| NCT03248427 | NCT03248427-0 | Letrozole | Endocrine Therapy | Breast cancer |
| NCT03249142 | NCT03249142-0 | Tremelimumab | CTLA-4 | Ovary cancer |
| NCT03249142 | NCT03249142-0 | Durvalumab | PD-L1 | Ovary cancer |
| NCT03249870 | NCT03249870-0 | Inotuzumab Ozogamicin | CD22/DNA | Leukaemia |
| NCT03249870 | NCT03249870-0 | Chemotherapy | chemotherapy | Leukaemia |
| NCT03250832 | NCT03250832-1 | Tsr-033 | LAG-3 | Other solid tumors |
| NCT03250832 | NCT03250832-1 | Dostarlimab-Gxly | PD-1 | Other solid tumors |
| NCT03250832 | NCT03250832-2 | Tsr-033 | LAG-3 | Other solid tumors |
| NCT03250832 | NCT03250832-2 | Dostarlimab-Gxly | PD-1 | Other solid tumors |
| NCT03250832 | NCT03250832-2 | Mfolfox6 | chemotherapy | Other solid tumors |
| NCT03250832 | NCT03250832-2 | Bevacizumab | VEGF | Other solid tumors |
| NCT03250832 | NCT03250832-3 | Tsr-033 | LAG-3 | Other solid tumors |
| NCT03250832 | NCT03250832-3 | Dostarlimab-Gxly | PD-1 | Other solid tumors |
| NCT03250832 | NCT03250832-3 | Folfiri | chemotherapy | Other solid tumors |
| NCT03250832 | NCT03250832-3 | Bevacizumab | VEGF | Other solid tumors |
| NCT03252938 | NCT03252938-0 | Avelumab | PD-L1 | Other solid tumors |
| NCT03252938 | NCT03252938-0 | Imp321 | LAG-3 | Other solid tumors |
| NCT03257761 | NCT03257761-0 | Guadecitabine | chemotherapy | Other solid tumors |
| NCT03257761 | NCT03257761-0 | Durvalumab | PD-L1 | Other solid tumors |
| NCT03258398 | NCT03258398-0 | Tomivosertib | MNK | Colon and rectum cancers |
| NCT03258398 | NCT03258398-0 | Avelumab | PD-L1 | Colon and rectum cancers |
| NCT03258593 | NCT03258593-0 | Durvalumab | PD-L1 | Bladder cancer |
| NCT03258593 | NCT03258593-0 | Vicinium | EPCAM | Bladder cancer |
| NCT03258931 | NCT03258931-0 | Midostaurin | PKC/PDGFR | Leukaemia |
| NCT03258931 | NCT03258931-0 | Chemotherapy | chemotherapy | Leukaemia |
| NCT03260023 | NCT03260023-0 | Tipapkinogene Sovacivec | vaccine | Cervix uteri cancer |
| NCT03260023 | NCT03260023-0 | Avelumab | PD-L1 | Cervix uteri cancer |
| NCT03267589 | NCT03267589-1 | Oleclumab | CD73 | Ovary cancer |
| NCT03267589 | NCT03267589-1 | Durvalumab | PD-L1 | Ovary cancer |
| NCT03267589 | NCT03267589-2 | Tavolixizumab | OX40 | Ovary cancer |
| NCT03267589 | NCT03267589-2 | Durvalumab | PD-L1 | Ovary cancer |
| NCT03268057 | NCT03268057-0 | Pepinemab | SEMA4D | lung cancers |
| NCT03268057 | NCT03268057-0 | Avelumab | PD-L1 | lung cancers |
| NCT03270176 | NCT03270176-0 | Xevinapant | IAP | lung cancers |
| NCT03270176 | NCT03270176-0 | Avelumab | PD-L1 | lung cancers |
| NCT03271047 | NCT03271047-1 | Binimetinib | MEK | Colon and rectum cancers |
| NCT03271047 | NCT03271047-1 | Nivolumab | PD-1 | Colon and rectum cancers |
| NCT03271047 | NCT03271047-2 | Binimetinib | MEK | Colon and rectum cancers |
| NCT03271047 | NCT03271047-2 | Nivolumab | PD-1 | Colon and rectum cancers |
| NCT03271047 | NCT03271047-2 | Ipilimumab | CTLA-4 | Colon and rectum cancers |
| NCT03274492 | NCT03274492-0 | Polatuzumab Vedotin | CD79B/Tubulin | Lymphomas |
| NCT03274492 | NCT03274492-0 | Rituximab | CD20 | Lymphomas |
| NCT03274492 | NCT03274492-0 | Chp | chemotherapy | Lymphomas |
| NCT03275285 | NCT03275285-0 | Isatuximab | CD38 | Multiple Myeloma |
| NCT03275285 | NCT03275285-0 | Carfilzomib | PSMB | Multiple Myeloma |
| NCT03275285 | NCT03275285-0 | Dexamethasone | Steroid Treatment | Multiple Myeloma |
| NCT03275597 | NCT03275597-0 | Tremelimumab | CTLA-4 | lung cancers |
| NCT03275597 | NCT03275597-0 | Durvalumab | PD-L1 | lung cancers |
| NCT03277482 | NCT03277482-0 | Tremelimumab | CTLA-4 | Other solid tumors |
| NCT03277482 | NCT03277482-0 | Durvalumab | PD-L1 | Other solid tumors |
| NCT03279250 | NCT03279250-1 | Lhrha | Endocrine Therapy | Prostate cancer |
| NCT03279250 | NCT03279250-1 | Apalutamide | Endocrine Therapy | Prostate cancer |
| NCT03279250 | NCT03279250-2 | Lhrha | Endocrine Therapy | Prostate cancer |
| NCT03279250 | NCT03279250-2 | Apalutamide | Endocrine Therapy | Prostate cancer |
| NCT03279250 | NCT03279250-2 | Abiraterone | CYP17A1 | Prostate cancer |
| NCT03280030 | NCT03280030-1 | Midostaurin | PKC/PDGFR | Leukaemia |
| NCT03280030 | NCT03280030-1 | Daunorubicin | chemotherapy | Leukaemia |
| NCT03280030 | NCT03280030-2 | Midostaurin | PKC/PDGFR | Leukaemia |
| NCT03280030 | NCT03280030-2 | Cytarabine | chemotherapy | Leukaemia |
| NCT03280563 | NCT03280563-1 | Atezolizumab | PD-L1 | Breast cancer |
| NCT03280563 | NCT03280563-1 | Entinostat | chemotherapy | Breast cancer |
| NCT03280563 | NCT03280563-2 | Atezolizumab | PD-L1 | Breast cancer |
| NCT03280563 | NCT03280563-2 | Fulvestrant | Endocrine Therapy | Breast cancer |
| NCT03280563 | NCT03280563-3 | Atezolizumab | PD-L1 | Breast cancer |
| NCT03280563 | NCT03280563-3 | Ipatasertib | AKT | Breast cancer |
| NCT03280563 | NCT03280563-4 | Atezolizumab | PD-L1 | Breast cancer |
| NCT03280563 | NCT03280563-4 | Ipatasertib | AKT | Breast cancer |
| NCT03280563 | NCT03280563-4 | Fulvestrant | Endocrine Therapy | Breast cancer |
| NCT03280563 | NCT03280563-5 | Atezolizumab | PD-L1 | Breast cancer |
| NCT03280563 | NCT03280563-5 | Bevacizumab | VEGF | Breast cancer |
| NCT03280563 | NCT03280563-5 | Endocrine Therapy | Endocrine Therapy | Breast cancer |
| NCT03280563 | NCT03280563-6 | Atezolizumab | PD-L1 | Breast cancer |
| NCT03280563 | NCT03280563-6 | Abemaciclib | CDK4/CDK6 | Breast cancer |
| NCT03280563 | NCT03280563-6 | Fulvestrant | Endocrine Therapy | Breast cancer |
| NCT03283137 | NCT03283137-0 | Pembrolizumab | PD-1 | Lymphomas |
| NCT03283137 | NCT03283137-0 | Umbralisib | PI3K | Lymphomas |
| NCT03283384 | NCT03283384-0 | Ribociclib | CDK4/CDK6 | Breast cancer |
| NCT03283384 | NCT03283384-0 | Letrozole | Endocrine Therapy | Breast cancer |
| NCT03283605 | NCT03283605-0 | Tremelimumab | CTLA-4 | Head and Neck Neoplasms |
| NCT03283605 | NCT03283605-0 | Durvalumab | PD-L1 | Head and Neck Neoplasms |
| NCT03284957 | NCT03284957-1 | Amcenestrant | Endocrine Therapy | Breast cancer |
| NCT03284957 | NCT03284957-1 | Palbociclib | CDK4/CDK6 | Breast cancer |
| NCT03284957 | NCT03284957-2 | Amcenestrant | Endocrine Therapy | Breast cancer |
| NCT03284957 | NCT03284957-2 | Alpelisib | PI3K | Breast cancer |
| NCT03284957 | NCT03284957-3 | Amcenestrant | Endocrine Therapy | Breast cancer |
| NCT03284957 | NCT03284957-3 | Everolimus | mTOR | Breast cancer |
| NCT03284957 | NCT03284957-4 | Amcenestrant | Endocrine Therapy | Breast cancer |
| NCT03284957 | NCT03284957-4 | Abemaciclib | CDK4/CDK6 | Breast cancer |
| NCT03285412 | NCT03285412-0 | Ribociclib | CDK4/CDK6 | Breast cancer |
| NCT03285412 | NCT03285412-0 | Endocrine Therapy | Endocrine Therapy | Breast cancer |
| NCT03288350 | NCT03288350-0 | Docetaxel | chemotherapy | Other solid tumors |
| NCT03288350 | NCT03288350-0 | Avelumab | PD-L1 | Other solid tumors |
| NCT03288532 | NCT03288532-0 | Tremelimumab | CTLA-4 | Kidney cancer |
| NCT03288532 | NCT03288532-0 | Durvalumab | PD-L1 | Kidney cancer |
| NCT03289039 | NCT03289039-0 | Neratinib | EGFR | Breast cancer |
| NCT03289039 | NCT03289039-0 | Fulvestrant | Endocrine Therapy | Breast cancer |
| NCT03289533 | NCT03289533-0 | Avelumab | PD-L1 | Liver cancer |
| NCT03289533 | NCT03289533-0 | Axitinib | VEGFR/PDGFR | Liver cancer |
| NCT03291314 | NCT03291314-0 | Axitinib | VEGFR/PDGFR | Brain and nervous system cancers |
| NCT03291314 | NCT03291314-0 | Avelumab | PD-L1 | Brain and nervous system cancers |
| NCT03292250 | NCT03292250-0 | Durvalumab | PD-L1 | Head and Neck Neoplasms |
| NCT03292250 | NCT03292250-0 | Tremelimumab | CTLA-4 | Head and Neck Neoplasms |
| NCT03294083 | NCT03294083-0 | Pexastimogene Devacirepvec | oncolytic virus | Kidney cancer |
| NCT03294083 | NCT03294083-0 | Cemiplimab-Rwlc | PD-1 | Kidney cancer |
| NCT03294694 | NCT03294694-1 | Ribociclib | CDK4/CDK6 | Other solid tumors |
| NCT03294694 | NCT03294694-1 | Spartalizumab | PD-1 | Other solid tumors |
| NCT03294694 | NCT03294694-2 | Ribociclib | CDK4/CDK6 | Other solid tumors |
| NCT03294694 | NCT03294694-2 | Spartalizumab | PD-1 | Other solid tumors |
| NCT03294694 | NCT03294694-2 | Fulvestrant | Endocrine Therapy | Other solid tumors |
| NCT03298087 | NCT03298087-0 | Lhrha | Endocrine Therapy | Prostate cancer |
| NCT03298087 | NCT03298087-0 | Apalutamide | Endocrine Therapy | Prostate cancer |
| NCT03298087 | NCT03298087-0 | Abiraterone | CYP17A1 | Prostate cancer |
| NCT03298451 | NCT03298451-0 | Tremelimumab | CTLA-4 | Liver cancer |
| NCT03298451 | NCT03298451-0 | Durvalumab | PD-L1 | Liver cancer |
| NCT03299660 | NCT03299660-0 | Avelumab | PD-L1 | Colon and rectum cancers |
| NCT03299660 | NCT03299660-0 | 5-Fu | chemotherapy | Colon and rectum cancers |
| NCT03299660 | NCT03299660-0 | Capecitabine | chemotherapy | Colon and rectum cancers |
| NCT03307785 | NCT03307785-1 | Dostarlimab-Gxly | PD-1 | lung cancers |
| NCT03307785 | NCT03307785-1 | Niraparib | PARP | lung cancers |
| NCT03307785 | NCT03307785-2 | Dostarlimab-Gxly | PD-1 | lung cancers |
| NCT03307785 | NCT03307785-2 | Niraparib | PARP | lung cancers |
| NCT03307785 | NCT03307785-2 | Bevacizumab | VEGF | lung cancers |
| NCT03308396 | NCT03308396-0 | Durvalumab | PD-L1 | Other solid tumors |
| NCT03308396 | NCT03308396-0 | Guadecitabine | chemotherapy | Other solid tumors |
| NCT03308942 | NCT03308942-1 | Niraparib | PARP | Others |
| NCT03308942 | NCT03308942-1 | Pembrolizumab | PD-1 | Others |
| NCT03308942 | NCT03308942-2 | Niraparib | PARP | Others |
| NCT03308942 | NCT03308942-2 | Dostarlimab-Gxly | PD-1 | Others |
| NCT03309878 | NCT03309878-0 | Pembrolizumab | PD-1 | Lymphomas |
| NCT03309878 | NCT03309878-0 | Mogamulizumab | CCR4 | Lymphomas |
| NCT03310619 | NCT03310619-0 | Lisocabtagene Maraleucel | cell therapy | Lymphomas |
| NCT03310619 | NCT03310619-0 | Durvalumab | PD-L1 | Lymphomas |
| NCT03311555 | NCT03311555-0 | Apalutamide | Endocrine Therapy | Prostate cancer |
| NCT03311555 | NCT03311555-0 | Docetaxel | chemotherapy | Prostate cancer |
| NCT03317158 | NCT03317158-0 | Durvalumab | PD-L1 | Bladder cancer |
| NCT03317158 | NCT03317158-0 | Bacille Calmette-Guérin | vaccine | Bladder cancer |
| NCT03317457 | NCT03317457-0 | Tremelimumab | CTLA-4 | Other solid tumors |
| NCT03317457 | NCT03317457-0 | Durvalumab | PD-L1 | Other solid tumors |
| NCT03317496 | NCT03317496-1 | Avelumab | PD-L1 | lung cancers |
| NCT03317496 | NCT03317496-1 | Pemetrexed | chemotherapy | lung cancers |
| NCT03317496 | NCT03317496-2 | Avelumab | PD-L1 | lung cancers |
| NCT03317496 | NCT03317496-2 | Platinum-Based Chemotherapy | chemotherapy | lung cancers |
| NCT03317496 | NCT03317496-3 | Avelumab | PD-L1 | lung cancers |
| NCT03317496 | NCT03317496-3 | Gemcitabine | chemotherapy | lung cancers |
| NCT03319316 | NCT03319316-0 | Tremelimumab | CTLA-4 | lung cancers |
| NCT03319316 | NCT03319316-0 | Durvalumab | PD-L1 | lung cancers |
| NCT03319667 | NCT03319667-0 | Isatuximab | CD38 | Multiple Myeloma |
| NCT03319667 | NCT03319667-0 | Bortezomib | PSMB | Multiple Myeloma |
| NCT03319667 | NCT03319667-0 | Lenalidomide | CRBN | Multiple Myeloma |
| NCT03319667 | NCT03319667-0 | Dexamethasone | Steroid Treatment | Multiple Myeloma |
| NCT03323398 | NCT03323398-0 | Mrna-2416 | vaccine | Others |
| NCT03323398 | NCT03323398-0 | Durvalumab | PD-L1 | Others |
| NCT03324282 | NCT03324282-0 | Gemcitabine | chemotherapy | Bladder cancer |
| NCT03324282 | NCT03324282-0 | Platinum-Based Chemotherapy | chemotherapy | Bladder cancer |
| NCT03324282 | NCT03324282-0 | Avelumab | PD-L1 | Bladder cancer |
| NCT03325816 | NCT03325816-0 | Nivolumab | PD-1 | lung cancers |
| NCT03325816 | NCT03325816-0 | Lutetium Lu 177 Dotatate | SSTR | lung cancers |
| NCT03326193 | NCT03326193-0 | Niraparib | PARP | Ovary cancer |
| NCT03326193 | NCT03326193-0 | Bevacizumab | VEGF | Ovary cancer |
| NCT03326310 | NCT03326310-0 | Azacitidine | chemotherapy | Leukaemia |
| NCT03326310 | NCT03326310-0 | Selumetinib | MEK | Leukaemia |
| NCT03328273 | NCT03328273-0 | Ceralasertib | ATR | Leukaemia |
| NCT03328273 | NCT03328273-0 | Acalabrutinib | BTK | Leukaemia |
| NCT03330405 | NCT03330405-0 | Avelumab | PD-L1 | Other solid tumors |
| NCT03330405 | NCT03330405-0 | Talazoparib | PARP | Other solid tumors |
| NCT03330990 | NCT03330990-0 | Entrectinib | TRK | Other solid tumors |
| NCT03330990 | NCT03330990-0 | Midazolam | Other supportive therapy | Other solid tumors |
| NCT03332017 | NCT03332017-0 | Obinutuzumab | CD20 | Lymphomas |
| NCT03332017 | NCT03332017-0 | Zanubrutinib | BTK | Lymphomas |
| NCT03333343 | NCT03333343-0 | Nazartinib | EGFR | lung cancers |
| NCT03333343 | NCT03333343-0 | Ribociclib | CDK4/CDK6 | lung cancers |
| NCT03334617 | NCT03334617-1 | Durvalumab | PD-L1 | lung cancers |
| NCT03334617 | NCT03334617-1 | Olaparib | PARP | lung cancers |
| NCT03334617 | NCT03334617-2 | Durvalumab | PD-L1 | lung cancers |
| NCT03334617 | NCT03334617-2 | Danvatirsen | STAT3 | lung cancers |
| NCT03334617 | NCT03334617-3 | Durvalumab | PD-L1 | lung cancers |
| NCT03334617 | NCT03334617-3 | Azd6738 | ATR | lung cancers |
| NCT03334617 | NCT03334617-4 | Durvalumab | PD-L1 | lung cancers |
| NCT03334617 | NCT03334617-4 | Vistusertib | mTOR | lung cancers |
| NCT03334617 | NCT03334617-5 | Durvalumab | PD-L1 | lung cancers |
| NCT03334617 | NCT03334617-5 | Oleclumab | CD73 | lung cancers |
| NCT03334617 | NCT03334617-6 | Durvalumab | PD-L1 | lung cancers |
| NCT03334617 | NCT03334617-6 | Trastuzumab Deruxtecan | HER2/TOP1 | lung cancers |
| NCT03334617 | NCT03334617-7 | Durvalumab | PD-L1 | lung cancers |
| NCT03334617 | NCT03334617-7 | Cediranib | KDR | lung cancers |
| NCT03336333 | NCT03336333-1 | Zanubrutinib | BTK | Leukaemia |
| NCT03336333 | NCT03336333-1 | Venetoclax | BCL2 | Leukaemia |
| NCT03336333 | NCT03336333-2 | Bendamustine | chemotherapy | Leukaemia |
| NCT03336333 | NCT03336333-2 | Rituximab | CD20 | Leukaemia |
| NCT03341845 | NCT03341845-0 | Axitinib | VEGFR/PDGFR | Kidney cancer |
| NCT03341845 | NCT03341845-0 | Avelumab | PD-L1 | Kidney cancer |
| NCT03345810 | NCT03345810-1 | Platinum-Based Chemotherapy | chemotherapy | lung cancers |
| NCT03345810 | NCT03345810-1 | Paclitaxel | chemotherapy | lung cancers |
| NCT03345810 | NCT03345810-1 | Durvalumab | PD-L1 | lung cancers |
| NCT03345810 | NCT03345810-2 | Gemcitabine | chemotherapy | lung cancers |
| NCT03345810 | NCT03345810-2 | Durvalumab | PD-L1 | lung cancers |
| NCT03345810 | NCT03345810-2 | Vinorelbine | chemotherapy | lung cancers |
| NCT03355794 | NCT03355794-0 | Ribociclib | CDK4/CDK6 | Brain and nervous system cancers |
| NCT03355794 | NCT03355794-0 | Everolimus | mTOR | Brain and nervous system cancers |
| NCT03356860 | NCT03356860-0 | Paclitaxel | chemotherapy | Breast cancer |
| NCT03356860 | NCT03356860-0 | Epirubicin | chemotherapy | Breast cancer |
| NCT03356860 | NCT03356860-0 | Cyclophosphamide | chemotherapy | Breast cancer |
| NCT03356860 | NCT03356860-0 | Durvalumab | PD-L1 | Breast cancer |
| NCT03357757 | NCT03357757-0 | Avelumab | PD-L1 | Others |
| NCT03357757 | NCT03357757-0 | Valproic Acid | chemotherapy | Others |
| NCT03363373 | NCT03336333-3 | Gm-Csf | CSF2 | Brain and nervous system cancers |
| NCT03363373 | NCT03336333-3 | Naxitamab | GD-2 | Brain and nervous system cancers |
| NCT03367819 | NCT03367819-0 | Cemiplimab-Rwlc | PD-1 | Other solid tumors |
| NCT03367819 | NCT03367819-0 | Isatuximab | CD38 | Other solid tumors |
| NCT03368729 | NCT03368729-0 | Niraparib | PARP | Breast cancer |
| NCT03368729 | NCT03368729-0 | Trastuzumab | HER2 | Breast cancer |
| NCT03371719 | NCT03371719-0 | Apalutamide | Endocrine Therapy | Prostate cancer |
| NCT03371719 | NCT03371719-0 | Androgen Deprivation Therapy (Adt) | Endocrine Therapy | Prostate cancer |
| NCT03373760 | NCT03373760-0 | Tremelimumab | CTLA-4 | lung cancers |
| NCT03373760 | NCT03373760-0 | Durvalumab | PD-L1 | lung cancers |
| NCT03374254 | NCT03374254-1 | Pembrolizumab | PD-1 | Colon and rectum cancers |
| NCT03374254 | NCT03374254-1 | Binimetinib | MEK | Colon and rectum cancers |
| NCT03374254 | NCT03374254-2 | Pembrolizumab | PD-1 | Colon and rectum cancers |
| NCT03374254 | NCT03374254-2 | Mfolfox7 | chemotherapy | Colon and rectum cancers |
| NCT03374254 | NCT03374254-3 | Pembrolizumab | PD-1 | Colon and rectum cancers |
| NCT03374254 | NCT03374254-3 | Mfolfox7 | chemotherapy | Colon and rectum cancers |
| NCT03374254 | NCT03374254-3 | Binimetinib | MEK | Colon and rectum cancers |
| NCT03374254 | NCT03374254-4 | Pembrolizumab | PD-1 | Colon and rectum cancers |
| NCT03374254 | NCT03374254-4 | Folfiri | chemotherapy | Colon and rectum cancers |
| NCT03374254 | NCT03374254-5 | Pembrolizumab | PD-1 | Colon and rectum cancers |
| NCT03374254 | NCT03374254-5 | Folfiri | chemotherapy | Colon and rectum cancers |
| NCT03374254 | NCT03374254-5 | Binimetinib | MEK | Colon and rectum cancers |
| NCT03376659 | NCT03376659-0 | Durvalumab | PD-L1 | Other solid tumors |
| NCT03376659 | NCT03376659-0 | Cv301 | vaccine | Other solid tumors |
| NCT03376659 | NCT03376659-0 | Chemotherapy | chemotherapy | Other solid tumors |
| NCT03377387 | NCT03377387-0 | Capecitabine | chemotherapy | Breast cancer |
| NCT03377387 | NCT03377387-0 | Neratinib | EGFR | Breast cancer |
| NCT03377400 | NCT03377400-0 | Tremelimumab | CTLA-4 | Other solid tumors |
| NCT03377400 | NCT03377400-0 | Durvalumab | PD-L1 | Other solid tumors |
| NCT03379051 | NCT03379051-0 | Ublituximab | CD20 | Leukaemia |
| NCT03379051 | NCT03379051-0 | Umbralisib | PI3K | Leukaemia |
| NCT03379051 | NCT03379051-0 | Venetoclax | BCL2 | Leukaemia |
| NCT03381183 | NCT03381183-0 | Durvalumab | PD-L1 | Other solid tumors |
| NCT03381183 | NCT03381183-0 | Irx-2Regimen | cell therapy | Other solid tumors |
| NCT03383575 | NCT03383575-0 | Azacitidine | chemotherapy | Leukaemia |
| NCT03383575 | NCT03383575-0 | Enasidenib | IDH | Leukaemia |
| NCT03385655 | NCT03385655-0 | Durvalumab | PD-L1 | Prostate cancer |
| NCT03385655 | NCT03385655-0 | Tremelimumab | CTLA-4 | Prostate cancer |
| NCT03386162 | NCT03386162-0 | Fulvestrant | Endocrine Therapy | Breast cancer |
| NCT03386162 | NCT03386162-0 | Alpelisib | PI3K | Breast cancer |
| NCT03386929 | NCT03386929-0 | Avelumab | PD-L1 | lung cancers |
| NCT03386929 | NCT03386929-0 | Axitinib | VEGFR/PDGFR | lung cancers |
| NCT03386929 | NCT03386929-0 | Palbociclib | CDK4/CDK6 | lung cancers |
| NCT03387020 | NCT03387020-0 | Ribociclib | CDK4/CDK6 | Brain and nervous system cancers |
| NCT03387020 | NCT03387020-0 | Everolimus | mTOR | Brain and nervous system cancers |
| NCT03390296 | NCT03390296-1 | Azacitidine | chemotherapy | Leukaemia |
| NCT03390296 | NCT03390296-1 | Venetoclax | BCL2 | Leukaemia |
| NCT03390296 | NCT03390296-1 | Gemtuzumab Ozogamicin | CD33/DNA | Leukaemia |
| NCT03390296 | NCT03390296-2 | Azacitidine | chemotherapy | Leukaemia |
| NCT03390296 | NCT03390296-2 | Avelumab | PD-L1 | Leukaemia |
| NCT03390296 | NCT03390296-2 | Gemtuzumab Ozogamicin | CD33/DNA | Leukaemia |
| NCT03390296 | NCT03390296-3 | Azacitidine | chemotherapy | Leukaemia |
| NCT03390296 | NCT03390296-3 | Venetoclax | BCL2 | Leukaemia |
| NCT03390296 | NCT03390296-3 | Avelumab | PD-L1 | Leukaemia |
| NCT03390296 | NCT03390296-4 | Azacitidine | chemotherapy | Leukaemia |
| NCT03390296 | NCT03390296-4 | Avelumab | PD-L1 | Leukaemia |
| NCT03390296 | NCT03390296-4 | Pf-04518600 | OX40 | Leukaemia |
| NCT03390296 | NCT03390296-5 | Glasdegib | SMO | Leukaemia |
| NCT03390296 | NCT03390296-5 | Gemtuzumab Ozogamicin | CD33/DNA | Leukaemia |
| NCT03390595 | NCT03390595-1 | Avelumab | PD-L1 | Bladder cancer |
| NCT03390595 | NCT03390595-1 | Gemcitabine | chemotherapy | Bladder cancer |
| NCT03390595 | NCT03390595-2 | Avelumab | PD-L1 | Bladder cancer |
| NCT03390595 | NCT03390595-2 | Platinum-Based Chemotherapy | chemotherapy | Bladder cancer |
| NCT03392246 | NCT03392246-0 | Osimertinib | EGFR | lung cancers |
| NCT03392246 | NCT03392246-0 | Selumetinib | MEK | lung cancers |
| NCT03394144 | NCT03394144-0 | Azd9150 | Oligonucleotide | Other solid tumors |
| NCT03394144 | NCT03394144-0 | Durvalumab | PD-L1 | Other solid tumors |
| NCT03395197 | NCT03395197-0 | Talazoparib | PARP | Prostate cancer |
| NCT03395197 | NCT03395197-0 | Enzalutamide | Endocrine Therapy | Prostate cancer |
| NCT03399071 | NCT03399071-0 | Flot | chemotherapy | Stomach cancer |
| NCT03399071 | NCT03399071-0 | Avelumab | PD-L1 | Stomach cancer |
| NCT03404960 | NCT03404960-1 | Niraparib | PARP | Pancreas cancer |
| NCT03404960 | NCT03404960-1 | Nivolumab | PD-1 | Pancreas cancer |
| NCT03404960 | NCT03404960-2 | Niraparib | PARP | Pancreas cancer |
| NCT03404960 | NCT03404960-2 | Ipilimumab | CTLA-4 | Pancreas cancer |
| NCT03406650 | NCT03406650-0 | Durvalumab | PD-L1 | Others |
| NCT03406650 | NCT03406650-0 | Chemotherapy | chemotherapy | Others |
| NCT03409458 | NCT03409458-0 | Pt-112 | chemotherapy | lung cancers |
| NCT03409458 | NCT03409458-0 | Avelumab | PD-L1 | lung cancers |
| NCT03409614 | NCT03409614-0 | Cemiplimab-Rwlc | PD-1 | lung cancers |
| NCT03409614 | NCT03409614-0 | Chemotherapy | chemotherapy | lung cancers |
| NCT03410615 | NCT03410615-0 | Tremelimumab | CTLA-4 | Mouth and oropharynx cancers |
| NCT03410615 | NCT03410615-0 | Durvalumab | PD-L1 | Mouth and oropharynx cancers |
| NCT03412890 | NCT03412890-0 | Relugolix | Endocrine Therapy | Other solid tumors |
| NCT03412890 | NCT03412890-0 | E2/Neta | Endocrine Therapy | Other solid tumors |
| NCT03416179 | NCT03416179-1 | Daunorubicin | chemotherapy | Leukaemia |
| NCT03416179 | NCT03416179-1 | Cytarabine | chemotherapy | Leukaemia |
| NCT03416179 | NCT03416179-1 | Glasdegib | SMO | Leukaemia |
| NCT03416179 | NCT03416179-2 | Glasdegib | SMO | Leukaemia |
| NCT03416179 | NCT03416179-2 | Azacitidine | chemotherapy | Leukaemia |
| NCT03420742 | NCT03420742-0 | Midazolam | Other supportive therapy | lung cancers |
| NCT03420742 | NCT03420742-0 | Brigatinib | ALK/EGFR | lung cancers |
| NCT03421353 | NCT03421353-1 | Danvatirsen | STAT3 | Other solid tumors |
| NCT03421353 | NCT03421353-1 | Durvalumab | PD-L1 | Other solid tumors |
| NCT03421353 | NCT03421353-2 | Danvatirsen | STAT3 | Other solid tumors |
| NCT03421353 | NCT03421353-2 | Durvalumab | PD-L1 | Other solid tumors |
| NCT03421353 | NCT03421353-2 | Gemcitabine | chemotherapy | Other solid tumors |
| NCT03421353 | NCT03421353-3 | Danvatirsen | STAT3 | Other solid tumors |
| NCT03421353 | NCT03421353-3 | Durvalumab | PD-L1 | Other solid tumors |
| NCT03421353 | NCT03421353-3 | Platinum-Based Chemotherapy | chemotherapy | Other solid tumors |
| NCT03421353 | NCT03421353-3 | Paclitaxel | chemotherapy | Other solid tumors |
| NCT03424005 | NCT03424005-1 | Atezolizumab | PD-L1 | Breast cancer |
| NCT03424005 | NCT03424005-1 | Paclitaxel | chemotherapy | Breast cancer |
| NCT03424005 | NCT03424005-2 | Atezolizumab | PD-L1 | Breast cancer |
| NCT03424005 | NCT03424005-2 | Paclitaxel | chemotherapy | Breast cancer |
| NCT03424005 | NCT03424005-2 | Tocilizumab | IL6 | Breast cancer |
| NCT03424005 | NCT03424005-3 | Atezolizumab | PD-L1 | Breast cancer |
| NCT03424005 | NCT03424005-3 | Sacituzumab Govitecan | Trop-2/TOP1 | Breast cancer |
| NCT03424005 | NCT03424005-4 | Atezolizumab | PD-L1 | Breast cancer |
| NCT03424005 | NCT03424005-4 | Ipatasertib | AKT | Breast cancer |
| NCT03424005 | NCT03424005-5 | Atezolizumab | PD-L1 | Breast cancer |
| NCT03424005 | NCT03424005-5 | Sgn-Liv1A | LIV-1 | Breast cancer |
| NCT03424005 | NCT03424005-6 | Atezolizumab | PD-L1 | Breast cancer |
| NCT03424005 | NCT03424005-6 | Selicrelumab | CD40 | Breast cancer |
| NCT03424005 | NCT03424005-6 | Bevacizumab | VEGF | Breast cancer |
| NCT03424005 | NCT03424005-7 | Atezolizumab | PD-L1 | Breast cancer |
| NCT03424005 | NCT03424005-7 | Chemotherapy | chemotherapy | Breast cancer |
| NCT03425201 | NCT03425201-0 | Niraparib | PARP | Others |
| NCT03425201 | NCT03425201-0 | Cabozantinib | VEGFR/MET/RET | Others |
| NCT03426657 | NCT03426657-0 | Durvalumab | PD-L1 | Head and Neck Neoplasms |
| NCT03426657 | NCT03426657-0 | Tremelimumab | CTLA-4 | Head and Neck Neoplasms |
| NCT03428126 | NCT03428126-0 | Durvalumab | PD-L1 | Colon and rectum cancers |
| NCT03428126 | NCT03428126-0 | Trametinib | MEK | Colon and rectum cancers |
| NCT03430063 | NCT03430063-0 | Cemiplimab-Rwlc | PD-1 | lung cancers |
| NCT03430063 | NCT03430063-0 | Ipilimumab | CTLA-4 | lung cancers |
| NCT03430518 | NCT03430518-0 | Durvalumab | PD-L1 | Breast cancer |
| NCT03430518 | NCT03430518-0 | Eribulin | chemotherapy | Breast cancer |
| NCT03430895 | NCT03430895-0 | Tremelimumab | CTLA-4 | Bladder cancer |
| NCT03430895 | NCT03430895-0 | Durvalumab | PD-L1 | Bladder cancer |
| NCT03431350 | NCT03431350-1 | Niraparib | PARP | Prostate cancer |
| NCT03431350 | NCT03431350-1 | Cetrelimab | PD-1 | Prostate cancer |
| NCT03431350 | NCT03431350-2 | Niraparib | PARP | Prostate cancer |
| NCT03431350 | NCT03431350-2 | Abiraterone | CYP17A1 | Prostate cancer |
| NCT03431350 | NCT03431350-2 | Prednisone | Steroid Treatment | Prostate cancer |
| NCT03433183 | NCT03433183-0 | Selumetinib | MEK | Other solid tumors |
| NCT03433183 | NCT03433183-0 | Sirolimus | mTOR | Other solid tumors |
| NCT03434262 | NCT03434262-1 | Ribociclib | CDK4/CDK6 | Other solid tumors |
| NCT03434262 | NCT03434262-1 | Gemcitabine | chemotherapy | Other solid tumors |
| NCT03434262 | NCT03434262-2 | Ribociclib | CDK4/CDK6 | Other solid tumors |
| NCT03434262 | NCT03434262-2 | Trametinib | MEK | Other solid tumors |
| NCT03434262 | NCT03434262-3 | Ribociclib | CDK4/CDK6 | Other solid tumors |
| NCT03434262 | NCT03434262-3 | Sonidegib | SMO | Other solid tumors |
| NCT03436654 | NCT03436654-0 | Apalutamide | Endocrine Therapy | Prostate cancer |
| NCT03436654 | NCT03436654-0 | Abiraterone | CYP17A1 | Prostate cancer |
| NCT03436654 | NCT03436654-0 | Prednisone | Steroid Treatment | Prostate cancer |
| NCT03439046 | NCT03439046-1 | Ribociclib | CDK4/CDK6 | Breast cancer |
| NCT03439046 | NCT03439046-1 | Letrozole | Endocrine Therapy | Breast cancer |
| NCT03439046 | NCT03439046-2 | Alpelisib | PI3K | Breast cancer |
| NCT03439046 | NCT03439046-2 | Fulvestrant | Endocrine Therapy | Breast cancer |
| NCT03439085 | NCT03439085-0 | Medi0457 | vaccine | Other solid tumors |
| NCT03439085 | NCT03439085-0 | Durvalumab | PD-L1 | Other solid tumors |
| NCT03440567 | NCT03440567-1 | Avelumab | PD-L1 | Lymphomas |
| NCT03440567 | NCT03440567-1 | Utomilumab | 4-1BB | Lymphomas |
| NCT03440567 | NCT03440567-1 | Rituximab | CD20 | Lymphomas |
| NCT03440567 | NCT03440567-1 | Chemotherapy | chemotherapy | Lymphomas |
| NCT03440567 | NCT03440567-2 | Avelumab | PD-L1 | Lymphomas |
| NCT03440567 | NCT03440567-2 | Utomilumab | 4-1BB | Lymphomas |
| NCT03440567 | NCT03440567-2 | Rituximab | CD20 | Lymphomas |
| NCT03440567 | NCT03440567-2 | Ibrutinib | BTK | Lymphomas |
| NCT03450967 | NCT03450967-0 | Tremelimumab | CTLA-4 | Head and Neck Neoplasms |
| NCT03450967 | NCT03450967-0 | Durvalumab | PD-L1 | Head and Neck Neoplasms |
| NCT03452332 | NCT03452332-0 | Tremelimumab | CTLA-4 | Other solid tumors |
| NCT03452332 | NCT03452332-0 | Durvalumab | PD-L1 | Other solid tumors |
| NCT03457896 | NCT03457896-1 | Trastuzumab | HER2 | Colon and rectum cancers |
| NCT03457896 | NCT03457896-1 | Neratinib | EGFR | Colon and rectum cancers |
| NCT03457896 | NCT03457896-2 | Cetuximab | EGFR | Colon and rectum cancers |
| NCT03457896 | NCT03457896-2 | Neratinib | EGFR | Colon and rectum cancers |
| NCT03459846 | NCT03459846-0 | Durvalumab | PD-L1 | Bladder cancer |
| NCT03459846 | NCT03459846-0 | Olaparib | PARP | Bladder cancer |
| NCT03462251 | NCT03462251-1 | Ribociclib | CDK4/CDK6 | Breast cancer |
| NCT03462251 | NCT03462251-1 | Aromatase Inhibitor | Endocrine Therapy | Breast cancer |
| NCT03462251 | NCT03462251-2 | Ribociclib | CDK4/CDK6 | Breast cancer |
| NCT03462251 | NCT03462251-2 | Fulvestrant | Endocrine Therapy | Breast cancer |
| NCT03465722 | NCT03465722-0 | Avapritinib | KIT | Other solid tumors |
| NCT03465722 | NCT03465722-0 | Regorafenib | VEGFR/PDGFR/RET/RAF | Other solid tumors |
| NCT03466450 | NCT03466450-0 | Glasdegib | SMO | Brain and nervous system cancers |
| NCT03466450 | NCT03466450-0 | Temozolomide | chemotherapy | Brain and nervous system cancers |
| NCT03471260 | NCT03471260-0 | Ivosidenib | IDH | Leukaemia |
| NCT03471260 | NCT03471260-0 | Venetoclax | BCL2 | Leukaemia |
| NCT03472274 | NCT03472274-0 | Tremelimumab | CTLA-4 | Bladder cancer |
| NCT03472274 | NCT03472274-0 | Durvalumab | PD-L1 | Bladder cancer |
| NCT03472560 | NCT03472560-0 | Avelumab | PD-L1 | lung cancers |
| NCT03472560 | NCT03472560-0 | Axitinib | VEGFR/PDGFR | lung cancers |
| NCT03473574 | NCT03473574-1 | Durvalumab | PD-L1 | Gallbladder and biliary tract cancer |
| NCT03473574 | NCT03473574-1 | Tremelimumab | CTLA-4 | Gallbladder and biliary tract cancer |
| NCT03473574 | NCT03473574-1 | Gemcitabine | chemotherapy | Gallbladder and biliary tract cancer |
| NCT03473574 | NCT03473574-2 | Durvalumab | PD-L1 | Gallbladder and biliary tract cancer |
| NCT03473574 | NCT03473574-2 | Tremelimumab | CTLA-4 | Gallbladder and biliary tract cancer |
| NCT03473574 | NCT03473574-2 | Gemcitabine | chemotherapy | Gallbladder and biliary tract cancer |
| NCT03473574 | NCT03473574-2 | Platinum-Based Chemotherapy | chemotherapy | Gallbladder and biliary tract cancer |
| NCT03473574 | NCT03473574-3 | Durvalumab | PD-L1 | Gallbladder and biliary tract cancer |
| NCT03473574 | NCT03473574-3 | Gemcitabine | chemotherapy | Gallbladder and biliary tract cancer |
| NCT03473574 | NCT03473574-3 | Platinum-Based Chemotherapy | chemotherapy | Gallbladder and biliary tract cancer |
| NCT03473743 | NCT03473743-0 | Erdafitinib | FGFR | Bladder cancer |
| NCT03473743 | NCT03473743-0 | Cetrelimab | PD-1 | Bladder cancer |
| NCT03474744 | NCT03474744-0 | Copanlisib | PI3K | Lymphomas |
| NCT03474744 | NCT03474744-0 | Rituximab | CD20 | Lymphomas |
| NCT03475004 | NCT03475004-0 | Pembrolizumab | PD-1 | Colon and rectum cancers |
| NCT03475004 | NCT03475004-0 | Binimetinib | MEK | Colon and rectum cancers |
| NCT03475004 | NCT03475004-0 | Bevacizumab | VEGF | Colon and rectum cancers |
| NCT03475953 | NCT03475953-0 | Regorafenib | VEGFR/PDGFR/RET/RAF | Other solid tumors |
| NCT03475953 | NCT03475953-0 | Avelumab | PD-L1 | Other solid tumors |
| NCT03477396 | NCT03477396-0 | Ribociclib | CDK4/CDK6 | Breast cancer |
| NCT03477396 | NCT03477396-0 | Aromatase Inhibitor | Endocrine Therapy | Breast cancer |
| NCT03482102 | NCT03482102-0 | Tremelimumab | CTLA-4 | Gallbladder and biliary tract cancer |
| NCT03482102 | NCT03482102-0 | Durvalumab | PD-L1 | Gallbladder and biliary tract cancer |
| NCT03483883 | NCT03483883-0 | Avelumab | PD-L1 | Kidney cancer |
| NCT03483883 | NCT03483883-0 | Gemcitabine | chemotherapy | Kidney cancer |
| NCT03484819 | NCT03484819-0 | Copanlisib | PI3K | Lymphomas |
| NCT03484819 | NCT03484819-0 | Nivolumab | PD-1 | Lymphomas |
| NCT03484923 | NCT03484923-1 | Capmatinib | MET | Melanoma |
| NCT03484923 | NCT03484923-1 | Spartalizumab | PD-1 | Melanoma |
| NCT03484923 | NCT03484923-2 | Ribociclib | CDK4/CDK6 | Melanoma |
| NCT03484923 | NCT03484923-2 | Spartalizumab | PD-1 | Melanoma |
| NCT03485209 | NCT03485209-0 | Tisotumab Vedotin | TF/Tubulin | Other solid tumors |
| NCT03485209 | NCT03485209-0 | Pembrolizumab | PD-1 | Other solid tumors |
| NCT03485209 | NCT03485209-0 | Platinum-Based Chemotherapy | chemotherapy | Other solid tumors |
| NCT03490292 | NCT03490292-0 | Platinum-Based Chemotherapy | chemotherapy | Stomach cancer |
| NCT03490292 | NCT03490292-0 | Paclitaxel | chemotherapy | Stomach cancer |
| NCT03490292 | NCT03490292-0 | Avelumab | PD-L1 | Stomach cancer |
| NCT03491683 | NCT03491683-0 | Ino-5401 | vaccine | Brain and nervous system cancers |
| NCT03491683 | NCT03491683-0 | Ino-9012 | vaccine | Brain and nervous system cancers |
| NCT03491683 | NCT03491683-0 | Cemiplimab-Rwlc | PD-1 | Brain and nervous system cancers |
| NCT03494322 | NCT03494322-0 | Avelumab | PD-L1 | Head and Neck Neoplasms |
| NCT03494322 | NCT03494322-0 | Cetuximab | EGFR | Head and Neck Neoplasms |
| NCT03498378 | NCT03498378-0 | Avelumab | PD-L1 | Head and Neck Neoplasms |
| NCT03498378 | NCT03498378-0 | Palbociclib | CDK4/CDK6 | Head and Neck Neoplasms |
| NCT03498378 | NCT03498378-0 | Cetuximab | EGFR | Head and Neck Neoplasms |
| NCT03501979 | NCT03501979-0 | Tucatinib | HER2 | Breast cancer |
| NCT03501979 | NCT03501979-0 | Trastuzumab | HER2 | Breast cancer |
| NCT03501979 | NCT03501979-0 | Capecitabine | chemotherapy | Breast cancer |
| NCT03502733 | NCT03502733-0 | Copanlisib | PI3K | Others |
| NCT03502733 | NCT03502733-0 | Nivolumab | PD-1 | Others |
| NCT03502733 | NCT03502733-0 | Ipilimumab | CTLA-4 | Others |
| NCT03503786 | NCT03503786-0 | Platinum-Based Chemotherapy | chemotherapy | Corpus uteri cancer |
| NCT03503786 | NCT03503786-0 | Paclitaxel | chemotherapy | Corpus uteri cancer |
| NCT03503786 | NCT03503786-0 | Avelumab | PD-L1 | Corpus uteri cancer |
| NCT03509012 | NCT03509012-1 | Durvalumab | PD-L1 | Other solid tumors |
| NCT03509012 | NCT03509012-1 | Platinum-Based Chemotherapy | chemotherapy | Other solid tumors |
| NCT03509012 | NCT03509012-2 | Durvalumab | PD-L1 | Other solid tumors |
| NCT03509012 | NCT03509012-2 | Platinum-Based Chemotherapy | chemotherapy | Other solid tumors |
| NCT03509012 | NCT03509012-2 | Paclitaxel | chemotherapy | Other solid tumors |
| NCT03512197 | NCT03512197-0 | Midostaurin | PKC/PDGFR | Leukaemia |
| NCT03512197 | NCT03512197-0 | Chemotherapy | chemotherapy | Leukaemia |
| NCT03512834 | NCT03512834-0 | Paclitaxel | chemotherapy | Other solid tumors |
| NCT03512834 | NCT03512834-0 | Avelumab | PD-L1 | Other solid tumors |
| NCT03515629 | NCT03515629-0 | Cemiplimab-Rwlc | PD-1 | lung cancers |
| NCT03515629 | NCT03515629-0 | Ipilimumab | CTLA-4 | lung cancers |
| NCT03515629 | NCT03515629-0 | Chemotherapy | chemotherapy | lung cancers |
| NCT03516617 | NCT03516617-0 | Acalabrutinib | BTK | Leukaemia |
| NCT03516617 | NCT03516617-0 | Obinutuzumab | CD20 | Leukaemia |
| NCT03517956 | NCT03517956-0 | Rogaratinib | FGFR | Other solid tumors |
| NCT03517956 | NCT03517956-0 | Copanlisib | PI3K | Other solid tumors |
| NCT03518606 | NCT03518606-0 | Durvalumab | PD-L1 | Others |
| NCT03518606 | NCT03518606-0 | Tremelimumab | CTLA-4 | Others |
| NCT03518606 | NCT03518606-0 | Vinorelbine | chemotherapy | Others |
| NCT03519971 | NCT03519971-0 | Durvalumab | PD-L1 | lung cancers |
| NCT03519971 | NCT03519971-0 | Platinum-Based Chemotherapy | chemotherapy | lung cancers |
| NCT03520920 | NCT03520920-0 | Zanubrutinib | BTK | Lymphomas |
| NCT03520920 | NCT03520920-0 | Rituximab | CD20 | Lymphomas |
| NCT03522584 | NCT03522584-0 | Tremelimumab | CTLA-4 | Head and Neck Neoplasms |
| NCT03522584 | NCT03522584-0 | Durvalumab | PD-L1 | Head and Neck Neoplasms |
| NCT03523572 | NCT03523572-0 | Trastuzumab Deruxtecan | HER2/TOP1 | Breast cancer |
| NCT03523572 | NCT03523572-0 | Nivolumab | PD-1 | Breast cancer |
| NCT03523585 | NCT03523585-0 | Trastuzumab | HER2 | Breast cancer |
| NCT03523585 | NCT03523585-0 | Capecitabine | chemotherapy | Breast cancer |
| NCT03527147 | NCT03527147-1 | Danvatirsen | STAT3 | Lymphomas |
| NCT03527147 | NCT03527147-1 | Acalabrutinib | BTK | Lymphomas |
| NCT03527147 | NCT03527147-2 | Azd6738 | ATR | Lymphomas |
| NCT03527147 | NCT03527147-2 | Acalabrutinib | BTK | Lymphomas |
| NCT03527147 | NCT03527147-3 | Magrolimab | CD47 | Lymphomas |
| NCT03527147 | NCT03527147-3 | Rituximab | CD20 | Lymphomas |
| NCT03527147 | NCT03527147-3 | Acalabrutinib | BTK | Lymphomas |
| NCT03527147 | NCT03527147-4 | Azd5153 | BRD | Lymphomas |
| NCT03527147 | NCT03527147-4 | Acalabrutinib | BTK | Lymphomas |
| NCT03528694 | NCT03528694-0 | Durvalumab | PD-L1 | Bladder cancer |
| NCT03528694 | NCT03528694-0 | Bacille Calmette-Guérin | vaccine | Bladder cancer |
| NCT03530397 | NCT03530397-1 | Medi5752 | PD1/CTLA-4 | Other solid tumors |
| NCT03530397 | NCT03530397-1 | Pemetrexed | chemotherapy | Other solid tumors |
| NCT03530397 | NCT03530397-1 | Carboplatin | chemotherapy | Other solid tumors |
| NCT03530397 | NCT03530397-2 | Pembrolizumab | PD-1 | Other solid tumors |
| NCT03530397 | NCT03530397-2 | Pemetrexed | chemotherapy | Other solid tumors |
| NCT03530397 | NCT03530397-2 | Carboplatin | chemotherapy | Other solid tumors |
| NCT03531645 | NCT03531645-0 | Fulvestrant | Endocrine Therapy | Other solid tumors |
| NCT03531645 | NCT03531645-0 | Abemaciclib | CDK4/CDK6 | Other solid tumors |
| NCT03532295 | NCT03532295-0 | Retifanlimab | PD-1 | Brain and nervous system cancers |
| NCT03532295 | NCT03532295-0 | Bevacizumab | VEGF | Brain and nervous system cancers |
| NCT03532295 | NCT03532295-0 | Avelumab | PD-L1 | Brain and nervous system cancers |
| NCT03533283 | NCT03533283-1 | Glofitamab | CD20/CD3 | Lymphomas |
| NCT03533283 | NCT03533283-1 | Atezolizumab | PD-L1 | Lymphomas |
| NCT03533283 | NCT03533283-2 | Polatuzumab Vedotin | CD79B/Tubulin | Lymphomas |
| NCT03533283 | NCT03533283-2 | Obinutuzumab | CD20 | Lymphomas |
| NCT03534323 | NCT03534323-0 | Duvelisib | PI3K | Leukaemia |
| NCT03534323 | NCT03534323-0 | Venetoclax | BCL2 | Leukaemia |
| NCT03534492 | NCT03534492-0 | Durvalumab | PD-L1 | Bladder cancer |
| NCT03534492 | NCT03534492-0 | Olaparib | PARP | Bladder cancer |
| NCT03536780 | NCT03536780-0 | Avelumab | PD-L1 | Other solid tumors |
| NCT03536780 | NCT03536780-0 | Gemcitabine | chemotherapy | Other solid tumors |
| NCT03539822 | NCT03539822-0 | Cabozantinib | VEGFR/MET/RET | Other solid tumors |
| NCT03539822 | NCT03539822-0 | Durvalumab | PD-L1 | Other solid tumors |
| NCT03544125 | NCT03544125-0 | Durvalumab | PD-L1 | Breast cancer |
| NCT03544125 | NCT03544125-0 | Olaparib | PARP | Breast cancer |
| NCT03544281 | NCT03544281-1 | Belantamab Mafodotin | BCMA/Tubulin | Multiple Myeloma |
| NCT03544281 | NCT03544281-1 | Lenalidomide | CRBN | Multiple Myeloma |
| NCT03544281 | NCT03544281-1 | Dexamethasone | Steroid Treatment | Multiple Myeloma |
| NCT03544281 | NCT03544281-2 | Belantamab Mafodotin | BCMA/Tubulin | Multiple Myeloma |
| NCT03544281 | NCT03544281-2 | Bortezomib | PSMB | Multiple Myeloma |
| NCT03544281 | NCT03544281-2 | Dexamethasone | Steroid Treatment | Multiple Myeloma |
| NCT03544723 | NCT03544723-0 | Ad-P53 | gene therapy | Others |
| NCT03544723 | NCT03544723-0 | Pd-1 | PD-1 | Others |
| NCT03547037 | NCT03547037-0 | Cetrelimab | PD-1 | Others |
| NCT03547037 | NCT03547037-0 | Erdafitinib | FGFR | Others |
| NCT03547973 | NCT03547973-1 | Sacituzumab Govitecan | Trop-2/TOP1 | Bladder cancer |
| NCT03547973 | NCT03547973-1 | Pembrolizumab | PD-1 | Bladder cancer |
| NCT03547973 | NCT03547973-2 | Sacituzumab Govitecan | Trop-2/TOP1 | Bladder cancer |
| NCT03547973 | NCT03547973-2 | Platinum-Based Chemotherapy | chemotherapy | Bladder cancer |
| NCT03547973 | NCT03547973-2 | Avelumab | PD-L1 | Bladder cancer |
| NCT03549715 | NCT03549715-0 | Tremelimumab | CTLA-4 | Bladder cancer |
| NCT03549715 | NCT03549715-0 | Durvalumab | PD-L1 | Bladder cancer |
| NCT03551782 | NCT03551782-0 | Cetrelimab | PD-1 | Prostate cancer |
| NCT03551782 | NCT03551782-0 | Apalutamide | Endocrine Therapy | Prostate cancer |
| NCT03555149 | NCT03555149-1 | Atezolizumab | PD-L1 | Colon and rectum cancers |
| NCT03555149 | NCT03555149-1 | Delolimogene Mupadenorepvec | oncolytic virus | Colon and rectum cancers |
| NCT03555149 | NCT03555149-2 | Atezolizumab | PD-L1 | Colon and rectum cancers |
| NCT03555149 | NCT03555149-2 | Regorafenib | VEGFR/PDGFR/RET/RAF | Colon and rectum cancers |
| NCT03555149 | NCT03555149-2 | Ab928 | A2aR/A2bR | Colon and rectum cancers |
| NCT03555149 | NCT03555149-3 | Atezolizumab | PD-L1 | Colon and rectum cancers |
| NCT03555149 | NCT03555149-3 | Regorafenib | VEGFR/PDGFR/RET/RAF | Colon and rectum cancers |
| NCT03555149 | NCT03555149-4 | Atezolizumab | PD-L1 | Colon and rectum cancers |
| NCT03555149 | NCT03555149-4 | Idasanutlin | MDM2/TP53 | Colon and rectum cancers |
| NCT03555149 | NCT03555149-5 | Atezolizumab | PD-L1 | Colon and rectum cancers |
| NCT03555149 | NCT03555149-5 | Selicrelumab | CD40 | Colon and rectum cancers |
| NCT03555149 | NCT03555149-5 | Bevacizumab | VEGF | Colon and rectum cancers |
| NCT03555149 | NCT03555149-6 | Atezolizumab | PD-L1 | Colon and rectum cancers |
| NCT03555149 | NCT03555149-6 | Isatuximab | CD38 | Colon and rectum cancers |
| NCT03555149 | NCT03555149-7 | Atezolizumab | PD-L1 | Colon and rectum cancers |
| NCT03555149 | NCT03555149-7 | Imprimepgg | Other supportive therapy | Colon and rectum cancers |
| NCT03555149 | NCT03555149-7 | Bevacizumab | VEGF | Colon and rectum cancers |
| NCT03555422 | NCT03555422-0 | Selinexor | XPO1 | Other solid tumors |
| NCT03555422 | NCT03555422-0 | Chemotherapy | chemotherapy | Other solid tumors |
| NCT03555877 | NCT03555877-0 | Anti-Hormonal Treatment | Endocrine Therapy | Breast cancer |
| NCT03555877 | NCT03555877-0 | Ribociclib | CDK4/CDK6 | Breast cancer |
| NCT03558139 | NCT03558139-0 | Magrolimab | CD47 | Ovary cancer |
| NCT03558139 | NCT03558139-0 | Avelumab | PD-L1 | Ovary cancer |
| NCT03563729 | NCT03563729-0 | Binimetinib | MEK | Melanoma |
| NCT03563729 | NCT03563729-0 | Encorafenib | RAF | Melanoma |
| NCT03564340 | NCT03564340-0 | Regn4018 | CD3/MUC16 | Ovary cancer |
| NCT03564340 | NCT03564340-0 | Cemiplimab-Rwlc | PD-1 | Ovary cancer |
| NCT03565991 | NCT03565991-0 | Avelumab | PD-L1 | Other solid tumors |
| NCT03565991 | NCT03565991-0 | Talazoparib | PARP | Other solid tumors |
| NCT03568097 | NCT03568097-0 | Avelumab | PD-L1 | lung cancers |
| NCT03568097 | NCT03568097-0 | Chemotherapy | chemotherapy | lung cancers |
| NCT03569280 | NCT03569280-0 | Enzalutamide | Endocrine Therapy | Prostate cancer |
| NCT03569280 | NCT03569280-0 | Apalutamide | Endocrine Therapy | Prostate cancer |
| NCT03571308 | NCT03571308-0 | Acalabrutinib | BTK | Lymphomas |
| NCT03571308 | NCT03571308-0 | Rituximab | CD20 | Lymphomas |
| NCT03571308 | NCT03571308-0 | Chemotherapy | chemotherapy | Lymphomas |
| NCT03572400 | NCT03572400-0 | Gemcitabine | chemotherapy | Other solid tumors |
| NCT03572400 | NCT03572400-0 | Durvalumab | PD-L1 | Other solid tumors |
| NCT03573648 | NCT03573648-0 | Avelumab | PD-L1 | Breast cancer |
| NCT03573648 | NCT03573648-0 | Palbociclib | CDK4/CDK6 | Breast cancer |
| NCT03573648 | NCT03573648-0 | Endocrine Therapy | Endocrine Therapy | Breast cancer |
| NCT03574779 | NCT03574779-1 | Tsr-042 | PD-1 | Ovary cancer |
| NCT03574779 | NCT03574779-1 | Bevacizumab | VEGF | Ovary cancer |
| NCT03574779 | NCT03574779-1 | Niraparib | PARP | Ovary cancer |
| NCT03574779 | NCT03574779-2 | Bevacizumab | VEGF | Ovary cancer |
| NCT03574779 | NCT03574779-2 | Niraparib | PARP | Ovary cancer |
| NCT03574779 | NCT03574779-3 | Bevacizumab | VEGF | Ovary cancer |
| NCT03574779 | NCT03574779-3 | Niraparib | PARP | Ovary cancer |
| NCT03574779 | NCT03574779-3 | Carboplatin | chemotherapy | Ovary cancer |
| NCT03574779 | NCT03574779-3 | Paclitaxel | chemotherapy | Ovary cancer |
| NCT03575013 | NCT03575013-0 | Avelumab | PD-L1 | Bladder cancer |
| NCT03575013 | NCT03575013-0 | Docetaxel | chemotherapy | Bladder cancer |
| NCT03578367 | NCT03578367-0 | Asciminib | BCR-ABL | Other hematologic Neoplasms |
| NCT03578367 | NCT03578367-0 | Imatinib | BCR-ABL | Other hematologic Neoplasms |
| NCT03579784 | NCT03579784-0 | Durvalumab | PD-L1 | Stomach cancer |
| NCT03579784 | NCT03579784-0 | Olaparib | PARP | Stomach cancer |
| NCT03579784 | NCT03579784-0 | Paclitaxel | chemotherapy | Stomach cancer |
| NCT03580928 | NCT03580928-0 | Acalabrutinib | BTK | Leukaemia |
| NCT03580928 | NCT03580928-0 | Venetoclax | BCL2 | Leukaemia |
| NCT03580928 | NCT03580928-0 | Obinutuzumab | CD20 | Leukaemia |
| NCT03581487 | NCT03581487-0 | Durvalumab | PD-L1 | lung cancers |
| NCT03581487 | NCT03581487-0 | Tremelimumab | CTLA-4 | lung cancers |
| NCT03581487 | NCT03581487-0 | Selumetinib | MEK | lung cancers |
| NCT03581942 | NCT03581942-0 | Copanlisib | PI3K | Lymphomas |
| NCT03581942 | NCT03581942-0 | Ibrutinib | BTK | Lymphomas |
| NCT03586661 | NCT03586661-0 | Niraparib | PARP | Other solid tumors |
| NCT03586661 | NCT03586661-0 | Copanlisib | PI3K | Other solid tumors |
| NCT03589222 | NCT03589222-0 | Selinexor | XPO1 | Multiple Myeloma |
| NCT03589222 | NCT03589222-0 | Bortezomib | PSMB | Multiple Myeloma |
| NCT03589222 | NCT03589222-0 | Dexamethasone | Steroid Treatment | Multiple Myeloma |
| NCT03589222 | NCT03589222-0 | Daratumumab | CD38 | Multiple Myeloma |
| NCT03591510 | NCT03591510-0 | Midostaurin | PKC/PDGFR | Leukaemia |
| NCT03591510 | NCT03591510-0 | Chemotherapy | chemotherapy | Leukaemia |
| NCT03594396 | NCT03594396-0 | Olaparib | PARP | Breast cancer |
| NCT03594396 | NCT03594396-0 | Durvalumab | PD-L1 | Breast cancer |
| NCT03595917 | NCT03595917-0 | Asciminib | BCR-ABL | Leukaemia |
| NCT03595917 | NCT03595917-0 | Dasatinib | BCR-ABL | Leukaemia |
| NCT03595917 | NCT03595917-0 | Prednisone | Steroid Treatment | Leukaemia |
| NCT03598270 | NCT03598270-0 | Chemotherapy | chemotherapy | Ovary cancer |
| NCT03598270 | NCT03598270-0 | Niraparib | PARP | Ovary cancer |
| NCT03598270 | NCT03598270-0 | Atezolizumab | PD-L1 | Ovary cancer |
| NCT03600883 | NCT03600883-1 | Sotorasib | KRAS | Other solid tumors |
| NCT03600883 | NCT03600883-1 | Pd-1 | PD-1 | Other solid tumors |
| NCT03600883 | NCT03600883-2 | Sotorasib | KRAS | Other solid tumors |
| NCT03600883 | NCT03600883-2 | Midazolam | Other supportive therapy | Other solid tumors |
| NCT03601455 | NCT03601455-0 | Durvalumab | PD-L1 | Bladder cancer |
| NCT03601455 | NCT03601455-0 | Tremelimumab | CTLA-4 | Bladder cancer |
| NCT03602859 | NCT03602859-0 | Platinum-Based Chemotherapy | chemotherapy | Ovary cancer |
| NCT03602859 | NCT03602859-0 | Paclitaxel | chemotherapy | Ovary cancer |
| NCT03602859 | NCT03602859-0 | Bevacizumab | VEGF | Ovary cancer |
| NCT03602859 | NCT03602859-0 | Niraparib | PARP | Ovary cancer |
| NCT03606967 | NCT03606967-0 | Neoantigen Vaccine | vaccine | Breast cancer |
| NCT03606967 | NCT03606967-0 | Durvalumab | PD-L1 | Breast cancer |
| NCT03606967 | NCT03606967-0 | Paclitaxel | chemotherapy | Breast cancer |
| NCT03608046 | NCT03608046-0 | Avelumab | PD-L1 | Colon and rectum cancers |
| NCT03608046 | NCT03608046-0 | Cetuximab | EGFR | Colon and rectum cancers |
| NCT03608046 | NCT03608046-0 | Irinotecan | chemotherapy | Colon and rectum cancers |
| NCT03608865 | NCT03608865-0 | Durvalumab | PD-L1 | Breast cancer |
| NCT03608865 | NCT03608865-0 | Tremelimumab | CTLA-4 | Breast cancer |
| NCT03611556 | NCT03611556-0 | Oleclumab | CD73 | Pancreas cancer |
| NCT03611556 | NCT03611556-0 | Durvalumab | PD-L1 | Pancreas cancer |
| NCT03611556 | NCT03611556-0 | Chemotherapy | chemotherapy | Pancreas cancer |
| NCT03613220 | NCT03613220-0 | Ribociclib | CDK4/CDK6 | Breast cancer |
| NCT03613220 | NCT03613220-0 | Letrozole | Endocrine Therapy | Breast cancer |
| NCT03616587 | NCT03616587-1 | Azd9833 | Endocrine Therapy | Breast cancer |
| NCT03616587 | NCT03616587-1 | Abemaciclib | CDK4/CDK6 | Breast cancer |
| NCT03616587 | NCT03616587-2 | Azd9833 | Endocrine Therapy | Breast cancer |
| NCT03616587 | NCT03616587-2 | Palbociclib | CDK4/CDK6 | Breast cancer |
| NCT03616886 | NCT03616886-0 | Paclitaxel | chemotherapy | Breast cancer |
| NCT03616886 | NCT03616886-0 | Platinum-Based Chemotherapy | chemotherapy | Breast cancer |
| NCT03616886 | NCT03616886-0 | Durvalumab | PD-L1 | Breast cancer |
| NCT03616886 | NCT03616886-0 | Oleclumab | CD73 | Breast cancer |
| NCT03617731 | NCT03617731-0 | Lenalidomide | CRBN | Multiple Myeloma |
| NCT03617731 | NCT03617731-0 | Isatuximab | CD38 | Multiple Myeloma |
| NCT03617913 | NCT03617913-0 | Avelumab | PD-L1 | Other solid tumors |
| NCT03617913 | NCT03617913-0 | Platinum-Based Chemotherapy | chemotherapy | Other solid tumors |
| NCT03617913 | NCT03617913-0 | 5-Fu | chemotherapy | Other solid tumors |
| NCT03617913 | NCT03617913-0 | Mitomycin | chemotherapy | Other solid tumors |
| NCT03618134 | NCT03618134-0 | Durvalumab | PD-L1 | Mouth and oropharynx cancers |
| NCT03618134 | NCT03618134-0 | Tremelimumab | CTLA-4 | Mouth and oropharynx cancers |
| NCT03618654 | NCT03618654-0 | Durvalumab | PD-L1 | Other solid tumors |
| NCT03618654 | NCT03618654-0 | Metformin | Antihyperglycemic | Other solid tumors |
| NCT03623373 | NCT03623373-1 | Acalabrutinib | BTK | Lymphomas |
| NCT03623373 | NCT03623373-1 | Bendamustine | chemotherapy | Lymphomas |
| NCT03623373 | NCT03623373-1 | Rituximab | CD20 | Lymphomas |
| NCT03623373 | NCT03623373-2 | Acalabrutinib | BTK | Lymphomas |
| NCT03623373 | NCT03623373-2 | Cytarabine | chemotherapy | Lymphomas |
| NCT03623373 | NCT03623373-2 | Rituximab | CD20 | Lymphomas |
| NCT03624231 | NCT03624231-0 | Durvalumab | PD-L1 | Head and Neck Neoplasms |
| NCT03624231 | NCT03624231-0 | Tremelimumab | CTLA-4 | Head and Neck Neoplasms |
| NCT03625505 | NCT03625505-0 | Gilteritinib | FLT3 | Leukaemia |
| NCT03625505 | NCT03625505-0 | Venetoclax | BCL2 | Leukaemia |
| NCT03631953 | NCT03631953-0 | Alpelisib | PI3K | Brain and nervous system cancers |
| NCT03631953 | NCT03631953-0 | Trametinib | MEK | Brain and nervous system cancers |
| NCT03634540 | NCT03634540-0 | Belzutifan | HIF-2 | Kidney cancer |
| NCT03634540 | NCT03634540-0 | Cabozantinib | VEGFR/MET/RET | Kidney cancer |
| NCT03636503 | NCT03636503-1 | Rituximab | CD20 | Lymphomas |
| NCT03636503 | NCT03636503-1 | Utomilumab | 4-1BB | Lymphomas |
| NCT03636503 | NCT03636503-1 | Avelumab | PD-L1 | Lymphomas |
| NCT03636503 | NCT03636503-2 | Rituximab | CD20 | Lymphomas |
| NCT03636503 | NCT03636503-2 | Avelumab | PD-L1 | Lymphomas |
| NCT03636503 | NCT03636503-2 | Pf-04518600 | OX40 | Lymphomas |
| NCT03637764 | NCT03637764-0 | Atezolizumab | PD-L1 | Others |
| NCT03637764 | NCT03637764-0 | Isatuximab | CD38 | Others |
| NCT03638141 | NCT03638141-0 | Durvalumab | PD-L1 | Liver cancer |
| NCT03638141 | NCT03638141-0 | Tremelimumab | CTLA-4 | Liver cancer |
| NCT03642132 | NCT03642132-1 | Chemotherapy | chemotherapy | Ovary cancer |
| NCT03642132 | NCT03642132-1 | Talazoparib | PARP | Ovary cancer |
| NCT03642132 | NCT03642132-2 | Chemotherapy | chemotherapy | Ovary cancer |
| NCT03642132 | NCT03642132-2 | Avelumab | PD-L1 | Ovary cancer |
| NCT03642132 | NCT03642132-2 | Talazoparib | PARP | Ovary cancer |
| NCT03643510 | NCT03643510-0 | Abemaciclib | CDK4/CDK6 | Corpus uteri cancer |
| NCT03643510 | NCT03643510-0 | Fulvestrant | Endocrine Therapy | Corpus uteri cancer |
| NCT03647488 | NCT03647488-0 | Capmatinib | MET | lung cancers |
| NCT03647488 | NCT03647488-0 | Spartalizumab | PD-1 | lung cancers |
| NCT03651206 | NCT03651206-0 | Niraparib | PARP | Other solid tumors |
| NCT03651206 | NCT03651206-0 | Dostarlimab-Gxly | PD-1 | Other solid tumors |
| NCT03654833 | NCT03654833-1 | Pembrolizumab | PD-1 | Other solid tumors |
| NCT03654833 | NCT03654833-1 | Bemcentinib | AXL | Other solid tumors |
| NCT03654833 | NCT03654833-2 | Atezolizumab | PD-L1 | Other solid tumors |
| NCT03654833 | NCT03654833-2 | Bevacizumab | VEGF | Other solid tumors |
| NCT03654833 | NCT03654833-3 | Dostarlimab-Gxly | PD-1 | Other solid tumors |
| NCT03654833 | NCT03654833-3 | Niraparib | PARP | Other solid tumors |
| NCT03655444 | NCT03655444-0 | Abemaciclib | CDK4/CDK6 | Head and Neck Neoplasms |
| NCT03655444 | NCT03655444-0 | Nivolumab | PD-1 | Head and Neck Neoplasms |
| NCT03656536 | NCT03656536-1 | Pemigatinib | FGFR | Other solid tumors |
| NCT03656536 | NCT03656536-1 | Cisplatin | chemotherapy | Other solid tumors |
| NCT03661515 | NCT03661515-0 | Fludarabine | chemotherapy | Leukaemia |
| NCT03661515 | NCT03661515-0 | Idarubicin | Chemotherapy | Leukaemia |
| NCT03661515 | NCT03661515-0 | Cytarabine | chemotherapy | Leukaemia |
| NCT03661515 | NCT03661515-0 | Selinexor | XPO1 | Leukaemia |
| NCT03665129 | NCT03665129-0 | Iph5401 | C5aR | Other solid tumors |
| NCT03665129 | NCT03665129-0 | Durvalumab | PD-L1 | Other solid tumors |
| NCT03669718 | NCT03669718-0 | Isa101B | vaccine | Others |
| NCT03669718 | NCT03669718-0 | Cemiplimab-Rwlc | PD-1 | Others |
| NCT03671018 | NCT03671018-0 | Mosunetuzumab | CD20/CD3 | Lymphomas |
| NCT03671018 | NCT03671018-0 | Polatuzumab Vedotin | CD79B/Tubulin | Lymphomas |
| NCT03671330 | NCT03671330-1 | Ribociclib | CDK4/CDK6 | Breast cancer |
| NCT03671330 | NCT03671330-1 | Letrozole | Endocrine Therapy | Breast cancer |
| NCT03671330 | NCT03671330-2 | Ribociclib | CDK4/CDK6 | Breast cancer |
| NCT03671330 | NCT03671330-2 | Anastrozole | Endocrine Therapy | Breast cancer |
| NCT03671590 | NCT03671590-0 | Tg-1701 | BTK | Other hematologic Neoplasms |
| NCT03671590 | NCT03671590-0 | Ublituximab | CD20 | Other hematologic Neoplasms |
| NCT03671590 | NCT03671590-0 | Umbralisib | PI3K | Other hematologic Neoplasms |
| NCT03672773 | NCT03672773-0 | Temozolomide | chemotherapy | lung cancers |
| NCT03672773 | NCT03672773-0 | Talazoparib | PARP | lung cancers |
| NCT03673124 | NCT03673124-0 | Ribociclib | CDK4/CDK6 | Ovary cancer |
| NCT03673124 | NCT03673124-0 | Letrozole | Endocrine Therapy | Ovary cancer |
| NCT03674424 | NCT03674424-1 | Dd-Mvac | chemotherapy | Bladder cancer |
| NCT03674424 | NCT03674424-1 | Avelumab | PD-L1 | Bladder cancer |
| NCT03674424 | NCT03674424-2 | Platinum-Based Chemotherapy | chemotherapy | Bladder cancer |
| NCT03674424 | NCT03674424-2 | Gemcitabine | chemotherapy | Bladder cancer |
| NCT03674424 | NCT03674424-2 | Avelumab | PD-L1 | Bladder cancer |
| NCT03674424 | NCT03674424-3 | Paclitaxel | chemotherapy | Bladder cancer |
| NCT03674424 | NCT03674424-3 | Gemcitabine | chemotherapy | Bladder cancer |
| NCT03674424 | NCT03674424-3 | Avelumab | PD-L1 | Bladder cancer |
| NCT03675893 | NCT03675893-0 | Abemaciclib | CDK4/CDK6 | Corpus uteri cancer |
| NCT03675893 | NCT03675893-0 | Letrozole | Endocrine Therapy | Corpus uteri cancer |
| NCT03677141 | NCT03677141-0 | Mosunetuzumab | CD20/CD3 | Lymphomas |
| NCT03677141 | NCT03677141-0 | Chp | chemotherapy | Lymphomas |
| NCT03677141 | NCT03677141-0 | Polatuzumab Vedotin | CD79B/Tubulin | Lymphomas |
| NCT03677154 | NCT03677154-0 | Mosunetuzumab | CD20/CD3 | Lymphomas |
| NCT03677154 | NCT03677154-0 | Polatuzumab Vedotin | CD79B/Tubulin | Lymphomas |
| NCT03680508 | NCT03680508-0 | Tsr-022 | TIM3 | Liver cancer |
| NCT03680508 | NCT03680508-0 | Tsr-042 | PD-1 | Liver cancer |
| NCT03682068 | NCT03682068-1 | Durvalumab | PD-L1 | Bladder cancer |
| NCT03682068 | NCT03682068-1 | Chemotherapy | chemotherapy | Bladder cancer |
| NCT03682068 | NCT03682068-2 | Durvalumab | PD-L1 | Bladder cancer |
| NCT03682068 | NCT03682068-2 | Tremelimumab | CTLA-4 | Bladder cancer |
| NCT03682068 | NCT03682068-2 | Chemotherapy | chemotherapy | Bladder cancer |
| NCT03683433 | NCT03683433-0 | Azacitidine | chemotherapy | Leukaemia |
| NCT03683433 | NCT03683433-0 | Enasidenib | IDH | Leukaemia |
| NCT03684694 | NCT03684694-0 | Loncastuximab Tesirine | CD19/DNA | Lymphomas |
| NCT03684694 | NCT03684694-0 | Ibrutinib | BTK | Lymphomas |
| NCT03684785 | NCT03684785-0 | Cavrotolimod | Oligonucleotide | Other solid tumors |
| NCT03684785 | NCT03684785-0 | Cemiplimab-Rwlc | PD-1 | Other solid tumors |
| NCT03691714 | NCT03691714-0 | Durvalumab | PD-L1 | Head and Neck Neoplasms |
| NCT03691714 | NCT03691714-0 | Cetuximab | EGFR | Head and Neck Neoplasms |
| NCT03693170 | NCT03693170-0 | Encorafenib | RAF | Colon and rectum cancers |
| NCT03693170 | NCT03693170-0 | Binimetinib | MEK | Colon and rectum cancers |
| NCT03693170 | NCT03693170-0 | Cetuximab | EGFR | Colon and rectum cancers |
| NCT03694236 | NCT03656536-2 | Chemotherapy | chemotherapy | lung cancers |
| NCT03694236 | NCT03656536-2 | Durvalumab | PD-L1 | lung cancers |
| NCT03695380 | NCT03695380-1 | Cobimetinib | MEK | Ovary cancer |
| NCT03695380 | NCT03695380-1 | Niraparib | PARP | Ovary cancer |
| NCT03695380 | NCT03695380-2 | Cobimetinib | MEK | Ovary cancer |
| NCT03695380 | NCT03695380-2 | Niraparib | PARP | Ovary cancer |
| NCT03695380 | NCT03695380-2 | Atezolizumab | PD-L1 | Ovary cancer |
| NCT03699449 | NCT03699449-1 | Durvalumab | PD-L1 | Ovary cancer |
| NCT03699449 | NCT03699449-1 | Olaparib | PARP | Ovary cancer |
| NCT03699449 | NCT03699449-2 | Durvalumab | PD-L1 | Ovary cancer |
| NCT03699449 | NCT03699449-2 | Chemotherapy | chemotherapy | Ovary cancer |
| NCT03699449 | NCT03699449-3 | Durvalumab | PD-L1 | Ovary cancer |
| NCT03699449 | NCT03699449-3 | Tremelimumab | CTLA-4 | Ovary cancer |
| NCT03699449 | NCT03699449-3 | Chemotherapy | chemotherapy | Ovary cancer |
| NCT03699449 | NCT03699449-4 | Durvalumab | PD-L1 | Ovary cancer |
| NCT03699449 | NCT03699449-4 | Tremelimumab | CTLA-4 | Ovary cancer |
| NCT03699449 | NCT03699449-4 | Paclitaxel | chemotherapy | Ovary cancer |
| NCT03699449 | NCT03699449-5 | Durvalumab | PD-L1 | Ovary cancer |
| NCT03699449 | NCT03699449-5 | Tremelimumab | CTLA-4 | Ovary cancer |
| NCT03701334 | NCT03701334-0 | Ribociclib | CDK4/CDK6 | Breast cancer |
| NCT03701334 | NCT03701334-0 | Endocrine Therapy | Endocrine Therapy | Breast cancer |
| NCT03702179 | NCT03702179-0 | Durvalumab | PD-L1 | Bladder cancer |
| NCT03702179 | NCT03702179-0 | Tremelimumab | CTLA-4 | Bladder cancer |
| NCT03703297 | NCT03703297-0 | Durvalumab | PD-L1 | lung cancers |
| NCT03703297 | NCT03703297-0 | Tremelimumab | CTLA-4 | lung cancers |
| NCT03704467 | NCT03704467-0 | Platinum-Based Chemotherapy | chemotherapy | Ovary cancer |
| NCT03704467 | NCT03704467-0 | Berzosertib | ATR | Ovary cancer |
| NCT03704467 | NCT03704467-0 | Avelumab | PD-L1 | Ovary cancer |
| NCT03704480 | NCT03704480-0 | Durvalumab | PD-L1 | Gallbladder and biliary tract cancer |
| NCT03704480 | NCT03704480-0 | Tremelimumab | CTLA-4 | Gallbladder and biliary tract cancer |
| NCT03705507 | NCT03705507-0 | Selumetinib | MEK | Leukaemia |
| NCT03705507 | NCT03705507-0 | Dexamethasone | Steroid Treatment | Leukaemia |
| NCT03706365 | NCT03706365-0 | Abiraterone | CYP17A1 | Prostate cancer |
| NCT03706365 | NCT03706365-0 | Prednisone | Steroid Treatment | Prostate cancer |
| NCT03706365 | NCT03706365-0 | Abemaciclib | CDK4/CDK6 | Prostate cancer |
| NCT03707808 | NCT03707808-0 | Autologous Cd1C | oncolytic virus | Other solid tumors |
| NCT03707808 | NCT03707808-0 | Myeloid Dendritic Cells | cell therapy | Other solid tumors |
| NCT03707808 | NCT03707808-0 | Avelumab | PD-L1 | Other solid tumors |
| NCT03707808 | NCT03707808-0 | Ipilimumab | CTLA-4 | Other solid tumors |
| NCT03707808 | NCT03707808-0 | Nivolumab | PD-1 | Other solid tumors |
| NCT03711058 | NCT03711058-0 | Copanlisib | PI3K | Colon and rectum cancers |
| NCT03711058 | NCT03711058-0 | Nivolumab | PD-1 | Colon and rectum cancers |
| NCT03715478 | NCT03715478-0 | Belantamab Mafodotin | BCMA/Tubulin | Multiple Myeloma |
| NCT03715478 | NCT03715478-0 | Pomalidomide | CRBN | Multiple Myeloma |
| NCT03715478 | NCT03715478-0 | Dexamethasone | Steroid Treatment | Multiple Myeloma |
| NCT03717155 | NCT03717155-0 | Avelumab | PD-L1 | lung cancers |
| NCT03717155 | NCT03717155-0 | Cetuximab | EGFR | lung cancers |
| NCT03717155 | NCT03717155-0 | Gemcitabine | chemotherapy | lung cancers |
| NCT03717155 | NCT03717155-0 | Platinum-Based Chemotherapy | chemotherapy | lung cancers |
| NCT03723967 | NCT03723967-1 | Durvalumab | PD-L1 | Head and Neck Neoplasms |
| NCT03723967 | NCT03723967-1 | Platinum-Based Chemotherapy | chemotherapy | Head and Neck Neoplasms |
| NCT03723967 | NCT03723967-2 | Durvalumab | PD-L1 | Head and Neck Neoplasms |
| NCT03723967 | NCT03723967-2 | Paclitaxel | chemotherapy | Head and Neck Neoplasms |
| NCT03724747 | NCT03724747-0 | Bay2315497 | PSMA | Prostate cancer |
| NCT03724747 | NCT03724747-0 | Darolutamide | Endocrine Therapy | Prostate cancer |
| NCT03724890 | NCT03724890-0 | Nedisertib | DNA-PK | Other solid tumors |
| NCT03724890 | NCT03724890-0 | Avelumab | PD-L1 | Other solid tumors |
| NCT03730012 | NCT03730012-0 | Atezolizumab | PD-L1 | Leukaemia |
| NCT03730012 | NCT03730012-0 | Gilteritinib | FLT3 | Leukaemia |
| NCT03732274 | NCT03732274-0 | Vactosertib | ALK | lung cancers |
| NCT03732274 | NCT03732274-0 | Durvalumab | PD-L1 | lung cancers |
| NCT03732677 | NCT03732677-0 | Chemotherapy | chemotherapy | Bladder cancer |
| NCT03732677 | NCT03732677-0 | Durvalumab | PD-L1 | Bladder cancer |
| NCT03732703 | NCT03732703-1 | Abemaciclib | CDK4/CDK6 | Multiple Myeloma |
| NCT03732703 | NCT03732703-1 | Dexamethasone | Steroid Treatment | Multiple Myeloma |
| NCT03732703 | NCT03732703-1 | Ixazomib | PSMB | Multiple Myeloma |
| NCT03732703 | NCT03732703-1 | Pomalidomide | CRBN | Multiple Myeloma |
| NCT03732703 | NCT03732703-2 | Enasidenib | IDH | Multiple Myeloma |
| NCT03732703 | NCT03732703-2 | Dexamethasone | Steroid Treatment | Multiple Myeloma |
| NCT03732703 | NCT03732703-2 | Ixazomib | PSMB | Multiple Myeloma |
| NCT03732703 | NCT03732703-2 | Pomalidomide | CRBN | Multiple Myeloma |
| NCT03732703 | NCT03732703-3 | Cobimetinib | MEK | Multiple Myeloma |
| NCT03732703 | NCT03732703-3 | Dexamethasone | Steroid Treatment | Multiple Myeloma |
| NCT03732703 | NCT03732703-3 | Ixazomib | PSMB | Multiple Myeloma |
| NCT03732703 | NCT03732703-3 | Pomalidomide | CRBN | Multiple Myeloma |
| NCT03732703 | NCT03732703-4 | Erdafitinib | FGFR | Multiple Myeloma |
| NCT03732703 | NCT03732703-4 | Dexamethasone | Steroid Treatment | Multiple Myeloma |
| NCT03732703 | NCT03732703-4 | Ixazomib | PSMB | Multiple Myeloma |
| NCT03732703 | NCT03732703-4 | Pomalidomide | CRBN | Multiple Myeloma |
| NCT03732703 | NCT03732703-5 | Venetoclax | BCL2 | Multiple Myeloma |
| NCT03732703 | NCT03732703-5 | Dexamethasone | Steroid Treatment | Multiple Myeloma |
| NCT03732703 | NCT03732703-5 | Ixazomib | PSMB | Multiple Myeloma |
| NCT03732703 | NCT03732703-5 | Pomalidomide | CRBN | Multiple Myeloma |
| NCT03732703 | NCT03732703-6 | Daratumumab | CD38 | Multiple Myeloma |
| NCT03732703 | NCT03732703-6 | Dexamethasone | Steroid Treatment | Multiple Myeloma |
| NCT03732703 | NCT03732703-6 | Ixazomib | PSMB | Multiple Myeloma |
| NCT03732703 | NCT03732703-6 | Pomalidomide | CRBN | Multiple Myeloma |
| NCT03732703 | NCT03732703-7 | Belantamab Mafodotin | BCMA/Tubulin | Multiple Myeloma |
| NCT03732703 | NCT03732703-7 | Dexamethasone | Steroid Treatment | Multiple Myeloma |
| NCT03732703 | NCT03732703-7 | Ixazomib | PSMB | Multiple Myeloma |
| NCT03732703 | NCT03732703-7 | Pomalidomide | CRBN | Multiple Myeloma |
| NCT03732703 | NCT03732703-8 | Selinexor | XPO1 | Multiple Myeloma |
| NCT03732703 | NCT03732703-8 | Dexamethasone | Steroid Treatment | Multiple Myeloma |
| NCT03732703 | NCT03732703-8 | Ixazomib | PSMB | Multiple Myeloma |
| NCT03732703 | NCT03732703-8 | Pomalidomide | CRBN | Multiple Myeloma |
| NCT03735628 | NCT03735628-0 | Copanlisib | PI3K | Other solid tumors |
| NCT03735628 | NCT03735628-0 | Nivolumab | PD-1 | Other solid tumors |
| NCT03736616 | NCT03736616-0 | Rituximab | CD20 | Lymphomas |
| NCT03736616 | NCT03736616-0 | Ifosfamide | Chemotherapy | Lymphomas |
| NCT03736616 | NCT03736616-0 | Platinum-Based Chemotherapy | chemotherapy | Lymphomas |
| NCT03736616 | NCT03736616-0 | Etoposide | chemotherapy | Lymphomas |
| NCT03736616 | NCT03736616-0 | Acalabrutinib | BTK | Lymphomas |
| NCT03737643 | NCT03737643-1 | Olaparib | PARP | Ovary cancer |
| NCT03737643 | NCT03737643-1 | Bevacizumab | VEGF | Ovary cancer |
| NCT03737643 | NCT03737643-1 | Durvalumab | PD-L1 | Ovary cancer |
| NCT03737643 | NCT03737643-2 | Chemotherapy | chemotherapy | Ovary cancer |
| NCT03737643 | NCT03737643-2 | Bevacizumab | VEGF | Ovary cancer |
| NCT03737643 | NCT03737643-2 | Durvalumab | PD-L1 | Ovary cancer |
| NCT03737968 | NCT03737968-0 | Durvalumab | PD-L1 | Mouth and oropharynx cancers |
| NCT03737968 | NCT03737968-0 | Tremelimumab | CTLA-4 | Mouth and oropharynx cancers |
| NCT03739710 | NCT03739710-0 | Gsk3359609 | ICOS | Others |
| NCT03739710 | NCT03739710-0 | Niraparib | PARP | Others |
| NCT03739814 | NCT03739814-0 | Inotuzumab Ozogamicin | CD22/DNA | Leukaemia |
| NCT03739814 | NCT03739814-0 | Blinatumomab | CD19/CD3 | Leukaemia |
| NCT03739931 | NCT03739931-0 | Mrna-2752 | vaccine | Others |
| NCT03739931 | NCT03739931-0 | Durvalumab | PD-L1 | Others |
| NCT03740334 | NCT03740334-1 | Ribociclib | CDK4/CDK6 | Leukaemia |
| NCT03740334 | NCT03740334-1 | Dexamethasone | Steroid Treatment | Leukaemia |
| NCT03740334 | NCT03740334-2 | Ribociclib | CDK4/CDK6 | Leukaemia |
| NCT03740334 | NCT03740334-2 | Everolimus | mTOR | Leukaemia |
| NCT03740334 | NCT03740334-2 | Dexamethasone | Steroid Treatment | Leukaemia |
| NCT03740529 | NCT03740529-1 | Loxo-305 | BTK | Other hematologic Neoplasms |
| NCT03740529 | NCT03740529-1 | Venetoclax | BCL2 | Other hematologic Neoplasms |
| NCT03740529 | NCT03740529-2 | Loxo-305 | BTK | Other hematologic Neoplasms |
| NCT03740529 | NCT03740529-2 | Venetoclax | BCL2 | Other hematologic Neoplasms |
| NCT03740529 | NCT03740529-2 | Rituximab | CD20 | Other hematologic Neoplasms |
| NCT03741426 | NCT03741426-0 | Olaparib | PARP | Kidney cancer |
| NCT03741426 | NCT03741426-0 | Durvalumab | PD-L1 | Kidney cancer |
| NCT03742102 | NCT03742102-0 | Durvalumab | PD-L1 | Breast cancer |
| NCT03742102 | NCT03742102-0 | Paclitaxel | chemotherapy | Breast cancer |
| NCT03742349 | NCT03742349-0 | Spartalizumab | PD-1 | Breast cancer |
| NCT03742349 | NCT03742349-0 | Lag525 | LAG-3 | Breast cancer |
| NCT03742349 | NCT03742349-0 | Capmatinib | MET | Breast cancer |
| NCT03744793 | NCT03744793-0 | Pemetrexed | chemotherapy | Bladder cancer |
| NCT03744793 | NCT03744793-0 | Avelumab | PD-L1 | Bladder cancer |
| NCT03745989 | NCT03745989-0 | Mk-8353 | ERK | Other solid tumors |
| NCT03745989 | NCT03745989-0 | Selumetinib | MEK | Other solid tumors |
| NCT03747484 | NCT03747484-0 | Tcr-T Cells | cell therapy | Other solid tumors |
| NCT03747484 | NCT03747484-0 | Avelumab | PD-L1 | Other solid tumors |
| NCT03748641 | NCT03748641-0 | Niraparib | PARP | Prostate cancer |
| NCT03748641 | NCT03748641-0 | Abiraterone | CYP17A1 | Prostate cancer |
| NCT03748641 | NCT03748641-0 | Prednisone | Steroid Treatment | Prostate cancer |
| NCT03750071 | NCT03750071-0 | Vxm01 | cell therapy | Brain and nervous system cancers |
| NCT03750071 | NCT03750071-0 | Avelumab | PD-L1 | Brain and nervous system cancers |
| NCT03751124 | NCT03751124-0 | Relugolix | Endocrine Therapy | Other solid tumors |
| NCT03751124 | NCT03751124-0 | Estradiol/Norethindrone | Endocrine Therapy | Other solid tumors |
| NCT03751761 | NCT03751761-0 | Durvalumab | PD-L1 | Stomach cancer |
| NCT03751761 | NCT03751761-0 | Tremelimumab | CTLA-4 | Stomach cancer |
| NCT03751761 | NCT03751761-0 | Paclitaxel | chemotherapy | Stomach cancer |
| NCT03753919 | NCT03753919-0 | Durvalumab | PD-L1 | Other solid tumors |
| NCT03753919 | NCT03753919-0 | Tremelimumab | CTLA-4 | Other solid tumors |
| NCT03755102 | NCT03755102-0 | Dacomitinib | EGFR | lung cancers |
| NCT03755102 | NCT03755102-0 | Osimertinib | EGFR | lung cancers |
| NCT03767244 | NCT03767244-0 | Apalutamide | Endocrine Therapy | Prostate cancer |
| NCT03767244 | NCT03767244-0 | Androgen Deprivation Therapy (Adt) | Endocrine Therapy | Prostate cancer |
| NCT03769181 | NCT03769181-0 | Cemiplimab-Rwlc | PD-1 | Lymphomas |
| NCT03769181 | NCT03769181-0 | Isatuximab | CD38 | Lymphomas |
| NCT03770455 | NCT03770455-0 | Avelumab | PD-L1 | Prostate cancer |
| NCT03770455 | NCT03770455-0 | Androgen Deprivation Therapy (Adt) | Endocrine Therapy | Prostate cancer |
| NCT03772561 | NCT03772561-0 | Capivasertib | AKT | Other solid tumors |
| NCT03772561 | NCT03772561-0 | Olaparib | PARP | Other solid tumors |
| NCT03772561 | NCT03772561-0 | Durvalumab | PD-L1 | Other solid tumors |
| NCT03773666 | NCT03773666-0 | Durvalumab | PD-L1 | Bladder cancer |
| NCT03773666 | NCT03773666-0 | Oleclumab | CD73 | Bladder cancer |
| NCT03775486 | NCT03775486-0 | Durvalumab | PD-L1 | lung cancers |
| NCT03775486 | NCT03775486-0 | Olaparib | PARP | lung cancers |
| NCT03776864 | NCT03776864-0 | Umbralisib | PI3K | Lymphomas |
| NCT03776864 | NCT03776864-0 | Pembrolizumab | PD-1 | Lymphomas |
| NCT03777657 | NCT03777657-1 | Tislelizumab | PD-1 | Stomach cancer |
| NCT03777657 | NCT03777657-1 | Platinum-Based Chemotherapy | chemotherapy | Stomach cancer |
| NCT03777657 | NCT03777657-1 | Capecitabine | chemotherapy | Stomach cancer |
| NCT03777657 | NCT03777657-1 | 5-Fu | chemotherapy | Stomach cancer |
| NCT03777657 | NCT03777657-2 | Tislelizumab | PD-1 | Stomach cancer |
| NCT03777657 | NCT03777657-2 | Platinum-Based Chemotherapy | chemotherapy | Stomach cancer |
| NCT03777657 | NCT03777657-2 | 5-Fu | chemotherapy | Stomach cancer |
| NCT03777813 | NCT03777813-0 | Durvalumab | PD-L1 | Other solid tumors |
| NCT03777813 | NCT03777813-0 | Oxaliplatin | chemotherapy | Other solid tumors |
| NCT03777813 | NCT03777813-0 | Leucovorin | Other supportive therapy | Other solid tumors |
| NCT03777813 | NCT03777813-0 | 5-Fu | chemotherapy | Other solid tumors |
| NCT03777982 | NCT03777982-0 | Prednisone | Steroid Treatment | Prostate cancer |
| NCT03777982 | NCT03777982-0 | Apalutamide | Endocrine Therapy | Prostate cancer |
| NCT03777982 | NCT03777982-0 | Abiraterone | CYP17A1 | Prostate cancer |
| NCT03777982 | NCT03777982-0 | Lhrha | Endocrine Therapy | Prostate cancer |
| NCT03778957 | NCT03778957-0 | Durvalumab | PD-L1 | Liver cancer |
| NCT03778957 | NCT03778957-0 | Bevacizumab | VEGF | Liver cancer |
| NCT03780608 | NCT03780608-0 | Azd6738 | ATR | Other solid tumors |
| NCT03780608 | NCT03780608-0 | Durvalumab | PD-L1 | Other solid tumors |
| NCT03781960 | NCT03781960-0 | Abemaciclib | CDK4/CDK6 | Liver cancer |
| NCT03781960 | NCT03781960-0 | Nivolumab | PD-1 | Liver cancer |
| NCT03783936 | NCT03783936-0 | Mfolfox6 | chemotherapy | Stomach cancer |
| NCT03783936 | NCT03783936-0 | Trastuzumab | HER2 | Stomach cancer |
| NCT03783936 | NCT03783936-0 | Avelumab | PD-L1 | Stomach cancer |
| NCT03783949 | NCT03783949-1 | Ganetespib | HSP90 | Ovary cancer |
| NCT03783949 | NCT03783949-1 | Platinum-Based Chemotherapy | chemotherapy | Ovary cancer |
| NCT03783949 | NCT03783949-1 | Niraparib | PARP | Ovary cancer |
| NCT03783949 | NCT03783949-2 | Ganetespib | HSP90 | Ovary cancer |
| NCT03783949 | NCT03783949-2 | Platinum-Based Chemotherapy | chemotherapy | Ovary cancer |
| NCT03783949 | NCT03783949-2 | Niraparib | PARP | Ovary cancer |
| NCT03784014 | NCT03784014-1 | Trametinib | MEK | Other solid tumors |
| NCT03784014 | NCT03784014-1 | Dabrafenib | RAF | Other solid tumors |
| NCT03784014 | NCT03784014-2 | Olaparib | PARP | Other solid tumors |
| NCT03784014 | NCT03784014-2 | Durvalumab | PD-L1 | Other solid tumors |
| NCT03784066 | NCT03784066-0 | Durvalumab | PD-L1 | Mouth and oropharynx cancers |
| NCT03784066 | NCT03784066-0 | Tremelimumab | CTLA-4 | Mouth and oropharynx cancers |
| NCT03786081 | NCT03786081-1 | Tisotumab Vedotin | TF/Tubulin | Cervix uteri cancer |
| NCT03786081 | NCT03786081-1 | Bevacizumab | VEGF | Cervix uteri cancer |
| NCT03786081 | NCT03786081-2 | Tisotumab Vedotin | TF/Tubulin | Cervix uteri cancer |
| NCT03786081 | NCT03786081-2 | Pembrolizumab | PD-1 | Cervix uteri cancer |
| NCT03786081 | NCT03786081-3 | Tisotumab Vedotin | TF/Tubulin | Cervix uteri cancer |
| NCT03786081 | NCT03786081-3 | Platinum-Based Chemotherapy | chemotherapy | Cervix uteri cancer |
| NCT03786081 | NCT03786081-4 | Tisotumab Vedotin | TF/Tubulin | Cervix uteri cancer |
| NCT03786081 | NCT03786081-4 | Pembrolizumab | PD-1 | Cervix uteri cancer |
| NCT03786081 | NCT03786081-4 | Platinum-Based Chemotherapy | chemotherapy | Cervix uteri cancer |
| NCT03786081 | NCT03786081-5 | Tisotumab Vedotin | TF/Tubulin | Cervix uteri cancer |
| NCT03786081 | NCT03786081-5 | Pembrolizumab | PD-1 | Cervix uteri cancer |
| NCT03786081 | NCT03786081-5 | Platinum-Based Chemotherapy | chemotherapy | Cervix uteri cancer |
| NCT03786081 | NCT03786081-5 | Bevacizumab | VEGF | Cervix uteri cancer |
| NCT03787264 | NCT03787264-0 | Bendamustine | chemotherapy | Leukaemia |
| NCT03787264 | NCT03787264-0 | Obinutuzumab | CD20 | Leukaemia |
| NCT03787264 | NCT03787264-0 | Acalabrutinib | BTK | Leukaemia |
| NCT03787264 | NCT03787264-0 | Venetoclax | BCL2 | Leukaemia |
| NCT03788291 | NCT03788291-0 | Acalabrutinib | BTK | Leukaemia |
| NCT03788291 | NCT03788291-0 | Rituximab | CD20 | Leukaemia |
| NCT03789240 | NCT03789240-0 | Copanlisib | PI3K | Lymphomas |
| NCT03789240 | NCT03789240-0 | Rituximab | CD20 | Lymphomas |
| NCT03794544 | NCT03794544-1 | Durvalumab | PD-L1 | lung cancers |
| NCT03794544 | NCT03794544-1 | Oleclumab | CD73 | lung cancers |
| NCT03794544 | NCT03794544-2 | Durvalumab | PD-L1 | lung cancers |
| NCT03794544 | NCT03794544-2 | Monalizumab | NKG2A | lung cancers |
| NCT03794544 | NCT03794544-3 | Durvalumab | PD-L1 | lung cancers |
| NCT03794544 | NCT03794544-3 | Danvatirsen | STAT3 | lung cancers |
| NCT03798106 | NCT03798106-0 | Durvalumab | PD-L1 | Other solid tumors |
| NCT03798106 | NCT03798106-0 | Pazopanib | VEGFR/PDGFR/FGFR | Other solid tumors |
| NCT03799744 | NCT03799744-0 | Vcn-01 | oncolytic virus | Head and Neck Neoplasms |
| NCT03799744 | NCT03799744-0 | Durvalumab | PD-L1 | Head and Neck Neoplasms |
| NCT03800134 | NCT03800134-0 | Durvalumab | PD-L1 | lung cancers |
| NCT03800134 | NCT03800134-0 | Platinum-Based Chemotherapy | chemotherapy | lung cancers |
| NCT03801369 | NCT03801369-0 | Olaparib | PARP | Breast cancer |
| NCT03801369 | NCT03801369-0 | Durvalumab | PD-L1 | Breast cancer |
| NCT03801525 | NCT03801525-0 | Ublituximab | CD20 | Leukaemia |
| NCT03801525 | NCT03801525-0 | Umbralisib | PI3K | Leukaemia |
| NCT03801525 | NCT03801525-0 | Venetoclax | BCL2 | Leukaemia |
| NCT03802071 | NCT03802071-0 | Durvalumab | PD-L1 | Other solid tumors |
| NCT03802071 | NCT03802071-0 | Doxorubicin | chemotherapy | Other solid tumors |
| NCT03803553 | NCT03803553-0 | Encorafenib | RAF | Colon and rectum cancers |
| NCT03803553 | NCT03803553-0 | Binimetinib | MEK | Colon and rectum cancers |
| NCT03803553 | NCT03803553-0 | Cetuximab | EGFR | Colon and rectum cancers |
| NCT03803761 | NCT03803761-0 | Copanlisib | PI3K | Breast cancer |
| NCT03803761 | NCT03803761-0 | Fulvestrant | Endocrine Therapy | Breast cancer |
| NCT03805594 | NCT03805594-0 | Lutetium Lu 177 Dotatate | SSTR | Prostate cancer |
| NCT03805594 | NCT03805594-0 | Pembrolizumab | PD-1 | Prostate cancer |
| NCT03805932 | NCT03805932-0 | Moxetumomab Pasudotox-Tdfk | CD22/DNA | Leukaemia |
| NCT03805932 | NCT03805932-0 | Rituximab | CD20 | Leukaemia |
| NCT03806049 | NCT03806049-1 | Niraparib | PARP | Ovary cancer |
| NCT03806049 | NCT03806049-1 | Bevacizumab | VEGF | Ovary cancer |
| NCT03806049 | NCT03806049-1 | Dostarlimab-Gxly | PD-1 | Ovary cancer |
| NCT03806049 | NCT03806049-2 | Niraparib | PARP | Ovary cancer |
| NCT03806049 | NCT03806049-2 | Bevacizumab | VEGF | Ovary cancer |
| NCT03810105 | NCT03810105-0 | Olaparib | PARP | Prostate cancer |
| NCT03810105 | NCT03810105-0 | Durvalumab | PD-L1 | Prostate cancer |
| NCT03810807 | NCT03810807-0 | Dacomitinib | EGFR | lung cancers |
| NCT03810807 | NCT03810807-0 | Osimertinib | EGFR | lung cancers |
| NCT03812393 | NCT03812393-0 | Neratinib | EGFR | Breast cancer |
| NCT03812393 | NCT03812393-0 | Chemotherapy | chemotherapy | Breast cancer |
| NCT03812796 | NCT03812796-0 | Domatinostat | chemotherapy | Stomach cancer |
| NCT03812796 | NCT03812796-0 | Avelumab | PD-L1 | Stomach cancer |
| NCT03819465 | NCT03819465-1 | Durvalumab | PD-L1 | lung cancers |
| NCT03819465 | NCT03819465-1 | Danvatirsen | STAT3 | lung cancers |
| NCT03819465 | NCT03819465-2 | Durvalumab | PD-L1 | lung cancers |
| NCT03819465 | NCT03819465-2 | Oleclumab | CD73 | lung cancers |
| NCT03819465 | NCT03819465-3 | Durvalumab | PD-L1 | lung cancers |
| NCT03819465 | NCT03819465-3 | Chemotherapy | chemotherapy | lung cancers |
| NCT03819465 | NCT03819465-4 | Durvalumab | PD-L1 | lung cancers |
| NCT03819465 | NCT03819465-4 | Chemotherapy | chemotherapy | lung cancers |
| NCT03819465 | NCT03819465-4 | Danvatirsen | STAT3 | lung cancers |
| NCT03819465 | NCT03819465-5 | Durvalumab | PD-L1 | lung cancers |
| NCT03819465 | NCT03819465-5 | Chemotherapy | chemotherapy | lung cancers |
| NCT03819465 | NCT03819465-5 | Oleclumab | CD73 | lung cancers |
| NCT03820141 | NCT03820141-0 | Durvalumab | PD-L1 | Breast cancer |
| NCT03820141 | NCT03820141-0 | Trastuzumab | HER2 | Breast cancer |
| NCT03820141 | NCT03820141-0 | Pertuzumab | HER2 | Breast cancer |
| NCT03821792 | NCT03821792-0 | Apalutamide | Endocrine Therapy | Prostate cancer |
| NCT03821792 | NCT03821792-0 | Abiraterone | CYP17A1 | Prostate cancer |
| NCT03821792 | NCT03821792-0 | Prednisone | Steroid Treatment | Prostate cancer |
| NCT03822351 | NCT03822351-1 | Durvalumab | PD-L1 | lung cancers |
| NCT03822351 | NCT03822351-1 | Oleclumab | CD73 | lung cancers |
| NCT03822351 | NCT03822351-2 | Durvalumab | PD-L1 | lung cancers |
| NCT03822351 | NCT03822351-2 | Monalizumab | NKG2A | lung cancers |
| NCT03822468 | NCT03822468-1 | Ribociclib | CDK4/CDK6 | Breast cancer |
| NCT03822468 | NCT03822468-1 | Letrozole | Endocrine Therapy | Breast cancer |
| NCT03822468 | NCT03822468-2 | Ribociclib | CDK4/CDK6 | Breast cancer |
| NCT03822468 | NCT03822468-2 | Anastrozole | Endocrine Therapy | Breast cancer |
| NCT03824483 | NCT03824483-0 | Zanubrutinib | BTK | Leukaemia |
| NCT03824483 | NCT03824483-0 | Obinutuzumab | CD20 | Leukaemia |
| NCT03824483 | NCT03824483-0 | Venetoclax | BCL2 | Leukaemia |
| NCT03824691 | NCT03824691-0 | Cabozantinib | VEGFR/MET/RET | Bladder cancer |
| NCT03824691 | NCT03824691-0 | Durvalumab | PD-L1 | Bladder cancer |
| NCT03825796 | NCT03825796-0 | Cytarabine | chemotherapy | Leukaemia |
| NCT03825796 | NCT03825796-0 | Daunorubicin | chemotherapy | Leukaemia |
| NCT03825796 | NCT03825796-0 | Enasidenib | IDH | Leukaemia |
| NCT03826589 | NCT03826589-0 | Avelumab | PD-L1 | Cervix uteri cancer |
| NCT03826589 | NCT03826589-0 | Axitinib | VEGFR/PDGFR | Cervix uteri cancer |
| NCT03827044 | NCT03827044-0 | Avelumab | PD-L1 | Colon and rectum cancers |
| NCT03827044 | NCT03827044-0 | Chemotherapy | chemotherapy | Colon and rectum cancers |
| NCT03828292 | NCT03828292-1 | Belantamab Mafodotin | BCMA/Tubulin | Multiple Myeloma |
| NCT03828292 | NCT03828292-1 | Bortezomib | PSMB | Multiple Myeloma |
| NCT03828292 | NCT03828292-2 | Belantamab Mafodotin | BCMA/Tubulin | Multiple Myeloma |
| NCT03828292 | NCT03828292-2 | Pomalidomide | CRBN | Multiple Myeloma |
| NCT03828292 | NCT03828292-3 | Belantamab Mafodotin | BCMA/Tubulin | Multiple Myeloma |
| NCT03828292 | NCT03828292-3 | Dexamethasone | Steroid Treatment | Multiple Myeloma |
| NCT03828448 | NCT03828448-0 | Umbralisib | PI3K | Lymphomas |
| NCT03828448 | NCT03828448-0 | Ublituximab | CD20 | Lymphomas |
| NCT03830866 | NCT03830866-0 | Durvalumab | PD-L1 | Cervix uteri cancer |
| NCT03830866 | NCT03830866-0 | Cisplatin | chemotherapy | Cervix uteri cancer |
| NCT03830918 | NCT03830918-0 | Niraparib | PARP | lung cancers |
| NCT03830918 | NCT03830918-0 | Temozolomide | chemotherapy | lung cancers |
| NCT03830918 | NCT03830918-0 | Atezolizumab | PD-L1 | lung cancers |
| NCT03833427 | NCT03833427-0 | Selumetinib | MEK | Others |
| NCT03833427 | NCT03833427-0 | Pembrolizumab | PD-1 | Others |
| NCT03833440 | NCT03833440-1 | Durvalumab | PD-L1 | lung cancers |
| NCT03833440 | NCT03833440-1 | Monalizumab | NKG2A | lung cancers |
| NCT03833440 | NCT03833440-2 | Durvalumab | PD-L1 | lung cancers |
| NCT03833440 | NCT03833440-2 | Oleclumab | CD73 | lung cancers |
| NCT03833440 | NCT03833440-3 | Durvalumab | PD-L1 | lung cancers |
| NCT03833440 | NCT03833440-3 | Azd6738 | ATR | lung cancers |
| NCT03834740 | NCT03834740-0 | Ribociclib | CDK4/CDK6 | Brain and nervous system cancers |
| NCT03834740 | NCT03834740-0 | Everolimus | mTOR | Brain and nervous system cancers |
| NCT03836209 | NCT03836209-0 | Daunorubicin | chemotherapy | Leukaemia |
| NCT03836209 | NCT03836209-0 | Cytarabine | chemotherapy | Leukaemia |
| NCT03836209 | NCT03836209-0 | Midostaurin | PKC/PDGFR | Leukaemia |
| NCT03836261 | NCT03836261-0 | Acalabrutinib | BTK | Leukaemia |
| NCT03836261 | NCT03836261-0 | Venetoclax | BCL2 | Leukaemia |
| NCT03836261 | NCT03836261-0 | Obinutuzumab | CD20 | Leukaemia |
| NCT03837899 | NCT03837899-0 | Durvalumab | PD-L1 | Others |
| NCT03837899 | NCT03837899-0 | Tremelimumab | CTLA-4 | Others |
| NCT03839342 | NCT03839342-0 | Binimetinib | MEK | Other solid tumors |
| NCT03839342 | NCT03839342-0 | Encorafenib | RAF | Other solid tumors |
| NCT03839823 | NCT03839823-0 | Nsai | Endocrine Therapy | Breast cancer |
| NCT03839823 | NCT03839823-0 | Goserelin | Endocrine Therapy | Breast cancer |
| NCT03839823 | NCT03839823-0 | Ribociclib | CDK4/CDK6 | Breast cancer |
| NCT03842228 | NCT03842228-0 | Copanlisib | PI3K | Other solid tumors |
| NCT03842228 | NCT03842228-0 | Olaparib | PARP | Other solid tumors |
| NCT03842228 | NCT03842228-0 | Durvalumab | PD-L1 | Other solid tumors |
| NCT03843775 | NCT03843775-0 | Binimetinib | MEK | Others |
| NCT03843775 | NCT03843775-0 | Encorafenib | RAF | Others |
| NCT03844763 | NCT03844763-0 | Cyclophosphamide | chemotherapy | Head and Neck Neoplasms |
| NCT03844763 | NCT03844763-0 | Avelumab | PD-L1 | Head and Neck Neoplasms |
| NCT03845166 | NCT03845166-1 | Xl092 | VEGFR/MET/AXL | Other solid tumors |
| NCT03845166 | NCT03845166-1 | Atezolizumab | PD-L1 | Other solid tumors |
| NCT03845166 | NCT03845166-2 | Xl092 | VEGFR/MET/AXL | Other solid tumors |
| NCT03845166 | NCT03845166-2 | Avelumab | PD-L1 | Other solid tumors |
| NCT03847428 | NCT03847428-0 | Durvalumab | PD-L1 | Liver cancer |
| NCT03847428 | NCT03847428-0 | Bevacizumab | VEGF | Liver cancer |
| NCT03847649 | NCT03847649-0 | Durvalumab | PD-L1 | Others |
| NCT03847649 | NCT03847649-0 | Prednisone | Steroid Treatment | Others |
| NCT03848845 | NCT03848845-0 | Belantamab Mafodotin | BCMA/Tubulin | Multiple Myeloma |
| NCT03848845 | NCT03848845-0 | Pembrolizumab | PD-1 | Multiple Myeloma |
| NCT03851081 | NCT03851081-0 | Inotuzumab Ozogamicin | CD22/DNA | Leukaemia |
| NCT03851081 | NCT03851081-0 | Vincristine | chemotherapy | Leukaemia |
| NCT03851614 | NCT03851614-1 | Olaparib | PARP | Other solid tumors |
| NCT03851614 | NCT03851614-1 | Durvalumab | PD-L1 | Other solid tumors |
| NCT03851614 | NCT03851614-2 | Cediranib | KDR | Other solid tumors |
| NCT03851614 | NCT03851614-2 | Durvalumab | PD-L1 | Other solid tumors |
| NCT03854474 | NCT03854474-0 | Tazemetostat | EZH2 | Bladder cancer |
| NCT03854474 | NCT03854474-0 | Pembrolizumab | PD-1 | Bladder cancer |
| NCT03854799 | NCT03854799-0 | Avelumab | PD-L1 | Colon and rectum cancers |
| NCT03854799 | NCT03854799-0 | Capecitabine | chemotherapy | Colon and rectum cancers |
| NCT03856216 | NCT03856216-0 | Inotuzumab Ozogamicin | CD22/DNA | Leukaemia |
| NCT03856216 | NCT03856216-0 | Chemotherapy | chemotherapy | Leukaemia |
| NCT03860844 | NCT03860844-0 | Isatuximab | CD38 | Leukaemia |
| NCT03860844 | NCT03860844-0 | Chemotherapy | chemotherapy | Leukaemia |
| NCT03863184 | NCT03863184-0 | Acalabrutinib | BTK | Lymphomas |
| NCT03863184 | NCT03863184-0 | Lenalidomide | CRBN | Lymphomas |
| NCT03863184 | NCT03863184-0 | Rituximab | CD20 | Lymphomas |
| NCT03864042 | NCT03864042-0 | Binimetinib | MEK | Melanoma |
| NCT03864042 | NCT03864042-0 | Encorafenib | RAF | Melanoma |
| NCT03868722 | NCT03868722-0 | Acalabrutinib | BTK | Leukaemia |
| NCT03868722 | NCT03868722-0 | Venetoclax | BCL2 | Leukaemia |
| NCT03869190 | NCT03869190-1 | Atezolizumab | PD-L1 | Bladder cancer |
| NCT03869190 | NCT03869190-1 | Enfortumab Vedotin | NECTIN4/Tubulin | Bladder cancer |
| NCT03869190 | NCT03869190-2 | Atezolizumab | PD-L1 | Bladder cancer |
| NCT03869190 | NCT03869190-2 | Niraparib | PARP | Bladder cancer |
| NCT03869190 | NCT03869190-3 | Atezolizumab | PD-L1 | Bladder cancer |
| NCT03869190 | NCT03869190-3 | Magrolimab | CD47 | Bladder cancer |
| NCT03869190 | NCT03869190-4 | Atezolizumab | PD-L1 | Bladder cancer |
| NCT03869190 | NCT03869190-4 | Tiragolumab | TIGIT | Bladder cancer |
| NCT03869190 | NCT03869190-5 | Atezolizumab | PD-L1 | Bladder cancer |
| NCT03869190 | NCT03869190-5 | Sacituzumab Govitecan | Trop-2/TOP1 | Bladder cancer |
| NCT03869190 | NCT03869190-6 | Atezolizumab | PD-L1 | Bladder cancer |
| NCT03869190 | NCT03869190-6 | Tocilizumab | IL6 | Bladder cancer |
| NCT03869190 | NCT03869190-7 | Atezolizumab | PD-L1 | Bladder cancer |
| NCT03869190 | NCT03869190-7 | Ro7122290 | FAP/4-1BB | Bladder cancer |
| NCT03871036 | NCT03871036-0 | Tremelimumab | CTLA-4 | Bladder cancer |
| NCT03871036 | NCT03871036-0 | Durvalumab | PD-L1 | Bladder cancer |
| NCT03871153 | NCT03871153-0 | Durvalumab | PD-L1 | lung cancers |
| NCT03871153 | NCT03871153-0 | Paclitaxel | chemotherapy | lung cancers |
| NCT03871153 | NCT03871153-0 | Carboplatin | chemotherapy | lung cancers |
| NCT03871348 | NCT03871348-0 | Sar441000 | vaccine | Others |
| NCT03871348 | NCT03871348-0 | Cemiplimab-Rwlc | PD-1 | Others |
| NCT03872505 | NCT03872505-0 | Platinum-Based Chemotherapy | chemotherapy | Breast cancer |
| NCT03872505 | NCT03872505-0 | Paclitaxel | chemotherapy | Breast cancer |
| NCT03872505 | NCT03872505-0 | Durvalumab | PD-L1 | Breast cancer |
| NCT03874325 | NCT03874325-0 | Durvalumab | PD-L1 | Breast cancer |
| NCT03874325 | NCT03874325-0 | Aromatase Inhibitor | Endocrine Therapy | Breast cancer |
| NCT03875235 | NCT03875235-0 | Durvalumab | PD-L1 | Gallbladder and biliary tract cancer |
| NCT03875235 | NCT03875235-0 | Gemcitabine | chemotherapy | Gallbladder and biliary tract cancer |
| NCT03875235 | NCT03875235-0 | Platinum-Based Chemotherapy | chemotherapy | Gallbladder and biliary tract cancer |
| NCT03875573 | NCT03875573-1 | Chemotherapy | chemotherapy | Breast cancer |
| NCT03875573 | NCT03875573-1 | Durvalumab | PD-L1 | Breast cancer |
| NCT03875573 | NCT03875573-2 | Paclitaxel | chemotherapy | Breast cancer |
| NCT03875573 | NCT03875573-2 | Doxorubicin | chemotherapy | Breast cancer |
| NCT03875573 | NCT03875573-2 | Cyclophosphamide | chemotherapy | Breast cancer |
| NCT03875573 | NCT03875573-2 | Durvalumab | PD-L1 | Breast cancer |
| NCT03875573 | NCT03875573-2 | Oleclumab | CD73 | Breast cancer |
| NCT03877055 | NCT03877055-0 | Copanlisib | PI3K | Lymphomas |
| NCT03877055 | NCT03877055-0 | Ibrutinib | BTK | Lymphomas |
| NCT03878719 | NCT03878719-0 | Binimetinib | MEK | Melanoma |
| NCT03878719 | NCT03878719-0 | Encorafenib | RAF | Melanoma |
| NCT03884998 | NCT03884998-0 | Copanlisib | PI3K | Other hematologic Neoplasms |
| NCT03884998 | NCT03884998-0 | Nivolumab | PD-1 | Other hematologic Neoplasms |
| NCT03886649 | NCT03886649-0 | Copanlisib | PI3K | Lymphomas |
| NCT03886649 | NCT03886649-0 | Venetoclax | BCL2 | Lymphomas |
| NCT03889275 | NCT03889275-0 | Medi5395 | oncolytic virus | Other solid tumors |
| NCT03889275 | NCT03889275-0 | Durvalumab | PD-L1 | Other solid tumors |
| NCT03891615 | NCT03891615-0 | Niraparib | PARP | lung cancers |
| NCT03891615 | NCT03891615-0 | Osimertinib | EGFR | lung cancers |
| NCT03892044 | NCT03892044-0 | Duvelisib | PI3K | Other hematologic Neoplasms |
| NCT03892044 | NCT03892044-0 | Nivolumab | PD-1 | Other hematologic Neoplasms |
| NCT03892642 | NCT03892642-0 | Bacille Calmette-Guérin | vaccine | Bladder cancer |
| NCT03892642 | NCT03892642-0 | Avelumab | PD-L1 | Bladder cancer |
| NCT03893903 | NCT03893903-0 | Idh1R132H Peptide Vaccine | vaccine | Brain and nervous system cancers |
| NCT03893903 | NCT03893903-0 | Avelumab | PD-L1 | Brain and nervous system cancers |
| NCT03895788 | NCT03895788-0 | Niraparib | PARP | Ovary cancer |
| NCT03895788 | NCT03895788-0 | Brivanib | VEGFR/FGFR | Ovary cancer |
| NCT03898908 | NCT03898908-0 | Binimetinib | MEK | Melanoma |
| NCT03898908 | NCT03898908-0 | Encorafenib | RAF | Melanoma |
| NCT03899337 | NCT03899337-0 | Acalabrutinib | BTK | Other hematologic Neoplasms |
| NCT03899337 | NCT03899337-0 | Rituximab | CD20 | Other hematologic Neoplasms |
| NCT03899337 | NCT03899337-0 | Chemotherapy | chemotherapy | Other hematologic Neoplasms |
| NCT03899610 | NCT03899610-0 | Chemotherapy | chemotherapy | Ovary cancer |
| NCT03899610 | NCT03899610-0 | Durvalumab | PD-L1 | Ovary cancer |
| NCT03899610 | NCT03899610-0 | Tremelimumab | CTLA-4 | Ovary cancer |
| NCT03900949 | NCT03900949-0 | Gemtuzumab Ozogamicin | CD33/DNA | Leukaemia |
| NCT03900949 | NCT03900949-0 | Midostaurin | PKC/PDGFR | Leukaemia |
| NCT03900949 | NCT03900949-0 | Cytarabine | chemotherapy | Leukaemia |
| NCT03900949 | NCT03900949-0 | Daunorubicin | chemotherapy | Leukaemia |
| NCT03901469 | NCT03901469-0 | Zen003694 | BET | Breast cancer |
| NCT03901469 | NCT03901469-0 | Talazoparib | PARP | Breast cancer |
| NCT03902951 | NCT03902951-0 | Leuprolide | Endocrine Therapy | Prostate cancer |
| NCT03902951 | NCT03902951-0 | Apalutamide | Endocrine Therapy | Prostate cancer |
| NCT03902951 | NCT03902951-0 | Abiraterone | CYP17A1 | Prostate cancer |
| NCT03905135 | NCT03905135-0 | Interleukin-15 | IL15 | Lymphomas |
| NCT03905135 | NCT03905135-0 | Avelumab | PD-L1 | Lymphomas |
| NCT03905889 | NCT03905889-0 | Abemaciclib | CDK4/CDK6 | Kidney cancer |
| NCT03905889 | NCT03905889-0 | Sunitinib | VEGFR/PDGFR | Kidney cancer |
| NCT03906292 | NCT03906292-1 | Imatinib | BCR-ABL | Leukaemia |
| NCT03906292 | NCT03906292-1 | Asciminib | BCR-ABL | Leukaemia |
| NCT03906292 | NCT03906292-2 | Nilotinib | BCR-ABL | Leukaemia |
| NCT03906292 | NCT03906292-2 | Asciminib | BCR-ABL | Leukaemia |
| NCT03906292 | NCT03906292-3 | Dasatinib | BCR-ABL | Leukaemia |
| NCT03906292 | NCT03906292-3 | Asciminib | BCR-ABL | Leukaemia |
| NCT03907475 | NCT03907475-1 | Gemcitabine | chemotherapy | Other solid tumors |
| NCT03907475 | NCT03907475-1 | Durvalumab | PD-L1 | Other solid tumors |
| NCT03907475 | NCT03907475-2 | Doxorubicin | chemotherapy | Other solid tumors |
| NCT03907475 | NCT03907475-2 | Durvalumab | PD-L1 | Other solid tumors |
| NCT03907475 | NCT03907475-3 | Capecitabine | chemotherapy | Other solid tumors |
| NCT03907475 | NCT03907475-3 | Durvalumab | PD-L1 | Other solid tumors |
| NCT03907475 | NCT03907475-4 | Platinum-Based Chemotherapy | chemotherapy | Other solid tumors |
| NCT03907475 | NCT03907475-4 | Durvalumab | PD-L1 | Other solid tumors |
| NCT03907475 | NCT03907475-5 | Paclitaxel | chemotherapy | Other solid tumors |
| NCT03907475 | NCT03907475-5 | Durvalumab | PD-L1 | Other solid tumors |
| NCT03911557 | NCT03911557-0 | Durvalumab | PD-L1 | Other solid tumors |
| NCT03911557 | NCT03911557-0 | Tremelimumab | CTLA-4 | Other solid tumors |
| NCT03911869 | NCT03911869-0 | Binimetinib | MEK | Others |
| NCT03911869 | NCT03911869-0 | Encorafenib | RAF | Others |
| NCT03911973 | NCT03911973-0 | Talazoparib | PARP | Breast cancer |
| NCT03911973 | NCT03911973-0 | Gedatolisib | PI3K/mTOR | Breast cancer |
| NCT03912818 | NCT03912818-1 | Durvalumab | PD-L1 | Bladder cancer |
| NCT03912818 | NCT03912818-1 | Platinum-Based Chemotherapy | chemotherapy | Bladder cancer |
| NCT03912818 | NCT03912818-1 | Gemcitabine | chemotherapy | Bladder cancer |
| NCT03912818 | NCT03912818-2 | Durvalumab | PD-L1 | Bladder cancer |
| NCT03912818 | NCT03912818-2 | Methotrexate | chemotherapy | Bladder cancer |
| NCT03912818 | NCT03912818-2 | Vinblastine | chemotherapy | Bladder cancer |
| NCT03912818 | NCT03912818-2 | Doxorubicin | chemotherapy | Bladder cancer |
| NCT03912818 | NCT03912818-2 | Platinum-Based Chemotherapy | chemotherapy | Bladder cancer |
| NCT03913234 | NCT03913234-0 | Letrozole | Endocrine Therapy | Breast cancer |
| NCT03913234 | NCT03913234-0 | Trastuzumab | HER2 | Breast cancer |
| NCT03913234 | NCT03913234-0 | Ribociclib | CDK4/CDK6 | Breast cancer |
| NCT03915405 | NCT03915405-0 | Khk2455 | IDO1 | Bladder cancer |
| NCT03915405 | NCT03915405-0 | Avelumab | PD-L1 | Bladder cancer |
| NCT03915951 | NCT03915951-0 | Binimetinib | MEK | lung cancers |
| NCT03915951 | NCT03915951-0 | Encorafenib | RAF | lung cancers |
| NCT03916419 | NCT03916419-0 | Durvalumab | PD-L1 | lung cancers |
| NCT03916419 | NCT03916419-0 | Carboplatin | chemotherapy | lung cancers |
| NCT03916419 | NCT03916419-0 | Paclitaxel | chemotherapy | lung cancers |
| NCT03916627 | NCT03916627-0 | Cemiplimab-Rwlc | PD-1 | Other solid tumors |
| NCT03916627 | NCT03916627-0 | Platinum-Based Chemotherapy | chemotherapy | Other solid tumors |
| NCT03919175 | NCT03919175-0 | Umbralisib | PI3K | Lymphomas |
| NCT03919175 | NCT03919175-0 | Rituximab | CD20 | Lymphomas |
| NCT03919292 | NCT03919292-0 | Neratinib | EGFR | Other solid tumors |
| NCT03919292 | NCT03919292-0 | Divalproex | chemotherapy | Other solid tumors |
| NCT03923270 | NCT03923270-1 | Durvalumab | PD-L1 | lung cancers |
| NCT03923270 | NCT03923270-1 | Tremelimumab | CTLA-4 | lung cancers |
| NCT03923270 | NCT03923270-2 | Durvalumab | PD-L1 | lung cancers |
| NCT03923270 | NCT03923270-2 | Olaparib | PARP | lung cancers |
| NCT03937830 | NCT03937830-0 | Durvalumab | PD-L1 | Liver cancer |
| NCT03937830 | NCT03937830-0 | Bevacizumab | VEGF | Liver cancer |
| NCT03937830 | NCT03937830-0 | Tremelimumab | CTLA-4 | Liver cancer |
| NCT03940703 | NCT03940703-0 | Tepotinib | MET | lung cancers |
| NCT03940703 | NCT03940703-0 | Osimertinib | EGFR | lung cancers |
| NCT03941262 | NCT03941262-0 | Snk01 | cell therapy | Other solid tumors |
| NCT03941262 | NCT03941262-0 | Avelumab | PD-L1 | Other solid tumors |
| NCT03944057 | NCT03944057-0 | Selinexor | XPO1 | Multiple Myeloma |
| NCT03944057 | NCT03944057-0 | Dexamethasone | Steroid Treatment | Multiple Myeloma |
| NCT03944252 | NCT03944252-0 | Cetuximab | EGFR | Others |
| NCT03944252 | NCT03944252-0 | Avelumab | PD-L1 | Others |
| NCT03944434 | NCT03944434-0 | Ribociclib | CDK4/CDK6 | Breast cancer |
| NCT03944434 | NCT03944434-0 | Aromatase Inhibitor | Endocrine Therapy | Breast cancer |
| NCT03944434 | NCT03944434-0 | Nonsteroideal | Other supportive therapy | Breast cancer |
| NCT03944434 | NCT03944434-0 | Lhrha | Endocrine Therapy | Breast cancer |
| NCT03944772 | NCT03944772-1 | Osimertinib | EGFR | lung cancers |
| NCT03944772 | NCT03944772-1 | Savolitinib | MET | lung cancers |
| NCT03944772 | NCT03944772-2 | Osimertinib | EGFR | lung cancers |
| NCT03944772 | NCT03944772-2 | Gefitinib | EGFR | lung cancers |
| NCT03944772 | NCT03944772-3 | Osimertinib | EGFR | lung cancers |
| NCT03944772 | NCT03944772-3 | Necitumumab | EGFR | lung cancers |
| NCT03944772 | NCT03944772-4 | Carboplatin | chemotherapy | lung cancers |
| NCT03944772 | NCT03944772-4 | Pemetrexed | chemotherapy | lung cancers |
| NCT03944772 | NCT03944772-4 | Durvalumab | PD-L1 | lung cancers |
| NCT03944772 | NCT03944772-5 | Osimertinib | EGFR | lung cancers |
| NCT03944772 | NCT03944772-5 | Alectinib | ALK | lung cancers |
| NCT03944772 | NCT03944772-6 | Osimertinib | EGFR | lung cancers |
| NCT03944772 | NCT03944772-6 | Selpercatinib | RET | lung cancers |
| NCT03944772 | NCT03944772-7 | Etoposide | chemotherapy | lung cancers |
| NCT03944772 | NCT03944772-7 | Durvalumab | PD-L1 | lung cancers |
| NCT03944772 | NCT03944772-7 | Carboplatin | chemotherapy | lung cancers |
| NCT03944772 | NCT03944772-8 | Osimertinib | EGFR | lung cancers |
| NCT03944772 | NCT03944772-8 | Pemetrexed | chemotherapy | lung cancers |
| NCT03944772 | NCT03944772-8 | Carboplatin | chemotherapy | lung cancers |
| NCT03944772 | NCT03944772-9 | Osimertinib | EGFR | lung cancers |
| NCT03944772 | NCT03944772-9 | Selumetinib | MEK | lung cancers |
| NCT03944772 | NCT03944772-10 | Osimertinib | EGFR | lung cancers |
| NCT03944772 | NCT03944772-10 | Datopotamab Deruxtecan | Trop-2/TOP1 | lung cancers |
| NCT03944902 | NCT03944902-0 | Niraparib | PARP | Ovary cancer |
| NCT03944902 | NCT03944902-0 | Telaglenastat | GLS1 | Ovary cancer |
| NCT03944941 | NCT03944941-0 | Cetuximab | EGFR | Others |
| NCT03944941 | NCT03944941-0 | Avelumab | PD-L1 | Others |
| NCT03946800 | NCT03946800-0 | Medi1191 | vaccine | Other solid tumors |
| NCT03946800 | NCT03946800-0 | Durvalumab | PD-L1 | Other solid tumors |
| NCT03946878 | NCT03946878-0 | Acalabrutinib | BTK | Lymphomas |
| NCT03946878 | NCT03946878-0 | Venetoclax | BCL2 | Lymphomas |
| NCT03947385 | NCT03947385-1 | Ide196 | PKC | Other solid tumors |
| NCT03947385 | NCT03947385-1 | Binimetinib | MEK | Other solid tumors |
| NCT03947385 | NCT03947385-2 | Ide196 | PKC | Other solid tumors |
| NCT03947385 | NCT03947385-2 | Crizotinib | ALK/MET | Other solid tumors |
| NCT03951415 | NCT03951415-0 | Olaparib | PARP | Corpus uteri cancer |
| NCT03951415 | NCT03951415-0 | Durvalumab | PD-L1 | Corpus uteri cancer |
| NCT03951831 | NCT03951831-0 | Cemiplimab-Rwlc | PD-1 | Prostate cancer |
| NCT03951831 | NCT03951831-0 | Docetaxel | chemotherapy | Prostate cancer |
| NCT03955471 | NCT03955471-0 | Niraparib | PARP | Ovary cancer |
| NCT03955471 | NCT03955471-0 | Dostarlimab-Gxly | PD-1 | Ovary cancer |
| NCT03955783 | NCT03955783-0 | Selinexor | XPO1 | Other hematologic Neoplasms |
| NCT03955783 | NCT03955783-0 | Venetoclax | BCL2 | Other hematologic Neoplasms |
| NCT03959293 | NCT03959293-1 | Folfiri | chemotherapy | Stomach cancer |
| NCT03959293 | NCT03959293-1 | Durvalumab | PD-L1 | Stomach cancer |
| NCT03959293 | NCT03959293-2 | Folfiri | chemotherapy | Stomach cancer |
| NCT03959293 | NCT03959293-2 | Durvalumab | PD-L1 | Stomach cancer |
| NCT03959293 | NCT03959293-2 | Tremelimumab | CTLA-4 | Stomach cancer |
| NCT03962465 | NCT03962465-0 | Prednisone | Steroid Treatment | Other hematologic Neoplasms |
| NCT03962465 | NCT03962465-0 | Vincristine | chemotherapy | Other hematologic Neoplasms |
| NCT03962465 | NCT03962465-0 | Daunorubicin | chemotherapy | Other hematologic Neoplasms |
| NCT03962465 | NCT03962465-0 | Inotuzumab Ozogamicin | CD22/DNA | Other hematologic Neoplasms |
| NCT03963414 | NCT03963414-0 | Durvalumab | PD-L1 | lung cancers |
| NCT03963414 | NCT03963414-0 | Tremelimumab | CTLA-4 | lung cancers |
| NCT03964532 | NCT03964532-0 | Avelumab | PD-L1 | Breast cancer |
| NCT03964532 | NCT03964532-0 | Talazoparib | PARP | Breast cancer |
| NCT03965468 | NCT03965468-0 | Chemotherapy | chemotherapy | lung cancers |
| NCT03965468 | NCT03965468-0 | Durvalumab | PD-L1 | lung cancers |
| NCT03966118 | NCT03966118-0 | Ramucirumab | KDR | Stomach cancer |
| NCT03966118 | NCT03966118-0 | Avelumab | PD-L1 | Stomach cancer |
| NCT03966118 | NCT03966118-0 | Paclitaxel | chemotherapy | Stomach cancer |
| NCT03970616 | NCT03970616-0 | Tivozanib | VEGFR/PDGFR | Liver cancer |
| NCT03970616 | NCT03970616-0 | Durvalumab | PD-L1 | Liver cancer |
| NCT03971409 | NCT03971409-1 | Avelumab | PD-L1 | Breast cancer |
| NCT03971409 | NCT03971409-1 | Doxorubicin | chemotherapy | Breast cancer |
| NCT03971409 | NCT03971409-2 | Avelumab | PD-L1 | Breast cancer |
| NCT03971409 | NCT03971409-2 | Sacituzumab Govitecan | Trop-2/TOP1 | Breast cancer |
| NCT03971409 | NCT03971409-3 | Avelumab | PD-L1 | Breast cancer |
| NCT03971409 | NCT03971409-3 | Binimetinib | MEK | Breast cancer |
| NCT03971409 | NCT03971409-3 | Doxorubicin | chemotherapy | Breast cancer |
| NCT03971409 | NCT03971409-4 | Utomilumab | 4-1BB | Breast cancer |
| NCT03971409 | NCT03971409-4 | Avelumab | PD-L1 | Breast cancer |
| NCT03971409 | NCT03971409-5 | Pf-04518600 | OX40 | Breast cancer |
| NCT03971409 | NCT03971409-5 | Avelumab | PD-L1 | Breast cancer |
| NCT03971409 | NCT03971409-6 | Binimetinib | MEK | Breast cancer |
| NCT03971409 | NCT03971409-6 | Avelumab | PD-L1 | Breast cancer |
| NCT03972488 | NCT03972488-0 | Lutetium Lu 177 Dotatate | SSTR | Stomach cancer |
| NCT03972488 | NCT03972488-0 | Octreotide | Endocrine Therapy | Stomach cancer |
| NCT03972657 | NCT03972657-0 | Regn5678 | CD28 | Prostate cancer |
| NCT03972657 | NCT03972657-0 | Cemiplimab-Rwlc | PD-1 | Prostate cancer |
| NCT03973918 | NCT03973918-0 | Binimetinib | MEK | Brain and nervous system cancers |
| NCT03973918 | NCT03973918-0 | Encorafenib | RAF | Brain and nervous system cancers |
| NCT03975114 | NCT03975114-0 | Chemotherapy | chemotherapy | lung cancers |
| NCT03975114 | NCT03975114-0 | Durvalumab | PD-L1 | lung cancers |
| NCT03975647 | NCT03975647-0 | Tucatinib | HER2 | Breast cancer |
| NCT03975647 | NCT03975647-0 | Trastuzumab Emtansine | HER2/Tubulin | Breast cancer |
| NCT03979131 | NCT03979131-0 | Avelumab | PD-L1 | Stomach cancer |
| NCT03979131 | NCT03979131-0 | Chemotherapy | chemotherapy | Stomach cancer |
| NCT03981614 | NCT03981614-0 | Binimetinib | MEK | Colon and rectum cancers |
| NCT03981614 | NCT03981614-0 | Palbociclib | CDK4/CDK6 | Colon and rectum cancers |
| NCT03981796 | NCT03981796-0 | Dostarlimab-Gxly | PD-1 | Others |
| NCT03981796 | NCT03981796-0 | Niraparib | PARP | Others |
| NCT03981796 | NCT03981796-0 | Platinum-Based Chemotherapy | chemotherapy | Others |
| NCT03981796 | NCT03981796-0 | Paclitaxel | chemotherapy | Others |
| NCT03982173 | NCT03982173-0 | Durvalumab | PD-L1 | Other solid tumors |
| NCT03982173 | NCT03982173-0 | Tremelimumab | CTLA-4 | Other solid tumors |
| NCT03983226 | NCT03983226-0 | Chemotherapy | chemotherapy | Ovary cancer |
| NCT03983226 | NCT03983226-0 | Niraparib | PARP | Ovary cancer |
| NCT03983954 | NCT03983954-1 | Naptumomab Estafenatox | 5T4 | Other solid tumors |
| NCT03983954 | NCT03983954-1 | Durvalumab | PD-L1 | Other solid tumors |
| NCT03983954 | NCT03983954-2 | Obinutuzumab | CD20 | Other solid tumors |
| NCT03983954 | NCT03983954-2 | Durvalumab | PD-L1 | Other solid tumors |
| NCT03983954 | NCT03983954-3 | Obinutuzumab | CD20 | Other solid tumors |
| NCT03983954 | NCT03983954-3 | Naptumomab Estafenatox | 5T4 | Other solid tumors |
| NCT03983954 | NCT03983954-3 | Durvalumab | PD-L1 | Other solid tumors |
| NCT03983993 | NCT03983993-0 | Niraparib | PARP | Colon and rectum cancers |
| NCT03983993 | NCT03983993-0 | Panitumumab | EGFR | Colon and rectum cancers |
| NCT03989414 | NCT03989414-1 | Cc-92480 | CRBN | Multiple Myeloma |
| NCT03989414 | NCT03989414-1 | Elotuzumab | SLAMF7 | Multiple Myeloma |
| NCT03989414 | NCT03989414-1 | Dexamethasone | Steroid Treatment | Multiple Myeloma |
| NCT03989414 | NCT03989414-2 | Cc-92480 | CRBN | Multiple Myeloma |
| NCT03989414 | NCT03989414-2 | Isatuximab | CD38 | Multiple Myeloma |
| NCT03989414 | NCT03989414-2 | Dexamethasone | Steroid Treatment | Multiple Myeloma |
| NCT03989414 | NCT03989414-3 | Cc-92480 | CRBN | Multiple Myeloma |
| NCT03989414 | NCT03989414-3 | Daratumumab | CD38 | Multiple Myeloma |
| NCT03989414 | NCT03989414-3 | Dexamethasone | Steroid Treatment | Multiple Myeloma |
| NCT03990571 | NCT03990571-0 | Axitinib | VEGFR/PDGFR | Other solid tumors |
| NCT03990571 | NCT03990571-0 | Avelumab | PD-L1 | Other solid tumors |
| NCT03991819 | NCT03991819-0 | Binimetinib | MEK | lung cancers |
| NCT03991819 | NCT03991819-0 | Pembrolizumab | PD-1 | lung cancers |
| NCT03991832 | NCT03991832-0 | Olaparib | PARP | Other solid tumors |
| NCT03991832 | NCT03991832-0 | Durvalumab | PD-L1 | Other solid tumors |
| NCT03991884 | NCT03991884-0 | Etoposide | chemotherapy | Other hematologic Neoplasms |
| NCT03991884 | NCT03991884-0 | Doxorubicin | chemotherapy | Other hematologic Neoplasms |
| NCT03991884 | NCT03991884-0 | Vincristine | chemotherapy | Other hematologic Neoplasms |
| NCT03991884 | NCT03991884-0 | Prednisone | Steroid Treatment | Other hematologic Neoplasms |
| NCT03991884 | NCT03991884-0 | Cyclophosphamide | chemotherapy | Other hematologic Neoplasms |
| NCT03991884 | NCT03991884-0 | Inotuzumab Ozogamicin | CD22/DNA | Other hematologic Neoplasms |
| NCT03992131 | NCT03992131-1 | Rucaparib | PARP | Other solid tumors |
| NCT03992131 | NCT03992131-1 | Lucitanib | VEGFR/FGFR | Other solid tumors |
| NCT03992131 | NCT03992131-2 | Rucaparib | PARP | Other solid tumors |
| NCT03992131 | NCT03992131-2 | Sacituzumab Govitecan | Trop-2/TOP1 | Other solid tumors |
| NCT03994393 | NCT03994393-0 | Durvalumab | PD-L1 | lung cancers |
| NCT03994393 | NCT03994393-0 | Tremelimumab | CTLA-4 | lung cancers |
| NCT03997448 | NCT03997448-0 | Abemaciclib | CDK4/CDK6 | Stomach cancer |
| NCT03997448 | NCT03997448-0 | Pembrolizumab | PD-1 | Stomach cancer |
| NCT03999515 | NCT03999515-0 | Abiraterone | CYP17A1 | Prostate cancer |
| NCT03999515 | NCT03999515-0 | Enzalutamide | Endocrine Therapy | Prostate cancer |
| NCT03999515 | NCT03999515-0 | Erdafitinib | FGFR | Prostate cancer |
| NCT04000529 | NCT04000529-0 | Tno155 | SHP2 | Other solid tumors |
| NCT04000529 | NCT04000529-0 | Ribociclib | CDK4/CDK6 | Other solid tumors |
| NCT04002297 | NCT04002297-0 | Zanubrutinib | BTK | Lymphomas |
| NCT04002297 | NCT04002297-0 | Rituximab | CD20 | Lymphomas |
| NCT04002947 | NCT04002947-0 | Chemotherapy | chemotherapy | Lymphomas |
| NCT04002947 | NCT04002947-0 | Acalabrutinib | BTK | Lymphomas |
| NCT04002947 | NCT04002947-0 | Rituximab | CD20 | Lymphomas |
| NCT04003610 | NCT04003610-0 | Pemigatinib | FGFR | Bladder cancer |
| NCT04003610 | NCT04003610-0 | Pembrolizumab | PD-1 | Bladder cancer |
| NCT04004442 | NCT04004442-0 | Avelumab | PD-L1 | Bladder cancer |
| NCT04004442 | NCT04004442-0 | Batiraxcept | AXL | Bladder cancer |
| NCT04005144 | NCT04005144-0 | Binimetinib | MEK | lung cancers |
| NCT04005144 | NCT04005144-0 | Brigatinib | ALK/EGFR | lung cancers |
| NCT04006119 | NCT04006119-0 | Ziopharm | gene therapy | Brain and nervous system cancers |
| NCT04006119 | NCT04006119-0 | Veledimex | Other supportive therapy | Brain and nervous system cancers |
| NCT04006119 | NCT04006119-0 | Cemiplimab-Rwlc | PD-1 | Brain and nervous system cancers |
| NCT04015739 | NCT04015739-0 | Bevacizumab | VEGF | Ovary cancer |
| NCT04015739 | NCT04015739-0 | Olaparib | PARP | Ovary cancer |
| NCT04015739 | NCT04015739-0 | Durvalumab | PD-L1 | Ovary cancer |
| NCT04016805 | NCT04016805-1 | Venetoclax | BCL2 | Leukaemia |
| NCT04016805 | NCT04016805-1 | Ublituximab | CD20 | Leukaemia |
| NCT04016805 | NCT04016805-1 | Umbralisib | PI3K | Leukaemia |
| NCT04016805 | NCT04016805-2 | Acalabrutinib | BTK | Leukaemia |
| NCT04016805 | NCT04016805-2 | Ublituximab | CD20 | Leukaemia |
| NCT04016805 | NCT04016805-2 | Umbralisib | PI3K | Leukaemia |
| NCT04016805 | NCT04016805-3 | Ibrutinib | BTK | Leukaemia |
| NCT04016805 | NCT04016805-3 | Ublituximab | CD20 | Leukaemia |
| NCT04016805 | NCT04016805-3 | Umbralisib | PI3K | Leukaemia |
| NCT04017650 | NCT04017650-0 | Encorafenib | RAF | Other solid tumors |
| NCT04017650 | NCT04017650-0 | Cetuximab | EGFR | Other solid tumors |
| NCT04017650 | NCT04017650-0 | Nivolumab | PD-1 | Other solid tumors |
| NCT04019288 | NCT04019288-0 | Batiraxcept | AXL | Ovary cancer |
| NCT04019288 | NCT04019288-0 | Durvalumab | PD-L1 | Ovary cancer |
| NCT04019327 | NCT04019327-0 | Talazoparib | PARP | Prostate cancer |
| NCT04019327 | NCT04019327-0 | Temozolomide | chemotherapy | Prostate cancer |
| NCT04025372 | NCT04025372-0 | Bicalutamide | Endocrine Therapy | Prostate cancer |
| NCT04025372 | NCT04025372-0 | Gnrh Agonist | Endocrine Therapy | Prostate cancer |
| NCT04026412 | NCT04026412-0 | Nivolumab | PD-1 | lung cancers |
| NCT04026412 | NCT04026412-0 | Ipilimumab | CTLA-4 | lung cancers |
| NCT04029428 | NCT04029428-0 | 90Y Dotatate | SSTR | Brain and nervous system cancers |
| NCT04029428 | NCT04029428-0 | Lutetium Lu 177 Dotatate | SSTR | Brain and nervous system cancers |
| NCT04037254 | NCT04037254-0 | Niraparib | PARP | Prostate cancer |
| NCT04037254 | NCT04037254-0 | Gnrh Agonist | Endocrine Therapy | Prostate cancer |
| NCT04039230 | NCT04039230-0 | Sacituzumab Govitecan | Trop-2/TOP1 | Breast cancer |
| NCT04039230 | NCT04039230-0 | Talazoparib | PARP | Breast cancer |
| NCT04042701 | NCT04042701-0 | Trastuzumab Deruxtecan | HER2/TOP1 | Other solid tumors |
| NCT04042701 | NCT04042701-0 | Pembrolizumab | PD-1 | Other solid tumors |
| NCT04044430 | NCT04044430-0 | Encorafenib | RAF | Colon and rectum cancers |
| NCT04044430 | NCT04044430-0 | Binimetinib | MEK | Colon and rectum cancers |
| NCT04044430 | NCT04044430-0 | Nivolumab | PD-1 | Colon and rectum cancers |
| NCT04045795 | NCT04045795-0 | Isatuximab | CD38 | Multiple Myeloma |
| NCT04045795 | NCT04045795-0 | Pomalidomide | CRBN | Multiple Myeloma |
| NCT04045795 | NCT04045795-0 | Dexamethasone | Steroid Treatment | Multiple Myeloma |
| NCT04049227 | NCT04049227-0 | Abemaciclib | CDK4/CDK6 | Corpus uteri cancer |
| NCT04049227 | NCT04049227-0 | Letrozole | Endocrine Therapy | Corpus uteri cancer |
| NCT04050436 | NCT04050436-0 | Cemiplimab-Rwlc | PD-1 | Others |
| NCT04050436 | NCT04050436-0 | Rp1 | oncolytic virus | Others |
| NCT04051996 | NCT04051996-0 | Glasdegib | SMO | Leukaemia |
| NCT04051996 | NCT04051996-0 | Decitabine | chemotherapy | Leukaemia |
| NCT04053322 | NCT04053322-0 | Durvalumab | PD-L1 | Breast cancer |
| NCT04053322 | NCT04053322-0 | Olaparib | PARP | Breast cancer |
| NCT04053322 | NCT04053322-0 | Fulvestrant | Endocrine Therapy | Breast cancer |
| NCT04055493 | NCT04055493-0 | Endocrine Therapy | Endocrine Therapy | Breast cancer |
| NCT04055493 | NCT04055493-0 | Ribociclib | CDK4/CDK6 | Breast cancer |
| NCT04056910 | NCT04056910-0 | Nivolumab | PD-1 | Brain and nervous system cancers |
| NCT04056910 | NCT04056910-0 | Ivosidenib | IDH | Brain and nervous system cancers |
| NCT04061980 | NCT04061980-1 | Encorafenib | RAF | Other solid tumors |
| NCT04061980 | NCT04061980-1 | Nivolumab | PD-1 | Other solid tumors |
| NCT04061980 | NCT04061980-2 | Binimetinib | MEK | Other solid tumors |
| NCT04061980 | NCT04061980-2 | Nivolumab | PD-1 | Other solid tumors |
| NCT04062708 | NCT04062708-0 | Chemotherapy | chemotherapy | lung cancers |
| NCT04062708 | NCT04062708-0 | Durvalumab | PD-L1 | lung cancers |
| NCT04064190 | NCT04064190-0 | Vactosertib | ALK | Bladder cancer |
| NCT04064190 | NCT04064190-0 | Durvalumab | PD-L1 | Bladder cancer |
| NCT04065555 | NCT04065555-0 | Tak-981 | SUMO | Head and Neck Neoplasms |
| NCT04065555 | NCT04065555-0 | Cetuximab | EGFR | Head and Neck Neoplasms |
| NCT04065555 | NCT04065555-0 | Avelumab | PD-L1 | Head and Neck Neoplasms |
| NCT04068194 | NCT04068194-0 | Nedisertib | DNA-PK | Other solid tumors |
| NCT04068194 | NCT04068194-0 | Avelumab | PD-L1 | Other solid tumors |
| NCT04068610 | NCT04068610-1 | Folfox | chemotherapy | Colon and rectum cancers |
| NCT04068610 | NCT04068610-1 | Bevacizumab | VEGF | Colon and rectum cancers |
| NCT04068610 | NCT04068610-2 | Folfox | chemotherapy | Colon and rectum cancers |
| NCT04068610 | NCT04068610-2 | Bevacizumab | VEGF | Colon and rectum cancers |
| NCT04068610 | NCT04068610-2 | Durvalumab | PD-L1 | Colon and rectum cancers |
| NCT04068610 | NCT04068610-2 | Oleclumab | CD73 | Colon and rectum cancers |
| NCT04068753 | NCT04068753-0 | Niraparib | PARP | Cervix uteri cancer |
| NCT04068753 | NCT04068753-0 | Dostarlimab-Gxly | PD-1 | Cervix uteri cancer |
| NCT04068831 | NCT04068831-0 | Avelumab | PD-L1 | Kidney cancer |
| NCT04068831 | NCT04068831-0 | Talazoparib | PARP | Kidney cancer |
| NCT04071236 | NCT04071236-0 | Nedisertib | DNA-PK | Prostate cancer |
| NCT04071236 | NCT04071236-0 | Avelumab | PD-L1 | Prostate cancer |
| NCT04071262 | NCT04071262-0 | Abemaciclib | CDK4/CDK6 | Others |
| NCT04071262 | NCT04071262-0 | Abiraterone | CYP17A1 | Others |
| NCT04071262 | NCT04071262-0 | Prednisolone | Steroid Treatment | Others |
| NCT04074096 | NCT04074096-0 | Binimetinib | MEK | Melanoma |
| NCT04074096 | NCT04074096-0 | Encorafenib | RAF | Melanoma |
| NCT04074785 | NCT04074785-0 | Abemaciclib | CDK4/CDK6 | Brain and nervous system cancers |
| NCT04074785 | NCT04074785-0 | Bevacizumab | VEGF | Brain and nervous system cancers |
| NCT04075604 | NCT04075604-0 | Nivolumab | PD-1 | Breast cancer |
| NCT04075604 | NCT04075604-0 | Palbociclib | CDK4/CDK6 | Breast cancer |
| NCT04075604 | NCT04075604-0 | Anastrozole | Endocrine Therapy | Breast cancer |
| NCT04075747 | NCT04075747-1 | Venetoclax | BCL2 | Leukaemia |
| NCT04075747 | NCT04075747-1 | Cytarabine | chemotherapy | Leukaemia |
| NCT04075747 | NCT04075747-1 | Daunorubicin | chemotherapy | Leukaemia |
| NCT04075747 | NCT04075747-2 | Midostaurin | PKC/PDGFR | Leukaemia |
| NCT04075747 | NCT04075747-2 | Cytarabine | chemotherapy | Leukaemia |
| NCT04075747 | NCT04075747-2 | Daunorubicin | chemotherapy | Leukaemia |
| NCT04075747 | NCT04075747-3 | Enasidenib | IDH | Leukaemia |
| NCT04075747 | NCT04075747-3 | Cytarabine | chemotherapy | Leukaemia |
| NCT04075747 | NCT04075747-3 | Daunorubicin | chemotherapy | Leukaemia |
| NCT04077463 | NCT04077463-1 | Lazertinib | EGFR | lung cancers |
| NCT04077463 | NCT04077463-1 | Amivantamab | EGFR/MET | lung cancers |
| NCT04077463 | NCT04077463-2 | Lazertinib | EGFR | lung cancers |
| NCT04077463 | NCT04077463-2 | Amivantamab | EGFR/MET | lung cancers |
| NCT04077463 | NCT04077463-2 | Platinum-Based Chemotherapy | chemotherapy | lung cancers |
| NCT04077463 | NCT04077463-2 | Pemetrexed | chemotherapy | lung cancers |
| NCT04083365 | NCT04083365-0 | Capecitabine | chemotherapy | Colon and rectum cancers |
| NCT04083365 | NCT04083365-0 | Durvalumab | PD-L1 | Colon and rectum cancers |
| NCT04083898 | NCT04083898-0 | Isatuximab | CD38 | Multiple Myeloma |
| NCT04083898 | NCT04083898-0 | Bendamustine | chemotherapy | Multiple Myeloma |
| NCT04083898 | NCT04083898-0 | Prednisone | Steroid Treatment | Multiple Myeloma |
| NCT04086485 | NCT04086485-0 | Lutetium Lu 177 Dotatate | SSTR | Brain and nervous system cancers |
| NCT04086485 | NCT04086485-0 | Olaparib | PARP | Brain and nervous system cancers |
| NCT04088188 | NCT04088188-1 | Gemcitabine | chemotherapy | Other solid tumors |
| NCT04088188 | NCT04088188-1 | Platinum-Based Chemotherapy | chemotherapy | Other solid tumors |
| NCT04088188 | NCT04088188-1 | Ivosidenib | IDH | Other solid tumors |
| NCT04088188 | NCT04088188-2 | Gemcitabine | chemotherapy | Other solid tumors |
| NCT04088188 | NCT04088188-2 | Platinum-Based Chemotherapy | chemotherapy | Other solid tumors |
| NCT04088188 | NCT04088188-2 | Pemigatinib | FGFR | Other solid tumors |
| NCT04089553 | NCT04089553-1 | Imaradenant | A2aR/A2bR | Prostate cancer |
| NCT04089553 | NCT04089553-1 | Durvalumab | PD-L1 | Prostate cancer |
| NCT04089553 | NCT04089553-2 | Imaradenant | A2aR/A2bR | Prostate cancer |
| NCT04089553 | NCT04089553-2 | Durvalumab | PD-L1 | Prostate cancer |
| NCT04089553 | NCT04089553-2 | Oleclumab | CD73 | Prostate cancer |
| NCT04091126 | NCT04091126-0 | Lenalidomide | CRBN | Multiple Myeloma |
| NCT04091126 | NCT04091126-0 | Belantamab Mafodotin | BCMA/Tubulin | Multiple Myeloma |
| NCT04091126 | NCT04091126-0 | Bortezomib | PSMB | Multiple Myeloma |
| NCT04091126 | NCT04091126-0 | Dexamethasone | Steroid Treatment | Multiple Myeloma |
| NCT04092179 | NCT04092179-0 | Enasidenib | IDH | Leukaemia |
| NCT04092179 | NCT04092179-0 | Venetoclax | BCL2 | Leukaemia |
| NCT04093505 | NCT04093505-0 | Gemtuzumab Ozogamicin | CD33/DNA | Leukaemia |
| NCT04093505 | NCT04093505-0 | Glasdegib | SMO | Leukaemia |
| NCT04094142 | NCT04094142-0 | Acalabrutinib | BTK | Lymphomas |
| NCT04094142 | NCT04094142-0 | Rituximab | CD20 | Lymphomas |
| NCT04094142 | NCT04094142-0 | Lenalidomide | CRBN | Lymphomas |
| NCT04097470 | NCT04097470-0 | Decitabine | chemotherapy | Lymphomas |
| NCT04097470 | NCT04097470-0 | Midostaurin | PKC/PDGFR | Lymphomas |
| NCT04105270 | NCT04105270-0 | Durvalumab | PD-L1 | lung cancers |
| NCT04105270 | NCT04105270-0 | Chemotherapy | chemotherapy | lung cancers |
| NCT04106115 | NCT04106115-1 | Durvalumab | PD-L1 | Bladder cancer |
| NCT04106115 | NCT04106115-1 | S-488210 | vaccine | Bladder cancer |
| NCT04106115 | NCT04106115-2 | Durvalumab | PD-L1 | Bladder cancer |
| NCT04106115 | NCT04106115-2 | S-488211Vaccine | vaccine | Bladder cancer |
| NCT04108208 | NCT04108208-0 | Apalutamide | Endocrine Therapy | Prostate cancer |
| NCT04108208 | NCT04108208-0 | Androgen Deprivation Therapy (Adt) | Endocrine Therapy | Prostate cancer |
| NCT04108858 | NCT04108858-0 | Copanlisib | PI3K | Breast cancer |
| NCT04108858 | NCT04108858-0 | Trastuzumab | HER2 | Breast cancer |
| NCT04108858 | NCT04108858-0 | Pertuzumab | HER2 | Breast cancer |
| NCT04109456 | NCT04109456-0 | In10018 | FAK | Melanoma |
| NCT04109456 | NCT04109456-0 | Cobimetinib | MEK | Melanoma |
| NCT04115631 | NCT04115631-1 | Acalabrutinib | BTK | Lymphomas |
| NCT04115631 | NCT04115631-1 | Bendamustine | chemotherapy | Lymphomas |
| NCT04115631 | NCT04115631-1 | Rituximab | CD20 | Lymphomas |
| NCT04115631 | NCT04115631-1 | Cytarabine | chemotherapy | Lymphomas |
| NCT04115631 | NCT04115631-2 | Acalabrutinib | BTK | Lymphomas |
| NCT04115631 | NCT04115631-2 | Bendamustine | chemotherapy | Lymphomas |
| NCT04115631 | NCT04115631-2 | Rituximab | CD20 | Lymphomas |
| NCT04116047 | NCT04116047-0 | Durvalumab | PD-L1 | Mouth and oropharynx cancers |
| NCT04116047 | NCT04116047-0 | Cisplatin | chemotherapy | Mouth and oropharynx cancers |
| NCT04116541 | NCT04116541-0 | Siremadlin | p53/MDM2 | Other solid tumors |
| NCT04116541 | NCT04116541-0 | Ribociclib | CDK4/CDK6 | Other solid tumors |
| NCT04126200 | NCT04126200-1 | Belantamab Mafodotin | BCMA/Tubulin | Multiple Myeloma |
| NCT04126200 | NCT04126200-1 | Gsk3174998 | OX40 | Multiple Myeloma |
| NCT04126200 | NCT04126200-2 | Belantamab Mafodotin | BCMA/Tubulin | Multiple Myeloma |
| NCT04126200 | NCT04126200-2 | Feladilimab | ICOS | Multiple Myeloma |
| NCT04126200 | NCT04126200-3 | Belantamab Mafodotin | BCMA/Tubulin | Multiple Myeloma |
| NCT04126200 | NCT04126200-3 | Nirogacestat | gamma secretase | Multiple Myeloma |
| NCT04126200 | NCT04126200-4 | Belantamab Mafodotin | BCMA/Tubulin | Multiple Myeloma |
| NCT04126200 | NCT04126200-4 | Dostarlimab-Gxly | PD-1 | Multiple Myeloma |
| NCT04126200 | NCT04126200-5 | Belantamab Mafodotin | BCMA/Tubulin | Multiple Myeloma |
| NCT04126200 | NCT04126200-5 | Isatuximab | CD38 | Multiple Myeloma |
| NCT04132505 | NCT04132505-0 | Binimetinib | MEK | Pancreas cancer |
| NCT04132505 | NCT04132505-0 | Hydroxychloroquine | antimalarial agent | Pancreas cancer |
| NCT04134260 | NCT04134260-0 | Apalutamide | Endocrine Therapy | Other solid tumors |
| NCT04134260 | NCT04134260-0 | Abiraterone | CYP17A1 | Other solid tumors |
| NCT04134260 | NCT04134260-0 | Prednisone | Steroid Treatment | Other solid tumors |
| NCT04134884 | NCT04134884-0 | Astx727(Decitabine/Cedazuridine) | chemotherapy | Breast cancer |
| NCT04134884 | NCT04134884-0 | Talazoparib | PARP | Breast cancer |
| NCT04134936 | NCT04134936-0 | Tafasitamab | CD19 | Lymphomas |
| NCT04134936 | NCT04134936-0 | Lenalidomide | CRBN | Lymphomas |
| NCT04134936 | NCT04134936-0 | Rituximab | CD20 | Lymphomas |
| NCT04134936 | NCT04134936-0 | Chemotherapy | chemotherapy | Lymphomas |
| NCT04136353 | NCT04136353-0 | Darolutamide | Endocrine Therapy | Prostate cancer |
| NCT04136353 | NCT04136353-0 | Lhrha | Endocrine Therapy | Prostate cancer |
| NCT04138381 | NCT04138381-0 | Selinexor | XPO1 | Other solid tumors |
| NCT04138381 | NCT04138381-0 | Imatinib | BCR-ABL | Other solid tumors |
| NCT04139317 | NCT04139317-0 | Capmatinib | MET | lung cancers |
| NCT04139317 | NCT04139317-0 | Pembrolizumab | PD-1 | lung cancers |
| NCT04139902 | NCT04139902-0 | Dostarlimab-Gxly | PD-1 | Melanoma |
| NCT04139902 | NCT04139902-0 | Cobolimab | TIM3 | Melanoma |
| NCT04140487 | NCT04140487-0 | Azacitidine | chemotherapy | Leukaemia |
| NCT04140487 | NCT04140487-0 | Venetoclax | BCL2 | Leukaemia |
| NCT04140487 | NCT04140487-0 | Gilteritinib | FLT3 | Leukaemia |
| NCT04149145 | NCT04149145-0 | M4344 | ATR | Ovary cancer |
| NCT04149145 | NCT04149145-0 | Niraparib | PARP | Ovary cancer |
| NCT04149821 | NCT04149821-0 | Umbralisib | PI3K | Leukaemia |
| NCT04149821 | NCT04149821-0 | Ublituximab | CD20 | Leukaemia |
| NCT04150562 | NCT04150562-0 | Avelumab | PD-L1 | Kidney cancer |
| NCT04150562 | NCT04150562-0 | Il-15 | IL15 | Kidney cancer |
| NCT04156087 | NCT04156087-0 | Durvalumab | PD-L1 | Pancreas cancer |
| NCT04156087 | NCT04156087-0 | Tremelimumab | CTLA-4 | Pancreas cancer |
| NCT04156828 | NCT04156828-0 | Copanlisib | PI3K | Lymphomas |
| NCT04156828 | NCT04156828-0 | Rituximab | CD20 | Lymphomas |
| NCT04156828 | NCT04156828-0 | Chemotherapy | chemotherapy | Lymphomas |
| NCT04158336 | NCT04158336-0 | Zn-C3 | WEE | Other solid tumors |
| NCT04158336 | NCT04158336-0 | Talazoparib | PARP | Other solid tumors |
| NCT04158362 | NCT04158362-0 | Letrozole | Endocrine Therapy | Others |
| NCT04158362 | NCT04158362-0 | Anastrozole | Endocrine Therapy | Others |
| NCT04158362 | NCT04158362-0 | Fulvestrant | Endocrine Therapy | Others |
| NCT04158362 | NCT04158362-0 | Abemaciclib | CDK4/CDK6 | Others |
| NCT04159155 | NCT04159155-0 | Niraparib | PARP | Corpus uteri cancer |
| NCT04159155 | NCT04159155-0 | Carboplatin | chemotherapy | Corpus uteri cancer |
| NCT04159155 | NCT04159155-0 | Paclitaxel | chemotherapy | Corpus uteri cancer |
| NCT04159974 | NCT04159974-0 | Durvalumab | PD-L1 | Other solid tumors |
| NCT04159974 | NCT04159974-0 | Tremelimumab | CTLA-4 | Other solid tumors |
| NCT04163432 | NCT04163432-0 | Durvalumab | PD-L1 | lung cancers |
| NCT04163432 | NCT04163432-0 | Platinum-Based Chemotherapy | chemotherapy | lung cancers |
| NCT04163432 | NCT04163432-0 | Pemetrexed | chemotherapy | lung cancers |
| NCT04165772 | NCT04165772-1 | Dostarlimab-Gxly | PD-1 | Colon and rectum cancers |
| NCT04165772 | NCT04165772-1 | Capecitabine | chemotherapy | Colon and rectum cancers |
| NCT04165772 | NCT04165772-2 | Dostarlimab-Gxly | PD-1 | Colon and rectum cancers |
| NCT04165772 | NCT04165772-2 | 5-Fu | chemotherapy | Colon and rectum cancers |
| NCT04168502 | NCT04168502-0 | Gemtuzumab Ozogamicin | CD33/DNA | Leukaemia |
| NCT04168502 | NCT04168502-0 | Glasdegib | SMO | Leukaemia |
| NCT04169074 | NCT04169074-0 | Abemaciclib | CDK4/CDK6 | Head and Neck Neoplasms |
| NCT04169074 | NCT04169074-0 | Nivolumab | PD-1 | Head and Neck Neoplasms |
| NCT04169737 | NCT04169737-0 | Acalabrutinib | BTK | Leukaemia |
| NCT04169737 | NCT04169737-0 | Venetoclax | BCL2 | Leukaemia |
| NCT04169737 | NCT04169737-0 | Obinutuzumab | CD20 | Leukaemia |
| NCT04169841 | NCT04169841-0 | Olaparib | PARP | Other solid tumors |
| NCT04169841 | NCT04169841-0 | Durvalumab | PD-L1 | Other solid tumors |
| NCT04169841 | NCT04169841-0 | Tremelimumab | CTLA-4 | Other solid tumors |
| NCT04170153 | NCT04170153-0 | Niraparib | PARP | Others |
| NCT04170153 | NCT04170153-0 | M1774 | ATR | Others |
| NCT04170283 | NCT04170283-0 | Zanubrutinib | BTK | Lymphomas |
| NCT04170283 | NCT04170283-0 | Tislelizumab | PD-1 | Lymphomas |
| NCT04173507 | NCT04173507-0 | Avelumab | PD-L1 | lung cancers |
| NCT04173507 | NCT04173507-0 | Talazoparib | PARP | lung cancers |
| NCT04174612 | NCT04174612-0 | Cytarabine | chemotherapy | Leukaemia |
| NCT04174612 | NCT04174612-0 | Daunorubicin | chemotherapy | Leukaemia |
| NCT04174612 | NCT04174612-0 | Midostaurin | PKC/PDGFR | Leukaemia |
| NCT04176081 | NCT04176081-0 | Darolutamide | Endocrine Therapy | Prostate cancer |
| NCT04176081 | NCT04176081-0 | Degarelix | Endocrine Therapy | Prostate cancer |
| NCT04176848 | NCT04176848-0 | Cfi-400945 | PLK4 | Breast cancer |
| NCT04176848 | NCT04176848-0 | Durvalumab | PD-L1 | Breast cancer |
| NCT04177810 | NCT04177810-0 | Cemiplimab-Rwlc | PD-1 | Pancreas cancer |
| NCT04177810 | NCT04177810-0 | Plerixafor | CXCR4 | Pancreas cancer |
| NCT04178460 | NCT04178460-0 | Niraparib | PARP | Other solid tumors |
| NCT04178460 | NCT04178460-0 | Mgd013 | PD-1/LAG-3 | Other solid tumors |
| NCT04179864 | NCT04179864-1 | Tazemetostat | EZH2 | Prostate cancer |
| NCT04179864 | NCT04179864-1 | Abiraterone | CYP17A1 | Prostate cancer |
| NCT04179864 | NCT04179864-2 | Tazemetostat | EZH2 | Prostate cancer |
| NCT04179864 | NCT04179864-2 | Prednisone | Steroid Treatment | Prostate cancer |
| NCT04179864 | NCT04179864-3 | Tazemetostat | EZH2 | Prostate cancer |
| NCT04179864 | NCT04179864-3 | Enzalutamide | Endocrine Therapy | Prostate cancer |
| NCT04181203 | NCT04181203-0 | Lhrha | Endocrine Therapy | Prostate cancer |
| NCT04181203 | NCT04181203-0 | Apalutamide | Endocrine Therapy | Prostate cancer |
| NCT04182204 | NCT04182204-0 | Polatuzumab Vedotin | CD79B/Tubulin | Lymphomas |
| NCT04182204 | NCT04182204-0 | Rituximab | CD20 | Lymphomas |
| NCT04182204 | NCT04182204-0 | Gemcitabine | chemotherapy | Lymphomas |
| NCT04182204 | NCT04182204-0 | Platinum-Based Chemotherapy | chemotherapy | Lymphomas |
| NCT04185220 | NCT04185220-0 | Rhil-15 | IL15 | Leukaemia |
| NCT04185220 | NCT04185220-0 | Mogamulizumab | CCR4 | Leukaemia |
| NCT04185883 | NCT04185883-1 | Sotorasib | KRAS | Other solid tumors |
| NCT04185883 | NCT04185883-1 | Trametinib | MEK | Other solid tumors |
| NCT04185883 | NCT04185883-10 | Sotorasib | KRAS | Other solid tumors |
| NCT04185883 | NCT04185883-10 | Everolimus | mTOR | Other solid tumors |
| NCT04185883 | NCT04185883-11 | Sotorasib | KRAS | Other solid tumors |
| NCT04185883 | NCT04185883-11 | Trametinib | MEK | Other solid tumors |
| NCT04185883 | NCT04185883-11 | Panitumumab | EGFR | Other solid tumors |
| NCT04185883 | NCT04185883-12 | Sotorasib | KRAS | Other solid tumors |
| NCT04185883 | NCT04185883-12 | Bevacizumab | VEGF | Other solid tumors |
| NCT04185883 | NCT04185883-12 | Folfiri | chemotherapy | Other solid tumors |
| NCT04185883 | NCT04185883-13 | Sotorasib | KRAS | Other solid tumors |
| NCT04185883 | NCT04185883-13 | Bevacizumab | VEGF | Other solid tumors |
| NCT04185883 | NCT04185883-13 | Folfox | chemotherapy | Other solid tumors |
| NCT04185883 | NCT04185883-14 | Sotorasib | KRAS | Other solid tumors |
| NCT04185883 | NCT04185883-14 | Tno155 | SHP2 | Other solid tumors |
| NCT04185883 | NCT04185883-15 | Sotorasib | KRAS | Other solid tumors |
| NCT04185883 | NCT04185883-15 | Afatinib | EGFR | Other solid tumors |
| NCT04185883 | NCT04185883-15 | Loperamide | Other supportive therapy | Other solid tumors |
| NCT04185883 | NCT04185883-2 | Sotorasib | KRAS | Other solid tumors |
| NCT04185883 | NCT04185883-2 | Amg404 | PD-1 | Other solid tumors |
| NCT04185883 | NCT04185883-3 | Sotorasib | KRAS | Other solid tumors |
| NCT04185883 | NCT04185883-3 | Rmc-4630 | SHP2 | Other solid tumors |
| NCT04185883 | NCT04185883-4 | Sotorasib | KRAS | Other solid tumors |
| NCT04185883 | NCT04185883-4 | Afatinib | EGFR | Other solid tumors |
| NCT04185883 | NCT04185883-5 | Sotorasib | KRAS | Other solid tumors |
| NCT04185883 | NCT04185883-5 | Pembrolizumab | PD-1 | Other solid tumors |
| NCT04185883 | NCT04185883-6 | Sotorasib | KRAS | Other solid tumors |
| NCT04185883 | NCT04185883-6 | Panitumumab | EGFR | Other solid tumors |
| NCT04185883 | NCT04185883-6 | Folfiri | chemotherapy | Other solid tumors |
| NCT04185883 | NCT04185883-7 | Sotorasib | KRAS | Other solid tumors |
| NCT04185883 | NCT04185883-7 | Atezolizumab | PD-L1 | Other solid tumors |
| NCT04185883 | NCT04185883-8 | Sotorasib | KRAS | Other solid tumors |
| NCT04185883 | NCT04185883-8 | Platinum-Based Chemotherapy | chemotherapy | Other solid tumors |
| NCT04185883 | NCT04185883-8 | Pemetrexed | chemotherapy | Other solid tumors |
| NCT04185883 | NCT04185883-8 | Docetaxel | chemotherapy | Other solid tumors |
| NCT04185883 | NCT04185883-8 | Pembrolizumab | PD-1 | Other solid tumors |
| NCT04185883 | NCT04185883-9 | Sotorasib | KRAS | Other solid tumors |
| NCT04185883 | NCT04185883-9 | Palbociclib | CDK4/CDK6 | Other solid tumors |
| NCT04187833 | NCT04187833-0 | Nivolumab | PD-1 | Melanoma |
| NCT04187833 | NCT04187833-0 | Talazoparib | PARP | Melanoma |
| NCT04188119 | NCT04188119-1 | Avelumab | PD-L1 | Breast cancer |
| NCT04188119 | NCT04188119-1 | Lansoprazole | Other supportive therapy | Breast cancer |
| NCT04188119 | NCT04188119-2 | Avelumab | PD-L1 | Breast cancer |
| NCT04188119 | NCT04188119-2 | Aspirin | PTGS | Breast cancer |
| NCT04188119 | NCT04188119-2 | Lansoprazole | Other supportive therapy | Breast cancer |
| NCT04188548 | NCT04188548-1 | Ly3484356 | Endocrine Therapy | Other solid tumors |
| NCT04188548 | NCT04188548-1 | Abemaciclib | CDK4/CDK6 | Other solid tumors |
| NCT04188548 | NCT04188548-1 | Aromatase Inhibitor | Endocrine Therapy | Other solid tumors |
| NCT04188548 | NCT04188548-2 | Ly3484356 | Endocrine Therapy | Other solid tumors |
| NCT04188548 | NCT04188548-2 | Alpelisib | PI3K | Other solid tumors |
| NCT04188548 | NCT04188548-3 | Ly3484356 | Endocrine Therapy | Other solid tumors |
| NCT04188548 | NCT04188548-3 | Trastuzumab | HER2 | Other solid tumors |
| NCT04188548 | NCT04188548-3 | Abemaciclib | CDK4/CDK6 | Other solid tumors |
| NCT04188548 | NCT04188548-4 | Ly3484356 | Endocrine Therapy | Other solid tumors |
| NCT04188548 | NCT04188548-4 | Abemaciclib | CDK4/CDK6 | Other solid tumors |
| NCT04189952 | NCT04189952-0 | Acalabrutinib | BTK | Leukaemia |
| NCT04189952 | NCT04189952-0 | Rituximab | CD20 | Leukaemia |
| NCT04189952 | NCT04189952-0 | Chemotherapy | chemotherapy | Leukaemia |
| NCT04194554 | NCT04194554-0 | Niraparib | PARP | Prostate cancer |
| NCT04194554 | NCT04194554-0 | Leuprolide | Endocrine Therapy | Prostate cancer |
| NCT04194554 | NCT04194554-0 | Abiraterone | CYP17A1 | Prostate cancer |
| NCT04194944 | NCT04194944-0 | Pemetrexed | chemotherapy | lung cancers |
| NCT04194944 | NCT04194944-0 | Pembrolizumab | PD-1 | lung cancers |
| NCT04204941 | NCT04204941-0 | Tazemetostat | EZH2 | Other solid tumors |
| NCT04204941 | NCT04204941-0 | Doxorubicin | chemotherapy | Other solid tumors |
| NCT04207190 | NCT04207190-0 | Talazoparib | PARP | Leukaemia |
| NCT04207190 | NCT04207190-0 | Gemtuzumab Ozogamicin | CD33/DNA | Leukaemia |
| NCT04208178 | NCT04208178-0 | Alpelisib | PI3K | Breast cancer |
| NCT04208178 | NCT04208178-0 | Trastuzumab | HER2 | Breast cancer |
| NCT04208178 | NCT04208178-0 | Pertuzumab | HER2 | Breast cancer |
| NCT04209621 | NCT04209621-0 | Duvelisib | PI3K | Leukaemia |
| NCT04209621 | NCT04209621-0 | Ibrutinib | BTK | Leukaemia |
| NCT04213404 | NCT04213404-0 | Ribociclib | CDK4/CDK6 | Head and Neck Neoplasms |
| NCT04213404 | NCT04213404-0 | Spartalizumab | PD-1 | Head and Neck Neoplasms |
| NCT04215146 | NCT04215146-0 | Pelareorep | oncolytic virus | Breast cancer |
| NCT04215146 | NCT04215146-0 | Paclitaxel | chemotherapy | Breast cancer |
| NCT04215146 | NCT04215146-0 | Avelumab | PD-L1 | Breast cancer |
| NCT04216290 | NCT04216290-1 | Durvalumab | PD-L1 | Gallbladder and biliary tract cancer |
| NCT04216290 | NCT04216290-1 | Gemcitabine | chemotherapy | Gallbladder and biliary tract cancer |
| NCT04216290 | NCT04216290-1 | Cisplatin | chemotherapy | Gallbladder and biliary tract cancer |
| NCT04216290 | NCT04216290-2 | Durvalumab | PD-L1 | Gallbladder and biliary tract cancer |
| NCT04216290 | NCT04216290-2 | Mitomycin | chemotherapy | Gallbladder and biliary tract cancer |
| NCT04216290 | NCT04216290-2 | 5-Fu | chemotherapy | Gallbladder and biliary tract cancer |
| NCT04216316 | NCT04216316-0 | Pembrolizumab | PD-1 | lung cancers |
| NCT04216316 | NCT04216316-0 | Gemcitabine | chemotherapy | lung cancers |
| NCT04216316 | NCT04216316-0 | Carboplatin | chemotherapy | lung cancers |
| NCT04216316 | NCT04216316-0 | Berzosertib | ATR | lung cancers |
| NCT04216329 | NCT04216329-0 | Selinexor | XPO1 | Brain and nervous system cancers |
| NCT04216329 | NCT04216329-0 | Temozolomide | chemotherapy | Brain and nervous system cancers |
| NCT04216472 | NCT04216472-0 | Alpelisib | PI3K | Breast cancer |
| NCT04216472 | NCT04216472-0 | Paclitaxel | chemotherapy | Breast cancer |
| NCT04216524 | NCT04216524-0 | Tagraxofusp-Erzs | CD123 | Lymphomas |
| NCT04216524 | NCT04216524-0 | Venetoclax | BCL2 | Lymphomas |
| NCT04216524 | NCT04216524-0 | Chemotherapy | chemotherapy | Lymphomas |
| NCT04216563 | NCT04216563-1 | Asciminib | BCR-ABL | Leukaemia |
| NCT04216563 | NCT04216563-1 | Dasatinib | BCR-ABL | Leukaemia |
| NCT04216563 | NCT04216563-2 | Asciminib | BCR-ABL | Leukaemia |
| NCT04216563 | NCT04216563-2 | Nilotinib | BCR-ABL | Leukaemia |
| NCT04217798 | NCT04217798-0 | Niraparib | PARP | Ovary cancer |
| NCT04217798 | NCT04217798-0 | Etoposide | chemotherapy | Ovary cancer |
| NCT04221438 | NCT04221438-0 | Binimetinib | MEK | Others |
| NCT04221438 | NCT04221438-0 | Encorafenib | RAF | Others |
| NCT04221555 | NCT04221555-0 | Durvalumab | PD-L1 | Stomach cancer |
| NCT04221555 | NCT04221555-0 | Docetaxel | chemotherapy | Stomach cancer |
| NCT04221555 | NCT04221555-0 | Platinum-Based Chemotherapy | chemotherapy | Stomach cancer |
| NCT04221555 | NCT04221555-0 | S-1 | chemotherapy | Stomach cancer |
| NCT04223648 | NCT04223648-0 | Durvalumab | PD-L1 | Melanoma |
| NCT04223648 | NCT04223648-0 | Tremelimumab | CTLA-4 | Melanoma |
| NCT04224337 | NCT04224337-0 | Durvalumab | PD-L1 | lung cancers |
| NCT04224337 | NCT04224337-0 | Doxorubicin | chemotherapy | lung cancers |
| NCT04224337 | NCT04224337-0 | Ifosfamide | Chemotherapy | lung cancers |
| NCT04224493 | NCT04224493-0 | Tazemetostat | EZH2 | Lymphomas |
| NCT04224493 | NCT04224493-0 | Lenalidomide | CRBN | Lymphomas |
| NCT04224493 | NCT04224493-0 | Rituximab | CD20 | Lymphomas |
| NCT04227028 | NCT04227028-0 | Brigatinib | ALK/EGFR | lung cancers |
| NCT04227028 | NCT04227028-0 | Bevacizumab | VEGF | lung cancers |
| NCT04227327 | NCT04227327-0 | Abemaciclib | CDK4/CDK6 | Breast cancer |
| NCT04227327 | NCT04227327-0 | Aromatase Inhibitor | Endocrine Therapy | Breast cancer |
| NCT04230109 | NCT04230109-0 | Sacituzumab Govitecan | Trop-2/TOP1 | Breast cancer |
| NCT04230109 | NCT04230109-0 | Pembrolizumab | PD-1 | Breast cancer |
| NCT04230408 | NCT04230408-0 | Durvalumab | PD-L1 | lung cancers |
| NCT04230408 | NCT04230408-0 | Platinum-Based Chemotherapy | chemotherapy | lung cancers |
| NCT04230408 | NCT04230408-0 | Paclitaxel | chemotherapy | lung cancers |
| NCT04230759 | NCT04230759-0 | 5-Fu | chemotherapy | Other solid tumors |
| NCT04230759 | NCT04230759-0 | Mitomycin | chemotherapy | Other solid tumors |
| NCT04230759 | NCT04230759-0 | Durvalumab | PD-L1 | Other solid tumors |
| NCT04231851 | NCT04231851-0 | Glasdegib | SMO | Leukaemia |
| NCT04231851 | NCT04231851-0 | Cytarabine | chemotherapy | Leukaemia |
| NCT04231851 | NCT04231851-0 | Daunorubicin | chemotherapy | Leukaemia |
| NCT04231877 | NCT04231877-0 | Polatuzumab Vedotin | CD79B/Tubulin | Lymphomas |
| NCT04231877 | NCT04231877-0 | Chemotherapy | chemotherapy | Lymphomas |
| NCT04234568 | NCT04234568-0 | Triapine | RRM1/RRM2 | Brain and nervous system cancers |
| NCT04234568 | NCT04234568-0 | Lutetium Lu 177 Dotatate | SSTR | Brain and nervous system cancers |
| NCT04235101 | NCT04235101-0 | Syd985 | HER2/DNA | Other solid tumors |
| NCT04235101 | NCT04235101-0 | Niraparib | PARP | Other solid tumors |
| NCT04236141 | NCT04236141-0 | Polatuzumab Vedotin | CD79B/Tubulin | Lymphomas |
| NCT04236141 | NCT04236141-0 | Bendamustine | chemotherapy | Lymphomas |
| NCT04236141 | NCT04236141-0 | Rituximab | CD20 | Lymphomas |
| NCT04238624 | NCT04238624-0 | Cemiplimab-Rwlc | PD-1 | Other solid tumors |
| NCT04238624 | NCT04238624-0 | Dabrafenib | RAF | Other solid tumors |
| NCT04238624 | NCT04238624-0 | Trametinib | MEK | Other solid tumors |
| NCT04238637 | NCT04238637-0 | Durvalumab | PD-L1 | Gallbladder and biliary tract cancer |
| NCT04238637 | NCT04238637-0 | Tremelimumab | CTLA-4 | Gallbladder and biliary tract cancer |
| NCT04238819 | NCT04238819-1 | Abemaciclib | CDK4/CDK6 | Other solid tumors |
| NCT04238819 | NCT04238819-1 | Irinotecan | chemotherapy | Other solid tumors |
| NCT04238819 | NCT04238819-1 | Temozolomide | chemotherapy | Other solid tumors |
| NCT04238819 | NCT04238819-2 | Abemaciclib | CDK4/CDK6 | Other solid tumors |
| NCT04238819 | NCT04238819-2 | Temozolomide | chemotherapy | Other solid tumors |
| NCT04240002 | NCT04240002-0 | Gilteritinib | FLT3 | Leukaemia |
| NCT04240002 | NCT04240002-0 | Chemotherapy | chemotherapy | Leukaemia |
| NCT04240054 | NCT04240054-0 | Bortezomib | PSMB | Multiple Myeloma |
| NCT04240054 | NCT04240054-0 | Isatuximab | CD38 | Multiple Myeloma |
| NCT04240054 | NCT04240054-0 | Cyclophosphamide | chemotherapy | Multiple Myeloma |
| NCT04240054 | NCT04240054-0 | Dexamethasone | Steroid Treatment | Multiple Myeloma |
| NCT04240106 | NCT04240106-0 | Niraparib | PARP | Breast cancer |
| NCT04240106 | NCT04240106-0 | Aromatase Inhibitor | Endocrine Therapy | Breast cancer |
| NCT04242238 | NCT04242238-0 | Vimseltinib | CSF1R | Other solid tumors |
| NCT04242238 | NCT04242238-0 | Avelumab | PD-L1 | Other solid tumors |
| NCT04243616 | NCT04243616-0 | Cemiplimab-Rwlc | PD-1 | Breast cancer |
| NCT04243616 | NCT04243616-0 | Paclitaxel | chemotherapy | Breast cancer |
| NCT04243616 | NCT04243616-0 | Carboplatin | chemotherapy | Breast cancer |
| NCT04243616 | NCT04243616-0 | Doxorubicin | chemotherapy | Breast cancer |
| NCT04243616 | NCT04243616-0 | Cyclophosphamide | chemotherapy | Breast cancer |
| NCT04246047 | NCT04246047-0 | Belantamab Mafodotin | BCMA/Tubulin | Multiple Myeloma |
| NCT04246047 | NCT04246047-0 | Bortezomib | PSMB | Multiple Myeloma |
| NCT04246047 | NCT04246047-0 | Dexamethasone | Steroid Treatment | Multiple Myeloma |
| NCT04250051 | NCT04250051-0 | Chemotherapy | chemotherapy | Other hematologic Neoplasms |
| NCT04250051 | NCT04250051-0 | Ivosidenib | IDH | Other hematologic Neoplasms |
| NCT04251533 | NCT04251533-0 | Alpelisib | PI3K | Breast cancer |
| NCT04251533 | NCT04251533-0 | Paclitaxel | chemotherapy | Breast cancer |
| NCT04253145 | NCT04253145-0 | Atezolizumab | PD-L1 | lung cancers |
| NCT04253145 | NCT04253145-0 | Lurbinectedin | chemotherapy | lung cancers |
| NCT04253262 | NCT04253262-0 | Copanlisib | PI3K | Prostate cancer |
| NCT04253262 | NCT04253262-0 | Rucaparib | PARP | Prostate cancer |
| NCT04255836 | NCT04255836-0 | Durvalumab | PD-L1 | lung cancers |
| NCT04255836 | NCT04255836-0 | Chemotherapy | chemotherapy | lung cancers |
| NCT04256707 | NCT04256707-1 | Selinexor | XPO1 | Other solid tumors |
| NCT04256707 | NCT04256707-1 | Docetaxel | chemotherapy | Other solid tumors |
| NCT04256707 | NCT04256707-2 | Selinexor | XPO1 | Other solid tumors |
| NCT04256707 | NCT04256707-2 | Pembrolizumab | PD-1 | Other solid tumors |
| NCT04256707 | NCT04256707-3 | Selinexor | XPO1 | Other solid tumors |
| NCT04256707 | NCT04256707-3 | Folfiri | chemotherapy | Other solid tumors |
| NCT04257448 | NCT04257448-0 | Durvalumab | PD-L1 | Pancreas cancer |
| NCT04257448 | NCT04257448-0 | Lenalidomide | CRBN | Pancreas cancer |
| NCT04257578 | NCT04257578-0 | Acalabrutinib | BTK | Lymphomas |
| NCT04257578 | NCT04257578-0 | Axicabtagene Ciloleucel | cell therapy | Lymphomas |
| NCT04258956 | NCT04258956-0 | Avelumab | PD-L1 | Other solid tumors |
| NCT04258956 | NCT04258956-0 | Axitinib | VEGFR/PDGFR | Other solid tumors |
| NCT04261075 | NCT04261075-1 | Iph5201 | CD39 | Other solid tumors |
| NCT04261075 | NCT04261075-1 | Durvalumab | PD-L1 | Other solid tumors |
| NCT04261075 | NCT04261075-2 | Iph5201 | CD39 | Other solid tumors |
| NCT04261075 | NCT04261075-2 | Durvalumab | PD-L1 | Other solid tumors |
| NCT04261075 | NCT04261075-2 | Oleclumab | CD73 | Other solid tumors |
| NCT04261855 | NCT04261855-0 | Avelumab | PD-L1 | Other solid tumors |
| NCT04261855 | NCT04261855-0 | Lutetium Lu 177 Dotatate | SSTR | Other solid tumors |
| NCT04262869 | NCT04262869-0 | Chemotherapy | chemotherapy | lung cancers |
| NCT04262869 | NCT04262869-0 | Durvalumab | PD-L1 | lung cancers |
| NCT04263584 | NCT04263584-0 | Copanlisib | PI3K | Lymphomas |
| NCT04263584 | NCT04263584-0 | Rituximab | CD20 | Lymphomas |
| NCT04263584 | NCT04263584-0 | Chemotherapy | chemotherapy | Lymphomas |
| NCT04266912 | NCT04266912-0 | Avelumab | PD-L1 | Other solid tumors |
| NCT04266912 | NCT04266912-0 | Berzosertib | ATR | Other solid tumors |
| NCT04267887 | NCT04267887-0 | Apalutamide | Endocrine Therapy | Prostate cancer |
| NCT04267887 | NCT04267887-0 | Abiraterone | CYP17A1 | Prostate cancer |
| NCT04267887 | NCT04267887-0 | Prednisone | Steroid Treatment | Prostate cancer |
| NCT04267887 | NCT04267887-0 | Androgen Deprivation Therapy (Adt) | Endocrine Therapy | Prostate cancer |
| NCT04267939 | NCT04267939-0 | Elimusertib | ATR | Ovary cancer |
| NCT04267939 | NCT04267939-0 | Niraparib | PARP | Ovary cancer |
| NCT04269200 | NCT04269200-0 | Durvalumab | PD-L1 | Corpus uteri cancer |
| NCT04269200 | NCT04269200-0 | Olaparib | PARP | Corpus uteri cancer |
| NCT04270409 | NCT04270409-0 | Isatuximab | CD38 | Multiple Myeloma |
| NCT04270409 | NCT04270409-0 | Lenalidomide | CRBN | Multiple Myeloma |
| NCT04270409 | NCT04270409-0 | Dexamethasone | Steroid Treatment | Multiple Myeloma |
| NCT04271956 | NCT04271956-0 | Tislelizumab | PD-1 | Other hematologic Neoplasms |
| NCT04271956 | NCT04271956-0 | Zanubrutinib | BTK | Other hematologic Neoplasms |
| NCT04277637 | NCT04277637-0 | Bgb-11417 | BCL | Lymphomas |
| NCT04277637 | NCT04277637-0 | Zanubrutinib | BTK | Lymphomas |
| NCT04281498 | NCT04281498-0 | Ruxolitinib | JAK | Lymphomas |
| NCT04281498 | NCT04281498-0 | Enasidenib | IDH | Lymphomas |
| NCT04282018 | NCT04282018-1 | Bgb-10188 | PI3K | Others |
| NCT04282018 | NCT04282018-1 | Zanubrutinib | BTK | Others |
| NCT04282018 | NCT04282018-2 | Bgb-10188 | PI3K | Others |
| NCT04282018 | NCT04282018-2 | Tislelizumab | PD-1 | Others |
| NCT04282187 | NCT04282187-0 | Decitabine | chemotherapy | Lymphomas |
| NCT04282187 | NCT04282187-0 | Fedratinib | JAK | Lymphomas |
| NCT04287855 | NCT04287855-0 | Isatuximab | CD38 | Multiple Myeloma |
| NCT04287855 | NCT04287855-0 | Pomalidomide | CRBN | Multiple Myeloma |
| NCT04287855 | NCT04287855-0 | Dexamethasone | Steroid Treatment | Multiple Myeloma |
| NCT04287855 | NCT04287855-0 | Carfilzomib | PSMB | Multiple Myeloma |
| NCT04287894 | NCT04287894-0 | Durvalumab | PD-L1 | lung cancers |
| NCT04287894 | NCT04287894-0 | Tremelimumab | CTLA-4 | lung cancers |
| NCT04291105 | NCT04291105-0 | Voyagerv1 | oncolytic virus | Other solid tumors |
| NCT04291105 | NCT04291105-0 | Cemiplimab-Rwlc | PD-1 | Other solid tumors |
| NCT04292119 | NCT04292119-1 | Lorlatinib | ALK | lung cancers |
| NCT04292119 | NCT04292119-1 | Crizotinib | ALK/MET | lung cancers |
| NCT04292119 | NCT04292119-2 | Lorlatinib | ALK | lung cancers |
| NCT04292119 | NCT04292119-2 | Tno155 | SHP2 | lung cancers |
| NCT04292119 | NCT04292119-3 | Binimetinib | MEK | lung cancers |
| NCT04292119 | NCT04292119-3 | Lorlatinib | ALK | lung cancers |
| NCT04293393 | NCT04293393-1 | Letrozole | Endocrine Therapy | Breast cancer |
| NCT04293393 | NCT04293393-1 | Abemaciclib | CDK4/CDK6 | Breast cancer |
| NCT04293393 | NCT04293393-1 | Goserelin | Endocrine Therapy | Breast cancer |
| NCT04293393 | NCT04293393-2 | Letrozole | Endocrine Therapy | Breast cancer |
| NCT04293393 | NCT04293393-2 | Abemaciclib | CDK4/CDK6 | Breast cancer |
| NCT04293393 | NCT04293393-2 | Leuprolide | Endocrine Therapy | Breast cancer |
| NCT04293419 | NCT04293419-0 | Oxaliplatin | chemotherapy | Colon and rectum cancers |
| NCT04293419 | NCT04293419-0 | Leucovorin | Other supportive therapy | Colon and rectum cancers |
| NCT04293419 | NCT04293419-0 | 5-Fu | chemotherapy | Colon and rectum cancers |
| NCT04293419 | NCT04293419-0 | Durvalumab | PD-L1 | Colon and rectum cancers |
| NCT04293562 | NCT04293562-0 | Gilteritinib | FLT3 | Leukaemia |
| NCT04293562 | NCT04293562-0 | Chemotherapy | chemotherapy | Leukaemia |
| NCT04298008 | NCT04298008-0 | Azd6738 | ATR | Gallbladder and biliary tract cancer |
| NCT04298008 | NCT04298008-0 | Durvalumab | PD-L1 | Gallbladder and biliary tract cancer |
| NCT04298021 | NCT04298021-1 | Azd6738 | ATR | Gallbladder and biliary tract cancer |
| NCT04298021 | NCT04298021-1 | Durvalumab | PD-L1 | Gallbladder and biliary tract cancer |
| NCT04298021 | NCT04298021-2 | Azd6738 | ATR | Gallbladder and biliary tract cancer |
| NCT04298021 | NCT04298021-2 | Olaparib | PARP | Gallbladder and biliary tract cancer |
| NCT04298983 | NCT04298983-0 | Abemaciclib | CDK4/CDK6 | Prostate cancer |
| NCT04298983 | NCT04298983-0 | Androgen Deprivation Therapy (Adt) | Endocrine Therapy | Prostate cancer |
| NCT04300790 | NCT04300790-0 | Alpelisib | PI3K | Breast cancer |
| NCT04300790 | NCT04300790-0 | Metformin | Antihyperglycemic | Breast cancer |
| NCT04300790 | NCT04300790-0 | Fulvestrant | Endocrine Therapy | Breast cancer |
| NCT04301778 | NCT04301778-0 | Durvalumab | PD-L1 | Gallbladder and biliary tract cancer |
| NCT04301778 | NCT04301778-0 | Sndx-6352 | CSF1R | Gallbladder and biliary tract cancer |
| NCT04305236 | NCT04305236-0 | Abemaciclib | CDK4/CDK6 | Breast cancer |
| NCT04305236 | NCT04305236-0 | Fulvestrant | Endocrine Therapy | Breast cancer |
| NCT04305795 | NCT04305795-0 | Asp-1929 | EGFR | Head and Neck Neoplasms |
| NCT04305795 | NCT04305795-0 | Cemiplimab-Rwlc | PD-1 | Head and Neck Neoplasms |
| NCT04308174 | NCT04308174-0 | Gemcitabine | chemotherapy | Gallbladder and biliary tract cancer |
| NCT04308174 | NCT04308174-0 | Platinum-Based Chemotherapy | chemotherapy | Gallbladder and biliary tract cancer |
| NCT04308174 | NCT04308174-0 | Durvalumab | PD-L1 | Gallbladder and biliary tract cancer |
| NCT04313504 | NCT04313504-0 | Niraparib | PARP | Head and Neck Neoplasms |
| NCT04313504 | NCT04313504-0 | Dostarlimab-Gxly | PD-1 | Head and Neck Neoplasms |
| NCT04314297 | NCT04314297-0 | Anlotinib | VEGFR/PDGFR/FGFR | lung cancers |
| NCT04314297 | NCT04314297-0 | Durvalumab | PD-L1 | lung cancers |
| NCT04315233 | NCT04315233-0 | Ribociclib | CDK4/CDK6 | Other solid tumors |
| NCT04315233 | NCT04315233-0 | Belinostat | chemotherapy | Other solid tumors |
| NCT04316169 | NCT04316169-0 | Abemaciclib | CDK4/CDK6 | Breast cancer |
| NCT04316169 | NCT04316169-0 | Hydroxychloroquine | antimalarial agent | Breast cancer |
| NCT04317105 | NCT04317105-1 | Copanlisib | PI3K | Other solid tumors |
| NCT04317105 | NCT04317105-1 | Nivolumab | PD-1 | Other solid tumors |
| NCT04317105 | NCT04317105-1 | Ipilimumab | CTLA-4 | Other solid tumors |
| NCT04317105 | NCT04317105-2 | Copanlisib | PI3K | Other solid tumors |
| NCT04317105 | NCT04317105-2 | Nivolumab | PD-1 | Other solid tumors |
| NCT04323436 | NCT04323436-0 | Capmatinib | MET | lung cancers |
| NCT04323436 | NCT04323436-0 | Spartalizumab | PD-1 | lung cancers |
| NCT04324112 | NCT04324112-0 | Binimetinib | MEK | Leukaemia |
| NCT04324112 | NCT04324112-0 | Encorafenib | RAF | Leukaemia |
| NCT04325828 | NCT04325828-0 | Apalutamide | Endocrine Therapy | Other solid tumors |
| NCT04325828 | NCT04325828-0 | Gnrh Agonist | Endocrine Therapy | Other solid tumors |
| NCT04332744 | NCT04332744-0 | Enzalutamide | Endocrine Therapy | Prostate cancer |
| NCT04332744 | NCT04332744-0 | Talazoparib | PARP | Prostate cancer |
| NCT04332822 | NCT04332822-0 | Polatuzumab Vedotin | CD79B/Tubulin | Lymphomas |
| NCT04332822 | NCT04332822-0 | Rituximab | CD20 | Lymphomas |
| NCT04332822 | NCT04332822-0 | Chemotherapy | chemotherapy | Lymphomas |
| NCT04334759 | NCT04334759-0 | Durvalumab | PD-L1 | Other solid tumors |
| NCT04334759 | NCT04334759-0 | Chemotherapy | chemotherapy | Other solid tumors |
| NCT04334941 | NCT04334941-0 | Atezolizumab | PD-L1 | lung cancers |
| NCT04334941 | NCT04334941-0 | Talazoparib | PARP | lung cancers |
| NCT04336943 | NCT04336943-0 | Durvalumab | PD-L1 | Prostate cancer |
| NCT04336943 | NCT04336943-0 | Olaparib | PARP | Prostate cancer |
| NCT04336982 | NCT04336982-1 | Cc-90009 | GSPT1 | Leukaemia |
| NCT04336982 | NCT04336982-1 | Venetoclax | BCL2 | Leukaemia |
| NCT04336982 | NCT04336982-1 | Azacitidine | chemotherapy | Leukaemia |
| NCT04336982 | NCT04336982-2 | Cc-90009 | GSPT1 | Leukaemia |
| NCT04336982 | NCT04336982-2 | Gilteritinib | FLT3 | Leukaemia |
| NCT04337827 | NCT04337827-0 | Rituximab | CD20 | Lymphomas |
| NCT04337827 | NCT04337827-0 | Acalabrutinib | BTK | Lymphomas |
| NCT04337970 | NCT04337970-0 | Talazoparib | PARP | Kidney cancer |
| NCT04337970 | NCT04337970-0 | Axitinib | VEGFR/PDGFR | Kidney cancer |
| NCT04339062 | NCT04339062-1 | Cemiplimab-Rwlc | PD-1 | Others |
| NCT04339062 | NCT04339062-1 | Everolimus | mTOR | Others |
| NCT04339062 | NCT04339062-1 | Prednisone | Steroid Treatment | Others |
| NCT04339062 | NCT04339062-2 | Cemiplimab-Rwlc | PD-1 | Others |
| NCT04339062 | NCT04339062-2 | Sirolimus | mTOR | Others |
| NCT04339062 | NCT04339062-2 | Prednisone | Steroid Treatment | Others |
| NCT04341181 | NCT04341181-1 | Vemurafenib | RAF | Others |
| NCT04341181 | NCT04341181-1 | Cobimetinib | MEK | Others |
| NCT04341181 | NCT04341181-2 | Trastuzumab | HER2 | Others |
| NCT04341181 | NCT04341181-2 | Pertuzumab | HER2 | Others |
| NCT04345913 | NCT04345913-0 | Copanlisib | PI3K | Breast cancer |
| NCT04345913 | NCT04345913-0 | Eribulin | chemotherapy | Breast cancer |
| NCT04348045 | NCT04348045-0 | Durvalumab | PD-L1 | Pancreas cancer |
| NCT04348045 | NCT04348045-0 | Selumetinib | MEK | Pancreas cancer |
| NCT04348292 | NCT04348292-0 | Sirolimus | mTOR | lung cancers |
| NCT04348292 | NCT04348292-0 | Durvalumab | PD-L1 | lung cancers |
| NCT04351230 | NCT04351230-0 | Trastuzumab Emtansine | HER2/Tubulin | Breast cancer |
| NCT04351230 | NCT04351230-0 | Abemaciclib | CDK4/CDK6 | Breast cancer |
| NCT04352777 | NCT04352777-1 | Fulvestrant | Endocrine Therapy | Breast cancer |
| NCT04352777 | NCT04352777-1 | Abemaciclib | CDK4/CDK6 | Breast cancer |
| NCT04352777 | NCT04352777-2 | Aromatase Inhibitor | Endocrine Therapy | Breast cancer |
| NCT04352777 | NCT04352777-2 | Abemaciclib | CDK4/CDK6 | Breast cancer |
| NCT04356222 | NCT04356222-0 | Durvalumab | PD-L1 | Brain and nervous system cancers |
| NCT04356222 | NCT04356222-0 | Chemotherapy | chemotherapy | Brain and nervous system cancers |
| NCT04358237 | NCT04358237-0 | Lurbinectedin | chemotherapy | lung cancers |
| NCT04358237 | NCT04358237-0 | Pembrolizumab | PD-1 | lung cancers |
| NCT04360941 | NCT04360941-0 | Avelumab | PD-L1 | Breast cancer |
| NCT04360941 | NCT04360941-0 | Palbociclib | CDK4/CDK6 | Breast cancer |
| NCT04361825 | NCT04361825-0 | Durvalumab | PD-L1 | lung cancers |
| NCT04361825 | NCT04361825-0 | Azd6738 | ATR | lung cancers |
| NCT04364048 | NCT04364048-0 | Durvalumab | PD-L1 | lung cancers |
| NCT04364048 | NCT04364048-0 | Platinum-Based Chemotherapy | chemotherapy | lung cancers |
| NCT04366713 | NCT04366713-0 | Neratinib | EGFR | Breast cancer |
| NCT04366713 | NCT04366713-0 | Loperamide | Other supportive therapy | Breast cancer |
| NCT04366713 | NCT04366713-0 | Capecitabine | chemotherapy | Breast cancer |
| NCT04372927 | NCT04372927-1 | Cisplatin | chemotherapy | lung cancers |
| NCT04372927 | NCT04372927-1 | Pemetrexed | chemotherapy | lung cancers |
| NCT04372927 | NCT04372927-1 | Durvalumab | PD-L1 | lung cancers |
| NCT04372927 | NCT04372927-2 | Cisplatin | chemotherapy | lung cancers |
| NCT04372927 | NCT04372927-2 | Etoposide | chemotherapy | lung cancers |
| NCT04372927 | NCT04372927-2 | Durvalumab | PD-L1 | lung cancers |
| NCT04375267 | NCT04375267-0 | Lutetium Lu 177 Dotatate | SSTR | Other solid tumors |
| NCT04375267 | NCT04375267-0 | Olaparib | PARP | Other solid tumors |
| NCT04375527 | NCT04375527-0 | Binimetinib | MEK | Other solid tumors |
| NCT04375527 | NCT04375527-0 | Nivolumab | PD-1 | Other solid tumors |
| NCT04376073 | NCT04376073-0 | Anlotinib | VEGFR/PDGFR/FGFR | Ovary cancer |
| NCT04376073 | NCT04376073-0 | Niraparib | PARP | Ovary cancer |
| NCT04379596 | NCT04379596-1 | Trastuzumab Deruxtecan | HER2/TOP1 | Stomach cancer |
| NCT04379596 | NCT04379596-1 | 5-Fu | chemotherapy | Stomach cancer |
| NCT04379596 | NCT04379596-2 | Trastuzumab Deruxtecan | HER2/TOP1 | Stomach cancer |
| NCT04379596 | NCT04379596-2 | Capecitabine | chemotherapy | Stomach cancer |
| NCT04379596 | NCT04379596-3 | Trastuzumab Deruxtecan | HER2/TOP1 | Stomach cancer |
| NCT04379596 | NCT04379596-3 | Durvalumab | PD-L1 | Stomach cancer |
| NCT04379596 | NCT04379596-4 | Trastuzumab Deruxtecan | HER2/TOP1 | Stomach cancer |
| NCT04379596 | NCT04379596-4 | 5-Fu | chemotherapy | Stomach cancer |
| NCT04379596 | NCT04379596-4 | Platinum-Based Chemotherapy | chemotherapy | Stomach cancer |
| NCT04379596 | NCT04379596-5 | Trastuzumab Deruxtecan | HER2/TOP1 | Stomach cancer |
| NCT04379596 | NCT04379596-5 | Capecitabine | chemotherapy | Stomach cancer |
| NCT04379596 | NCT04379596-5 | Platinum-Based Chemotherapy | chemotherapy | Stomach cancer |
| NCT04379596 | NCT04379596-6 | Trastuzumab Deruxtecan | HER2/TOP1 | Stomach cancer |
| NCT04379596 | NCT04379596-6 | 5-Fu | chemotherapy | Stomach cancer |
| NCT04379596 | NCT04379596-6 | Durvalumab | PD-L1 | Stomach cancer |
| NCT04379596 | NCT04379596-7 | Trastuzumab Deruxtecan | HER2/TOP1 | Stomach cancer |
| NCT04379596 | NCT04379596-7 | Capecitabine | chemotherapy | Stomach cancer |
| NCT04379596 | NCT04379596-7 | Durvalumab | PD-L1 | Stomach cancer |
| NCT04379596 | NCT04379596-8 | Trastuzumab | HER2 | Stomach cancer |
| NCT04379596 | NCT04379596-8 | 5-Fu | chemotherapy | Stomach cancer |
| NCT04379596 | NCT04379596-8 | Platinum-Based Chemotherapy | chemotherapy | Stomach cancer |
| NCT04380636 | NCT04380636-0 | Pembrolizumab | PD-1 | lung cancers |
| NCT04380636 | NCT04380636-0 | Olaparib | PARP | lung cancers |
| NCT04380636 | NCT04380636-0 | Durvalumab | PD-L1 | lung cancers |
| NCT04382898 | NCT04382898-0 | W_Pro1 | vaccine | Prostate cancer |
| NCT04382898 | NCT04382898-0 | Cemiplimab-Rwlc | PD-1 | Prostate cancer |
| NCT04384484 | NCT04384484-0 | Loncastuximab Tesirine | CD19/DNA | Lymphomas |
| NCT04384484 | NCT04384484-0 | Rituximab | CD20 | Lymphomas |
| NCT04385290 | NCT04385290-1 | Midostaurin | PKC/PDGFR | Leukaemia |
| NCT04385290 | NCT04385290-1 | Gemtuzumab Ozogamicin | CD33/DNA | Leukaemia |
| NCT04385290 | NCT04385290-1 | Daunorubicin | chemotherapy | Leukaemia |
| NCT04385290 | NCT04385290-2 | Midostaurin | PKC/PDGFR | Leukaemia |
| NCT04385290 | NCT04385290-2 | Cytarabine | chemotherapy | Leukaemia |
| NCT04385290 | NCT04385290-2 | Daunorubicin | chemotherapy | Leukaemia |
| NCT04385290 | NCT04385290-3 | Gemtuzumab Ozogamicin | CD33/DNA | Leukaemia |
| NCT04385290 | NCT04385290-3 | Midostaurin | PKC/PDGFR | Leukaemia |
| NCT04385290 | NCT04385290-3 | Chemotherapy | chemotherapy | Leukaemia |
| NCT04385290 | NCT04385290-4 | Chemotherapy | chemotherapy | Leukaemia |
| NCT04385290 | NCT04385290-4 | Midostaurin | PKC/PDGFR | Leukaemia |
| NCT04385368 | NCT04385368-0 | Durvalumab | PD-L1 | lung cancers |
| NCT04385368 | NCT04385368-0 | Chemotherapy | chemotherapy | lung cancers |
| NCT04390243 | NCT04390243-0 | Binimetinib | MEK | Pancreas cancer |
| NCT04390243 | NCT04390243-0 | Encorafenib | RAF | Pancreas cancer |
| NCT04391595 | NCT04391595-0 | Abemaciclib | CDK4/CDK6 | Brain and nervous system cancers |
| NCT04391595 | NCT04391595-0 | Temuterkib | ERK | Brain and nervous system cancers |
| NCT04393285 | NCT04393285-0 | Abemaciclib | CDK4/CDK6 | Corpus uteri cancer |
| NCT04393285 | NCT04393285-0 | Letrozole | Endocrine Therapy | Corpus uteri cancer |
| NCT04393753 | NCT04393753-0 | Domatinostat | chemotherapy | Other solid tumors |
| NCT04393753 | NCT04393753-0 | Avelumab | PD-L1 | Other solid tumors |
| NCT04395079 | NCT04395079-0 | Durvalumab | PD-L1 | Other solid tumors |
| NCT04395079 | NCT04395079-0 | Tremelimumab | CTLA-4 | Other solid tumors |
| NCT04395612 | NCT04395612-1 | Niraparib | PARP | Cervix uteri cancer |
| NCT04395612 | NCT04395612-1 | Brivanib | VEGFR/FGFR | Cervix uteri cancer |
| NCT04395612 | NCT04395612-2 | Niraparib | PARP | Cervix uteri cancer |
| NCT04395612 | NCT04395612-2 | Toripalimab | PD-1 | Cervix uteri cancer |
| NCT04396223 | NCT04396223-0 | Avelumab | PD-L1 | Other solid tumors |
| NCT04396223 | NCT04396223-0 | Methotrexate | chemotherapy | Other solid tumors |
| NCT04398524 | NCT04398524-0 | Isa101B | vaccine | Others |
| NCT04398524 | NCT04398524-0 | Cemiplimab-Rwlc | PD-1 | Others |
| NCT04404088 | NCT04404088-0 | Acalabrutinib | BTK | Lymphomas |
| NCT04404088 | NCT04404088-0 | Lenalidomide | CRBN | Lymphomas |
| NCT04404088 | NCT04404088-0 | Rituximab | CD20 | Lymphomas |
| NCT04409002 | NCT04409002-0 | Niraparib | PARP | Pancreas cancer |
| NCT04409002 | NCT04409002-0 | Dostarlimab-Gxly | PD-1 | Pancreas cancer |
| NCT04409288 | NCT04409288-0 | Enzalutamide | Endocrine Therapy | Prostate cancer |
| NCT04409288 | NCT04409288-0 | Apalutamide | Endocrine Therapy | Prostate cancer |
| NCT04414475 | NCT04414475-0 | Selinexor | XPO1 | Multiple Myeloma |
| NCT04414475 | NCT04414475-0 | Dexamethasone | Steroid Treatment | Multiple Myeloma |
| NCT04417621 | NCT04417621-0 | Naporafenib | RAF | Melanoma |
| NCT04417621 | NCT04417621-0 | Ribociclib | CDK4/CDK6 | Melanoma |
| NCT04421378 | NCT04421378-1 | Selinexor | XPO1 | Brain and nervous system cancers |
| NCT04421378 | NCT04421378-1 | Temozolomide | chemotherapy | Brain and nervous system cancers |
| NCT04421378 | NCT04421378-2 | Selinexor | XPO1 | Brain and nervous system cancers |
| NCT04421378 | NCT04421378-2 | Lomustine | chemotherapy | Brain and nervous system cancers |
| NCT04421378 | NCT04421378-2 | Carmustine | chemotherapy | Brain and nervous system cancers |
| NCT04421378 | NCT04421378-3 | Selinexor | XPO1 | Brain and nervous system cancers |
| NCT04421378 | NCT04421378-3 | Bevacizumab | VEGF | Brain and nervous system cancers |
| NCT04423185 | NCT04423185-1 | Niraparib | PARP | Others |
| NCT04423185 | NCT04423185-1 | Sintilimab | PD-1 | Others |
| NCT04423185 | NCT04423185-2 | Vemurafenib | RAF | Others |
| NCT04423185 | NCT04423185-2 | Atezolizumab | PD-L1 | Others |
| NCT04423185 | NCT04423185-3 | Palbociclib | CDK4/CDK6 | Others |
| NCT04423185 | NCT04423185-3 | Atezolizumab | PD-L1 | Others |
| NCT04423211 | NCT04423211-0 | Goserelin | Endocrine Therapy | Prostate cancer |
| NCT04423211 | NCT04423211-0 | Leuprolide | Endocrine Therapy | Prostate cancer |
| NCT04423211 | NCT04423211-0 | Apalutamide | Endocrine Therapy | Prostate cancer |
| NCT04425070 | NCT04425070-0 | Selinexor | XPO1 | Lymphomas |
| NCT04425070 | NCT04425070-0 | Chemotherapy | chemotherapy | Lymphomas |
| NCT04427072 | NCT04427072-0 | Capmatinib | MET | lung cancers |
| NCT04427072 | NCT04427072-0 | Docetaxel | chemotherapy | lung cancers |
| NCT04430452 | NCT04430452-0 | Durvalumab | PD-L1 | Liver cancer |
| NCT04430452 | NCT04430452-0 | Tremelimumab | CTLA-4 | Liver cancer |
| NCT04430738 | NCT04430738-1 | Tucatinib | HER2 | Other solid tumors |
| NCT04430738 | NCT04430738-1 | Trastuzumab | HER2 | Other solid tumors |
| NCT04430738 | NCT04430738-1 | Folfox | chemotherapy | Other solid tumors |
| NCT04430738 | NCT04430738-2 | Tucatinib | HER2 | Other solid tumors |
| NCT04430738 | NCT04430738-2 | Trastuzumab | HER2 | Other solid tumors |
| NCT04430738 | NCT04430738-2 | Platinum-Based Chemotherapy | chemotherapy | Other solid tumors |
| NCT04430738 | NCT04430738-2 | Capecitabine | chemotherapy | Other solid tumors |
| NCT04430894 | NCT04430894-0 | Carfilzomib | PSMB | Multiple Myeloma |
| NCT04430894 | NCT04430894-0 | Isatuximab | CD38 | Multiple Myeloma |
| NCT04430894 | NCT04430894-0 | Lenalidomide | CRBN | Multiple Myeloma |
| NCT04430894 | NCT04430894-0 | Dexamethasone | Steroid Treatment | Multiple Myeloma |
| NCT04431635 | NCT04431635-0 | Copanlisib | PI3K | Lymphomas |
| NCT04431635 | NCT04431635-0 | Nivolumab | PD-1 | Lymphomas |
| NCT04431635 | NCT04431635-0 | Rituximab | CD20 | Lymphomas |
| NCT04432454 | NCT04432454-0 | Lasofoxifene | Endocrine Therapy | Breast cancer |
| NCT04432454 | NCT04432454-0 | Abemaciclib | CDK4/CDK6 | Breast cancer |
| NCT04433182 | NCT04433182-0 | Copanlisib | PI3K | Lymphomas |
| NCT04433182 | NCT04433182-0 | Rituximab | CD20 | Lymphomas |
| NCT04433182 | NCT04433182-0 | Bendamustine | chemotherapy | Lymphomas |
| NCT04436107 | NCT04436107-1 | Zanubrutinib | BTK | Lymphomas |
| NCT04436107 | NCT04436107-1 | Rituximab | CD20 | Lymphomas |
| NCT04436107 | NCT04436107-1 | Lenalidomide | CRBN | Lymphomas |
| NCT04436107 | NCT04436107-2 | Zanubrutinib | BTK | Lymphomas |
| NCT04436107 | NCT04436107-2 | Lenalidomide | CRBN | Lymphomas |
| NCT04442022 | NCT04442022-0 | Selinexor | XPO1 | Lymphomas |
| NCT04442022 | NCT04442022-0 | Rituximab | CD20 | Lymphomas |
| NCT04442022 | NCT04442022-0 | Chemotherapy | chemotherapy | Lymphomas |
| NCT04443322 | NCT04443322-0 | Durvalumab | PD-L1 | Liver cancer |
| NCT04443322 | NCT04443322-0 | Lenvatinib | VEGFR/PDGFR/FGFR | Liver cancer |
| NCT04444193 | NCT04444193-0 | Durvalumab | PD-L1 | Corpus uteri cancer |
| NCT04444193 | NCT04444193-0 | Lenvatinib | VEGFR/PDGFR/FGFR | Corpus uteri cancer |
| NCT04446351 | NCT04446351-1 | Gsk6097608 | CD96 | Others |
| NCT04446351 | NCT04446351-1 | Dostarlimab-Gxly | PD-1 | Others |
| NCT04446351 | NCT04446351-2 | Dostarlimab-Gxly | PD-1 | Others |
| NCT04446351 | NCT04446351-2 | Gsk4428859A | TIGIT | Others |
| NCT04447612 | NCT04447612-0 | Durvalumab | PD-L1 | Mouth and oropharynx cancers |
| NCT04447612 | NCT04447612-0 | Chemotherapy | chemotherapy | Mouth and oropharynx cancers |
| NCT04448886 | NCT04448886-0 | Sacituzumab Govitecan | Trop-2/TOP1 | Breast cancer |
| NCT04448886 | NCT04448886-0 | Pembrolizumab | PD-1 | Breast cancer |
| NCT04449861 | NCT04449861-0 | Durvalumab | PD-L1 | lung cancers |
| NCT04449861 | NCT04449861-0 | Chemotherapy | chemotherapy | lung cancers |
| NCT04457596 | NCT04457596-0 | Tucatinib | HER2 | Breast cancer |
| NCT04457596 | NCT04457596-0 | Trastuzumab Emtansine | HER2/Tubulin | Breast cancer |
| NCT04458610 | NCT04458610-0 | Zanubrutinib | BTK | Leukaemia |
| NCT04458610 | NCT04458610-0 | Rituximab | CD20 | Leukaemia |
| NCT04460248 | NCT04460248-0 | Zanubrutinib | BTK | Lymphomas |
| NCT04460248 | NCT04460248-0 | Lenalidomide | CRBN | Lymphomas |
| NCT04460248 | NCT04460248-0 | Rituximab | CD20 | Lymphomas |
| NCT04460430 | NCT04460430-0 | Neratinib | EGFR | Breast cancer |
| NCT04460430 | NCT04460430-0 | Endocrine Therapy | Endocrine Therapy | Breast cancer |
| NCT04462328 | NCT04462328-0 | Acalabrutinib | BTK | Lymphomas |
| NCT04462328 | NCT04462328-0 | Durvalumab | PD-L1 | Lymphomas |
| NCT04462471 | NCT04462471-0 | Vemurafenib | RAF | Other solid tumors |
| NCT04462471 | NCT04462471-0 | Copanlisib | PI3K | Other solid tumors |
| NCT04463771 | NCT04463771-0 | Retifanlimab | PD-1 | Corpus uteri cancer |
| NCT04463771 | NCT04463771-0 | Pemigatinib | FGFR | Corpus uteri cancer |
| NCT04463953 | NCT04463953-0 | Zanubrutinib | BTK | Other hematologic Neoplasms |
| NCT04463953 | NCT04463953-0 | Ixazomib | PSMB | Other hematologic Neoplasms |
| NCT04463953 | NCT04463953-0 | Dexamethasone | Steroid Treatment | Other hematologic Neoplasms |
| NCT04465487 | NCT04465487-0 | Regn6569 | GITR | Head and Neck Neoplasms |
| NCT04465487 | NCT04465487-0 | Cemiplimab-Rwlc | PD-1 | Head and Neck Neoplasms |
| NCT04465968 | NCT04465968-0 | Cisplatin | chemotherapy | lung cancers |
| NCT04465968 | NCT04465968-0 | S-1 | chemotherapy | lung cancers |
| NCT04465968 | NCT04465968-0 | Durvalumab | PD-L1 | lung cancers |
| NCT04468061 | NCT04468061-0 | Sacituzumab Govitecan | Trop-2/TOP1 | Breast cancer |
| NCT04468061 | NCT04468061-0 | Pembrolizumab | PD-1 | Breast cancer |
| NCT04469764 | NCT04469764-1 | Abemaciclib | CDK4/CDK6 | Other solid tumors |
| NCT04469764 | NCT04469764-1 | Anastrozole | Endocrine Therapy | Other solid tumors |
| NCT04469764 | NCT04469764-2 | Abemaciclib | CDK4/CDK6 | Other solid tumors |
| NCT04469764 | NCT04469764-2 | Letrozole | Endocrine Therapy | Other solid tumors |
| NCT04470674 | NCT04470674-0 | Durvalumab | PD-L1 | lung cancers |
| NCT04470674 | NCT04470674-0 | Chemotherapy | chemotherapy | lung cancers |
| NCT04472949 | NCT04472949-0 | Platinum-Based Chemotherapy | chemotherapy | lung cancers |
| NCT04472949 | NCT04472949-0 | Etoposide | chemotherapy | lung cancers |
| NCT04472949 | NCT04472949-0 | Durvalumab | PD-L1 | lung cancers |
| NCT04475939 | NCT04475939-0 | Niraparib | PARP | lung cancers |
| NCT04475939 | NCT04475939-0 | Pembrolizumab | PD-1 | lung cancers |
| NCT04479267 | NCT04479267-0 | Polatuzumab Vedotin | CD79B/Tubulin | Lymphomas |
| NCT04479267 | NCT04479267-0 | Rituximab | CD20 | Lymphomas |
| NCT04479267 | NCT04479267-0 | Cyclophosphamide | chemotherapy | Lymphomas |
| NCT04479267 | NCT04479267-0 | Doxorubicin | chemotherapy | Lymphomas |
| NCT04479267 | NCT04479267-0 | Prednisone | Steroid Treatment | Lymphomas |
| NCT04481113 | NCT04481113-0 | Abemaciclib | CDK4/CDK6 | Breast cancer |
| NCT04481113 | NCT04481113-0 | Niraparib | PARP | Breast cancer |
| NCT04483739 | NCT04483739-0 | Isatuximab | CD38 | Multiple Myeloma |
| NCT04483739 | NCT04483739-0 | Carfilzomib | PSMB | Multiple Myeloma |
| NCT04483739 | NCT04483739-0 | Lenalidomide | CRBN | Multiple Myeloma |
| NCT04483739 | NCT04483739-0 | Dexamethasone | Steroid Treatment | Multiple Myeloma |
| NCT04484012 | NCT04484012-0 | Cd19 Cart Cells | cell therapy | Lymphomas |
| NCT04484012 | NCT04484012-0 | Acalabrutinib | BTK | Lymphomas |
| NCT04484623 | NCT04484623-0 | Belantamab Mafodotin | BCMA/Tubulin | Multiple Myeloma |
| NCT04484623 | NCT04484623-0 | Pomalidomide | CRBN | Multiple Myeloma |
| NCT04484623 | NCT04484623-0 | Dexamethasone | Steroid Treatment | Multiple Myeloma |
| NCT04484818 | NCT04484818-0 | Androgen Deprivation Therapy (Adt) | Endocrine Therapy | Prostate cancer |
| NCT04484818 | NCT04484818-0 | Darolutamide | Endocrine Therapy | Prostate cancer |
| NCT04486352 | NCT04486352-1 | Atezolizumab | PD-L1 | Corpus uteri cancer |
| NCT04486352 | NCT04486352-1 | Bevacizumab | VEGF | Corpus uteri cancer |
| NCT04486352 | NCT04486352-2 | Atezolizumab | PD-L1 | Corpus uteri cancer |
| NCT04486352 | NCT04486352-2 | Ipatasertib | AKT | Corpus uteri cancer |
| NCT04486352 | NCT04486352-3 | Atezolizumab | PD-L1 | Corpus uteri cancer |
| NCT04486352 | NCT04486352-3 | Talazoparib | PARP | Corpus uteri cancer |
| NCT04487080 | NCT04988295-1 | Amivantamab | EGFR/MET | lung cancers |
| NCT04487080 | NCT04988295-1 | Lazertinib | EGFR | lung cancers |
| NCT04492033 | NCT04492033-1 | Asciminib | BCR-ABL | Gallbladder and biliary tract cancer |
| NCT04492033 | NCT04492033-1 | Paclitaxel | chemotherapy | Gallbladder and biliary tract cancer |
| NCT04492033 | NCT04492033-2 | Asciminib | BCR-ABL | Gallbladder and biliary tract cancer |
| NCT04492033 | NCT04492033-2 | Irinotecan | chemotherapy | Gallbladder and biliary tract cancer |
| NCT04493060 | NCT04493060-0 | Niraparib | PARP | Pancreas cancer |
| NCT04493060 | NCT04493060-0 | Dostarlimab-Gxly | PD-1 | Pancreas cancer |
| NCT04493164 | NCT04493164-0 | Ivosidenib | IDH | Leukaemia |
| NCT04493164 | NCT04493164-0 | Cytarabine | chemotherapy | Leukaemia |
| NCT04493164 | NCT04493164-0 | Daunorubicin | chemotherapy | Leukaemia |
| NCT04494958 | NCT04494958-0 | Binimetinib | MEK | Breast cancer |
| NCT04494958 | NCT04494958-0 | Palbociclib | CDK4/CDK6 | Breast cancer |
| NCT04495179 | NCT04495179-1 | Imaradenant | A2aR/A2bR | Prostate cancer |
| NCT04495179 | NCT04495179-1 | Durvalumab | PD-L1 | Prostate cancer |
| NCT04495179 | NCT04495179-2 | Imaradenant | A2aR/A2bR | Prostate cancer |
| NCT04495179 | NCT04495179-2 | Durvalumab | PD-L1 | Prostate cancer |
| NCT04495179 | NCT04495179-2 | Cabazitaxel | chemotherapy | Prostate cancer |
| NCT04496999 | NCT04496999-0 | Midostaurin | PKC/PDGFR | Leukaemia |
| NCT04496999 | NCT04496999-0 | Siremadlin | p53/MDM2 | Leukaemia |
| NCT04497116 | NCT04497116-0 | Rp-3500 | ATR | Other solid tumors |
| NCT04497116 | NCT04497116-0 | Talazoparib | PARP | Other solid tumors |
| NCT04497844 | NCT04497844-0 | Niraparib | PARP | Prostate cancer |
| NCT04497844 | NCT04497844-0 | Abiraterone | CYP17A1 | Prostate cancer |
| NCT04497844 | NCT04497844-0 | Prednisone | Steroid Treatment | Prostate cancer |
| NCT04499053 | NCT04499053-1 | Durvalumab | PD-L1 | lung cancers |
| NCT04499053 | NCT04499053-1 | Carboplatin | chemotherapy | lung cancers |
| NCT04499053 | NCT04499053-1 | Paclitaxel | chemotherapy | lung cancers |
| NCT04499053 | NCT04499053-2 | Durvalumab | PD-L1 | lung cancers |
| NCT04499053 | NCT04499053-2 | Carboplatin | chemotherapy | lung cancers |
| NCT04499053 | NCT04499053-2 | Pemetrexed | chemotherapy | lung cancers |
| NCT04499924 | NCT04499924-0 | Tucatinib | HER2 | Stomach cancer |
| NCT04499924 | NCT04499924-0 | Trastuzumab | HER2 | Stomach cancer |
| NCT04499924 | NCT04499924-0 | Ramucirumab | KDR | Stomach cancer |
| NCT04499924 | NCT04499924-0 | Paclitaxel | chemotherapy | Stomach cancer |
| NCT04502394 | NCT04502394-0 | Navtemadlin | p53/MDM2 | Leukaemia |
| NCT04502394 | NCT04502394-0 | Acalabrutinib | BTK | Leukaemia |
| NCT04502602 | NCT04502602-0 | Neratinib | EGFR | Ovary cancer |
| NCT04502602 | NCT04502602-0 | Niraparib | PARP | Ovary cancer |
| NCT04504331 | NCT04504331-0 | Infigratinib | FGFR | Breast cancer |
| NCT04504331 | NCT04504331-0 | Tamoxifen | Endocrine Therapy | Breast cancer |
| NCT04504669 | NCT04504669-0 | Azd8701 | FOXP3 | Other solid tumors |
| NCT04504669 | NCT04504669-0 | Durvalumab | PD-L1 | Other solid tumors |
| NCT04505254 | NCT04505254-0 | Acalabrutinib | BTK | Leukaemia |
| NCT04505254 | NCT04505254-0 | Obinutuzumab | CD20 | Leukaemia |
| NCT04508647 | NCT04508647-0 | Ublituximab | CD20 | Lymphomas |
| NCT04508647 | NCT04508647-0 | Umbralisib | PI3K | Lymphomas |
| NCT04508803 | NCT04508803-0 | Hx008 | PD-1 | Breast cancer |
| NCT04508803 | NCT04508803-0 | Niraparib | PARP | Breast cancer |
| NCT04511013 | NCT04511013-0 | Encorafenib | RAF | Melanoma |
| NCT04511013 | NCT04511013-0 | Binimetinib | MEK | Melanoma |
| NCT04511013 | NCT04511013-0 | Nivolumab | PD-1 | Melanoma |
| NCT04511039 | NCT04511039-0 | Trifluridine/Tipiracil | chemotherapy | Other solid tumors |
| NCT04511039 | NCT04511039-0 | Talazoparib | PARP | Other solid tumors |
| NCT04512261 | NCT04512261-0 | Tucatinib | HER2 | Other solid tumors |
| NCT04512261 | NCT04512261-0 | Pembrolizumab | PD-1 | Other solid tumors |
| NCT04512261 | NCT04512261-0 | Trastuzumab | HER2 | Other solid tumors |
| NCT04513717 | NCT04513717-0 | Androgen Deprivation Therapy (Adt) | Endocrine Therapy | Prostate cancer |
| NCT04513717 | NCT04513717-0 | Apalutamide | Endocrine Therapy | Prostate cancer |
| NCT04513717 | NCT04513717-0 | Abiraterone | CYP17A1 | Prostate cancer |
| NCT04513717 | NCT04513717-0 | Prednisone | Steroid Treatment | Prostate cancer |
| NCT04513925 | NCT04513925-0 | Atezolizumab | PD-L1 | lung cancers |
| NCT04513925 | NCT04513925-0 | Tiragolumab | TIGIT | lung cancers |
| NCT04513951 | NCT04513951-0 | Mfolfoxiri | chemotherapy | Colon and rectum cancers |
| NCT04513951 | NCT04513951-0 | Cetuximab | EGFR | Colon and rectum cancers |
| NCT04513951 | NCT04513951-0 | Avelumab | PD-L1 | Colon and rectum cancers |
| NCT04514159 | NCT04514159-0 | Zn-C5 | Endocrine Therapy | Breast cancer |
| NCT04514159 | NCT04514159-0 | Abemaciclib | CDK4/CDK6 | Breast cancer |
| NCT04515238 | NCT04515238-0 | Bendamustine | chemotherapy | Leukaemia |
| NCT04515238 | NCT04515238-0 | Obinutuzumab | CD20 | Leukaemia |
| NCT04515238 | NCT04515238-0 | Zanubrutinib | BTK | Leukaemia |
| NCT04515238 | NCT04515238-0 | Venetoclax | BCL2 | Leukaemia |
| NCT04515394 | NCT04515394-0 | Tepotinib | MET | Colon and rectum cancers |
| NCT04515394 | NCT04515394-0 | Cetuximab | EGFR | Colon and rectum cancers |
| NCT04517526 | NCT04517526-0 | Chemotherapy | chemotherapy | lung cancers |
| NCT04517526 | NCT04517526-0 | Bevacizumab | VEGF | lung cancers |
| NCT04517526 | NCT04517526-0 | Durvalumab | PD-L1 | lung cancers |
| NCT04519476 | NCT04519476-0 | Selinexor | XPO1 | Multiple Myeloma |
| NCT04519476 | NCT04519476-0 | Lenalidomide | CRBN | Multiple Myeloma |
| NCT04522544 | NCT04522544-0 | Tremelimumab | CTLA-4 | Liver cancer |
| NCT04522544 | NCT04522544-0 | Durvalumab | PD-L1 | Liver cancer |
| NCT04523207 | NCT04523207-0 | Apalutamide | Endocrine Therapy | Prostate cancer |
| NCT04523207 | NCT04523207-0 | Androgen Deprivation Therapy (Adt) | Endocrine Therapy | Prostate cancer |
| NCT04523428 | NCT04523428-0 | Acalabrutinib | BTK | Leukaemia |
| NCT04523428 | NCT04523428-0 | Venetoclax | BCL2 | Leukaemia |
| NCT04523857 | NCT04523857-0 | Abemaciclib | CDK4/CDK6 | Breast cancer |
| NCT04523857 | NCT04523857-0 | Hydroxychloroquine | antimalarial agent | Breast cancer |
| NCT04524000 | NCT04524000-0 | Fulvestrant | Endocrine Therapy | Breast cancer |
| NCT04524000 | NCT04524000-0 | Alpelisib | PI3K | Breast cancer |
| NCT04525391 | NCT04525391-0 | Durvalumab | PD-L1 | lung cancers |
| NCT04525391 | NCT04525391-0 | Azd2811 | AURKB | lung cancers |
| NCT04525638 | NCT04525638-0 | Lutetium Lu 177 Dotatate | SSTR | Brain and nervous system cancers |
| NCT04525638 | NCT04525638-0 | Nivolumab | PD-1 | Brain and nervous system cancers |
| NCT04526470 | NCT04526470-0 | Alpelisib | PI3K | Stomach cancer |
| NCT04526470 | NCT04526470-0 | Paclitaxel | chemotherapy | Stomach cancer |
| NCT04526782 | NCT04526782-0 | Binimetinib | MEK | lung cancers |
| NCT04526782 | NCT04526782-0 | Encorafenib | RAF | lung cancers |
| NCT04526899 | NCT04526899-0 | Bnt111 | vaccine | Melanoma |
| NCT04526899 | NCT04526899-0 | Cemiplimab-Rwlc | PD-1 | Melanoma |
| NCT04529772 | NCT04529772-0 | Acalabrutinib | BTK | Lymphomas |
| NCT04529772 | NCT04529772-0 | Rituximab | CD20 | Lymphomas |
| NCT04529772 | NCT04529772-0 | Chemotherapy | chemotherapy | Lymphomas |
| NCT04534283 | NCT04534283-0 | Abemaciclib | CDK4/CDK6 | Others |
| NCT04534283 | NCT04534283-0 | Temuterkib | ERK | Others |
| NCT04535102 | NCT04535102-0 | Polatuzumab Vedotin | CD79B/Tubulin | Lymphomas |
| NCT04535102 | NCT04535102-0 | Bendamustine | chemotherapy | Lymphomas |
| NCT04535102 | NCT04535102-0 | Rituximab | CD20 | Lymphomas |
| NCT04538378 | NCT04538378-0 | Durvalumab | PD-L1 | Other solid tumors |
| NCT04538378 | NCT04538378-0 | Olaparib | PARP | Other solid tumors |
| NCT04538664 | NCT04538664-0 | Amivantamab | EGFR/MET | lung cancers |
| NCT04538664 | NCT04538664-0 | Platinum-Based Chemotherapy | chemotherapy | lung cancers |
| NCT04538664 | NCT04538664-0 | Pemetrexed | chemotherapy | lung cancers |
| NCT04538742 | NCT04538742-1 | Trastuzumab Deruxtecan | HER2/TOP1 | Breast cancer |
| NCT04538742 | NCT04538742-1 | Durvalumab | PD-L1 | Breast cancer |
| NCT04538742 | NCT04538742-2 | Trastuzumab Deruxtecan | HER2/TOP1 | Breast cancer |
| NCT04538742 | NCT04538742-2 | Pertuzumab | HER2 | Breast cancer |
| NCT04538742 | NCT04538742-3 | Trastuzumab Deruxtecan | HER2/TOP1 | Breast cancer |
| NCT04538742 | NCT04538742-3 | Paclitaxel | chemotherapy | Breast cancer |
| NCT04538742 | NCT04538742-4 | Trastuzumab Deruxtecan | HER2/TOP1 | Breast cancer |
| NCT04538742 | NCT04538742-4 | Durvalumab | PD-L1 | Breast cancer |
| NCT04538742 | NCT04538742-4 | Paclitaxel | chemotherapy | Breast cancer |
| NCT04538742 | NCT04538742-5 | Trastuzumab Deruxtecan | HER2/TOP1 | Breast cancer |
| NCT04538742 | NCT04538742-5 | Tucatinib | HER2 | Breast cancer |
| NCT04539938 | NCT04539938-0 | Tucatinib | HER2 | Breast cancer |
| NCT04539938 | NCT04539938-0 | Trastuzumab Deruxtecan | HER2/TOP1 | Breast cancer |
| NCT04541017 | NCT04541017-0 | Magrolimab | CD47 | Lymphomas |
| NCT04541017 | NCT04541017-0 | Mogamulizumab | CCR4 | Lymphomas |
| NCT04541407 | NCT04541407-1 | Temozolomide | chemotherapy | lung cancers |
| NCT04541407 | NCT04541407-1 | Osimertinib | EGFR | lung cancers |
| NCT04541407 | NCT04541407-2 | Temozolomide | chemotherapy | lung cancers |
| NCT04541407 | NCT04541407-2 | Lorlatinib | ALK | lung cancers |
| NCT04543071 | NCT04543071-0 | Motixafortide | CXCR4 | Pancreas cancer |
| NCT04543071 | NCT04543071-0 | Cemiplimab-Rwlc | PD-1 | Pancreas cancer |
| NCT04543071 | NCT04543071-0 | Gemcitabine | chemotherapy | Pancreas cancer |
| NCT04543071 | NCT04543071-0 | Paclitaxel | chemotherapy | Pancreas cancer |
| NCT04543188 | NCT04543188-1 | Pf-07284890 | RAF | Other solid tumors |
| NCT04543188 | NCT04543188-1 | Binimetinib | MEK | Other solid tumors |
| NCT04543188 | NCT04543188-2 | Pf-07284890 | RAF | Other solid tumors |
| NCT04543188 | NCT04543188-2 | Binimetinib | MEK | Other solid tumors |
| NCT04543188 | NCT04543188-2 | Midazolam | Other supportive therapy | Other solid tumors |
| NCT04543955 | NCT04543955-0 | Telotristat | Other supportive therapy | Brain and nervous system cancers |
| NCT04543955 | NCT04543955-0 | Lutetium Lu 177 Dotatate | SSTR | Brain and nervous system cancers |
| NCT04544189 | NCT04544189-0 | Alpelisib | PI3K | Breast cancer |
| NCT04544189 | NCT04544189-0 | Fulvestrant | Endocrine Therapy | Breast cancer |
| NCT04544995 | NCT04544995-0 | Niraparib | PARP | Others |
| NCT04544995 | NCT04544995-0 | Dostarlimab-Gxly | PD-1 | Others |
| NCT04545710 | NCT04545710-0 | Abemaciclib | CDK4/CDK6 | lung cancers |
| NCT04545710 | NCT04545710-0 | Osimertinib | EGFR | lung cancers |
| NCT04546620 | NCT04546620-0 | Acalabrutinib | BTK | Lymphomas |
| NCT04546620 | NCT04546620-0 | Rituximab | CD20 | Lymphomas |
| NCT04546620 | NCT04546620-0 | Chemotherapy | chemotherapy | Lymphomas |
| NCT04550260 | NCT04550260-1 | Durvalumab | PD-L1 | Other solid tumors |
| NCT04550260 | NCT04550260-1 | Cisplatin | chemotherapy | Other solid tumors |
| NCT04550260 | NCT04550260-1 | 5-Fu | chemotherapy | Other solid tumors |
| NCT04550260 | NCT04550260-2 | Durvalumab | PD-L1 | Other solid tumors |
| NCT04550260 | NCT04550260-2 | Cisplatin | chemotherapy | Other solid tumors |
| NCT04550260 | NCT04550260-2 | Capecitabine | chemotherapy | Other solid tumors |
| NCT04551495 | NCT04551495-0 | Letrozole | Endocrine Therapy | Breast cancer |
| NCT04551495 | NCT04551495-0 | Entrectinib | TRK | Breast cancer |
| NCT04551885 | NCT04551885-0 | Ft516 | cell therapy | Other solid tumors |
| NCT04551885 | NCT04551885-0 | Avelumab | PD-L1 | Other solid tumors |
| NCT04553770 | NCT04553770-0 | Trastuzumab Deruxtecan | HER2/TOP1 | Breast cancer |
| NCT04553770 | NCT04553770-0 | Anastrozole | Endocrine Therapy | Breast cancer |
| NCT04556071 | NCT04556071-0 | Niraparib | PARP | Other solid tumors |
| NCT04556071 | NCT04556071-0 | Bevacizumab | VEGF | Other solid tumors |
| NCT04556773 | NCT04556773-1 | Trastuzumab Deruxtecan | HER2/TOP1 | Breast cancer |
| NCT04556773 | NCT04556773-1 | Capecitabine | chemotherapy | Breast cancer |
| NCT04556773 | NCT04556773-2 | Trastuzumab Deruxtecan | HER2/TOP1 | Breast cancer |
| NCT04556773 | NCT04556773-2 | Durvalumab | PD-L1 | Breast cancer |
| NCT04556773 | NCT04556773-2 | Paclitaxel | chemotherapy | Breast cancer |
| NCT04556773 | NCT04556773-3 | Trastuzumab Deruxtecan | HER2/TOP1 | Breast cancer |
| NCT04556773 | NCT04556773-3 | Capivasertib | AKT | Breast cancer |
| NCT04556773 | NCT04556773-4 | Trastuzumab Deruxtecan | HER2/TOP1 | Breast cancer |
| NCT04556773 | NCT04556773-4 | Anastrozole | Endocrine Therapy | Breast cancer |
| NCT04556773 | NCT04556773-5 | Trastuzumab Deruxtecan | HER2/TOP1 | Breast cancer |
| NCT04556773 | NCT04556773-5 | Fulvestrant | Endocrine Therapy | Breast cancer |
| NCT04557059 | NCT04557059-0 | Lhrha | Endocrine Therapy | Prostate cancer |
| NCT04557059 | NCT04557059-0 | Apalutamide | Endocrine Therapy | Prostate cancer |
| NCT04557956 | NCT04557956-0 | Tazemetostat | EZH2 | Melanoma |
| NCT04557956 | NCT04557956-0 | Dabrafenib | RAF | Melanoma |
| NCT04557956 | NCT04557956-0 | Trametinib | MEK | Melanoma |
| NCT04558866 | NCT04558866-0 | Darolutamide | Endocrine Therapy | Prostate cancer |
| NCT04558866 | NCT04558866-0 | Testosterone Cypionate | Endocrine Therapy | Prostate cancer |
| NCT04558931 | NCT04558931-0 | Nk Cell | cell therapy | Multiple Myeloma |
| NCT04558931 | NCT04558931-0 | Isatuximab | CD38 | Multiple Myeloma |
| NCT04560166 | NCT04560166-1 | Naxitamab | GD-2 | Brain and nervous system cancers |
| NCT04560166 | NCT04560166-1 | Gm-Csf | CSF2 | Brain and nervous system cancers |
| NCT04560166 | NCT04560166-1 | Irinotecan | chemotherapy | Brain and nervous system cancers |
| NCT04560166 | NCT04560166-1 | Temozolomide | chemotherapy | Brain and nervous system cancers |
| NCT04560166 | NCT04560166-2 | Irinotecan | chemotherapy | Brain and nervous system cancers |
| NCT04560166 | NCT04560166-2 | Temozolomide | chemotherapy | Brain and nervous system cancers |
| NCT04561336 | NCT04561336-0 | Avelumab | PD-L1 | Colon and rectum cancers |
| NCT04561336 | NCT04561336-0 | Cetuximab | EGFR | Colon and rectum cancers |
| NCT04562389 | NCT04562389-0 | Selinexor | XPO1 | Other hematologic Neoplasms |
| NCT04562389 | NCT04562389-0 | Ruxolitinib | JAK | Other hematologic Neoplasms |
| NCT04562441 | NCT04562441-0 | Axitinib | VEGFR/PDGFR | Mouth and oropharynx cancers |
| NCT04562441 | NCT04562441-0 | Avelumab | PD-L1 | Mouth and oropharynx cancers |
| NCT04565054 | NCT04565054-0 | Abemaciclib | CDK4/CDK6 | Breast cancer |
| NCT04565054 | NCT04565054-0 | Endocrine Therapy | Endocrine Therapy | Breast cancer |
| NCT04566887 | NCT04566887-0 | Acalabrutinib | BTK | Lymphomas |
| NCT04566887 | NCT04566887-0 | Rituximab | CD20 | Lymphomas |
| NCT04566887 | NCT04566887-0 | Chemotherapy | chemotherapy | Lymphomas |
| NCT04568200 | NCT04568200-0 | Durvalumab | PD-L1 | Other solid tumors |
| NCT04568200 | NCT04568200-0 | Platinum-Based Chemotherapy | chemotherapy | Other solid tumors |
| NCT04568200 | NCT04568200-0 | Paclitaxel | chemotherapy | Other solid tumors |
| NCT04571632 | NCT04571632-0 | Avelumab | PD-L1 | lung cancers |
| NCT04571632 | NCT04571632-0 | Ipilimumab | CTLA-4 | lung cancers |
| NCT04571632 | NCT04571632-0 | Pembrolizumab | PD-1 | lung cancers |
| NCT04572763 | NCT04572763-0 | Copanlisib | PI3K | Lymphomas |
| NCT04572763 | NCT04572763-0 | Venetoclax | BCL2 | Lymphomas |
| NCT04576117 | NCT04576117-0 | Selumetinib | MEK | Brain and nervous system cancers |
| NCT04576117 | NCT04576117-0 | Vinblastine | chemotherapy | Brain and nervous system cancers |
| NCT04577833 | NCT04577833-0 | Niraparib | PARP | Prostate cancer |
| NCT04577833 | NCT04577833-0 | Abiraterone | CYP17A1 | Prostate cancer |
| NCT04577833 | NCT04577833-0 | Prednisone | Steroid Treatment | Prostate cancer |
| NCT04579380 | NCT04579380-0 | Tucatinib | HER2 | Other solid tumors |
| NCT04579380 | NCT04579380-0 | Trastuzumab | HER2 | Other solid tumors |
| NCT04579380 | NCT04579380-0 | Fulvestrant | Endocrine Therapy | Other solid tumors |
| NCT04581824 | NCT04581824-0 | Dostarlimab-Gxly | PD-1 | lung cancers |
| NCT04581824 | NCT04581824-0 | Pemetrexed | chemotherapy | lung cancers |
| NCT04581824 | NCT04581824-0 | Platinum-Based Chemotherapy | chemotherapy | lung cancers |
| NCT04584255 | NCT04584255-0 | Niraparib | PARP | Breast cancer |
| NCT04584255 | NCT04584255-0 | Dostarlimab-Gxly | PD-1 | Breast cancer |
| NCT04584853 | NCT04584853-0 | Abemaciclib | CDK4/CDK6 | Breast cancer |
| NCT04584853 | NCT04584853-0 | Endocrine Therapy | Endocrine Therapy | Breast cancer |
| NCT04585490 | NCT04585490-1 | Durvalumab | PD-L1 | lung cancers |
| NCT04585490 | NCT04585490-1 | Carboplatin | chemotherapy | lung cancers |
| NCT04585490 | NCT04585490-1 | Pemetrexed | chemotherapy | lung cancers |
| NCT04585490 | NCT04585490-2 | Durvalumab | PD-L1 | lung cancers |
| NCT04585490 | NCT04585490-2 | Carboplatin | chemotherapy | lung cancers |
| NCT04585490 | NCT04585490-2 | Paclitaxel | chemotherapy | lung cancers |
| NCT04585815 | NCT04585815-0 | Sasanlimab | PD-1 | lung cancers |
| NCT04585815 | NCT04585815-0 | Encorafenib | RAF | lung cancers |
| NCT04585815 | NCT04585815-0 | Binimetinib | MEK | lung cancers |
| NCT04585958 | NCT04585958-0 | Trastuzumab Deruxtecan | HER2/TOP1 | Other solid tumors |
| NCT04585958 | NCT04585958-0 | Olaparib | PARP | Other solid tumors |
| NCT04586231 | NCT04586231-0 | Belzutifan | HIF-2 | Kidney cancer |
| NCT04586231 | NCT04586231-0 | Lenvatinib | VEGFR/PDGFR/FGFR | Kidney cancer |
| NCT04586244 | NCT04586244-1 | Epacadostat | IDO1 | Bladder cancer |
| NCT04586244 | NCT04586244-1 | Retifanlimab | PD-1 | Bladder cancer |
| NCT04586244 | NCT04586244-2 | Incagn02385 | LAG-3 | Bladder cancer |
| NCT04586244 | NCT04586244-2 | Retifanlimab | PD-1 | Bladder cancer |
| NCT04586244 | NCT04586244-3 | Retifanlimab | PD-1 | Bladder cancer |
| NCT04586244 | NCT04586244-3 | Incagn02385 | LAG-3 | Bladder cancer |
| NCT04586244 | NCT04586244-3 | Incagn02390 | TIM3 | Bladder cancer |
| NCT04589832 | NCT04589832-0 | Pac-1 | CASP3 | Melanoma |
| NCT04589832 | NCT04589832-0 | Entrectinib | TRK | Melanoma |
| NCT04590326 | NCT04590326-0 | Regn5668 | CD28/MUC16 | Ovary cancer |
| NCT04590326 | NCT04590326-0 | Cemiplimab-Rwlc | PD-1 | Ovary cancer |
| NCT04592237 | NCT04592237-0 | Niraparib | PARP | Other solid tumors |
| NCT04592237 | NCT04592237-0 | Cetrelimab | PD-1 | Other solid tumors |
| NCT04592913 | NCT04592913-0 | Durvalumab | PD-L1 | Stomach cancer |
| NCT04592913 | NCT04592913-0 | Flot | chemotherapy | Stomach cancer |
| NCT04594005 | NCT04594005-0 | Abemaciclib | CDK4/CDK6 | Others |
| NCT04594005 | NCT04594005-0 | Paclitaxel | chemotherapy | Others |
| NCT04594798 | NCT04594798-0 | Polatuzumab Vedotin | CD79B/Tubulin | Lymphomas |
| NCT04594798 | NCT04594798-0 | Rituximab | CD20 | Lymphomas |
| NCT04594798 | NCT04594798-0 | Chemotherapy | chemotherapy | Lymphomas |
| NCT04595994 | NCT04595994-0 | Selinexor | XPO1 | Other solid tumors |
| NCT04595994 | NCT04595994-0 | Gemcitabine | chemotherapy | Other solid tumors |
| NCT04598009 | NCT04598009-0 | Binimetinib | MEK | Melanoma |
| NCT04598009 | NCT04598009-0 | Imatinib | BCR-ABL | Melanoma |
| NCT04601402 | NCT04601402-0 | Gen-001 | gene therapy | Other solid tumors |
| NCT04601402 | NCT04601402-0 | Avelumab | PD-L1 | Other solid tumors |
| NCT04602533 | NCT04602533-1 | Platinum-Based Chemotherapy | chemotherapy | lung cancers |
| NCT04602533 | NCT04602533-1 | Durvalumab | PD-L1 | lung cancers |
| NCT04602533 | NCT04602533-2 | Etoposide | chemotherapy | lung cancers |
| NCT04602533 | NCT04602533-2 | Durvalumab | PD-L1 | lung cancers |
| NCT04603183 | NCT04603183-0 | Abemaciclib | CDK4/CDK6 | Breast cancer |
| NCT04603183 | NCT04603183-0 | Letrozole | Endocrine Therapy | Breast cancer |
| NCT04603183 | NCT04603183-0 | Fulvestrant | Endocrine Therapy | Breast cancer |
| NCT04604067 | NCT04604067-0 | Acalabrutinib | BTK | Lymphomas |
| NCT04604067 | NCT04604067-0 | Rituximab | CD20 | Lymphomas |
| NCT04604067 | NCT04604067-0 | Cyclophosphamide | chemotherapy | Lymphomas |
| NCT04604067 | NCT04604067-0 | Doxorubicin | chemotherapy | Lymphomas |
| NCT04604067 | NCT04604067-0 | Vincristine | chemotherapy | Lymphomas |
| NCT04604067 | NCT04604067-0 | Prednisone | Steroid Treatment | Lymphomas |
| NCT04606381 | NCT04606381-1 | Amivantamab | EGFR/MET | Other solid tumors |
| NCT04606381 | NCT04606381-1 | Hyaluronidase | Other supportive therapy | Other solid tumors |
| NCT04606381 | NCT04606381-2 | Amivantamab | EGFR/MET | Other solid tumors |
| NCT04606381 | NCT04606381-2 | Hyaluronidase | Other supportive therapy | Other solid tumors |
| NCT04606381 | NCT04606381-2 | Lazertinib | EGFR | Other solid tumors |
| NCT04607421 | NCT04607421-0 | Encorafenib | RAF | Others |
| NCT04607421 | NCT04607421-0 | Cetuximab | EGFR | Others |
| NCT04607421 | NCT04607421-0 | Chemotherapy | chemotherapy | Others |
| NCT04607954 | NCT04607954-0 | Durvalumab | PD-L1 | lung cancers |
| NCT04607954 | NCT04607954-0 | Topotecan | chemotherapy | lung cancers |
| NCT04610658 | NCT04610658-0 | Nivolumab | PD-1 | lung cancers |
| NCT04610658 | NCT04610658-0 | Ipilimumab | CTLA-4 | lung cancers |
| NCT04610658 | NCT04610658-0 | Lurbinectedin | chemotherapy | lung cancers |
| NCT04612751 | NCT04612751-0 | Datopotamab Deruxtecan | Trop-2/TOP1 | lung cancers |
| NCT04612751 | NCT04612751-0 | Durvalumab | PD-L1 | lung cancers |
| NCT04612751 | NCT04612751-0 | Chemotherapy | chemotherapy | lung cancers |
| NCT04613284 | NCT04613284-0 | Endostatin | Endostatin | lung cancers |
| NCT04613284 | NCT04613284-0 | Durvalumab | PD-L1 | lung cancers |
| NCT04613492 | NCT04613492-0 | Medi9253 | oncolytic virus | Other solid tumors |
| NCT04613492 | NCT04613492-0 | Durvalumab | PD-L1 | Other solid tumors |
| NCT04614194 | NCT04614194-0 | Abemaciclib | CDK4/CDK6 | Breast cancer |
| NCT04614194 | NCT04614194-0 | Letrozole | Endocrine Therapy | Breast cancer |
| NCT04616183 | NCT04616183-0 | Temuterkib | ERK | Other solid tumors |
| NCT04616183 | NCT04616183-0 | Cetuximab | EGFR | Other solid tumors |
| NCT04616183 | NCT04616183-0 | Abemaciclib | CDK4/CDK6 | Other solid tumors |
| NCT04617756 | NCT04617756-1 | Durvalumab | PD-L1 | Bladder cancer |
| NCT04617756 | NCT04617756-1 | Gemcitabine | chemotherapy | Bladder cancer |
| NCT04617756 | NCT04617756-2 | Durvalumab | PD-L1 | Bladder cancer |
| NCT04617756 | NCT04617756-2 | Platinum-Based Chemotherapy | chemotherapy | Bladder cancer |
| NCT04621370 | NCT04621370-0 | Durvalumab | PD-L1 | Colon and rectum cancers |
| NCT04621370 | NCT04621370-0 | Folfox | chemotherapy | Colon and rectum cancers |
| NCT04624113 | NCT04624113-0 | Tazemetostat | EZH2 | Head and Neck Neoplasms |
| NCT04624113 | NCT04624113-0 | Pembrolizumab | PD-1 | Head and Neck Neoplasms |
| NCT04624633 | NCT04624633-0 | Acalabrutinib | BTK | Leukaemia |
| NCT04624633 | NCT04624633-0 | Umbralisib | PI3K | Leukaemia |
| NCT04624633 | NCT04624633-0 | Ublituximab | CD20 | Leukaemia |
| NCT04624906 | NCT04624906-0 | Bendamustine | chemotherapy | Lymphomas |
| NCT04624906 | NCT04624906-0 | Rituximab | CD20 | Lymphomas |
| NCT04624906 | NCT04624906-0 | Acalabrutinib | BTK | Lymphomas |
| NCT04624958 | NCT04624958-0 | Zanubrutinib | BTK | Lymphomas |
| NCT04624958 | NCT04624958-0 | Rituximab | CD20 | Lymphomas |
| NCT04624958 | NCT04624958-0 | Chemotherapy | chemotherapy | Lymphomas |
| NCT04625699 | NCT04625699-0 | Durvalumab | PD-L1 | lung cancers |
| NCT04625699 | NCT04625699-0 | Tremelimumab | CTLA-4 | lung cancers |
| NCT04626479 | NCT04626479-1 | Pembrolizumab | PD-1 | Kidney cancer |
| NCT04626479 | NCT04626479-1 | Lenvatinib | VEGFR/PDGFR/FGFR | Kidney cancer |
| NCT04626479 | NCT04626479-2 | Quavonlimab | CTLA-4 | Kidney cancer |
| NCT04626479 | NCT04626479-2 | Lenvatinib | VEGFR/PDGFR/FGFR | Kidney cancer |
| NCT04626479 | NCT04626479-3 | Favezelimab | LAG-3 | Kidney cancer |
| NCT04626479 | NCT04626479-3 | Lenvatinib | VEGFR/PDGFR/FGFR | Kidney cancer |
| NCT04626479 | NCT04626479-4 | Pembrolizumab | PD-1 | Kidney cancer |
| NCT04626479 | NCT04626479-4 | Belzutifan | HIF-2 | Kidney cancer |
| NCT04626479 | NCT04626479-4 | Lenvatinib | VEGFR/PDGFR/FGFR | Kidney cancer |
| NCT04626518 | NCT04626518-1 | Belzutifan | HIF-2 | Kidney cancer |
| NCT04626518 | NCT04626518-1 | Lenvatinib | VEGFR/PDGFR/FGFR | Kidney cancer |
| NCT04626518 | NCT04626518-2 | Pembrolizumab | PD-1 | Kidney cancer |
| NCT04626518 | NCT04626518-2 | Lenvatinib | VEGFR/PDGFR/FGFR | Kidney cancer |
| NCT04626635 | NCT04626635-0 | Regn7075 | EGFR/CD28 | Other solid tumors |
| NCT04626635 | NCT04626635-0 | Cemiplimab-Rwlc | PD-1 | Other solid tumors |
| NCT04626791 | NCT04626791-0 | Acalabrutinib | BTK | Lymphomas |
| NCT04626791 | NCT04626791-0 | Rituximab | CD20 | Lymphomas |
| NCT04626791 | NCT04626791-0 | Chemotherapy | chemotherapy | Lymphomas |
| NCT04627064 | NCT04627064-0 | Abemaciclib | CDK4/CDK6 | Kidney cancer |
| NCT04627064 | NCT04627064-0 | Belzutifan | HIF-2 | Kidney cancer |
| NCT04628767 | NCT04628767-0 | Durvalumab | PD-L1 | Bladder cancer |
| NCT04628767 | NCT04628767-0 | Chemotherapy | chemotherapy | Bladder cancer |
| NCT04630756 | NCT04630756-0 | Azd4573 | CDK9 | Other hematologic Neoplasms |
| NCT04630756 | NCT04630756-0 | Acalabrutinib | BTK | Other hematologic Neoplasms |
| NCT04632992 | NCT04632992-1 | Trastuzumab Emtansine | HER2/Tubulin | Other solid tumors |
| NCT04632992 | NCT04632992-1 | Atezolizumab | PD-L1 | Other solid tumors |
| NCT04632992 | NCT04632992-2 | Pertuzumab | HER2 | Other solid tumors |
| NCT04632992 | NCT04632992-2 | Trastuzumab | HER2 | Other solid tumors |
| NCT04632992 | NCT04632992-2 | Hyaluronidase | Other supportive therapy | Other solid tumors |
| NCT04632992 | NCT04632992-3 | Trastuzumab Emtansine | HER2/Tubulin | Other solid tumors |
| NCT04632992 | NCT04632992-3 | Tucatinib | HER2 | Other solid tumors |
| NCT04632992 | NCT04632992-4 | Ipatasertib | AKT | Other solid tumors |
| NCT04632992 | NCT04632992-4 | Atezolizumab | PD-L1 | Other solid tumors |
| NCT04632992 | NCT04632992-5 | Ipatasertib | AKT | Other solid tumors |
| NCT04632992 | NCT04632992-5 | Paclitaxel | chemotherapy | Other solid tumors |
| NCT04632992 | NCT04632992-6 | Atezolizumab | PD-L1 | Other solid tumors |
| NCT04632992 | NCT04632992-6 | Tiragolumab | TIGIT | Other solid tumors |
| NCT04633122 | NCT04633122-0 | Ripretinib | VEGFR/PDGFR/RAF | Other solid tumors |
| NCT04633122 | NCT04633122-0 | Sunitinib | VEGFR/PDGFR | Other solid tumors |
| NCT04633239 | NCT04633239-0 | Abemaciclib | CDK4/CDK6 | Ovary cancer |
| NCT04633239 | NCT04633239-0 | Olaparib | PARP | Ovary cancer |
| NCT04639843 | NCT04639843-0 | Doxorubicin | chemotherapy | Other hematologic Neoplasms |
| NCT04639843 | NCT04639843-0 | Azacitidine | chemotherapy | Other hematologic Neoplasms |
| NCT04639843 | NCT04639843-0 | Romidepsin | chemotherapy | Other hematologic Neoplasms |
| NCT04639843 | NCT04639843-0 | Duvelisib | PI3K | Other hematologic Neoplasms |
| NCT04640779 | NCT04640779-0 | Selinexor | XPO1 | Lymphomas |
| NCT04640779 | NCT04640779-0 | Choline Salicylate | PTGS | Lymphomas |
| NCT04643002 | NCT04643002-1 | Isatuximab | CD38 | Lymphomas |
| NCT04643002 | NCT04643002-1 | Dexamethasone | Steroid Treatment | Lymphomas |
| NCT04643002 | NCT04643002-2 | Isatuximab | CD38 | Lymphomas |
| NCT04643002 | NCT04643002-2 | Pomalidomide | CRBN | Lymphomas |
| NCT04643002 | NCT04643002-2 | Dexamethasone | Steroid Treatment | Lymphomas |
| NCT04644289 | NCT04644289-0 | Olaparib | PARP | Ovary cancer |
| NCT04644289 | NCT04644289-0 | Durvalumab | PD-L1 | Ovary cancer |
| NCT04646005 | NCT04646005-0 | Cemiplimab-Rwlc | PD-1 | Cervix uteri cancer |
| NCT04646005 | NCT04646005-0 | Isa101B | vaccine | Cervix uteri cancer |
| NCT04646395 | NCT04646395-0 | Acalabrutinib | BTK | Lymphomas |
| NCT04646395 | NCT04646395-0 | Tafasitamab | CD19 | Lymphomas |
| NCT04646837 | NCT04646837-0 | Durvalumab | PD-L1 | lung cancers |
| NCT04646837 | NCT04646837-0 | Paclitaxel | chemotherapy | lung cancers |
| NCT04646837 | NCT04646837-0 | Platinum-Based Chemotherapy | chemotherapy | lung cancers |
| NCT04653246 | NCT04653246-0 | Isatuximab | CD38 | Multiple Myeloma |
| NCT04653246 | NCT04653246-0 | Lenalidomide | CRBN | Multiple Myeloma |
| NCT04653246 | NCT04653246-0 | Bortezomib | PSMB | Multiple Myeloma |
| NCT04653246 | NCT04653246-0 | Dexamethasone | Steroid Treatment | Multiple Myeloma |
| NCT04655157 | NCT04655157-0 | Encorafenib | RAF | Melanoma |
| NCT04655157 | NCT04655157-0 | Binimetinib | MEK | Melanoma |
| NCT04655157 | NCT04655157-0 | Nivolumab | PD-1 | Melanoma |
| NCT04655157 | NCT04655157-0 | Ipilimumab | CTLA-4 | Melanoma |
| NCT04655183 | NCT04655183-1 | Niraparib | PARP | Breast cancer |
| NCT04655183 | NCT04655183-1 | M4344 | ATR | Breast cancer |
| NCT04655183 | NCT04655183-2 | Niraparib | PARP | Breast cancer |
| NCT04655183 | NCT04655183-2 | M4344 | ATR | Breast cancer |
| NCT04655391 | NCT04655391-1 | Glasdegib | SMO | Leukaemia |
| NCT04655391 | NCT04655391-1 | Decitabine | chemotherapy | Leukaemia |
| NCT04655391 | NCT04655391-1 | Venetoclax | BCL2 | Leukaemia |
| NCT04655391 | NCT04655391-2 | Glasdegib | SMO | Leukaemia |
| NCT04655391 | NCT04655391-2 | Gilteritinib | FLT3 | Leukaemia |
| NCT04655391 | NCT04655391-3 | Glasdegib | SMO | Leukaemia |
| NCT04655391 | NCT04655391-3 | Bosutinib | BCR-ABL | Leukaemia |
| NCT04655391 | NCT04655391-4 | Glasdegib | SMO | Leukaemia |
| NCT04655391 | NCT04655391-4 | Ivosidenib | IDH | Leukaemia |
| NCT04655391 | NCT04655391-5 | Glasdegib | SMO | Leukaemia |
| NCT04655391 | NCT04655391-5 | Enasidenib | IDH | Leukaemia |
| NCT04655976 | NCT04655976-1 | Cobolimab | TIM3 | lung cancers |
| NCT04655976 | NCT04655976-1 | Dostarlimab-Gxly | PD-1 | lung cancers |
| NCT04655976 | NCT04655976-1 | Docetaxel | chemotherapy | lung cancers |
| NCT04655976 | NCT04655976-2 | Dostarlimab-Gxly | PD-1 | lung cancers |
| NCT04655976 | NCT04655976-2 | Docetaxel | chemotherapy | lung cancers |
| NCT04657991 | NCT04657991-0 | Encorafenib | RAF | Melanoma |
| NCT04657991 | NCT04657991-0 | Binimetinib | MEK | Melanoma |
| NCT04657991 | NCT04657991-0 | Pembrolizumab | PD-1 | Melanoma |
| NCT04659044 | NCT04659044-0 | Polatuzumab Vedotin | CD79B/Tubulin | Leukaemia |
| NCT04659044 | NCT04659044-0 | Venetoclax | BCL2 | Leukaemia |
| NCT04659044 | NCT04659044-0 | Rituximab | CD20 | Leukaemia |
| NCT04659044 | NCT04659044-0 | Hyaluronidase | Other supportive therapy | Leukaemia |
| NCT04659616 | NCT04659616-0 | Pemigatinib | FGFR | Leukaemia |
| NCT04659616 | NCT04659616-0 | Chemotherapy | chemotherapy | Leukaemia |
| NCT04660097 | NCT04660097-0 | Anlotinib | VEGFR/PDGFR/FGFR | lung cancers |
| NCT04660097 | NCT04660097-0 | Durvalumab | PD-L1 | lung cancers |
| NCT04660097 | NCT04660097-0 | Chemotherapy | chemotherapy | lung cancers |
| NCT04661007 | NCT04661007-1 | Tafasitamab | CD19 | Lymphomas |
| NCT04661007 | NCT04661007-1 | Lenalidomide | CRBN | Lymphomas |
| NCT04661007 | NCT04661007-1 | Parsaclisib | PI3K | Lymphomas |
| NCT04661007 | NCT04661007-2 | Tafasitamab | CD19 | Lymphomas |
| NCT04661007 | NCT04661007-2 | Parsaclisib | PI3K | Lymphomas |
| NCT04661137 | NCT04661137-1 | Selinexor | XPO1 | Multiple Myeloma |
| NCT04661137 | NCT04661137-1 | Carfilzomib | PSMB | Multiple Myeloma |
| NCT04661137 | NCT04661137-1 | Daratumumab | CD38 | Multiple Myeloma |
| NCT04661137 | NCT04661137-2 | Selinexor | XPO1 | Multiple Myeloma |
| NCT04661137 | NCT04661137-2 | Carfilzomib | PSMB | Multiple Myeloma |
| NCT04661137 | NCT04661137-2 | Pomalidomide | CRBN | Multiple Myeloma |
| NCT04665765 | NCT04665765-0 | Polatuzumab Vedotin | CD79B/Tubulin | Lymphomas |
| NCT04665765 | NCT04665765-0 | Rituximab | CD20 | Lymphomas |
| NCT04665765 | NCT04665765-0 | Ifosfamide | Chemotherapy | Lymphomas |
| NCT04665765 | NCT04665765-0 | Platinum-Based Chemotherapy | chemotherapy | Lymphomas |
| NCT04665765 | NCT04665765-0 | Etoposide | chemotherapy | Lymphomas |
| NCT04666129 | NCT04666129-0 | Relugolix | Endocrine Therapy | Prostate cancer |
| NCT04666129 | NCT04666129-0 | Abiraterone | CYP17A1 | Prostate cancer |
| NCT04666129 | NCT04666129-0 | Prednisone | Steroid Treatment | Prostate cancer |
| NCT04668300 | NCT04668300-0 | Oleclumab | CD73 | Other solid tumors |
| NCT04668300 | NCT04668300-0 | Durvalumab | PD-L1 | Other solid tumors |
| NCT04668365 | NCT04668365-0 | Zanubrutinib | BTK | Lymphomas |
| NCT04668365 | NCT04668365-0 | Chemotherapy | chemotherapy | Lymphomas |
| NCT04673448 | NCT04673448-0 | Niraparib | PARP | Others |
| NCT04673448 | NCT04673448-0 | Dostarlimab-Gxly | PD-1 | Others |
| NCT04676243 | NCT04676243-0 | Quizartinib | FLT3 | Leukaemia |
| NCT04676243 | NCT04676243-0 | Chemotherapy | chemotherapy | Leukaemia |
| NCT04678362 | NCT04678362-0 | Avelumab | PD-L1 | Bladder cancer |
| NCT04678362 | NCT04678362-0 | Talazoparib | PARP | Bladder cancer |
| NCT04679012 | NCT04679012-0 | Polatuzumab Vedotin | CD79B/Tubulin | Leukaemia |
| NCT04679012 | NCT04679012-0 | Rituximab | CD20 | Leukaemia |
| NCT04679012 | NCT04679012-0 | Chemotherapy | chemotherapy | Leukaemia |
| NCT04679064 | NCT04679064-0 | Niraparib | PARP | Ovary cancer |
| NCT04679064 | NCT04679064-0 | Dostarlimab-Gxly | PD-1 | Ovary cancer |
| NCT04679480 | NCT04679480-0 | Cemiplimab-Rwlc | PD-1 | Other solid tumors |
| NCT04679480 | NCT04679480-0 | Sonidegib | SMO | Other solid tumors |
| NCT04680052 | NCT04680052-0 | Tafasitamab | CD19 | Lymphomas |
| NCT04680052 | NCT04680052-0 | Rituximab | CD20 | Lymphomas |
| NCT04680052 | NCT04680052-0 | Lenalidomide | CRBN | Lymphomas |
| NCT04685915 | NCT04685915-0 | Ibrutinib | BTK | Leukaemia |
| NCT04685915 | NCT04685915-0 | Copanlisib | PI3K | Leukaemia |
| NCT04686305 | NCT04686305-1 | Trastuzumab Deruxtecan | HER2/TOP1 | lung cancers |
| NCT04686305 | NCT04686305-1 | Durvalumab | PD-L1 | lung cancers |
| NCT04686305 | NCT04686305-1 | Platinum-Based Chemotherapy | chemotherapy | lung cancers |
| NCT04686305 | NCT04686305-2 | Trastuzumab Deruxtecan | HER2/TOP1 | lung cancers |
| NCT04686305 | NCT04686305-2 | Durvalumab | PD-L1 | lung cancers |
| NCT04686305 | NCT04686305-2 | Platinum-Based Chemotherapy | chemotherapy | lung cancers |
| NCT04686305 | NCT04686305-2 | Pemetrexed | chemotherapy | lung cancers |
| NCT04688151 | NCT04688151-0 | Rituximab | CD20 | Lymphomas |
| NCT04688151 | NCT04688151-0 | Acalabrutinib | BTK | Lymphomas |
| NCT04688151 | NCT04688151-0 | Durvalumab | PD-L1 | Lymphomas |
| NCT04688658 | NCT04688658-0 | Duvelisib | PI3K | Melanoma |
| NCT04688658 | NCT04688658-0 | Nivolumab | PD-1 | Melanoma |
| NCT04690855 | NCT04690855-0 | Atezolizumab | PD-L1 | Breast cancer |
| NCT04690855 | NCT04690855-0 | Talazoparib | PARP | Breast cancer |
| NCT04692155 | NCT04692155-0 | Ublituximab | CD20 | Lymphomas |
| NCT04692155 | NCT04692155-0 | Umbralisib | PI3K | Lymphomas |
| NCT04693468 | NCT04693468-1 | Talazoparib | PARP | Other solid tumors |
| NCT04693468 | NCT04693468-1 | Palbociclib | CDK4/CDK6 | Other solid tumors |
| NCT04693468 | NCT04693468-1 | Axitinib | VEGFR/PDGFR | Other solid tumors |
| NCT04693468 | NCT04693468-2 | Talazoparib | PARP | Other solid tumors |
| NCT04693468 | NCT04693468-2 | Palbociclib | CDK4/CDK6 | Other solid tumors |
| NCT04693468 | NCT04693468-2 | Crizotinib | ALK/MET | Other solid tumors |
| NCT04696848 | NCT04696848-0 | Valecobulin | chemotherapy | Colon and rectum cancers |
| NCT04696848 | NCT04696848-0 | Durvalumab | PD-L1 | Colon and rectum cancers |
| NCT04698213 | NCT04698213-0 | Axitinib | VEGFR/PDGFR | Kidney cancer |
| NCT04698213 | NCT04698213-0 | Avelumab | PD-L1 | Kidney cancer |
| NCT04699838 | NCT04699838-0 | Platinum-Based Chemotherapy | chemotherapy | lung cancers |
| NCT04699838 | NCT04699838-0 | Etoposide | chemotherapy | lung cancers |
| NCT04699838 | NCT04699838-0 | Durvalumab | PD-L1 | lung cancers |
| NCT04699838 | NCT04699838-0 | Ceralasertib | ATR | lung cancers |
| NCT04700124 | NCT04700124-0 | Enfortumab Vedotin | NECTIN4/Tubulin | Gallbladder and biliary tract cancer |
| NCT04700124 | NCT04700124-0 | Pembrolizumab | PD-1 | Gallbladder and biliary tract cancer |
| NCT04701307 | NCT04701307-0 | Niraparib | PARP | Other solid tumors |
| NCT04701307 | NCT04701307-0 | Dostarlimab-Gxly | PD-1 | Other solid tumors |
| NCT04703920 | NCT04703920-0 | Talazoparib | PARP | Other solid tumors |
| NCT04703920 | NCT04703920-0 | Belinostat | chemotherapy | Other solid tumors |
| NCT04704661 | NCT04704661-0 | Trastuzumab Deruxtecan | HER2/TOP1 | Other solid tumors |
| NCT04704661 | NCT04704661-0 | Ceralasertib | ATR | Other solid tumors |
| NCT04705129 | NCT04705129-0 | Zanubrutinib | BTK | Lymphomas |
| NCT04705129 | NCT04705129-0 | Tislelizumab | PD-1 | Lymphomas |
| NCT04705818 | NCT04705818-0 | Durvalumab | PD-L1 | Other solid tumors |
| NCT04705818 | NCT04705818-0 | Tazemetostat | EZH2 | Other solid tumors |
| NCT04707196 | NCT04707196-1 | Abemaciclib | CDK4/CDK6 | Breast cancer |
| NCT04707196 | NCT04707196-1 | Nsai | Endocrine Therapy | Breast cancer |
| NCT04707196 | NCT04707196-2 | Abemaciclib | CDK4/CDK6 | Breast cancer |
| NCT04707196 | NCT04707196-2 | Fulvestrant | Endocrine Therapy | Breast cancer |
| NCT04711824 | NCT04711824-0 | Olaparib | PARP | Breast cancer |
| NCT04711824 | NCT04711824-0 | Durvalumab | PD-L1 | Breast cancer |
| NCT04712903 | NCT04712903-0 | Durvalumab | PD-L1 | lung cancers |
| NCT04712903 | NCT04712903-0 | Platinum-Based Chemotherapy | chemotherapy | lung cancers |
| NCT04712903 | NCT04712903-0 | Etoposide | chemotherapy | lung cancers |
| NCT04716075 | NCT04716075-0 | Acalabrutinib | BTK | Leukaemia |
| NCT04716075 | NCT04716075-0 | Allosct | cell therapy | Leukaemia |
| NCT04717700 | NCT04717700-1 | Selinexor | XPO1 | Multiple Myeloma |
| NCT04717700 | NCT04717700-1 | Bortezomib | PSMB | Multiple Myeloma |
| NCT04717700 | NCT04717700-1 | Dexamethasone | Steroid Treatment | Multiple Myeloma |
| NCT04717700 | NCT04717700-2 | Selinexor | XPO1 | Multiple Myeloma |
| NCT04717700 | NCT04717700-2 | Lenalidomide | CRBN | Multiple Myeloma |
| NCT04717700 | NCT04717700-2 | Dexamethasone | Steroid Treatment | Multiple Myeloma |
| NCT04720768 | NCT04720768-0 | Encorafenib | RAF | Melanoma |
| NCT04720768 | NCT04720768-0 | Binimetinib | MEK | Melanoma |
| NCT04720768 | NCT04720768-0 | Palbociclib | CDK4/CDK6 | Melanoma |
| NCT04720976 | NCT04720976-1 | Jab-3312 | SHP2 | Other solid tumors |
| NCT04720976 | NCT04720976-1 | Pembrolizumab | PD-1 | Other solid tumors |
| NCT04720976 | NCT04720976-2 | Jab-3312 | SHP2 | Other solid tumors |
| NCT04720976 | NCT04720976-2 | Binimetinib | MEK | Other solid tumors |
| NCT04720976 | NCT04720976-3 | Jab-3312 | SHP2 | Other solid tumors |
| NCT04720976 | NCT04720976-3 | Sotorasib | KRAS | Other solid tumors |
| NCT04720976 | NCT04720976-4 | Jab-3312 | SHP2 | Other solid tumors |
| NCT04720976 | NCT04720976-4 | Osimertinib | EGFR | Other solid tumors |
| NCT04721977 | NCT04721977-0 | Tucatinib | HER2 | Breast cancer |
| NCT04721977 | NCT04721977-0 | Trastuzumab | HER2 | Breast cancer |
| NCT04721977 | NCT04721977-0 | Capecitabine | chemotherapy | Breast cancer |
| NCT04722172 | NCT04722172-0 | Acalabrutinib | BTK | Leukaemia |
| NCT04722172 | NCT04722172-0 | Obinutuzumab | CD20 | Leukaemia |
| NCT04722523 | NCT04722523-0 | Cemiplimab-Rwlc | PD-1 | Head and Neck Neoplasms |
| NCT04722523 | NCT04722523-0 | Platinum-Based Chemotherapy | chemotherapy | Head and Neck Neoplasms |
| NCT04722523 | NCT04722523-0 | Docetaxel | chemotherapy | Head and Neck Neoplasms |
| NCT04722523 | NCT04722523-0 | Cetuximab | EGFR | Head and Neck Neoplasms |
| NCT04724018 | NCT04724018-0 | Sacituzumab Govitecan | Trop-2/TOP1 | Bladder cancer |
| NCT04724018 | NCT04724018-0 | Enfortumab Vedotin | NECTIN4/Tubulin | Bladder cancer |
| NCT04728230 | NCT04728230-0 | Olaparib | PARP | lung cancers |
| NCT04728230 | NCT04728230-0 | Durvalumab | PD-L1 | lung cancers |
| NCT04728230 | NCT04728230-0 | Platinum-Based Chemotherapy | chemotherapy | lung cancers |
| NCT04728230 | NCT04728230-0 | Etoposide | chemotherapy | lung cancers |
| NCT04729387 | NCT04729387-0 | Alpelisib | PI3K | Ovary cancer |
| NCT04729387 | NCT04729387-0 | Olaparib | PARP | Ovary cancer |
| NCT04729725 | NCT04729725-0 | Sar439459 | TGFβ | Other solid tumors |
| NCT04729725 | NCT04729725-0 | Cemiplimab-Rwlc | PD-1 | Other solid tumors |
| NCT04734665 | NCT04734665-0 | Niraparib | PARP | Ovary cancer |
| NCT04734665 | NCT04734665-0 | Bevacizumab | VEGF | Ovary cancer |
| NCT04734730 | NCT04734730-0 | Talazoparib | PARP | Prostate cancer |
| NCT04734730 | NCT04734730-0 | Androgen Deprivation Therapy (Adt) | Endocrine Therapy | Prostate cancer |
| NCT04735068 | NCT04735068-0 | Binimetinib | MEK | lung cancers |
| NCT04735068 | NCT04735068-0 | Hydroxychloroquine | antimalarial agent | lung cancers |
| NCT04736199 | NCT04736199-0 | Darolutamide | Endocrine Therapy | Prostate cancer |
| NCT04736199 | NCT04736199-0 | Androgen Deprivation Therapy (Adt) | Endocrine Therapy | Prostate cancer |
| NCT04736706 | NCT04736706-1 | Pembrolizumab | PD-1 | Kidney cancer |
| NCT04736706 | NCT04736706-1 | Belzutifan | HIF-2 | Kidney cancer |
| NCT04736706 | NCT04736706-1 | Lenvatinib | VEGFR/PDGFR/FGFR | Kidney cancer |
| NCT04736706 | NCT04736706-2 | Pembrolizumab | PD-1 | Kidney cancer |
| NCT04736706 | NCT04736706-2 | Quavonlimab | CTLA-4 | Kidney cancer |
| NCT04736706 | NCT04736706-2 | Lenvatinib | VEGFR/PDGFR/FGFR | Kidney cancer |
| NCT04736706 | NCT04736706-3 | Pembrolizumab | PD-1 | Kidney cancer |
| NCT04736706 | NCT04736706-3 | Lenvatinib | VEGFR/PDGFR/FGFR | Kidney cancer |
| NCT04736914 | NCT04736914-0 | Zanubrutinib | BTK | Lymphomas |
| NCT04736914 | NCT04736914-0 | Rituximab | CD20 | Lymphomas |
| NCT04736914 | NCT04736914-0 | Chemotherapy | chemotherapy | Lymphomas |
| NCT04737109 | NCT04737109-0 | Androgen Deprivation Therapy (Adt) | Endocrine Therapy | Prostate cancer |
| NCT04737109 | NCT04737109-0 | Ipatasertib | AKT | Prostate cancer |
| NCT04737109 | NCT04737109-0 | Darolutamide | Endocrine Therapy | Prostate cancer |
| NCT04739800 | NCT04739800-1 | Durvalumab | PD-L1 | Other solid tumors |
| NCT04739800 | NCT04739800-1 | Cediranib | KDR | Other solid tumors |
| NCT04739800 | NCT04739800-2 | Durvalumab | PD-L1 | Other solid tumors |
| NCT04739800 | NCT04739800-2 | Cediranib | KDR | Other solid tumors |
| NCT04739800 | NCT04739800-2 | Olaparib | PARP | Other solid tumors |
| NCT04739813 | NCT04739813-0 | Obinutuzumab | CD20 | Lymphomas |
| NCT04739813 | NCT04739813-0 | Prednisone | Steroid Treatment | Lymphomas |
| NCT04739813 | NCT04739813-0 | Lenalidomide | CRBN | Lymphomas |
| NCT04739813 | NCT04739813-0 | Polatuzumab Vedotin | CD79B/Tubulin | Lymphomas |
| NCT04739813 | NCT04739813-0 | Ibrutinib | BTK | Lymphomas |
| NCT04739813 | NCT04739813-0 | Venetoclax | BCL2 | Lymphomas |
| NCT04740190 | NCT04740190-0 | Talazoparib | PARP | Brain and nervous system cancers |
| NCT04740190 | NCT04740190-0 | Carboplatin | chemotherapy | Brain and nervous system cancers |
| NCT04741997 | NCT04741997-0 | Encorafenib | RAF | Melanoma |
| NCT04741997 | NCT04741997-0 | Binimetinib | MEK | Melanoma |
| NCT04742075 | NCT04742075-0 | Olaparib | PARP | Ovary cancer |
| NCT04742075 | NCT04742075-0 | Durvalumab | PD-L1 | Ovary cancer |
| NCT04745689 | NCT04745689-0 | Azd2811 | AURKB | lung cancers |
| NCT04745689 | NCT04745689-0 | Durvalumab | PD-L1 | lung cancers |
| NCT04747912 | NCT04747912-0 | Inotuzumab Ozogamicin | CD22/DNA | Leukaemia |
| NCT04747912 | NCT04747912-0 | Dasatinib | BCR-ABL | Leukaemia |
| NCT04747912 | NCT04747912-0 | Dexamethasone | Steroid Treatment | Leukaemia |
| NCT04747912 | NCT04747912-0 | Methotrexate | chemotherapy | Leukaemia |
| NCT04750954 | NCT04750954-0 | Peposertib | DNA-PK | Brain and nervous system cancers |
| NCT04750954 | NCT04750954-0 | Lutetium Lu 177 Dotatate | SSTR | Brain and nervous system cancers |
| NCT04751877 | NCT04751877-1 | Isatuximab | CD38 | Multiple Myeloma |
| NCT04751877 | NCT04751877-1 | Lenalidomide | CRBN | Multiple Myeloma |
| NCT04751877 | NCT04751877-1 | Dexamethasone | Steroid Treatment | Multiple Myeloma |
| NCT04751877 | NCT04751877-1 | Bortezomib | PSMB | Multiple Myeloma |
| NCT04751877 | NCT04751877-2 | Isatuximab | CD38 | Multiple Myeloma |
| NCT04751877 | NCT04751877-2 | Lenalidomide | CRBN | Multiple Myeloma |
| NCT04751877 | NCT04751877-2 | Dexamethasone | Steroid Treatment | Multiple Myeloma |
| NCT04751929 | NCT04751929-0 | Abemaciclib | CDK4/CDK6 | Prostate cancer |
| NCT04751929 | NCT04751929-0 | Atezolizumab | PD-L1 | Prostate cancer |
| NCT04752332 | NCT04752332-0 | Abemaciclib | CDK4/CDK6 | Breast cancer |
| NCT04752332 | NCT04752332-0 | Adjuvant Endocrine Therapy | Endocrine Therapy | Breast cancer |
| NCT04753203 | NCT04753203-0 | Alpelisib | PI3K | Colon and rectum cancers |
| NCT04753203 | NCT04753203-0 | Capecitabine | chemotherapy | Colon and rectum cancers |
| NCT04756401 | NCT04756401-0 | Carfilzomib | PSMB | Multiple Myeloma |
| NCT04756401 | NCT04756401-0 | Daratumumab | CD38 | Multiple Myeloma |
| NCT04756401 | NCT04756401-0 | Dexamethasone | Steroid Treatment | Multiple Myeloma |
| NCT04756401 | NCT04756401-0 | Selinexor | XPO1 | Multiple Myeloma |
| NCT04759846 | NCT04759846-0 | Binimetinib | MEK | Melanoma |
| NCT04759846 | NCT04759846-0 | Encorafenib | RAF | Melanoma |
| NCT04760431 | NCT04760431-0 | Trastuzumab | HER2 | Breast cancer |
| NCT04760431 | NCT04760431-0 | Taxanes | chemotherapy | Breast cancer |
| NCT04760431 | NCT04760431-0 | Pertuzumab | HER2 | Breast cancer |
| NCT04762030 | NCT04762030-0 | Durvalumab | PD-L1 | lung cancers |
| NCT04762030 | NCT04762030-0 | Platinum-Based Chemotherapy | chemotherapy | lung cancers |
| NCT04762030 | NCT04762030-0 | Paclitaxel | chemotherapy | lung cancers |
| NCT04762030 | NCT04762030-0 | Anlotinib | VEGFR/PDGFR/FGFR | lung cancers |
| NCT04762160 | NCT04762160-0 | Tazemetostat | EZH2 | Lymphomas |
| NCT04762160 | NCT04762160-0 | Rituximab | CD20 | Lymphomas |
| NCT04762979 | NCT04762979-1 | Alpelisib | PI3K | Breast cancer |
| NCT04762979 | NCT04762979-1 | Fulvestrant | Endocrine Therapy | Breast cancer |
| NCT04762979 | NCT04762979-2 | Alpelisib | PI3K | Breast cancer |
| NCT04762979 | NCT04762979-2 | Aromatase Inhibitor | Endocrine Therapy | Breast cancer |
| NCT04763616 | NCT04763616-0 | Isatuximab | CD38 | Lymphomas |
| NCT04763616 | NCT04763616-0 | Cemiplimab-Rwlc | PD-1 | Lymphomas |
| NCT04764084 | NCT04764084-0 | Niraparib | PARP | Other solid tumors |
| NCT04764084 | NCT04764084-0 | Anlotinib | VEGFR/PDGFR/FGFR | Other solid tumors |
| NCT04764942 | NCT04764942-1 | Selinexor | XPO1 | Multiple Myeloma |
| NCT04764942 | NCT04764942-1 | Dexamethasone | Steroid Treatment | Multiple Myeloma |
| NCT04764942 | NCT04764942-1 | Carfilzomib | PSMB | Multiple Myeloma |
| NCT04764942 | NCT04764942-2 | Selinexor | XPO1 | Multiple Myeloma |
| NCT04764942 | NCT04764942-2 | Dexamethasone | Steroid Treatment | Multiple Myeloma |
| NCT04764942 | NCT04764942-2 | Pomalidomide | CRBN | Multiple Myeloma |
| NCT04765111 | NCT04765111-0 | Acalabrutinib | BTK | Lymphomas |
| NCT04765111 | NCT04765111-0 | Rituximab | CD20 | Lymphomas |
| NCT04765709 | NCT04765709-0 | Durvalumab | PD-L1 | lung cancers |
| NCT04765709 | NCT04765709-0 | Platinum-Based Chemotherapy | chemotherapy | lung cancers |
| NCT04768881 | NCT04768881-0 | Selinexor | XPO1 | Melanoma |
| NCT04768881 | NCT04768881-0 | Pembrolizumab | PD-1 | Melanoma |
| NCT04774380 | NCT04774380-0 | Durvalumab | PD-L1 | lung cancers |
| NCT04774380 | NCT04774380-0 | Platinum-Based Chemotherapy | chemotherapy | lung cancers |
| NCT04774380 | NCT04774380-0 | Etoposide | chemotherapy | lung cancers |
| NCT04774393 | NCT04774393-1 | Astx727(Decitabine/Cedazuridine) | chemotherapy | Leukaemia |
| NCT04774393 | NCT04774393-1 | Venetoclax | BCL2 | Leukaemia |
| NCT04774393 | NCT04774393-1 | Ivosidenib | IDH | Leukaemia |
| NCT04774393 | NCT04774393-2 | Astx727(Decitabine/Cedazuridine) | chemotherapy | Leukaemia |
| NCT04774393 | NCT04774393-2 | Venetoclax | BCL2 | Leukaemia |
| NCT04774393 | NCT04774393-2 | Enasidenib | IDH | Leukaemia |
| NCT04779151 | NCT04779151-0 | Niraparib | PARP | Other solid tumors |
| NCT04779151 | NCT04779151-0 | Dostarlimab-Gxly | PD-1 | Other solid tumors |
| NCT04781192 | NCT04781192-0 | Regorafenib | VEGFR/PDGFR/RET/RAF | Gallbladder and biliary tract cancer |
| NCT04781192 | NCT04781192-0 | Durvalumab | PD-L1 | Gallbladder and biliary tract cancer |
| NCT04782687 | NCT04782687-0 | Selinexor | XPO1 | Multiple Myeloma |
| NCT04782687 | NCT04782687-0 | Dexamethasone | Steroid Treatment | Multiple Myeloma |
| NCT04782687 | NCT04782687-0 | Daratumumab | CD38 | Multiple Myeloma |
| NCT04782687 | NCT04782687-0 | Lenalidomide | CRBN | Multiple Myeloma |
| NCT04783415 | NCT04783415-0 | Ublituximab | CD20 | Lymphomas |
| NCT04783415 | NCT04783415-0 | Acalabrutinib | BTK | Lymphomas |
| NCT04783415 | NCT04783415-0 | Umbralisib | PI3K | Lymphomas |
| NCT04784715 | NCT04784715-0 | Trastuzumab Deruxtecan | HER2/TOP1 | Breast cancer |
| NCT04784715 | NCT04784715-0 | Pertuzumab | HER2 | Breast cancer |
| NCT04786028 | NCT04786028-0 | Isatuximab | CD38 | Multiple Myeloma |
| NCT04786028 | NCT04786028-0 | Cyclophosphamide | chemotherapy | Multiple Myeloma |
| NCT04786028 | NCT04786028-0 | Bortezomib | PSMB | Multiple Myeloma |
| NCT04786028 | NCT04786028-0 | Dexamethasone | Steroid Treatment | Multiple Myeloma |
| NCT04786028 | NCT04786028-0 | Lenalidomide | CRBN | Multiple Myeloma |
| NCT04789096 | NCT04789096-0 | Tucatinib | HER2 | Breast cancer |
| NCT04789096 | NCT04789096-0 | Pembrolizumab | PD-1 | Breast cancer |
| NCT04789096 | NCT04789096-0 | Trastuzumab | HER2 | Breast cancer |
| NCT04790903 | NCT04790903-0 | Venetoclax | BCL2 | Lymphomas |
| NCT04790903 | NCT04790903-0 | Polatuzumab Vedotin | CD79B/Tubulin | Lymphomas |
| NCT04790903 | NCT04790903-0 | Rituximab | CD20 | Lymphomas |
| NCT04790903 | NCT04790903-0 | Cyclophosphamide | chemotherapy | Lymphomas |
| NCT04790903 | NCT04790903-0 | Doxorubicin | chemotherapy | Lymphomas |
| NCT04790903 | NCT04790903-0 | Prednisone | Steroid Treatment | Lymphomas |
| NCT04791384 | NCT04791384-0 | Abemaciclib | CDK4/CDK6 | Breast cancer |
| NCT04791384 | NCT04791384-0 | Elacestrant | Endocrine Therapy | Breast cancer |
| NCT04800822 | NCT04800822-1 | Pf-07284892 | SHP2 | Other solid tumors |
| NCT04800822 | NCT04800822-1 | Lorlatinib | ALK | Other solid tumors |
| NCT04800822 | NCT04800822-2 | Pf-07284892 | SHP2 | Other solid tumors |
| NCT04800822 | NCT04800822-2 | Encorafenib | RAF | Other solid tumors |
| NCT04800822 | NCT04800822-2 | Cetuximab | EGFR | Other solid tumors |
| NCT04800822 | NCT04800822-3 | Pf-07284892 | SHP2 | Other solid tumors |
| NCT04800822 | NCT04800822-3 | Binimetinib | MEK | Other solid tumors |
| NCT04800978 | NCT04800978-0 | Durvalumab | PD-L1 | Cervix uteri cancer |
| NCT04800978 | NCT04800978-0 | Vaccine | vaccine | Cervix uteri cancer |
| NCT04802174 | NCT04802174-0 | Berzosertib | ATR | Other solid tumors |
| NCT04802174 | NCT04802174-0 | Lurbinectedin | chemotherapy | Other solid tumors |
| NCT04802356 | NCT04802356-0 | Belantamab Mafodotin | BCMA/Tubulin | Multiple Myeloma |
| NCT04802356 | NCT04802356-0 | Bortezomib | PSMB | Multiple Myeloma |
| NCT04802356 | NCT04802356-0 | Lenalidomide | CRBN | Multiple Myeloma |
| NCT04802356 | NCT04802356-0 | Dexamethasone | Steroid Treatment | Multiple Myeloma |
| NCT04802759 | NCT04802759-0 | Giredestrant | Endocrine Therapy | Breast cancer |
| NCT04802759 | NCT04802759-0 | Abemaciclib | CDK4/CDK6 | Breast cancer |
| NCT04803201 | NCT04803201-0 | Cyclophosphamide | chemotherapy | Lymphomas |
| NCT04803201 | NCT04803201-0 | Doxorubicin | chemotherapy | Lymphomas |
| NCT04803201 | NCT04803201-0 | Vincristine | chemotherapy | Lymphomas |
| NCT04803201 | NCT04803201-0 | Etoposide | chemotherapy | Lymphomas |
| NCT04803201 | NCT04803201-0 | Prednisone | Steroid Treatment | Lymphomas |
| NCT04803201 | NCT04803201-0 | Duvelisib | PI3K | Lymphomas |
| NCT04808037 | NCT04808037-0 | Belantamab Mafodotin | BCMA/Tubulin | Multiple Myeloma |
| NCT04808037 | NCT04808037-0 | Lenalidomide | CRBN | Multiple Myeloma |
| NCT04808037 | NCT04808037-0 | Dexamethasone | Steroid Treatment | Multiple Myeloma |
| NCT04809467 | NCT04809467-0 | Tafasitamab | CD19 | Leukaemia |
| NCT04809467 | NCT04809467-0 | Parsaclisib | PI3K | Leukaemia |
| NCT04812366 | NCT04812366-1 | Lhrha | Endocrine Therapy | Prostate cancer |
| NCT04812366 | NCT04812366-1 | Abiraterone | CYP17A1 | Prostate cancer |
| NCT04812366 | NCT04812366-1 | Prednisone | Steroid Treatment | Prostate cancer |
| NCT04812366 | NCT04812366-1 | Niraparib | PARP | Prostate cancer |
| NCT04812366 | NCT04812366-2 | Lhrha | Endocrine Therapy | Prostate cancer |
| NCT04812366 | NCT04812366-2 | Apalutamide | Endocrine Therapy | Prostate cancer |
| NCT04812366 | NCT04812366-2 | Atezolizumab | PD-L1 | Prostate cancer |
| NCT04812366 | NCT04812366-3 | Lhrha | Endocrine Therapy | Prostate cancer |
| NCT04812366 | NCT04812366-3 | Apalutamide | Endocrine Therapy | Prostate cancer |
| NCT04812366 | NCT04812366-4 | Lhrha | Endocrine Therapy | Prostate cancer |
| NCT04812366 | NCT04812366-4 | Apalutamide | Endocrine Therapy | Prostate cancer |
| NCT04812366 | NCT04812366-4 | Abiraterone | CYP17A1 | Prostate cancer |
| NCT04812366 | NCT04812366-4 | Prednisone | Steroid Treatment | Prostate cancer |
| NCT04812366 | NCT04812366-5 | Lhrha | Endocrine Therapy | Prostate cancer |
| NCT04812366 | NCT04812366-5 | Abiraterone | CYP17A1 | Prostate cancer |
| NCT04812366 | NCT04812366-5 | Prednisone | Steroid Treatment | Prostate cancer |
| NCT04812366 | NCT04812366-6 | Lhrha | Endocrine Therapy | Prostate cancer |
| NCT04812366 | NCT04812366-6 | Abiraterone | CYP17A1 | Prostate cancer |
| NCT04812366 | NCT04812366-6 | Prednisone | Steroid Treatment | Prostate cancer |
| NCT04812366 | NCT04812366-6 | Docetaxel | chemotherapy | Prostate cancer |
| NCT04816214 | NCT04816214-0 | Capmatinib | MET | lung cancers |
| NCT04816214 | NCT04816214-0 | Osimertinib | EGFR | lung cancers |
| NCT04817007 | NCT04817007-1 | Bms-986158 | BET | Other hematologic Neoplasms |
| NCT04817007 | NCT04817007-1 | Ruxolitinib | JAK | Other hematologic Neoplasms |
| NCT04817007 | NCT04817007-2 | Bms-986158 | BET | Other hematologic Neoplasms |
| NCT04817007 | NCT04817007-2 | Fedratinib | JAK | Other hematologic Neoplasms |
| NCT04817826 | NCT04817826-0 | Tremelimumab | CTLA-4 | Stomach cancer |
| NCT04817826 | NCT04817826-0 | Durvalumab | PD-L1 | Stomach cancer |
| NCT04819243 | NCT04819243-0 | Atezolizumab | PD-L1 | Breast cancer |
| NCT04819243 | NCT04819243-0 | Talazoparib | PARP | Breast cancer |
| NCT04821622 | NCT04821622-0 | Talazoparib | PARP | Prostate cancer |
| NCT04821622 | NCT04821622-0 | Enzalutamide | Endocrine Therapy | Prostate cancer |
| NCT04822337 | NCT04822337-0 | Carfilzomib | PSMB | Multiple Myeloma |
| NCT04822337 | NCT04822337-0 | Lenalidomide | CRBN | Multiple Myeloma |
| NCT04822337 | NCT04822337-0 | Dexamethasone | Steroid Treatment | Multiple Myeloma |
| NCT04822337 | NCT04822337-0 | Belantamab Mafodotin | BCMA/Tubulin | Multiple Myeloma |
| NCT04824092 | NCT04824092-0 | Tafasitamab | CD19 | Lymphomas |
| NCT04824092 | NCT04824092-0 | Lenalidomide | CRBN | Lymphomas |
| NCT04824937 | NCT04824937-0 | Telaglenastat | GLS1 | Prostate cancer |
| NCT04824937 | NCT04824937-0 | Talazoparib | PARP | Prostate cancer |
| NCT04826341 | NCT04826341-0 | Sacituzumab Govitecan | Trop-2/TOP1 | lung cancers |
| NCT04826341 | NCT04826341-0 | Berzosertib | ATR | lung cancers |
| NCT04826393 | NCT04826393-0 | Asp8374 | TIGIT | Brain and nervous system cancers |
| NCT04826393 | NCT04826393-0 | Cemiplimab-Rwlc | PD-1 | Brain and nervous system cancers |
| NCT04833114 | NCT04833114-0 | Polatuzumab Vedotin | CD79B/Tubulin | Lymphomas |
| NCT04833114 | NCT04833114-0 | Rituximab | CD20 | Lymphomas |
| NCT04833114 | NCT04833114-0 | Ifosfamide | Chemotherapy | Lymphomas |
| NCT04833114 | NCT04833114-0 | Platinum-Based Chemotherapy | chemotherapy | Lymphomas |
| NCT04833114 | NCT04833114-0 | Etoposide | chemotherapy | Lymphomas |
| NCT04835129 | NCT04835129-0 | Isatuximab | CD38 | Multiple Myeloma |
| NCT04835129 | NCT04835129-0 | Pomalidomide | CRBN | Multiple Myeloma |
| NCT04835129 | NCT04835129-0 | Elotuzumab | SLAMF7 | Multiple Myeloma |
| NCT04835129 | NCT04835129-0 | Dexamethasone | Steroid Treatment | Multiple Myeloma |
| NCT04835870 | NCT04835870-0 | Zanubrutinib | BTK | Lymphomas |
| NCT04835870 | NCT04835870-0 | Rituximab | CD20 | Lymphomas |
| NCT04835870 | NCT04835870-0 | Cyclophosphamide | chemotherapy | Lymphomas |
| NCT04835870 | NCT04835870-0 | Doxorubicin | chemotherapy | Lymphomas |
| NCT04835870 | NCT04835870-0 | Vincristine | chemotherapy | Lymphomas |
| NCT04835870 | NCT04835870-0 | Prednisone | Steroid Treatment | Lymphomas |
| NCT04836832 | NCT04836832-0 | Acalabrutinib | BTK | Lymphomas |
| NCT04836832 | NCT04836832-0 | Duvelisib | PI3K | Lymphomas |
| NCT04837209 | NCT04837209-0 | Niraparib | PARP | Breast cancer |
| NCT04837209 | NCT04837209-0 | Dostarlimab-Gxly | PD-1 | Breast cancer |
| NCT04838041 | NCT04838041-0 | Asciminib | BCR-ABL | Leukaemia |
| NCT04838041 | NCT04838041-0 | Imatinib | BCR-ABL | Leukaemia |
| NCT04841148 | NCT04841148-0 | Palbociclib | CDK4/CDK6 | Breast cancer |
| NCT04841148 | NCT04841148-0 | Avelumab | PD-L1 | Breast cancer |
| NCT04842604 | NCT04842604-0 | Glasdegib | SMO | Leukaemia |
| NCT04842604 | NCT04842604-0 | Azacitidine | chemotherapy | Leukaemia |
| NCT04846478 | NCT04846478-0 | Talazoparib | PARP | Prostate cancer |
| NCT04846478 | NCT04846478-0 | Tazemetostat | EZH2 | Prostate cancer |
| NCT04848064 | NCT04848064-0 | Mogamulizumab | CCR4 | Other hematologic Neoplasms |
| NCT04848064 | NCT04848064-0 | Chemotherapy | chemotherapy | Other hematologic Neoplasms |
| NCT04848064 | NCT04848064-0 | Nk Cell | cell therapy | Other hematologic Neoplasms |
| NCT04849364 | NCT04849364-0 | Talazoparib | PARP | Breast cancer |
| NCT04849364 | NCT04849364-0 | Atezolizumab | PD-L1 | Breast cancer |
| NCT04849364 | NCT04849364-0 | Capecitabine | chemotherapy | Breast cancer |
| NCT04850495 | NCT04850495-0 | Zanubrutinib | BTK | Lymphomas |
| NCT04850495 | NCT04850495-0 | Rituximab | CD20 | Lymphomas |
| NCT04850495 | NCT04850495-0 | Chemotherapy | chemotherapy | Lymphomas |
| NCT04850599 | NCT04850599-0 | Isatuximab | CD38 | Multiple Myeloma |
| NCT04850599 | NCT04850599-0 | Carfilzomib | PSMB | Multiple Myeloma |
| NCT04850599 | NCT04850599-0 | Pomalidomide | CRBN | Multiple Myeloma |
| NCT04850755 | NCT04850755-0 | Selinexor | XPO1 | Other solid tumors |
| NCT04850755 | NCT04850755-0 | Nivolumab | PD-1 | Other solid tumors |
| NCT04850755 | NCT04850755-0 | Ipilimumab | CTLA-4 | Other solid tumors |
| NCT04854434 | NCT04854434-0 | Selinexor | XPO1 | Colon and rectum cancers |
| NCT04854434 | NCT04854434-0 | Pembrolizumab | PD-1 | Colon and rectum cancers |
| NCT04855695 | NCT04855695-0 | Acalabrutinib | BTK | Lymphomas |
| NCT04855695 | NCT04855695-0 | Venetoclax | BCL2 | Lymphomas |
| NCT04855695 | NCT04855695-0 | Obinutuzumab | CD20 | Lymphomas |
| NCT04856189 | NCT04856189-0 | Selinexor | XPO1 | Bladder cancer |
| NCT04856189 | NCT04856189-0 | Pembrolizumab | PD-1 | Bladder cancer |
| NCT04862143 | NCT04862143-0 | Alpelisib | PI3K | Breast cancer |
| NCT04862143 | NCT04862143-0 | Fulvestrant | Endocrine Therapy | Breast cancer |
| NCT04862650 | NCT04862650-0 | Cemiplimab-Rwlc | PD-1 | Head and Neck Neoplasms |
| NCT04862650 | NCT04862650-0 | Paclitaxel | chemotherapy | Head and Neck Neoplasms |
| NCT04862650 | NCT04862650-0 | Platinum-Based Chemotherapy | chemotherapy | Head and Neck Neoplasms |
| NCT04863885 | NCT04863885-0 | Ipilimumab | CTLA-4 | Bladder cancer |
| NCT04863885 | NCT04863885-0 | Nivolumab | PD-1 | Bladder cancer |
| NCT04863885 | NCT04863885-0 | Sacituzumab Govitecan | Trop-2/TOP1 | Bladder cancer |
| NCT04866017 | NCT04866017-0 | Ociperlimab | TIGIT | lung cancers |
| NCT04866017 | NCT04866017-0 | Tislelizumab | PD-1 | lung cancers |
| NCT04866017 | NCT04866017-0 | Chemotherapy | chemotherapy | lung cancers |
| NCT04870034 | NCT04870034-0 | Binimetinib | MEK | Other solid tumors |
| NCT04870034 | NCT04870034-0 | Palbociclib | CDK4/CDK6 | Other solid tumors |
| NCT04870112 | NCT04870112-0 | Durvalumab | PD-L1 | lung cancers |
| NCT04870112 | NCT04870112-0 | Cisplatin | chemotherapy | lung cancers |
| NCT04870112 | NCT04870112-0 | Etoposide | chemotherapy | lung cancers |
| NCT04871529 | NCT04871529-0 | Avelumab | PD-L1 | Bladder cancer |
| NCT04871529 | NCT04871529-0 | Gemcitabine | chemotherapy | Bladder cancer |
| NCT04871529 | NCT04871529-0 | Platinum-Based Chemotherapy | chemotherapy | Bladder cancer |
| NCT04876248 | NCT04876248-0 | Belantamab Mafodotin | BCMA/Tubulin | Multiple Myeloma |
| NCT04876248 | NCT04876248-0 | Lenalidomide | CRBN | Multiple Myeloma |
| NCT04877275 | NCT04877275-1 | Selinexor | XPO1 | Multiple Myeloma |
| NCT04877275 | NCT04877275-1 | Doxorubicin | chemotherapy | Multiple Myeloma |
| NCT04877275 | NCT04877275-1 | Dexamethasone | Steroid Treatment | Multiple Myeloma |
| NCT04877275 | NCT04877275-2 | Selinexor | XPO1 | Multiple Myeloma |
| NCT04877275 | NCT04877275-2 | Cyclophosphamide | chemotherapy | Multiple Myeloma |
| NCT04877275 | NCT04877275-2 | Dexamethasone | Steroid Treatment | Multiple Myeloma |
| NCT04878029 | NCT04878029-0 | Cabozantinib | VEGFR/MET/RET | Gallbladder and biliary tract cancer |
| NCT04878029 | NCT04878029-0 | Enfortumab Vedotin | NECTIN4/Tubulin | Gallbladder and biliary tract cancer |
| NCT04882163 | NCT04882163-1 | Cc-220 | CRBN | Lymphomas |
| NCT04882163 | NCT04882163-1 | Polatuzumab Vedotin | CD79B/Tubulin | Lymphomas |
| NCT04882163 | NCT04882163-1 | Rituximab | CD20 | Lymphomas |
| NCT04882163 | NCT04882163-2 | Cc-220 | CRBN | Lymphomas |
| NCT04882163 | NCT04882163-2 | Tafasitamab | CD19 | Lymphomas |
| NCT04883242 | NCT04883242-0 | Isatuximab | CD38 | Multiple Myeloma |
| NCT04883242 | NCT04883242-0 | Carfilzomib | PSMB | Multiple Myeloma |
| NCT04883242 | NCT04883242-0 | Pomalidomide | CRBN | Multiple Myeloma |
| NCT04883242 | NCT04883242-0 | Dexamethasone | Steroid Treatment | Multiple Myeloma |
| NCT04883437 | NCT04883437-0 | Acalabrutinib | BTK | Lymphomas |
| NCT04883437 | NCT04883437-0 | Obinutuzumab | CD20 | Lymphomas |
| NCT04885413 | NCT04885413-0 | Niraparib | PARP | Corpus uteri cancer |
| NCT04885413 | NCT04885413-0 | Pd-1 | PD-1 | Corpus uteri cancer |
| NCT04886531 | NCT04886531-0 | Neratinib | EGFR | Breast cancer |
| NCT04886531 | NCT04886531-0 | Endocrine Therapy | Endocrine Therapy | Breast cancer |
| NCT04886531 | NCT04886531-0 | Trastuzumab | HER2 | Breast cancer |
| NCT04887831 | NCT04887831-0 | Trilaciclib | CDK4/CDK6 | Bladder cancer |
| NCT04887831 | NCT04887831-0 | Chemotherapy | chemotherapy | Bladder cancer |
| NCT04890236 | NCT04890236-0 | Duvelisib | PI3K | Lymphomas |
| NCT04890236 | NCT04890236-0 | Cart-19 | cell therapy | Lymphomas |
| NCT04891744 | NCT04891744-0 | Selinexor | XPO1 | Multiple Myeloma |
| NCT04891744 | NCT04891744-0 | Thalidomide | CRBN | Multiple Myeloma |
| NCT04891744 | NCT04891744-0 | Dexamethasone | Steroid Treatment | Multiple Myeloma |
| NCT04891809 | NCT04891809-0 | Isatuximab | CD38 | Multiple Myeloma |
| NCT04891809 | NCT04891809-0 | Lenalidomide | CRBN | Multiple Myeloma |
| NCT04891809 | NCT04891809-0 | Dexamethasone | Steroid Treatment | Multiple Myeloma |
| NCT04892264 | NCT04892264-0 | Belantamab Mafodotin | BCMA/Tubulin | Multiple Myeloma |
| NCT04892264 | NCT04892264-0 | Lenalidomide | CRBN | Multiple Myeloma |
| NCT04892264 | NCT04892264-0 | Daratumumab | CD38 | Multiple Myeloma |
| NCT04892953 | NCT04892953-0 | Durvalumab | PD-L1 | lung cancers |
| NCT04892953 | NCT04892953-0 | Chemotherapy | chemotherapy | lung cancers |
| NCT04893252 | NCT04893252-0 | Vactosertib | ALK | Stomach cancer |
| NCT04893252 | NCT04893252-0 | Durvalumab | PD-L1 | Stomach cancer |
| NCT04895046 | NCT04895046-0 | Niraparib | PARP | Gallbladder and biliary tract cancer |
| NCT04895046 | NCT04895046-0 | Dostarlimab-Gxly | PD-1 | Gallbladder and biliary tract cancer |
| NCT04895579 | NCT04895579-0 | Copanlisib | PI3K | lung cancers |
| NCT04895579 | NCT04895579-0 | Durvalumab | PD-L1 | lung cancers |
| NCT04896320 | NCT04896320-1 | Gemcitabine | chemotherapy | Breast cancer |
| NCT04896320 | NCT04896320-1 | Tucatinib | HER2 | Breast cancer |
| NCT04896320 | NCT04896320-1 | Trastuzumab | HER2 | Breast cancer |
| NCT04896320 | NCT04896320-2 | Vinorelbine | chemotherapy | Breast cancer |
| NCT04896320 | NCT04896320-2 | Tucatinib | HER2 | Breast cancer |
| NCT04896320 | NCT04896320-2 | Trastuzumab | HER2 | Breast cancer |
| NCT04896658 | NCT04896658-0 | Belantamab Mafodotin | BCMA/Tubulin | Multiple Myeloma |
| NCT04896658 | NCT04896658-0 | Cyclophosphamide | chemotherapy | Multiple Myeloma |
| NCT04896658 | NCT04896658-0 | Dexamethasone | Steroid Treatment | Multiple Myeloma |
| NCT04897386 | NCT04897386-0 | Durvalumab | PD-L1 | lung cancers |
| NCT04897386 | NCT04897386-0 | Chemotherapy | chemotherapy | lung cancers |
| NCT04898894 | NCT04898894-0 | Selinexor | XPO1 | Leukaemia |
| NCT04898894 | NCT04898894-0 | Venetoclax | BCL2 | Leukaemia |
| NCT04898894 | NCT04898894-0 | Chemotherapy | chemotherapy | Leukaemia |
| NCT04899349 | NCT04899349-1 | Alpelisib | PI3K | Breast cancer |
| NCT04899349 | NCT04899349-1 | Fulvestrant | Endocrine Therapy | Breast cancer |
| NCT04899349 | NCT04899349-1 | Dapagliflozin | Antihyperglycemic | Breast cancer |
| NCT04899349 | NCT04899349-1 | Metformin | Antihyperglycemic | Breast cancer |
| NCT04899349 | NCT04899349-2 | Alpelisib | PI3K | Breast cancer |
| NCT04899349 | NCT04899349-2 | Fulvestrant | Endocrine Therapy | Breast cancer |
| NCT04899349 | NCT04899349-2 | Metformin | Antihyperglycemic | Breast cancer |
| NCT04899453 | NCT04899453-0 | Methotrexate | chemotherapy | Lymphomas |
| NCT04899453 | NCT04899453-0 | Zanubrutinib | BTK | Lymphomas |
| NCT04899453 | NCT04899453-0 | Rituximab | CD20 | Lymphomas |
| NCT04899570 | NCT04899570-0 | Zanubrutinib | BTK | Lymphomas |
| NCT04899570 | NCT04899570-0 | Rituximab | CD20 | Lymphomas |
| NCT04899570 | NCT04899570-0 | Chemotherapy | chemotherapy | Lymphomas |
| NCT04901299 | NCT04901299-0 | Fulvestrant | Endocrine Therapy | Breast cancer |
| NCT04901299 | NCT04901299-0 | Neratinib | EGFR | Breast cancer |
| NCT04901702 | NCT04901702-0 | Talazoparib | PARP | Other solid tumors |
| NCT04901702 | NCT04901702-0 | Irinotecan | chemotherapy | Other solid tumors |
| NCT04905316 | NCT04905316-0 | Canakinumab | IL1β | lung cancers |
| NCT04905316 | NCT04905316-0 | Chemotherapy | chemotherapy | lung cancers |
| NCT04905316 | NCT04905316-0 | Durvalumab | PD-L1 | lung cancers |
| NCT04909515 | NCT04909515-0 | Naxitamab | GD-2 | Brain and nervous system cancers |
| NCT04909515 | NCT04909515-0 | Gm-Csf | CSF2 | Brain and nervous system cancers |
| NCT04909515 | NCT04909515-0 | Isotretinoin | chemotherapy | Brain and nervous system cancers |
| NCT04912427 | NCT04912427-0 | Isatuximab | CD38 | Multiple Myeloma |
| NCT04912427 | NCT04912427-0 | Bortezomib | PSMB | Multiple Myeloma |
| NCT04912427 | NCT04912427-0 | Dexamethasone | Steroid Treatment | Multiple Myeloma |
| NCT04913103 | NCT04913103-0 | Polatuzumab Vedotin | CD79B/Tubulin | Lymphomas |
| NCT04913103 | NCT04913103-0 | Bendamustine | chemotherapy | Lymphomas |
| NCT04913103 | NCT04913103-0 | Rituximab | CD20 | Lymphomas |
| NCT04913220 | NCT04913220-0 | Sar444245 | IL2 | Melanoma |
| NCT04913220 | NCT04913220-0 | Cemiplimab-Rwlc | PD-1 | Melanoma |
| NCT04913285 | NCT04913285-0 | Kin-2787 | RAF | lung cancers |
| NCT04913285 | NCT04913285-0 | Binimetinib | MEK | lung cancers |
| NCT04914741 | NCT04914741-0 | Glofitamab | CD20/CD3 | Lymphomas |
| NCT04914741 | NCT04914741-0 | Polatuzumab Vedotin | CD79B/Tubulin | Lymphomas |
| NCT04914741 | NCT04914741-0 | Rituximab | CD20 | Lymphomas |
| NCT04914741 | NCT04914741-0 | Chemotherapy | chemotherapy | Lymphomas |
| NCT04916002 | NCT04916002-0 | Cmp-001 | TLR | Others |
| NCT04916002 | NCT04916002-0 | Cemiplimab-Rwlc | PD-1 | Others |
| NCT04916613 | NCT04916613-0 | Androgen Deprivation Therapy (Adt) | Endocrine Therapy | Prostate cancer |
| NCT04916613 | NCT04916613-0 | Darolutamide | Endocrine Therapy | Prostate cancer |
| NCT04918186 | NCT04918186-1 | Durvalumab | PD-L1 | Ovary cancer |
| NCT04918186 | NCT04918186-1 | Batiraxcept | AXL | Ovary cancer |
| NCT04918186 | NCT04918186-2 | Durvalumab | PD-L1 | Ovary cancer |
| NCT04918186 | NCT04918186-2 | Ba3021 | ROR | Ovary cancer |
| NCT04918628 | NCT04918628-0 | Sintilimab | PD-1 | Cervix uteri cancer |
| NCT04918628 | NCT04918628-0 | Chemotherapy | chemotherapy | Cervix uteri cancer |
| NCT04920708 | NCT04920708-0 | Palbociclib | CDK4/CDK6 | Breast cancer |
| NCT04920708 | NCT04920708-0 | Fulvestrant | Endocrine Therapy | Breast cancer |
| NCT04920708 | NCT04920708-0 | Ipatasertib | AKT | Breast cancer |
| NCT04921904 | NCT04921904-0 | Abemaciclib | CDK4/CDK6 | Stomach cancer |
| NCT04921904 | NCT04921904-0 | Ramucirumab | KDR | Stomach cancer |
| NCT04923542 | NCT04923542-0 | Abemaciclib | CDK4/CDK6 | Breast cancer |
| NCT04923542 | NCT04923542-0 | Endocrine Therapy | Endocrine Therapy | Breast cancer |
| NCT04925193 | NCT04925193-1 | Selinexor | XPO1 | Multiple Myeloma |
| NCT04925193 | NCT04925193-1 | Pomalidomide | CRBN | Multiple Myeloma |
| NCT04925193 | NCT04925193-1 | Dexamethasone | Steroid Treatment | Multiple Myeloma |
| NCT04925193 | NCT04925193-2 | Selinexor | XPO1 | Multiple Myeloma |
| NCT04925193 | NCT04925193-2 | Daratumumab | CD38 | Multiple Myeloma |
| NCT04925193 | NCT04925193-2 | Dexamethasone | Steroid Treatment | Multiple Myeloma |
| NCT04925193 | NCT04925193-3 | Selinexor | XPO1 | Multiple Myeloma |
| NCT04925193 | NCT04925193-3 | Carfilzomib | PSMB | Multiple Myeloma |
| NCT04925193 | NCT04925193-3 | Dexamethasone | Steroid Treatment | Multiple Myeloma |
| NCT04925648 | NCT04925648-0 | Dasatinib | BCR-ABL | Prostate cancer |
| NCT04925648 | NCT04925648-0 | Darolutamide | Endocrine Therapy | Prostate cancer |
| NCT04926181 | NCT04926181-0 | Apalutamide | Endocrine Therapy | Prostate cancer |
| NCT04926181 | NCT04926181-0 | Cetrelimab | PD-1 | Prostate cancer |
| NCT04926324 | NCT04926324-0 | Niraparib | PARP | Colon and rectum cancers |
| NCT04926324 | NCT04926324-0 | Dostarlimab-Gxly | PD-1 | Colon and rectum cancers |
| NCT04927884 | NCT04927884-0 | Sacituzumab Govitecan | Trop-2/TOP1 | Breast cancer |
| NCT04927884 | NCT04927884-0 | Cyclophosphamide | chemotherapy | Breast cancer |
| NCT04927884 | NCT04927884-0 | N-803 | IL15 | Breast cancer |
| NCT04927884 | NCT04927884-0 | Pd-L1 T-Hank | cell therapy | Breast cancer |
| NCT04938297 | NCT04938297-0 | Rituximab | CD20 | Lymphomas |
| NCT04938297 | NCT04938297-0 | Zanubrutinib | BTK | Lymphomas |
| NCT04938297 | NCT04938297-0 | Lenalidomide | CRBN | Lymphomas |
| NCT04939142 | NCT04939142-0 | Selinexor | XPO1 | Multiple Myeloma |
| NCT04939142 | NCT04939142-0 | Bortezomib | PSMB | Multiple Myeloma |
| NCT04939142 | NCT04939142-0 | Dexamethasone | Steroid Treatment | Multiple Myeloma |
| NCT04939844 | NCT04939844-0 | Isatuximab | CD38 | Multiple Myeloma |
| NCT04939844 | NCT04939844-0 | Bortezomib | PSMB | Multiple Myeloma |
| NCT04939844 | NCT04939844-0 | Lenalidomide | CRBN | Multiple Myeloma |
| NCT04939844 | NCT04939844-0 | Dexamethasone | Steroid Treatment | Multiple Myeloma |
| NCT04940286 | NCT04940286-0 | Gemcitabine | chemotherapy | Pancreas cancer |
| NCT04940286 | NCT04940286-0 | Paclitaxel | chemotherapy | Pancreas cancer |
| NCT04940286 | NCT04940286-0 | Durvalumab | PD-L1 | Pancreas cancer |
| NCT04940286 | NCT04940286-0 | Oleclumab | CD73 | Pancreas cancer |
| NCT04940637 | NCT04940637-0 | Niraparib | PARP | Other solid tumors |
| NCT04940637 | NCT04940637-0 | Dostarlimab-Gxly | PD-1 | Other solid tumors |
| NCT04945720 | NCT04945720-0 | Chemotherapy | chemotherapy | Liver cancer |
| NCT04945720 | NCT04945720-0 | Durvalumab | PD-L1 | Liver cancer |
| NCT04946370 | NCT04946370-0 | Pembrolizumab | PD-1 | Prostate cancer |
| NCT04946370 | NCT04946370-0 | Apalutamide | Endocrine Therapy | Prostate cancer |
| NCT04949191 | NCT04949191-0 | Pemigatinib | FGFR | Others |
| NCT04949191 | NCT04949191-0 | Retifanlimab | PD-1 | Others |
| NCT04951115 | NCT04951115-0 | Durvalumab | PD-L1 | lung cancers |
| NCT04951115 | NCT04951115-0 | Carboplatin | chemotherapy | lung cancers |
| NCT04951115 | NCT04951115-0 | Etoposide | chemotherapy | lung cancers |
| NCT04955938 | NCT04955938-1 | Ivosidenib | IDH | Others |
| NCT04955938 | NCT04955938-1 | Fedratinib | JAK | Others |
| NCT04955938 | NCT04955938-2 | Fedratinib | JAK | Others |
| NCT04955938 | NCT04955938-2 | Enasidenib | IDH | Others |
| NCT04959981 | NCT04959981-1 | Eras-007 | ERK | lung cancers |
| NCT04959981 | NCT04959981-1 | Osimertinib | EGFR | lung cancers |
| NCT04959981 | NCT04959981-2 | Eras-007 | ERK | lung cancers |
| NCT04959981 | NCT04959981-2 | Sotorasib | KRAS | lung cancers |
| NCT04959981 | NCT04959981-3 | Eras-601 | SHP2 | lung cancers |
| NCT04959981 | NCT04959981-3 | Sotorasib | KRAS | lung cancers |
| NCT04959981 | NCT04959981-4 | Eras-007 | ERK | lung cancers |
| NCT04959981 | NCT04959981-4 | Osimertinib | EGFR | lung cancers |
| NCT04959981 | NCT04959981-5 | Eras-007 | ERK | lung cancers |
| NCT04959981 | NCT04959981-5 | Sotorasib | KRAS | lung cancers |
| NCT04959981 | NCT04959981-6 | Eras-601 | SHP2 | lung cancers |
| NCT04959981 | NCT04959981-6 | Sotorasib | KRAS | lung cancers |
| NCT04960709 | NCT04960709-1 | Durvalumab | PD-L1 | Gallbladder and biliary tract cancer |
| NCT04960709 | NCT04960709-1 | Tremelimumab | CTLA-4 | Gallbladder and biliary tract cancer |
| NCT04960709 | NCT04960709-1 | Enfortumab Vedotin | NECTIN4/Tubulin | Gallbladder and biliary tract cancer |
| NCT04960709 | NCT04960709-2 | Durvalumab | PD-L1 | Gallbladder and biliary tract cancer |
| NCT04960709 | NCT04960709-2 | Enfortumab Vedotin | NECTIN4/Tubulin | Gallbladder and biliary tract cancer |
| NCT04961918 | NCT04961918-0 | Lenvatinib | VEGFR/PDGFR/FGFR | Liver cancer |
| NCT04961918 | NCT04961918-0 | Durvalumab | PD-L1 | Liver cancer |
| NCT04963153 | NCT04963153-0 | Erdafitinib | FGFR | Gallbladder and biliary tract cancer |
| NCT04963153 | NCT04963153-0 | Enfortumab Vedotin | NECTIN4/Tubulin | Gallbladder and biliary tract cancer |
| NCT04965090 | NCT04965090-0 | Amivantamab | EGFR/MET | lung cancers |
| NCT04965090 | NCT04965090-0 | Lazertinib | EGFR | lung cancers |
| NCT04965155 | NCT04965155-0 | Isatuximab | CD38 | Multiple Myeloma |
| NCT04965155 | NCT04965155-0 | Dexamethasone | Steroid Treatment | Multiple Myeloma |
| NCT04965818 | NCT04965818-0 | Futibatinib | FGFR | lung cancers |
| NCT04965818 | NCT04965818-0 | Binimetinib | MEK | lung cancers |
| NCT04972110 | NCT04972110-0 | Rp-3500 | ATR | Other solid tumors |
| NCT04972110 | NCT04972110-0 | Niraparib | PARP | Other solid tumors |
| NCT04975308 | NCT04975308-0 | Imlunestrant | Endocrine Therapy | Breast cancer |
| NCT04975308 | NCT04975308-0 | Abemaciclib | CDK4/CDK6 | Breast cancer |
| NCT04976634 | NCT04976634-0 | Pembrolizumab | PD-1 | Other solid tumors |
| NCT04976634 | NCT04976634-0 | Lenvatinib | VEGFR/PDGFR/FGFR | Other solid tumors |
| NCT04976634 | NCT04976634-0 | Belzutifan | HIF-2 | Other solid tumors |
| NCT04983745 | NCT04983745-0 | Niraparib | PARP | Other solid tumors |
| NCT04983745 | NCT04983745-0 | Dostarlimab-Gxly | PD-1 | Other solid tumors |
| NCT04985851 | NCT04985851-0 | Durvalumab | PD-L1 | lung cancers |
| NCT04985851 | NCT04985851-0 | Anlotinib | VEGFR/PDGFR/FGFR | lung cancers |
| NCT04987203 | NCT04987203-0 | Tivozanib | VEGFR/PDGFR | Kidney cancer |
| NCT04987203 | NCT04987203-0 | Nivolumab | PD-1 | Kidney cancer |
| NCT04988295 | NCT04988295-0 | Amivantamab | EGFR/MET | lung cancers |
| NCT04988295 | NCT04988295-0 | Lazertinib | EGFR | lung cancers |
| NCT04988295 | NCT04988295-0 | Chemotherapy | chemotherapy | lung cancers |
| NCT04989218 | NCT04989218-0 | Durvalumab | PD-L1 | Gallbladder and biliary tract cancer |
| NCT04989218 | NCT04989218-0 | Tremelimumab | CTLA-4 | Gallbladder and biliary tract cancer |
| NCT04989218 | NCT04989218-0 | Chemotherapy | chemotherapy | Gallbladder and biliary tract cancer |
| NCT04991480 | NCT04991480-1 | Art4215 | Pol θ | Breast cancer |
| NCT04991480 | NCT04991480-1 | Talazoparib | PARP | Breast cancer |
| NCT04991480 | NCT04991480-2 | Art4215 | Pol θ | Breast cancer |
| NCT04991480 | NCT04991480-2 | Niraparib | PARP | Breast cancer |
| NCT04997902 | NCT04997902-0 | Tipifarnib | FNTA/FNTB | Head and Neck Neoplasms |
| NCT04997902 | NCT04997902-0 | Alpelisib | PI3K | Head and Neck Neoplasms |
| NCT04999969 | NCT04999969-0 | Azd0171 | LIF | Other solid tumors |
| NCT04999969 | NCT04999969-0 | Durvalumab | PD-L1 | Other solid tumors |
| NCT04999969 | NCT04999969-0 | Chemotherapy | chemotherapy | Other solid tumors |
| NCT05000294 | NCT05000294-0 | Atezolizumab | PD-L1 | Other solid tumors |
| NCT05000294 | NCT05000294-0 | Tivozanib | VEGFR/PDGFR | Other solid tumors |
| NCT05000710 | NCT05000710-0 | Tremelimumab | CTLA-4 | lung cancers |
| NCT05000710 | NCT05000710-0 | Durvalumab | PD-L1 | lung cancers |
| NCT05004064 | NCT05004064-0 | Acalabrutinib | BTK | Lymphomas |
| NCT05004064 | NCT05004064-0 | Rituximab | CD20 | Lymphomas |
| NCT05004350 | NCT05004350-0 | Encorafenib | RAF | Colon and rectum cancers |
| NCT05004350 | NCT05004350-0 | Cetuximab | EGFR | Colon and rectum cancers |
| NCT05004974 | NCT05004974-0 | Sintilimab | PD-1 | lung cancers |
| NCT05004974 | NCT05004974-0 | Pemigatinib | FGFR | lung cancers |
| NCT05006794 | NCT05006794-1 | Gs-9716 | MCL1 | Other solid tumors |
| NCT05006794 | NCT05006794-1 | Sacituzumab Govitecan | Trop-2/TOP1 | Other solid tumors |
| NCT05006794 | NCT05006794-2 | Gs-9716 | MCL1 | Other solid tumors |
| NCT05006794 | NCT05006794-2 | Chemotherapy | chemotherapy | Other solid tumors |
| NCT05010005 | NCT05010005-0 | Ruxolitinib | JAK | Lymphomas |
| NCT05010005 | NCT05010005-0 | Duvelisib | PI3K | Lymphomas |
| NCT05010096 | NCT05010096-0 | Bay1895344 | ATR | Other solid tumors |
| NCT05010096 | NCT05010096-0 | Copanlisib | PI3K | Other solid tumors |
| NCT05010122 | NCT05010122-0 | Astx727(Decitabine/Cedazuridine) | chemotherapy | Leukaemia |
| NCT05010122 | NCT05010122-0 | Venetoclax | BCL2 | Leukaemia |
| NCT05010122 | NCT05010122-0 | Gilteritinib | FLT3 | Leukaemia |
| NCT05010772 | NCT05010772-1 | Astx727(Decitabine/Cedazuridine) | chemotherapy | Leukaemia |
| NCT05010772 | NCT05010772-1 | Venetoclax | BCL2 | Leukaemia |
| NCT05010772 | NCT05010772-2 | Astx727(Decitabine/Cedazuridine) | chemotherapy | Leukaemia |
| NCT05010772 | NCT05010772-2 | Gilteritinib | FLT3 | Leukaemia |
| NCT05010772 | NCT05010772-3 | Astx727(Decitabine/Cedazuridine) | chemotherapy | Leukaemia |
| NCT05010772 | NCT05010772-3 | Enasidenib | IDH | Leukaemia |
| NCT05010772 | NCT05010772-4 | Astx727(Decitabine/Cedazuridine) | chemotherapy | Leukaemia |
| NCT05010772 | NCT05010772-4 | Ivosidenib | IDH | Leukaemia |
| NCT05016947 | NCT05016947-0 | Venetoclax | BCL2 | Leukaemia |
| NCT05016947 | NCT05016947-0 | Inotuzumab Ozogamicin | CD22/DNA | Leukaemia |
| NCT05016947 | NCT05016947-0 | Dexamethasone | Steroid Treatment | Leukaemia |
| NCT05019534 | NCT05019534-0 | Vemurafenib | RAF | Colon and rectum cancers |
| NCT05019534 | NCT05019534-0 | Cetuximab | EGFR | Colon and rectum cancers |
| NCT05019534 | NCT05019534-0 | Camrelizumab | PD-1 | Colon and rectum cancers |
| NCT05024552 | NCT05024552-0 | Vyxeos(Daunorubicin/Cytarabine) | chemotherapy | Leukaemia |
| NCT05024552 | NCT05024552-0 | Gilteritinib | FLT3 | Leukaemia |
| NCT05025735 | NCT05025735-0 | Fulvestrant | Endocrine Therapy | Breast cancer |
| NCT05025735 | NCT05025735-0 | Alpelisib | PI3K | Breast cancer |
| NCT05025735 | NCT05025735-0 | Dapagliflozin | Antihyperglycemic | Breast cancer |
| NCT05026983 | NCT05026983-0 | Binimetinib | MEK | Melanoma |
| NCT05026983 | NCT05026983-0 | Encorafenib | RAF | Melanoma |
| NCT05027425 | NCT05027425-0 | Durvalumab | PD-L1 | Liver cancer |
| NCT05027425 | NCT05027425-0 | Tremelimumab | CTLA-4 | Liver cancer |
| NCT05030506 | NCT05030506-1 | Belzutifan | HIF-2 | Kidney cancer |
| NCT05030506 | NCT05030506-1 | Lenvatinib | VEGFR/PDGFR/FGFR | Kidney cancer |
| NCT05030506 | NCT05030506-2 | Belzutifan | HIF-2 | Kidney cancer |
| NCT05030506 | NCT05030506-2 | Lenvatinib | VEGFR/PDGFR/FGFR | Kidney cancer |
| NCT05030506 | NCT05030506-2 | Pembrolizumab | PD-1 | Kidney cancer |
| NCT05034133 | NCT05034133-0 | Durvalumab | PD-L1 | lung cancers |
| NCT05034133 | NCT05034133-0 | Chemotherapy | chemotherapy | lung cancers |
| NCT05035745 | NCT05035745-0 | Selinexor | XPO1 | Breast cancer |
| NCT05035745 | NCT05035745-0 | Talazoparib | PARP | Breast cancer |
| NCT05038735 | NCT05038735-0 | Alpelisib | PI3K | Breast cancer |
| NCT05038735 | NCT05038735-0 | Fulvestrant | Endocrine Therapy | Breast cancer |
| NCT05039177 | NCT05039177-0 | Eras-007 | ERK | Colon and rectum cancers |
| NCT05039177 | NCT05039177-0 | Encorafenib | RAF | Colon and rectum cancers |
| NCT05039177 | NCT05039177-0 | Cetuximab | EGFR | Colon and rectum cancers |
| NCT05043090 | NCT05043090-0 | Savolitinib | MET | Kidney cancer |
| NCT05043090 | NCT05043090-0 | Durvalumab | PD-L1 | Kidney cancer |
| NCT05053854 | NCT05053854-0 | Lutetium Lu 177 Dotatate | SSTR | Brain and nervous system cancers |
| NCT05053854 | NCT05053854-0 | Talazoparib | PARP | Brain and nervous system cancers |
| NCT05054725 | NCT05054725-0 | Rmc-4630 | SHP2 | lung cancers |
| NCT05054725 | NCT05054725-0 | Sotorasib | KRAS | lung cancers |
| NCT05057247 | NCT05057247-0 | Duvelisib | PI3K | Head and Neck Neoplasms |
| NCT05057247 | NCT05057247-0 | Docetaxel | chemotherapy | Head and Neck Neoplasms |
| NCT05059236 | NCT05059236-0 | Darolutamide | Endocrine Therapy | Prostate cancer |
| NCT05059236 | NCT05059236-0 | Androgen Deprivation Therapy (Adt) | Endocrine Therapy | Prostate cancer |
| NCT05059522 | NCT05059522-1 | Avelumab | PD-L1 | Other solid tumors |
| NCT05059522 | NCT05059522-1 | Cmp-001 | TLR | Other solid tumors |
| NCT05059522 | NCT05059522-1 | Utomilumab | 4-1BB | Other solid tumors |
| NCT05059522 | NCT05059522-1 | Pf-04518600 | OX40 | Other solid tumors |
| NCT05059522 | NCT05059522-2 | Avelumab | PD-L1 | Other solid tumors |
| NCT05059522 | NCT05059522-2 | Lorlatinib | ALK | Other solid tumors |
| NCT05059522 | NCT05059522-3 | Avelumab | PD-L1 | Other solid tumors |
| NCT05059522 | NCT05059522-3 | Pemetrexed | chemotherapy | Other solid tumors |
| NCT05059522 | NCT05059522-4 | Avelumab | PD-L1 | Other solid tumors |
| NCT05059522 | NCT05059522-4 | Talazoparib | PARP | Other solid tumors |
| NCT05059522 | NCT05059522-5 | Avelumab | PD-L1 | Other solid tumors |
| NCT05059522 | NCT05059522-5 | Axitinib | VEGFR/PDGFR | Other solid tumors |
| NCT05060627 | NCT05060627-0 | Belantamab Mafodotin | BCMA/Tubulin | Multiple Myeloma |
| NCT05060627 | NCT05060627-0 | Carfilzomib | PSMB | Multiple Myeloma |
| NCT05060627 | NCT05060627-0 | Dexamethasone | Steroid Treatment | Multiple Myeloma |
| NCT05061550 | NCT05061550-1 | Durvalumab | PD-L1 | lung cancers |
| NCT05061550 | NCT05061550-1 | Oleclumab | CD73 | lung cancers |
| NCT05061550 | NCT05061550-1 | Chemotherapy | chemotherapy | lung cancers |
| NCT05061550 | NCT05061550-2 | Durvalumab | PD-L1 | lung cancers |
| NCT05061550 | NCT05061550-2 | Monalizumab | NKG2A | lung cancers |
| NCT05061550 | NCT05061550-2 | Chemotherapy | chemotherapy | lung cancers |
| NCT05063786 | NCT05063786-1 | Trastuzumab | HER2 | Breast cancer |
| NCT05063786 | NCT05063786-1 | Alpelisib | PI3K | Breast cancer |
| NCT05063786 | NCT05063786-2 | Trastuzumab | HER2 | Breast cancer |
| NCT05063786 | NCT05063786-2 | Alpelisib | PI3K | Breast cancer |
| NCT05063786 | NCT05063786-2 | Fulvestrant | Endocrine Therapy | Breast cancer |
| NCT05063786 | NCT05063786-3 | Trastuzumab | HER2 | Breast cancer |
| NCT05063786 | NCT05063786-3 | Vinorelbine | chemotherapy | Breast cancer |
| NCT05063786 | NCT05063786-3 | Capecitabine | chemotherapy | Breast cancer |
| NCT05063786 | NCT05063786-3 | Eribulin | chemotherapy | Breast cancer |
| NCT05064085 | NCT05064085-0 | Capecitabine | chemotherapy | Breast cancer |
| NCT05064085 | NCT05064085-0 | Cemiplimab-Rwlc | PD-1 | Breast cancer |
| NCT05065021 | NCT05065021-0 | Niraparib | PARP | Other solid tumors |
| NCT05065021 | NCT05065021-0 | Bevacizumab | VEGF | Other solid tumors |
| NCT05065554 | NCT05065554-0 | Acalabrutinib | BTK | Others |
| NCT05065554 | NCT05065554-0 | Rituximab | CD20 | Others |
| NCT05065866 | NCT05065866-0 | Duvelisib | PI3K | Lymphomas |
| NCT05065866 | NCT05065866-0 | Azacitidine | chemotherapy | Lymphomas |
| NCT05081180 | NCT05081180-0 | Avelumab | PD-L1 | Brain and nervous system cancers |
| NCT05081180 | NCT05081180-0 | Lenvatinib | VEGFR/PDGFR/FGFR | Brain and nervous system cancers |
| NCT05091567 | NCT05091567-0 | Atezolizumab | PD-L1 | lung cancers |
| NCT05091567 | NCT05091567-0 | Lurbinectedin | chemotherapy | lung cancers |
| NCT05092412 | NCT05092412-0 | Durvalumab | PD-L1 | lung cancers |
| NCT05092412 | NCT05092412-0 | Etoposide | chemotherapy | lung cancers |
| NCT05092412 | NCT05092412-0 | Cisplatin | chemotherapy | lung cancers |
| NCT05093608 | NCT05093608-0 | Selinexor | XPO1 | Liver cancer |
| NCT05093608 | NCT05093608-0 | Bevacizumab | VEGF | Liver cancer |
| NCT05093608 | NCT05093608-0 | Atezolizumab | PD-L1 | Liver cancer |
| NCT05095207 | NCT05095207-0 | Abemaciclib | CDK4/CDK6 | Breast cancer |
| NCT05095207 | NCT05095207-0 | Bicalutamide | Endocrine Therapy | Breast cancer |
| NCT05097599 | NCT05097599-0 | Encorafenib | RAF | Other solid tumors |
| NCT05097599 | NCT05097599-0 | Binimetinib | MEK | Other solid tumors |
| NCT05113251 | NCT05113251-0 | Trastuzumab Deruxtecan | HER2/TOP1 | Breast cancer |
| NCT05113251 | NCT05113251-0 | Paclitaxel | chemotherapy | Breast cancer |
| NCT05113251 | NCT05113251-0 | Trastuzumab | HER2 | Breast cancer |
| NCT05113251 | NCT05113251-0 | Pertuzumab | HER2 | Breast cancer |
| NCT05113966 | NCT05113966-0 | Trilaciclib | CDK4/CDK6 | Breast cancer |
| NCT05113966 | NCT05113966-0 | Sacituzumab Govitecan | Trop-2/TOP1 | Breast cancer |
| NCT05120622 | NCT05120622-0 | Tremelimumab | CTLA-4 | Gallbladder and biliary tract cancer |
| NCT05120622 | NCT05120622-0 | Durvalumab | PD-L1 | Gallbladder and biliary tract cancer |
| NCT05125016 | NCT05125016-0 | Regn4336 | PSMA/CD3 | Prostate cancer |
| NCT05125016 | NCT05125016-0 | Cemiplimab-Rwlc | PD-1 | Prostate cancer |
| NCT05130515 | NCT05130515-0 | Anlotinib | VEGFR/PDGFR/FGFR | Ovary cancer |
| NCT05130515 | NCT05130515-0 | Niraparib | PARP | Ovary cancer |
| NCT05135845 | NCT05135845-0 | Capmatinib | MET | Stomach cancer |
| NCT05135845 | NCT05135845-0 | Spartalizumab | PD-1 | Stomach cancer |
| NCT05137262 | NCT05137262-0 | Chemotherapy | chemotherapy | Gallbladder and biliary tract cancer |
| NCT05137262 | NCT05137262-0 | Durvalumab | PD-L1 | Gallbladder and biliary tract cancer |
| NCT05147493 | NCT05147493-0 | Isatuximab | CD38 | Others |
| NCT05147493 | NCT05147493-0 | Bortezomib | PSMB | Others |
| NCT05147493 | NCT05147493-0 | Cyclophosphamide | chemotherapy | Others |
| NCT05147493 | NCT05147493-0 | Dexamethasone | Steroid Treatment | Others |
| NCT05157542 | NCT05157542-0 | Durvalumab | PD-L1 | lung cancers |
| NCT05157542 | NCT05157542-0 | Paclitaxel | chemotherapy | lung cancers |
| NCT05162196 | NCT05162196-0 | Niraparib | PARP | lung cancers |
| NCT05162196 | NCT05162196-0 | Toripalimab | PD-1 | lung cancers |
| NCT05162872 | NCT05162872-0 | Niraparib | PARP | Mouth and oropharynx cancers |
| NCT05162872 | NCT05162872-0 | Sintilimab | PD-1 | Mouth and oropharynx cancers |
| NCT05164770 | NCT05164770-1 | Zanubrutinib | BTK | Lymphomas |
| NCT05164770 | NCT05164770-1 | Rituximab | CD20 | Lymphomas |
| NCT05164770 | NCT05164770-1 | Cyclophosphamide | chemotherapy | Lymphomas |
| NCT05164770 | NCT05164770-1 | Doxorubicin | chemotherapy | Lymphomas |
| NCT05164770 | NCT05164770-1 | Vincristine | chemotherapy | Lymphomas |
| NCT05164770 | NCT05164770-1 | Prednisone | Steroid Treatment | Lymphomas |
| NCT05164770 | NCT05164770-2 | Zanubrutinib | BTK | Lymphomas |
| NCT05164770 | NCT05164770-2 | Rituximab | CD20 | Lymphomas |
| NCT05164770 | NCT05164770-2 | Epirubicin | chemotherapy | Lymphomas |
| NCT05164770 | NCT05164770-2 | Cyclophosphamide | chemotherapy | Lymphomas |
| NCT05164770 | NCT05164770-2 | Vincristine | chemotherapy | Lymphomas |
| NCT05164770 | NCT05164770-2 | Prednisone | Steroid Treatment | Lymphomas |
| NCT05164770 | NCT05164770-3 | Zanubrutinib | BTK | Lymphomas |
| NCT05164770 | NCT05164770-3 | Rituximab | CD20 | Lymphomas |
| NCT05164770 | NCT05164770-3 | Methotrexate | chemotherapy | Lymphomas |
| NCT05170334 | NCT05170334-0 | Binimetinib | MEK | Melanoma |
| NCT05170334 | NCT05170334-0 | Belinostat | chemotherapy | Melanoma |
| NCT05171387 | NCT05171387-0 | Darolutamide | Endocrine Therapy | Prostate cancer |
| NCT05171387 | NCT05171387-0 | Androgen Deprivation Therapy (Adt) | Endocrine Therapy | Prostate cancer |
| NCT05179733 | NCT05179733-0 | Zanubrutinib | BTK | Lymphomas |
| NCT05179733 | NCT05179733-0 | Rituximab | CD20 | Lymphomas |
| NCT05179733 | NCT05179733-0 | Lenalidomide | CRBN | Lymphomas |
| NCT05187338 | NCT05187338-0 | Ipilimumab | CTLA-4 | Other solid tumors |
| NCT05187338 | NCT05187338-0 | Pembrolizumab | PD-1 | Other solid tumors |
| NCT05187338 | NCT05187338-0 | Durvalumab | PD-L1 | Other solid tumors |
| NCT05190445 | NCT05190445-1 | Cinrebafusp Alfa | HER2/4-1BB | Stomach cancer |
| NCT05190445 | NCT05190445-1 | Ramucirumab | KDR | Stomach cancer |
| NCT05190445 | NCT05190445-1 | Paclitaxel | chemotherapy | Stomach cancer |
| NCT05190445 | NCT05190445-2 | Cinrebafusp Alfa | HER2/4-1BB | Stomach cancer |
| NCT05190445 | NCT05190445-2 | Tucatinib | HER2 | Stomach cancer |
| NCT05205252 | NCT05205252-1 | Tafasitamab | CD19 | Other hematologic Neoplasms |
| NCT05205252 | NCT05205252-1 | Lenalidomide | CRBN | Other hematologic Neoplasms |
| NCT05205252 | NCT05205252-1 | Tazemetostat | EZH2 | Other hematologic Neoplasms |
| NCT05205252 | NCT05205252-2 | Lenalidomide | CRBN | Other hematologic Neoplasms |
| NCT05205252 | NCT05205252-2 | Tazemetostat | EZH2 | Other hematologic Neoplasms |
| NCT05205252 | NCT05205252-3 | Tazemetostat | EZH2 | Other hematologic Neoplasms |
| NCT05205252 | NCT05205252-3 | Acalabrutinib | BTK | Other hematologic Neoplasms |
| NCT05214183 | NCT05214183-0 | Acalabrutinib | BTK | Lymphomas |
| NCT05214183 | NCT05214183-0 | Rituximab | CD20 | Lymphomas |
| NCT05216432 | NCT05216432-0 | Rly-2608 | PI3K | Breast cancer |
| NCT05216432 | NCT05216432-0 | Fulvestrant | Endocrine Therapy | Breast cancer |
