# Supplementary material for: A target map of clinical combination therapies in oncology: an analysis of clinicaltrials.gov
Source: Discov Oncol. 2023 Aug 21;14:151. doi: 10.1007/s12672-023-00758-4 (PMC10441974; doi:10.1007/s12672-023-00758-4)
Supplement: Supplementary file 3 — (DOCX 26 KB) [file 12672_2023_758_MOESM3_ESM.docx]

Supplementary Table 3 The approved combination therapies in oncology by FDA

| Approval date | Combination reagents | Target/treatment type | Indication |
| --- | --- | --- | --- |
| 9/02/2022 | Durvalumab + gemcitabine + cisplatin | PD-L1+chemotherapy | Locally advanced or metastatic biliary tract cancer |
| 8/5/2022 | Darolutamide + docetaxel | AR+chemotherapy | Metastatic hormone-sensitive prostate cancer |
| 6/22/2022 | Dabrafenib + trametinib | BRAF+MEK | Unresectable or metastatic solid tumors with BRAF V600E mutation |
| 5/27/2022 | Nivolumab + fluoropyrimidine- and platinum-based chemotherapy;  Nivolumab + ipilimumab | PD-1+chemotherapy;  PD-1+CTLA-4 | First-line treatment of esophageal squamous cell carcinoma |
| 5/25/2022 | Ivosidenib + azacitidine | IDH+chemotherapy | Newly diagnosed acute myeloid leukemia |
| 3/18/2022 | Nivolumab + relatlimab | PD-1+LAG3 | Unresectable or metastatic melanoma |
| 3/4/2022 | Nivolumab + platinum-doublet chemotherapy | PD-1+chemotherapy | Early-stage NSCLC |
| 12/2/2021 | Rituximab + chemotherapy | CD20+chemotherapy | CD20-positive DLBCL, BL,BLL or B-AL |
| 12/01/2021 | Daratumumab + hyaluronidase-fihj + carfilzomib + dexamethasone | CD38+PSMB+hyaluronidase+steroid | Multiple myeloma |
| 10/13/2021 | Pembrolizumab + chemotherapy + bevacizumab | PD-1+VEGF+chemotherapy | First-line treatment of cervical cancer |
| 10/12/2021 | Abemaciclib + endocrine therapy (tamoxifen or an aromatase inhibitor) | CDK+endocrine therapy | Early breast cancer |
| 8/10/2021 | Lenvatinib + pembrolizumab | VEGFR/FGFR/PDGFR+PD-1 | Advanced renal cell carcinoma |
| 7/26/2021 | Pembrolizumab + chemotherapy | PD-1+chemotherapy | High-risk early-stage triple-negative breast cancer |
| 7/21/2021 | Pembrolizumab + lenvatinib | VEGFR/FGFR/PDGFR+PD-1 | Advanced endometrial carcinoma |
| 7/9/2021 | Daratumumab + hyaluronidase-fihj + pomalidomide + dexamethasone | CD38+CRBN+hyaluronidase+steroid | Multiple myeloma |
| 5/5/2021 | Pembrolizumab + trastuzumab + fluoropyrimidine + platinum | PD-1+HER2+chemotherapy | HER2-positive gastric cancer |
| 4/16/2021 | Nivolumab + fluoropyrimidine + platinum | PD-1+chemotherapy | Metastatic gastric cancer and esophageal adenocarcinoma |
| 3/31/2021 | Isatuximab-irfc + carfilzomib + dexamethasone | CD38+PSMB+steroid | Multiple myeloma |
| 3/22/2021 | Pembrolizumab + platinum + fluoropyrimidine | PD-1+chemotherapy | Esophageal or GEJ carcinoma |
| 2/26/2021 | Melphalan flufenamide + dexamethasone | chemotherapy+steroid | Advanced renal cell carcinoma |
| 1/22/2021 | Nivolumab + cabozantinib | PD-1+MET/KDR/RET | Advanced renal cell carcinoma |
| 1/15/2021 | Daratumumab + hyaluronidase + bortezomib + cyclophosphamide + dexamethasone | CD38+PSMB+hyaluronidase+steroid+chemotherapy | Newly diagnosed light chain amyloidosis |
| 12/18/2020 | Selinexor + bortezomib + dexamethasone | XPO1+PSMB+steroid | Refractory or relapsed multiple myeloma |
| 12/16/2020 | Margetuximab-cmkb + chemotherapy | HER2+chemotherapy | Metastatic HER2-positive breast cancer |
| 11/25/2020 | Naxitamab + GM-CSF | GD-2+GM-CSF | High-risk neuroblastoma in bone or bone marrow |
| 11/13/2020 | Pembrolizumab + chemotherapy | PD-1+chemotherapy | Locally recurrent unresectable or metastatic triple negative breast cancer |
| 10/2/2020 | Nivolumab + ipilimumab | PD-1+CTLA-4 | Unresectable malignant pleural mesothelioma |
| 9/8/2020 | Atezolizumab + paclitaxel | PD-L1+chemotherapy | Breast cancer |
| 8/20/2020 | Carfilzomib + daratumumab + dexamethasone | CD38+PSMB+steroid | Multiple myeloma |
| 7/31/2020 | Tafasitamab-cxix + Lenalidomide | CD19+CRBN | DLBCL |
| 7/30/2020 | Atezolizumab + cobimetinib + vemurafenib | PD-L1+MEK2+BRAF | BRAF V600 unresectable or metastatic melanoma |
| 6/29/2020 | Pertuzumab + trastuzumab + hyaluronidase–zzxf | HER2+HER2+hyaluronidase | HER2-positive breast cancer |
| 5/29/2020 | Ramucirumab + erlotinib | KDR+EGFR | First-line treatment of metastatic NSCLC |
| 5/29/2020 | Atezolizumab + bevacizumab | PD-L1+VEGF | Unresectable hepatocellular carcinoma |
| 5/26/2020 | Nivolumab + ipilimumab + chemotherapy | PD-1+CTLA-4+chemotherapy | Metastatic NSCLC |
| 5/15/2020 | Nivolumab + ipilimumab | PD-1+CTLA-4 | First-line treatment of metastatic NSCLC (PD-L1 tumor expression ≥1%) |
| 5/8/2020 | Olaparib + bevacizumab | PARP+VEGF | Ovarian, fallopian tube, or primary peritoneal cancers |
| 5/1/2020 | Daratumumab + hyaluronidase-fihj | CD38+hyaluronidase | Multiple myeloma |
| 4/21/2020 | Ibrutinib + rituximab | BTK+CD20 | Chronic lymphocytic leukemia |
| 4/17/2020 | Tucatinib + trastuzumab + capecitabine | HER2+HER2+chemotherapy | HER2-positive metastatic breast cancer |
| 4/8/2020 | Encorafenib + cetuximab | BRAF+EGFR | Metastatic colorectal cancer with a BRAF V600E mutation |
| 3/30/2020 | Durvalumab + etoposide + carboplatin/cisplatin | PD-L1+chemotherapy | Extensive-stage small cell lung cancer |
| 3/10/2020 | Nivolumab + ipilimumab | PD-1+CTLA-4 | Hepatocellular carcinoma |
| 3/2/2020 | isatuximab-irfc+pomalidomide+dexamethasone | CD38+CRBN+steroid | Multiple myeloma |
| 2/25/2020 | Neratinib + capecitabine | EGFR+chemotherapy | Metastatic HER2-positive breast cancer |
| 12/3/2019 | Atezolizumab + paclitaxel + carboplatin | PD-L1+chemotherapy | Metastatic NSCLC with no EGFR or ALK genomic  tumor aberrations |
| 9/26/2019 | Daratumumab + bortezomib + thalidomide + dexamethasone | CD38+PSMB+hyaluronidase+CRBN+steroid | Multiple myeloma |
| 9/17/2019 | Pembrolizumab + lenvatinib | PD-1+VEGFR/FGFR/PDGFR | Advanced endometrial carcinoma |
| 7/3/2019 | Selinexor + dexamethasone | XPO1+steroid | Relapsed or refractory multiple myeloma |
| 6/27/2019 | Daratumumab + lenalidomide + dexamethasone | CD38+CRBN+steroid | Newly diagnosed multiple myeloma |
| 6/10/2019 | Polatuzumab vedotin + bendamustine + rituximab | CD79B+chemotherapy+CD20 | Relapsed or refractory DLBCL |
| 5/28/2019 | Lenalidomide + rituximab | CRBN+CD20 | Previously treated follicular lymphoma and previously treated marginal zone lymphoma |
| 5/24/2019 | Alpelisib + fulvestrant | PI3K+endocrine therapy | HR-positive, HER2-negative, PIK3CA-mutated, advanced or metastatic breast cancer |
| 5/14/2019 | Avelumab + axitinib | PD-L1+VEGFR | First-line treatment of advanced renal cell carcinoma (RCC) |
| 4/19/2019 | Pembrolizumab + axitinib | PD-1+VEGFR | First-line treatment of advanced renal cell carcinoma (RCC) |
| 3/18/2019 | Atezolizumab + carboplatin + etoposide | PD-L1+chemotherapy | first-line treatment of extensive-stage SCLC |
| 2/28/2019 | Trastuzumab + hyaluronidase | HER2+chemotherapy | HER2 overexpressing breast cancer |
| 12/6/2018 | Atezolizumab + bevacizumab + paclitaxel + carboplatin | PD-L1+VEGF+chemotherapy | First-line treatment of metastatic non-squamous NSCLC with no EGFR or ALK genomic tumor aberrations |
| 11/21/2018 | Venetoclax + azacitidine + decitabine + cytarabine | BCL+chemotherapy | Newly-diagnosed AML |
| 11/21/2018 | Glasdegib + cytarabine | SMO+chemotherapy | Newly-diagnosed AML |
| 11/16/2018 | Brentuximab vedotin + chemotherapy | CD30/MMAE+chemotherapy | Previously untreated systemic anaplastic large cell lymphoma or other CD30-expressing peripheral T-cell lymphomas |
| 10/20/2018 | Pembrolizumab + carboplatin + paclitaxel | PD-1+chemotherapy | First-line treatment of metastatic squamous NSCLC |
| 8/20/2018 | Pembrolizumab + pemetrexed + platinum | PD-1+chemotherapy | first-line treatment of metastatic, non-squamous NSCLC |
| 8/16/2018 | Pembrolizumab + atezolizumab | PD-1+PD-L1 | Locally advanced or metastatic urothelial cancer |
| 7/18/2018 | ribociclib+aromatase inhibitor | CDK+endocrine therapy | HR-positive, HER2-negative advanced or metastatic breast cancer |
| 7/10/2018 | Ipilimumab + nivolumab | PD-1+CTLA-4 | Microsatellite instability-high (MSI-H) or mismatch repair deficient (dMMR) metastatic colorectal cancer |
| 6/19/2018 | Tecentriq + Keytruda | PD-1+PD-L1 | Locally advanced or metastatic urothelial cancer |
| 6/27/2018 | Encorafenib + binimetinib | BRAF+MEK | Unresectable or metastatic melanoma with a BRAF V600E or V600K mutation |
| 6/13/2018 | Bevacizumab + carboplatin + paclitaxel | VEGF+chemotherapy | Epithelial ovarian, fallopian tube, or primary peritoneal cancer |
| 5/4/2018 | Dabrafenib + trametinib | BRAF+MEK | Anaplastic thyroid cancer with BRAF V600E mutation |
| 4/30/2018 | Dabrafenib + trametinib | BRAF+MEK | Melanoma with BRAF V600E or V600K mutations |
| 4/16/2018 | Nivolumab + ipilimumab | PD-1+CTLA-4 | Intermediate or poor risk, previously untreated advanced renal cell carcinoma |
| 5/20/2018 | Brentuximab vedotin + chemotherapy | CD30+chemotherapy | Previously untreated classical Hodgkin lymphoma |
| 2/26/2018 | Abemaciclib + aromatase inhibitor | CDK+endocrine therapy | HR-positive, HER2-negative advanced or metastatic breast cancer |
| 2/7/2018 | Abiraterone acetate + prednisone | CYP17+steroid | Metastatic high-risk castration-sensitive prostate cancer |
| 12/20/2017 | Pertuzumab + trastuzumab + chemotherapy | HER2+HER2+chemotherapy | Adjuvant treatment of patients with HER2-positive early breast cancer |
| 11/16/2017 | Obinutuzumab + chemotherapy | CD20+chemotherapy | Follicular lymphoma |
| 9/28/2017 | Abemaciclib + fulvestrant | CDK+endocrine therapy | HR-positive, HER2-negative advanced or metastatic breast cancer |
| 9/14/2017 | Cabazitaxel + prednisone | chemotherapy+steroid | Metastatic castration-resistant prostate cancer |
| 9/01/2017 | Gemtuzumab ozogamicin + daunorubicin + cytarabine | CD33+chemotherapy | CD33-positive AML |
| 8/3/2017 | Daunorubicin + cytarabine | chemotherapy | Newly-diagnosed therapy-related AML or AML with myelodysplasia-related changes |
| 6/22/2017 | Dabrafenib + trametinib | BRAF+MEK2 | Metastatic NSCLC with BRAF V600E mutation |
| 6/22/2017 | Rituximab + hyaluronidase | CD20+hyaluronidase | Follicular lymphoma, DLBCL, and CLL |
| 5/10/2017 | Pembrolizumab + pemetrexed + carboplatin | PD-1+chemotherapy | Previously untreated metastatic non-squamous NSCLC |
| 11/21/2016 | Daratumumab + lenalidomide + dexamethasone; daratumumab + bortezomib + dexamethasone | CD38+CRBN+steroid;  CD38+PSMB+steroid | Multiple myeloma |
| 5/13/2016 | Lenvatinib + everolimus | CRBN+mTOR | Advanced renal cell carcinoma |
| 2/26/2016 | Obinutuzumab + bendamustine | CD20+chemotherapy | Follicular lymphoma |
| 2/19/2016 | Palbociclib + fulvestrant | CDK+endocrine therapy | HR-positive, HER2-negative advanced or metastatic breast cancer |
| 11/30/2015 | Elotuzumab + lenalidomide + dexamethasone | SLAMF7+CRBN+steroid | Multiple myeloma |
| 11/24/2015 | Necitumumab + gemcitabine + cisplatin | EGFR+chemotherapy | First-line treatment of metastatic squamous NSCLC |
| 11/20/2015 | Trametinib + dabrafenib | BRAF+MEK2 | Unresectable or metastatic melanoma with BRAF V600E or V600K mutations |
| 11/20/2015 | Ixazomib + lenalidomide + dexamethasone | PSMB+CRBN+steroid | Multiple myeloma |
| 11/10/2015 | Cobimetinib + vemurafenib | BRAF+MEK | Unresectable or metastatic melanoma with BRAF V600E or V600K mutation |
| 10/22/2015 | Irinotecan +5-FU + leucovorin | chemotherapy | Metastatic adenocarcinoma |
| 9/30/2015 | Nivolumab + ipilimumab | PD-1+CTLA-4 | BRAF V600 wild-type, unresectable or metastatic melanoma |
| 7/24/2015 | Carfilzomib + lenalidomide + dexamethasone | PSMB+CRBN+steroid | Relapsed multiple myeloma |
| 4/24/2015 | Ramucirumab + FOLFIRI | KDR+chemotherapy | Metastatic colorectal cancer |
| 3/10/2015 | Dinutuximab + GM-CSF + IL-2 + 13-cis-retinoic acid | GD2+GM-CSF+IL-2 | High-risk neuroblastoma |
| 2/23/2015 | Panobinostat + bortezomib + dexamethasone | HDAC+PSMB+steroid | Multiple myeloma |
| 2/3/2015 | Palbociclib + letrozole | CDK+chemotherapy | ER-positive, HER2-negative advanced breast cancer |
| 12/12/2014 | Ramucirumab + docetaxel | KDR+chemotherapy | Metastatic NSCLC |
| 11/14/2014 | Bevacizumab + paclitaxel + doxorubicin; bevacizumab + paclitaxel + topotecan | VEGF+chemotherapy | Recurrent epithelial ovarian, fallopian tube, or primary peritoneal cancer |
| 11/5/2014 | Ramucirumab + paclitaxel | KDR+chemotherapy | Advanced gastric or GEJ adenocarcinoma |
| 8/14/2014 | Bevacizumab + paclitaxel + cisplatin; bevacizumab + paclitaxel + topotecan | VEGF+chemotherapy | Persistent, recurrent or metastatic cervical cancer |
| 7/23/2014 | Idelalisib + rituximab | PI3K+CD20 | Relapsed CLL |
| 4/17/2014 | Ofatumumab + chlorambucil | CD20+chemotherapy | CLL |
| 1/10/2014 | Trametinib + dabrafenib | BRAF+MEK | Unresectable or metastatic melanoma with a BRAF V600E or V600K mutation |
| 11/1/2013 | Obinutuzumab + chlorambucil | CD20+chemotherapy | Previously untreated CLL |
| 9/30/2013 | Pertuzumab + trastuzumab + docetaxel | HER2+HER2+chemotherapy | HER2-positive, locally advanced, inflammatory, or early stage breast cancer |
| 9/6/2013 | Paclitaxel protein-bound + gemcitabine | chemotherapy | First-line treatment of metastatic adenocarcinoma of the pancreas |
| 1/23/2013 | Bevacizumab + fluoropyrimidine + irinotecan;  bevacizumab + fluoropyrimidine+oxaliplatin | VEGF+chemotherapy | Metastatic colorectal cancer |

Abbreviates: AML: acute myeloid leukemia; B-AL: mature B-cell acute leukemia; BL: Burkitt lymphoma; BLL: Burkitt-like lymphoma; CLL: chronic lymphocytic leukemia; CRC: colorectal cancer; DLBCL: diffuse large B-cell lymphoma; GEJ: gastroesophageal junction; NSCLC:non-small cell lung cancer; RCC: renal cell carcinoma; SCLC: small cell lung cancer.
