# Supplementary material for: A target map of clinical combination therapies in oncology: an analysis of clinicaltrials.gov
Source: Discov Oncol. 2023 Aug 21;14:151. doi: 10.1007/s12672-023-00758-4 (PMC10441974; doi:10.1007/s12672-023-00758-4)
Supplement: Supplementary file 4 — (DOCX 24 KB) [file 12672_2023_758_MOESM4_ESM.docx]

Supplementary Table 4 The collected Bispecific antibodies (bsAbs) or Bispecific T-cell engager (BiTE) approved or under clinical investigation

| Drug | The highest R&D status | Target | Key indication | Company |
| --- | --- | --- | --- | --- |
| Teclistamab | FDA approved | BCMA x CD3 | Haematological malignancies | Janssen |
| Amivantamab | FDA approved | EGFR x MET | Solid Tumors(NSCLC) | Janssen |
| Blinatumomab | FDA approved | CD19 x CD3 | Haematological malignancies | Amgen |
| Cadonilimab | NMPA Approved | PD-1 x CTLA4 | Solid Tumors | Akeso Biopharma |
| Mosunetuzumab | EMA Approved | CD20 x CD3 | Haematological malignancies | Roche/Genentech/ Chugai/Biogen |
| Talquetamab | Phase III | GPRC5D x CD3 | Haematological malignancies | Janssen |
| Ivonescimab (AK-112) | Phase III | PD-1 x VEGFR; | Solid Tumors | Akeso Biopharma |
| EMB-01 | Phase III | EGFR x MET | Solid Tumors (NSCLC) | EpimAb Biotherapeutics |
| KN-026 | Phase III | HER2 x HER2 | Solid Tumors | Alphamab Oncology |
| Zanidatamab | Phase III | HER2 x HER2 | Solid Tumors | Zymeworks/BeiGene |
| Epcoritamab | Phase III | CD20 x CD3; | Haematological malignancies | Genmab |
| Catumaxomab | Phase III | EpCAM x CD3 | Solid Tumors (Stomach cancer) | Neovii Biotech/Trion Research |
| Glofitamab | Phase III | CD20 x CD3; | Haematological malignancies | Roche |
| Tebotelimab | Phase III | PD-1 x LAG-3 | Solid Tumors (Stomach cancer) | Macrogenics |
| SI-B001 | Phase III | HER3 x EGFR | Solid Tumors (NSCLC) | Biokin pharmaceutical |
| Erfonrilimab (KN-046) | Phase III | PD-L1 x CTLA4 | Solid Tumors (NSCLC) | Alphamab Oncology |
| SHR-1701 | Phase III | PD-L1 x TGF-β | Solid Tumors | Hengrui Pharmaceuticals |
| Bintrafusp alfa | Phase III | PD-L1 x TGF-β | Solid Tumors | Merck KGaA |
| Elranatamab | Phase III | BCMA x CD3 | Haematological malignancies | Pfizer |
| Navicixizumab | Phase III | VEGF x DLL4 | Solid Tumors | Oncomed Pharmaceuticals |
| Acapatamab | Phase II, suspended | PSMA x CD3 | Solid Tumors (prostate cancer) | Amgen |
| HX-009 | Phase II | PD-1 x CD47 | Solid Tumors | Waterstone Hanxbio Pty Ltd |
| REGN-4018 | Phase II | MUC16 x CD3 | Solid Tumors | Regeneron |
| REGN-5668 | Phase II | MUC16 x CD28 | Solid Tumors | Regeneron |
| REGN-7075 | Phase II | EGFR x CD28 | Solid Tumors | Regeneron |
| EMB-02 | Phase II | PD-1 x LAG-3 | Solid Tumors | EpimAb Biotherapeutics |
| EMB-06 | Phase II | BCMA x CD3 | Haematological malignancies | EpimAb Biotherapeutics |
| 6MW3211 | Phase II | CD47 x PD-L1 | Solid Tumors | Mabwell Bioscience |
| NVG-111 | Phase II | ROR1 x CD3 | Haematological malignancies | NovalGen Ltd |
| NGM-707 | Phase II | LILRB1 x LIRB2 | Solid Tumors | Merck & Co/Ngm Biopharmaceuticals; |
| REGN-5458 | Phase II | BCMA x CD3 | Haematological malignancies | Regeneron |
| AZD-2936 | Phase II | PD-1 x TIGIT | Solid Tumors (NSCLC) | AstraZeneca |
| GEN-1044 | Phase II | 5T4 x CD3 | Solid Tumors | Genmab |
| GEN-1044 | Phase II | 5T4 x CD3 | Solid Tumors | Genmab |
| REGN-5459 | Phase II | BCMA x CD3 | Haematological malignancies | Regeneron |
| GEN-1046 | Phase II | PD-L1 x 4-1BB | Solid Tumors | Genmab |
| FS 118 | Phase II | PD-L1 x LAG-3 | Solid Tumors | Merck KGaA |
| IBI318 | Phase II | PD-1 x PD-L1 | Haematological malignancies | Innovent |
| GB-261 | Phase II | CD20 x CD3 | Haematological malignancies | Genor Biopharma |
| GEN-1042 | Phase II | CD40 x 4-1BB | Solid Tumors | Genmab |
| ES101 | Phase II | PD-L1 x 4-1BB | Solid Tumors | Inhibrx |
| Zenocutuzumab | Phase II | HER2 x HER3 | Solid Tumors | Merus |
| Cibisatamab | Phase II | CEA x CD3 | Solid Tumors | Roche |
| RO-7121661 | Phase II | PD-1 x TIM-3 | Solid Tumors | Roche |
| AFM-24 | Phase II | EGFR x CD16A | Solid Tumors | Affimed |
| Odronextamab | Phase II | CD20 x CD3 | Haematological malignancies | Regeneron |
| REGN-5678 | Phase II | PSMA x CD28 | Solid Tumors (prostate cancer) | Regeneron |
| MCLA-129 | Phase II | EGFR×MET | Solid Tumors | Merus |
| Nivatrotamab | Phase II | GD2 x CD3 | Solid Tumors | Memorial Sloan Kettering Cancer Center |
| XmAb20717 | Phase II | PD-1 x CTLA4 | Solid Tumors | Xencor |
| GNR-084 | Phase II | CD19 x CD3 | Haematological malignancies | Generium Pharmaceuticals/Iontas |
| Vanucizumab | Phase II | VEGFA×ANGPT2 | Solid Tumors (CRC) | Roche |
| XmAb-18087 | Phase II | SSTR2 x CD3 | Solid Tumors | Xencor |
| RO-7247669 | Phase II | PD-1 x LAG-3 | Solid Tumors | Roche |
| AFM-13 | Phase II | CD16A x CD3 | Haematological malignancies | Affimed GmbH |
| Cevostamab | Phase II | FcRH5 x CD3 | Haematological malignancies | Genentech |
| Plamotamab | Phase II | CD20 x CD3 | Haematological malignancies | Xencor |
| Cinrebafusp alfa | Phase II | HER2 x 4-1BB | Solid Tumors | Pieris Pharmaceuticals |
| Tarlatamab | Phase II | DLL3 x CD3 | Solid Tumors | Amgen |
| Vibecotamab | Phase II | CD123 x CD3 | Haematological malignancies | Xencor |
| Flotetuzumab | Phase II | CD123 x CD3 | Haematological malignancies | Macrogenics |
| Orlotamab | Phase I, suspended | CD276 x CD3 | Solid Tumors | Macrogenics |
| Pavurutamab | Phase I, suspended | BCMA x CD3 | Haematological malignancies | Amgen |
| PM-8001 | Phase I | PD-L1 x TGF-β | Solid Tumors | Biotheus Inc. |
| ABL001 | Phase I | VEGF × DLL4 | Solid Tumors | Abl Bio |
| ERY-974 | Phase I | GPC3 x CD3 | Solid Tumors (HCC) | Chugai Pharmaceutical |
| INBRX-105 | Phase II | PD-L1 x 4-1BB | Haematological malignancies/Solid Tumors | Inhibrx Inc |
| AMG-424 | Phase I | CD38 x CD3 | Haematological malignancies | Amgen |
| AMG-596 | Phase I | EGFRvIII x CD3 | Solid Tumors | Amgen |
| MBS-301 | Phase I | HER2 × HER2 | Solid Tumors | Beijing Mabworks Biotech |
| EX-103 | Phase I | CD20 x CD3 | Haematological malignancies | ExcelMab Inc. |
| BJ-005 | Phase I | PD-L1 x TGF-β | Solid Tumors | BJ Bioscience, Inc. |
| Y-150 | Phase I | CD38 x CD3 | Haematological malignancies | Wuhan YZY Biopharma |
| SG12473 | Phase I | PD-L1 x CD47 | Haematological malignancies | Sumgen Biotech |
| PM-1003 | Phase I | PD-L1 x 4-1BB | Solid Tumors | Biotheus Inc. |
| IBI319 | Phase I | PD-1 x 4-1BB | Solid Tumors | Innovent |
| IMM-2510 | Phase I | PD-L1 x VEGF | Solid Tumors | ImmuneOnco Biopharmaceuticals |
| HB-0025 | Phase I | PD-L1 x VEGF | Solid Tumors | Huabo Biopharm |
| SKB-337 | Phase I | PD-L1 x CTLA4 | Solid Tumors | KELUN Pharmaceutical |
| CKD-702 | Phase I | EGFR×MET | Solid Tumors (NSCLC) | Chong Kun Dang Pharmaceutical |
| Q-1802 | Phase I | Claudin 18.2 × PD-L1 | Solid Tumors | QureBio Ltd. |
| IMM2505 | Phase I | PD-L1 x CD47 | Solid Tumors | SunHo Biopharmaceutical |
| QLS31901 | Phase I | PD-L1 x TGF-β | Solid Tumors | Qilu Pharmaceutical |
| JMT-601 | Phase I | CD20 x CD47 | Haematological malignancies | Conjupro Biotherapeutics |
| M-1231 | Phase I | EGFR × MUC1 | Solid Tumors | Merck KGaA |
| ABL-503 | Phase I | PD-L1 x 4-1BB | Solid Tumors | Abl Bio |
| JS201 | Phase II | PD-1 x TGF-β | Solid Tumors | Junshi Bioscience |
| Y-101D | Phase I | PD-L1 x TGF-β | Solid Tumors | Wuhan YZY Biopharma. |
| IBI-321 | Phase I | PD-1 x TIGIT | Solid Tumors | Innovent |
| TJ-CD4B | Phase I | Claudin 18.2 × 4-1BB | Solid Tumors | I-MAB Biopharma |
| TST-005 | Phase I | PD-L1 x TGF-β | Solid Tumors | Suzhou Transcenta Therapeutics |
| LBL-015 | Phase I | PD-1 x TGF-β | Solid Tumors | Nanjing Leads Biolabs |
| IMM-2902 | Phase I | HER2 × CD47 | Solid Tumors | ImmuneOnco Biopharmaceuticals |
| RO-7300490 | Phase I | FAPα × CD40 | Solid Tumors | Roche |
| RGV-004 | Phase I | CD19 x CD3 | Haematological malignancies | Hangzhou Rongu Biotechnology |
| TQB-2825 | Phase I | CD20 x CD3 | Haematological malignancies | Chia Tai Tianqing Pharmaceutical Group |
| JNJ-78278343 | Phase I | KLK2 x CD3 | Solid Tumors (Prostate cancer) | Janssen |
| PF-07257876 | Phase I | PD-L1 x CD47 | Solid Tumors | Pfizer |
| hEGFRvIII-CD3 Bi-scFv | Phase I | EGFRvIII x CD3 | Solid Tumors | Duke University |
| SSGJ-705 | Phase I | HER2 × PD-1 | Solid Tumors | Sunshine Guojian Pharmaceutical |
| SMET-12 | Phase I | EGFR x CD3 | Solid Tumors | Zhejiang Shimai Pharmaceutical |
| CM-355 | Phase I | CD20 x CD3 | Haematological malignancies | Beijing InnoCare Pharma Tech |
| TNB-585 | Phase I | PSMA x CD3 | Solid Tumors (Prostate cancer) | Teneobio |
| FS-222 | Phase I | PD-L1 x 4-1BB | Solid Tumors | F-star Beta |
| JNJ-78306358 | Phase I | Human leukocyte antigen (HLA-G) x CD3 | Solid Tumors | Janssen |
| RG-6232 | Phase I | TYRP1 x CD3 | Solid Tumors | Roche |
| GBR-1342 | Phase I | CD38 x CD3 | Haematological malignancies | Glenmark |
| AMG-910 | Phase I | Claudin 18.2 x CD3 | Solid Tumors | Amgen |
| AGEN-1777 | Phase I | TIGIT × undisclosed target | Solid Tumors | Agenus Inc |
| ATG-101 | Phase I | PD-L1 x 4-1BB | Haematological malignancies/Solid Tumors | Antengene Biologics Limited |
| Duvortuxizumab | Phase I | CD19 x CD3 | Haematological malignancies | Janssen |
| AMG-509 | Phase I | STEAP1 x CD3 | Solid Tumors (Prostate cancer) | Amgen |
| IBI-322 | Phase I | PD-L1 x CD47 | Haematological malignancies/Solid Tumors | Innovent |
| MGD-007 | Phase I | GPA33 x CD3 | Solid Tumors (CRC) | Macrogenics |
| IMM-0306 | Phase I | CD20 x CD47 | Haematological malignancies | ImmuneOnco Biopharmaceuticals |
| MEDI-5752 | Phase I | PD-1 x CTLA4 | Solid Tumors | Medimmune Llc |
| BI-905711 | Phase I | TRAILR2 x CDH17 | Solid Tumors | Boehringer Ingelheim |
| CC-93269 | Phase I | BCMA x CD3 | Haematological malignancies | Celgene Corp; |
| AMG-199 | Phase I | MUC17 x CD3 | Solid Tumors (Gastric) | Amgen |
| CC-1 | Phase I | PSMA x CD3 | Solid Tumors (Prostate cancer) | Deutsches Krebsforschungszentrum |
| M-802 | Phase I | HER2 x CD3 | Solid Tumors | Wuhan YZY Biopharma |
| AMG-562 | Phase I | CD19 x CD3 | Haematological malignancies | Amgen |
| RG-6194 | Phase I | HER2 x CD3 | Solid Tumors | Roche/Genentech |
| TG-1801 | Phase I | CD19 x CD47 | Haematological malignancies | Novimmune |
| XmAb-23104 | Phase I | PD-1 x ICOS | Solid Tumors | Xencor |
| ABBV-428 | Phase I | Mesothelin x CD40 | Solid Tumors | AbbVie |
| MGD-019 | Phase I | PD-1 x CTLA4 | Solid Tumors | Macrogenics |
| K193 | Phase I | CD19 x CD3 | Haematological malignancies | Beijing Luzhu Biotechnology Co., Ltd |
| TNB-383B | Phase I | BCMA x CD3 | Haematological malignancies | Teneobio |
| ATOR-1015 | Phase I | OX40 x CTLA4 | Solid Tumors | Alligator Bioscience |
| MCLA-145 | Phase I | PD-L1 x 4-1BB | Solid Tumors | Incyte Corp/Merus |
| XmAb-22841 | Phase I | LAG-3 x CTLA4 | Solid Tumors | Xencor |
| AFM-11 | Phase I | CD19 x CD3 | Haematological malignancies | Affimed |
| AMV-564 | Phase I | CD33 x CD3 | Haematological malignancies/Solid Tumors | Amphivena Therapeutics |
| FS-120 | Phase I | OX40 x 4-1BB | Solid Tumors | F-Star |
| PF-06671008 | Phase I | P-cadherin x CD3 | Solid Tumors | Macrogenics/Pfizer |
| MCLA-158 | Phase I | EGFR x LGR5 | Solid Tumors (CRC) | Merus |
| IGM-2323 | Phase I | CD20 x CD3 | Haematological malignancies | Igm Biosciences |
| SI-B003 | Phase I | PD-1 x CTLA4 | Solid Tumors | Biokin pharmaceutical |
| A-319 | Phase I | CD19 x CD3 | Haematological malignancies | Evive Biotech |
| MCLA-117 | Phase I | CLEC12A x CD3 | Haematological malignancies | Merus Nv |
| RG-6007 | Phase I | HLA-A2-WT1 x CD3 | Haematological malignancies | Roche |
| APVO436 | Phase I | CD123 x CD3 | Haematological malignancies | Aptevo |
| AMG-330 | Phase I | CD33 x CD3 | Haematological malignancies | Amgen |
| JNJ-67571244 | Phase I | CD33 x CD3 | Haematological malignancies | Janssen |
| JNJ-63709178 | Phase I | CD123 x CD3 | Haematological malignancies | Janssen |
| GEM-333 | Phase I | CD33 x CD3 | Haematological malignancies | Gemoab Monoconals |
| AMG-427 | Phase I | FLT3 x CD3 | Haematological malignancies | Amgen |
| AMG-673 | Phase I | CD33 x CD3 | Haematological malignancies | Amgen |

Abbreviates: CRC: colorectal cancer; HCC: Hepatocellular carcinoma; NMPA: National Medical Products Administration (China); NSCLC:non-small cell lung cancer.
